# Dibenzotropylium-Capped Orthogonal Geometry Enabling Isolation and Examination of a Series of Hydrocarbons with Multiple 14π-Aromatic Units

**DOI:** 10.1021/jacs.2c12574

**Published:** 2023-01-06

**Authors:** Yuki Hayashi, Shuichi Suzuki, Takanori Suzuki, Yusuke Ishigaki

**Affiliations:** † Department of Chemistry, Faculty of Science, Hokkaido University, Sapporo 060-0810, Japan; ‡ Graduate School of Engineering Science, Osaka University, Osaka 560-8531, Japan

## Abstract

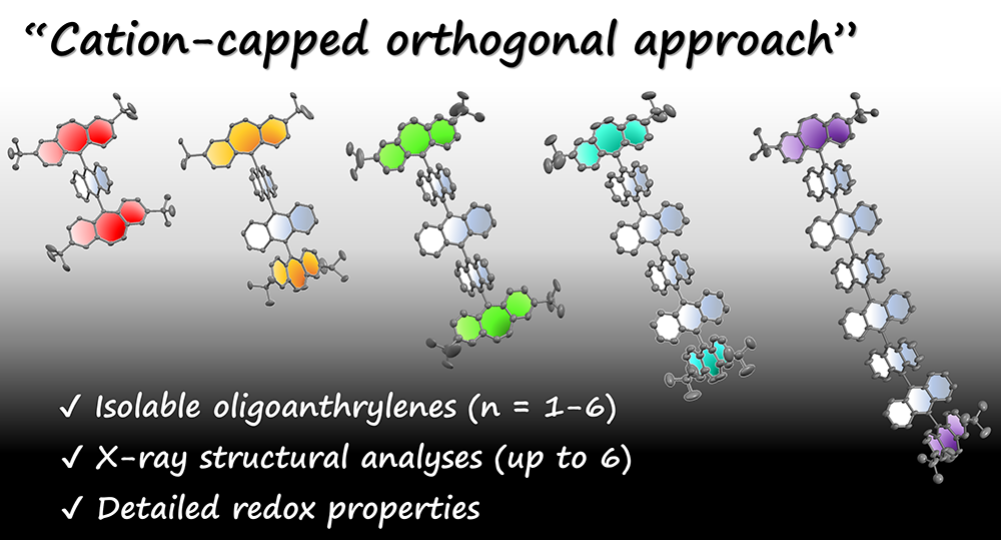

A series of six dications composed of pure hydrocarbons with one to six non-substituted 9,10-anthrylene units end-capped with two dibenzotropyliums were designed and synthesized to elucidate the electronic properties of huge oligo(9,10-anthrylene) backbones. Their structures were successfully determined by X-ray analyses even in the case of eight planar 14π-electron units, revealing that all dications adopt almost orthogonally twisted structures between neighboring units. Spectroscopic and voltammetric analyses show that neither the significant overlap of orbitals nor the delocalization of electrons between 14π-electron units occurs due to the orthogonally twisted geometry even in solution. As a result, sequential oxidation processes were observed with the reversible formation of multivalent cations with the release of the same number of electrons as the number of anthrylene units. Upon two-electron reduction, a closed-shell butterfly-shaped form was obtained from the dication containing one anthrylene unit, whereas open-shell twisted biradicals were isolated as stable entities in the cases of derivatives containing three to six anthrylene units. Notably, from the derivative with two anthrylene units, a metastable open-shell isomer was obtained quantitatively and underwent slow thermal conversion to the most stable closed-shell isomer (*E*_a_ = 23.1 kcal mol^–1^). There is a drastic change in oxidation potentials between two neutral species (Δ*E* = 1.32 V in CH_2_Cl_2_). Since the present dications were regenerated upon oxidation of the isolated reduction products, these systems may contribute to the development of advanced response systems capable of switching color, magnetic properties, and oxidative properties by using a “cation-capped orthogonal geometry”.

## Introduction

Rigid and planar π-conjugated carbon scaffolds are important components that can determine the fundamental characteristics of organic molecules, such as their geometries and physical properties. The arrangement of carbon atoms involving π-conjugation and the modification of substituents and fused-ring structures responsible for localization and/or delocalization of π-electrons can allow us to control properties such as color, luminescence behavior, electrochemical properties, magnetism, and reactivity of the molecules. The linking of several π-conjugated carbon skeletons, especially those in which the same units are connected by C–C single bonds, is of special interest since unique properties that are not possessed by the original skeleton can appear due to the accumulation of simple π-conjugated scaffolds.^1−5^ For example, oligophenylene-^6−15^ and oligonaphthylene^16−23^-based frameworks with various topologies such as linear and cyclic structures have been developed by accumulating simple planar π-systems (benzene and naphthalene, respectively), which has led to the discovery of numerous functions and physical properties.

**Scheme 1:**
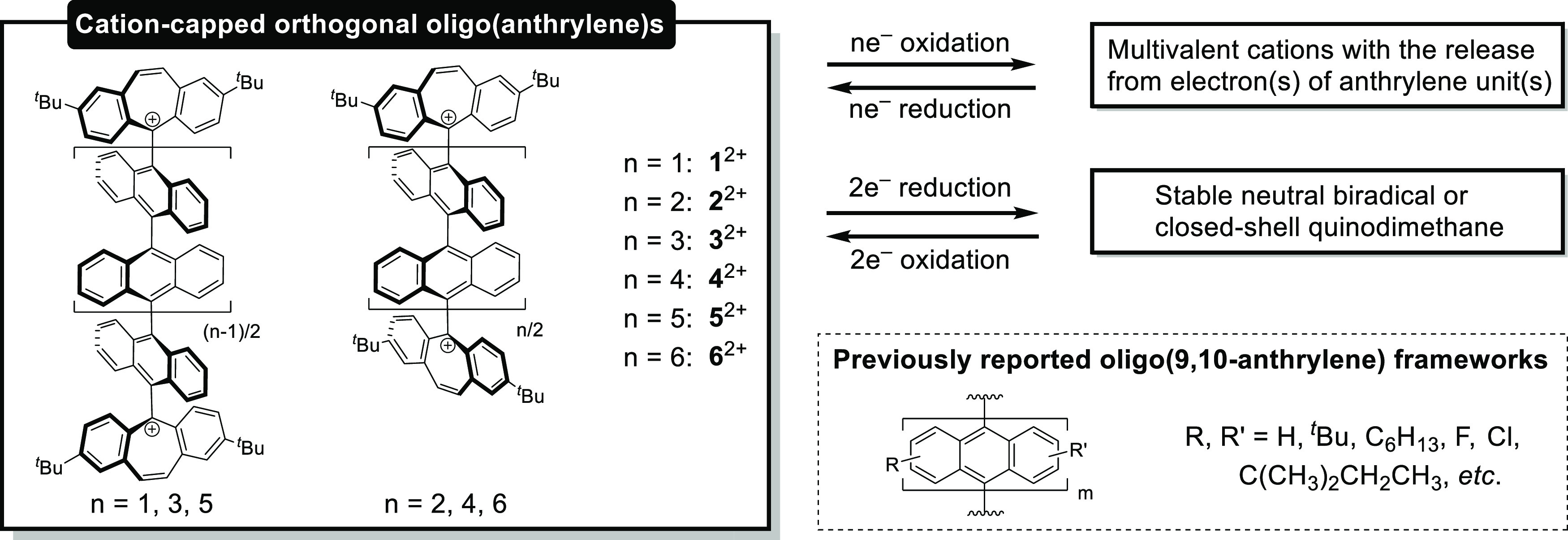
Design Concept, “Cation-Capping Approach with an Orthogonally Twisted Geometry”

Anthracene is a highly attractive carbon π-skeleton with intrinsic photophysical and electrochemical properties that has attracted attention since its discovery in 1832.^24^ It has been widely used as an essential component in the development of smart functional materials such as organic light-emitting diodes, organic solar cells, and organic thin-film transistors.^25−27^ Since anthracene can be a key building block for a variety of organic molecules, several oligoanthrylenes [X–(C_14_H_8_)_*m*_–Y] with multiple anthracenes linked at arbitrary positions have been reported.^28−30^ Especially, 9,9′-bianthracene (*m* = 2)^31−34^ and its derivatives,^35−39^ in which two anthracene units are directly linked to each other at the 9-position (Scheme 1), have been investigated for their unique physical properties such as their emission behavior based on twisted intramolecular charge transfer states in their excited states.^40^

Compared to the plethora of studies on bianthracenes, there have been only a few reported examples of oligo(9,10-anthrylene) derivatives of *m* ≥ 3, in which anthracene units are connected at 9,10-positions in a linear manner. In the 1990s, Müllen *et al*. synthesized several derivatives (*m* = 3 and 4) and reported their photophysical properties, such as the absorption and emission behavior of neutral species.^41−45^ The magnetic properties of the corresponding anion radical species obtained upon treatment with potassium metal showed that high-spin states are stabilized due to the orthogonally twisted structure between anthrylene units, which can suppress electron delocalization over the connected anthrylene units. In addition, several other derivatives with three or four anthrylene units have been studied by Baumgarten *et al*.,^46^ Ajibade and Adeloye,^47,48^ Wu *et al*.,^49^ Kubo *et al*.,^50^ and Ruffieux *et al*.^51,52^ and have recently been used as starting materials for the bottom-up synthesis of nanocarbon materials by Kubo *et al*.,^53,54^ Yamada *et al.*,^55^ and Amsharov *et al*.^56^ However, most of these structures have only been determined by mass spectrometry (MS) and/or NMR measurements. There are no reports in which the geometrical features of oligo(9,10-anthrylene)s with more than three anthrylene units have been determined by X-ray analyses. A much longer analogue was reported, though its NMR spectrum is rather broad and its identity was just confirmed only by field desorption (FD) MS.^42^ In addition, while oligophenylenes and oligonaphthylenes without substituents on the π-skeletons are soluble enough to be isolated and were investigated in detail, the introduction of multiple substituents on the anthrylene skeletons is required to make the previously reported oligo(9,10-anthrylene)s soluble so that the effects of attached substituents must always be taken into account when considering the properties of longer derivatives. Therefore, there have been no studies on the redox behavior of oligo(9,10-anthrylene)s, especially for longer derivatives of *m* > 4, without substituents on the anthrylenes. It is still challenging to evaluate the relationship between the number of anthrylene units and the redox behavior of oligoanthrylenes. For this purpose, a new concept is necessary for the molecular design of oligo(9,10-anthrylene)s, which would enable detailed spectroscopic and voltammetric analyses for elongated analogues (e.g., *m* = 6) by providing high solubility as well as crystallographic analyses by providing high crystallinity.

In this study, a series of dications **1**^2+^, **2**^2+^, **3**^2+^, **4**^2+^, **5**^2+^, and **6**^2+^ with non-substituted oligo(9,10-anthrylene) backbone(s) (*n* = 1–6) were designed by end-capping with a dibenzotropylium skeleton at each end of the molecules, which is a stable and planar cation unit (Scheme 1).^57,58^ These compounds are expected to be easily handled in solution despite their huge size (molecular formula: C_130_H_100_, molecular weight of **6**^2+^: 1660.78 excepting anions) since aggregation and/or precipitation would be suppressed due to the electrostatic repulsion between charged moieties. These dications would adopt an orthogonally twisted structure between all 14π-aromatic units, which would also increase solubility in common organic solvents. A change of counter anions would enable easy modification of the solubility of dicationic salts. Thus, we envisaged that the electronic properties of non-substituted oligo(9,10-anthrylene)s could be readily clarified by spectroscopic measures in solution. Although the cationic moieties at both ends act as electron-withdrawing groups, their effects on the electronic structure of the oligo(9,10-anthrylene)s would be minimized (e.g., only Coulombic effects) due to the orthogonal geometry. Thus, the relationship between the electron-donating properties of oligo(9,10-anthrylene)s and the number of anthrylene units could be elucidated in detail due to the number-dependent localization of the HOMO on certain anthrylene units. Another concern is the conversion of oligo(9,10-anthrylene)s into oligo(9,10-anthraquinodimethane)s. Thus, upon reduction, due to the unique rigidity of the seven-membered carbon ring, a dibenzotropylium moiety would be transformed into a planar dibenzocycloheptatrienyl radical or a folded dibenzoheptafulvene structure. Accordingly, the neutral species generated upon two-electron (2e) reduction of **1**^2+^–**6**^2+^ would be an anthrylene-based bis(dibenzocycloheptatrienyl) biradical as an open-shell species while maintaining the orthogonally twisted structure as in the original dications. However, they would, if possibly, be isomerized into oligo(anthraquinodimethane)-based bis(dibenzoheptafulvene) as closed-shell species, in which all of the anthracene units adopt a folded structure. By tuning the number of anthrylene unit(s) between two dibenzotropyliums, the preference of one isomeric structure over the other could be controlled with a drastic change in their properties.

Herein, we reveal that an oligo(9,10-anthrylene) scaffold with dibenzotropyliums is one of the best strategies for examining the potential functions and tunability of structures and properties based on the orthogonally twisted structure with multiple π-conjugated carbon backbones. In particular, this “cation-capping approach with an orthogonally twisted geometry” in linearly connected oligomers would enable the observation of potentially unstable electronic states such as multi-cationic and/or open-shell states that are short-lived without adopting this strategy. Therefore, this approach, which allows us to isolate a family of compounds with a non-substituted oligo(9,10-anthrylene) and to elucidate their structures and properties, represents an important tool for the future design and development of unique molecules with an extended π-conjugated backbone.

## Results and Discussion

### Preparation of Dications **1**^2+^(BF_4_^–^)_2_–**6**^2+^(BF_4_^–^)_2_

**Scheme 2:**
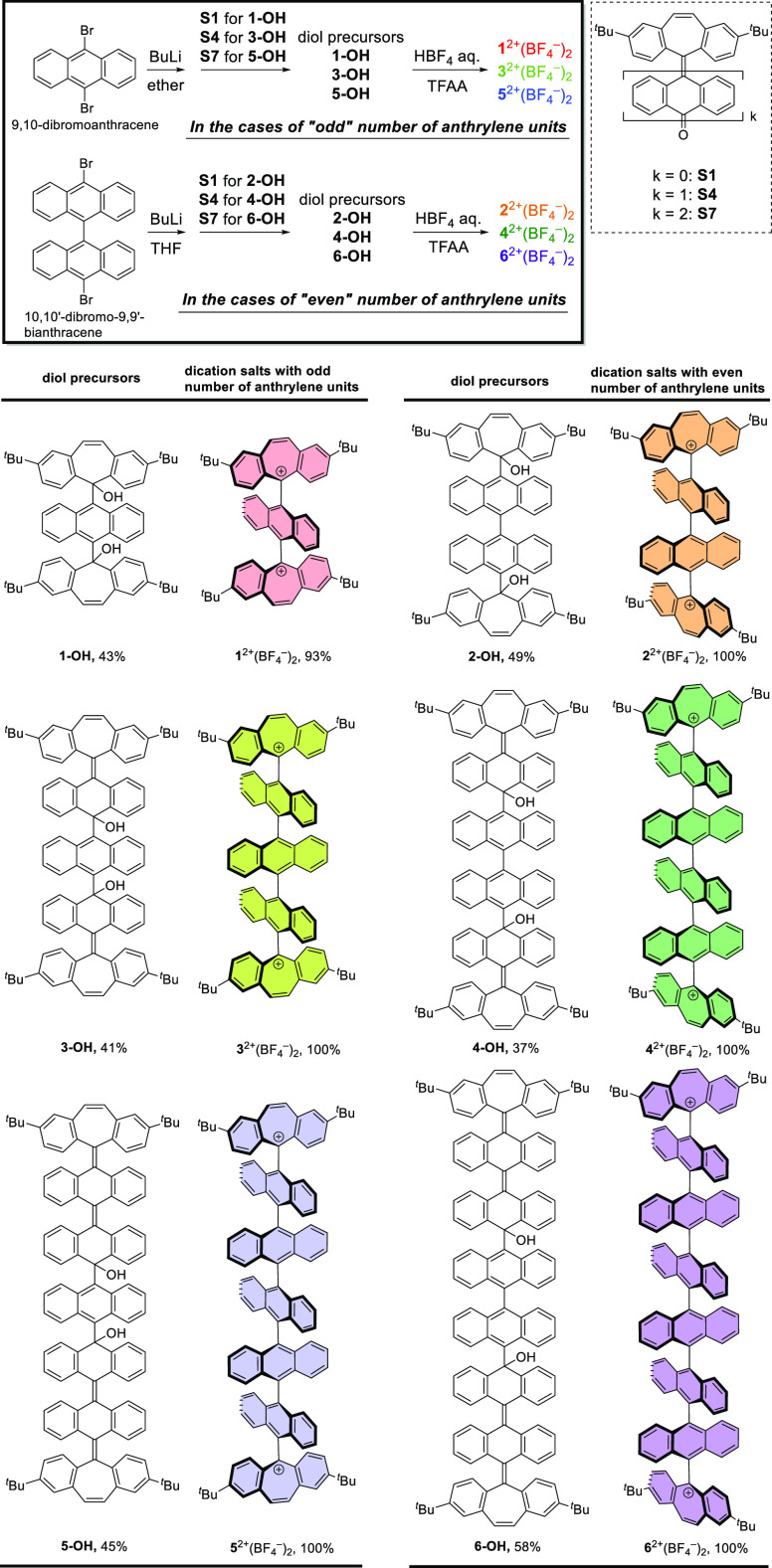
Preparation of Dications **1**^2+^(BF_4_^–^)_2_–**6**^2+^(BF_4_^–^)_2_

As shown in Scheme 2, six kinds of dication salts with odd and even numbers of anthrylene units were synthesized from 9,10-dibromoanthracene and 10,10′-dibromo-9,9′-bianthracene, respectively. Diol precursors **1-OH**, **2-OH**, **3-OH**, **4-OH**, **5-OH**, and **6-OH** were prepared by dilithiation of the dibromides followed by addition of the corresponding ketones **S1**, **S4**, and **S7** (Scheme S1 and Figure S37) for **1-OH** and **2-OH**, **3-OH** and **4-OH**, and **5-OH** and **6-OH**, respectively. In contrast to **1-OH** and **2-OH** synthesized as single diastereomers, **3-OH**, **4-OH**, **5-OH**, and **6-OH** were obtained as mixtures of diastereomers, each of which was identified to be one of the diastereomers by FD-MS. Upon treatment of these diols with tetrafluoroboric acid in the presence of trifluoroacetic anhydride (TFAA), BF_4_^–^ salts of the desired dications were cleanly isolated as red powders in high yield for all derivatives, even when mixtures of multiple diastereomers were used as in the cases of **3-OH**–**6-OH**. These dication salts **1^2+^**(BF_4_^–^)_2_–**6^2+^**(BF_4_^–^)_2_ were fully characterized by ^1^H NMR and ^13^C NMR spectroscopy, FD-MS or ESI-MS, and single-crystal X-ray structure analyses (*vide infra*). They are stable enough to be easily handled under air at ambient temperature in both the solid state and solution. This is the first example of the synthesis of a family of compounds with one to six non-substituted oligoanthrylene units that could be isolated as stable entities.

### Single-Crystal X-ray Structure Analyses of Dications

**Figure 1:**
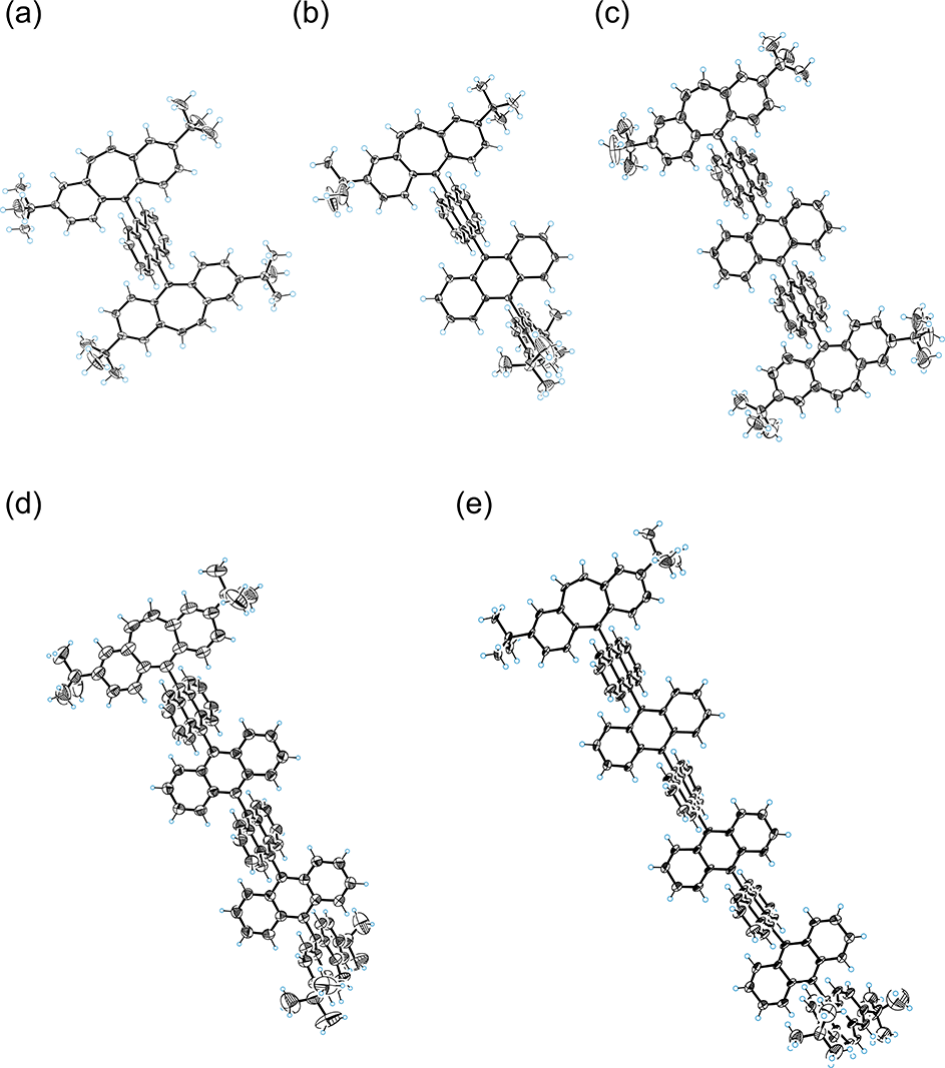
ORTEP drawings of (a) **1**^2+^(BF_4_^–^)_2_, (b) **2**^2+^(PF_6_^–^)_2_, (c) **3**^2+^(BF_4_^–^)_2_, (d) **4**^2+^(BF_4_^–^)_2_, and (e) **6**^2+^(NTf_2_^–^)_2_. The counterions and solvent molecules are omitted for clarity. Thermal ellipsoids are shown at the 50% probability level for (a), (b), (c), and (e) and the 30% probability level for (d).

The structures of dications **1**^2+^, **2**^2+^, **3**^2+^, **4**^2+^, and **6**^2+^ were successfully determined by single-crystal X-ray structure analyses, for which single crystals of **1**^2+^, **3**^2+^, and **4**^2+^ were obtained as BF_4_^–^ salts. Dications **2**^2+^ and **6**^2+^ were prepared by using PF_6_^–^ and bis(trifluoromethanesulfonyl)imide (NTf_2_^–^) as counter anions, respectively, to achieve better crystallinity (Figure 1 and Table S3). Previous studies were limited only to determining the X-ray structures of bianthracene (*m* = 2)^31−33,35−39^ and teranthracene derivatives (*m* = 3),^43,50−52,55^ the latter of which have multiple substituents on the anthrylene units. In contrast, the molecular design in this study actually allowed us to determine the X-ray structures of the longer oligoanthrylene series of **3**^2+^, **4**^2+^, and **6**^2+^ with an orthogonally twisted geometry of five to eight 14π-electron units connected in a linear manner, where the number of anthrylene units (three to six) is significantly greater than that previously reported. These results demonstrated that our “cation-capping approach” offers a significant benefit for obtaining the structure of a huge carbon skeleton such as oligoanthrylene, which would serve as a valuable strategy for studying novel π-conjugated carbon frameworks.

Based on the results of X-ray analyses, the dihedral angles between adjacent 14π-electron units were determined based on the mean planes defined by the 14 (anthrylene) or 15 (dibenzotropylium) carbon atoms that compose each aromatic unit (Table S2). We confirmed that all dications adopt an almost orthogonally twisted structure for each pair of 14π-aromatic units in the crystal of their salts, which is in good agreement with the optimized structures obtained by density functional theory (DFT) calculations at the CAM-B3LYP/6-31G(d) level, which gave all the dihedral angles of 90.0° for all of the dications (Figure S25). The maximum deviation of the dihedral angles from the calculated value is only 11.77(3)° in **2**^2+^, indicating that the orthogonality between neighboring units is highly retained. Thus, the molecules of **1**^2+^, **2**^2+^, **3**^2+^, **4**^2+^, and **6**^2+^ are less perturbed by the crystal packing force. This is because no obvious intermolecular interactions between anthrylene units are observed in the crystals of these dication salts due to the orthogonally twisted structures (closest distance between carbon atoms: >3.4 Å). Furthermore, the bulky ^*t*^Bu groups effectively suppress intermolecular π–π stacking and C–H···π contacts between the dibenzotropylium units at both ends. The scarcity of intermolecular interactions is the key for a high enough solubility of these dications to perform various measurements for a series of oligoanthrylenes composed of rigid and planar anthrylene units without any substituents.

### UV–vis–NIR Absorption Properties of Dications

**Figure 2:**
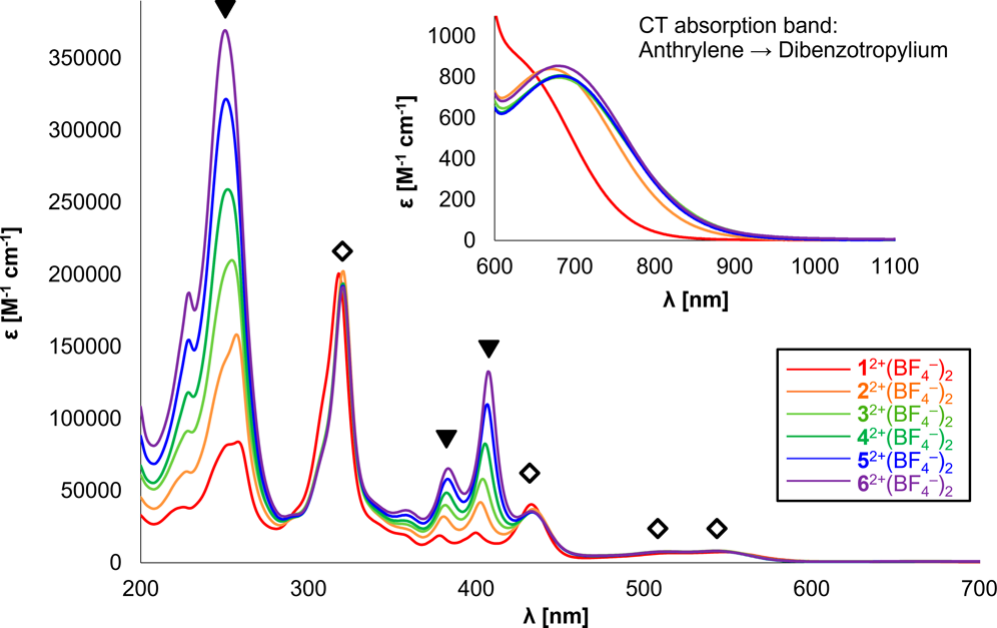
UV–vis–NIR spectra of dications **1**^2+^(BF_4_^–^)_2_, **2**^2+^(BF_4_^–^)_2_, **3**^2+^(BF_4_^–^)_2_, **4**^2+^(BF_4_^–^)_2_, **5**^2+^(BF_4_^–^)_2_, and **6**^2+^(BF_4_^–^)_2_ in CH_3_CN. Triangle- and diamond-marked absorption peaks were mainly assigned to the absorptions of the anthrylene units and the dibenzotropylium skeleton, respectively.

Since anthrylene unit(s) and the terminal dibenzotropylium units are almost orthogonally connected to each other in the crystals of **1**^2+^–**6**^2+^, each anthrylene unit can be considered to be electronically independent due to a negligible overlap of their p orbitals between neighboring units. To gain insights into whether or not the oligo(9,10-anthrylene)-based dications would maintain this orthogonally twisted geometry even in solution, we investigated the electronic properties of **1**^2+^–**6**^2+^ in solution. First, UV–vis–NIR absorption spectra of BF_4_^–^ salts of dications **1**^2+^–**6**^2+^ were measured in CH_3_CN (Figure 2 and Table S4). Characteristic absorption bands in the UV region show a nearly equally spaced increase in molar absorption coefficient values with an increase in the number of anthrylene units, where a vibrational structure assigned to absorptions of the anthrylene skeleton was clearly observed (λ_max_ = 250–258, 378–383, and 400–407 nm in CH_3_CN). The absorption bands in the UV–vis region that show hardly any change in molar absorption coefficients among the derivatives with different numbers of anthrylene units can be assigned to the absorptions of the dibenzotropylium skeleton (peak wavelength: 320–321, 433–434, 514–519, and 543–546 nm in CH_3_CN) because all of the dications have the same two cationic chromophores. Since no change in the peak wavelengths of the main absorption bands was observed among dications **1**^2+^(BF_4_^–^)_2_–**6**^2+^(BF_4_^–^)_2_, there is no significant electronic interaction between anthrylene units even in solution.

On the other hand, quite weak absorption bands attributed to forbidden charge-transfer (CT) transitions from anthrylene unit(s) to dibenzotropyliums were observed in the NIR region. Time-dependent (TD) DFT calculations were conducted on all dications **1**^2+^–**6**^2+^ at the CAM-B3LYP/6-31G(d) level, which can predict the origin of the lowest-energy electronic transitions (Supporting Information pp. 49–54 and Figures S26–S28). According to TD-DFT calculations, these CT absorption bands are assigned to be electronic transitions from the anthrylene unit, which is placed next to the dibenzotropylium unit, to the dibenzotropylium. The molar absorption coefficients of these CT bands for **1**^2+^–**6**^2+^ were considerably smaller than those observed in other anthrylene-based derivatives with more flexible diarylmethylium moieties,^59^ indicating that the orthogonally twisted structures in **1**^2+^–**6**^2+^ are robust to suppress the interaction between anthrylene unit(s) and terminal dibenzotropyliums.

These results showed that the orthogonally twisted structures of all 14π-conjugated units are highly preserved in solution due to the oligo(9,10-anthrylene) scaffold in combination with the end-capping with two rigid dibenzotropyliums, and thus the electronic interaction between 14π-aromatic units is weak for all dications **1**^2+^–**6**^2+^, as designed.

### Oxidation Behavior of Dications and Formation of Multivalent Cations

**Figure 3:**
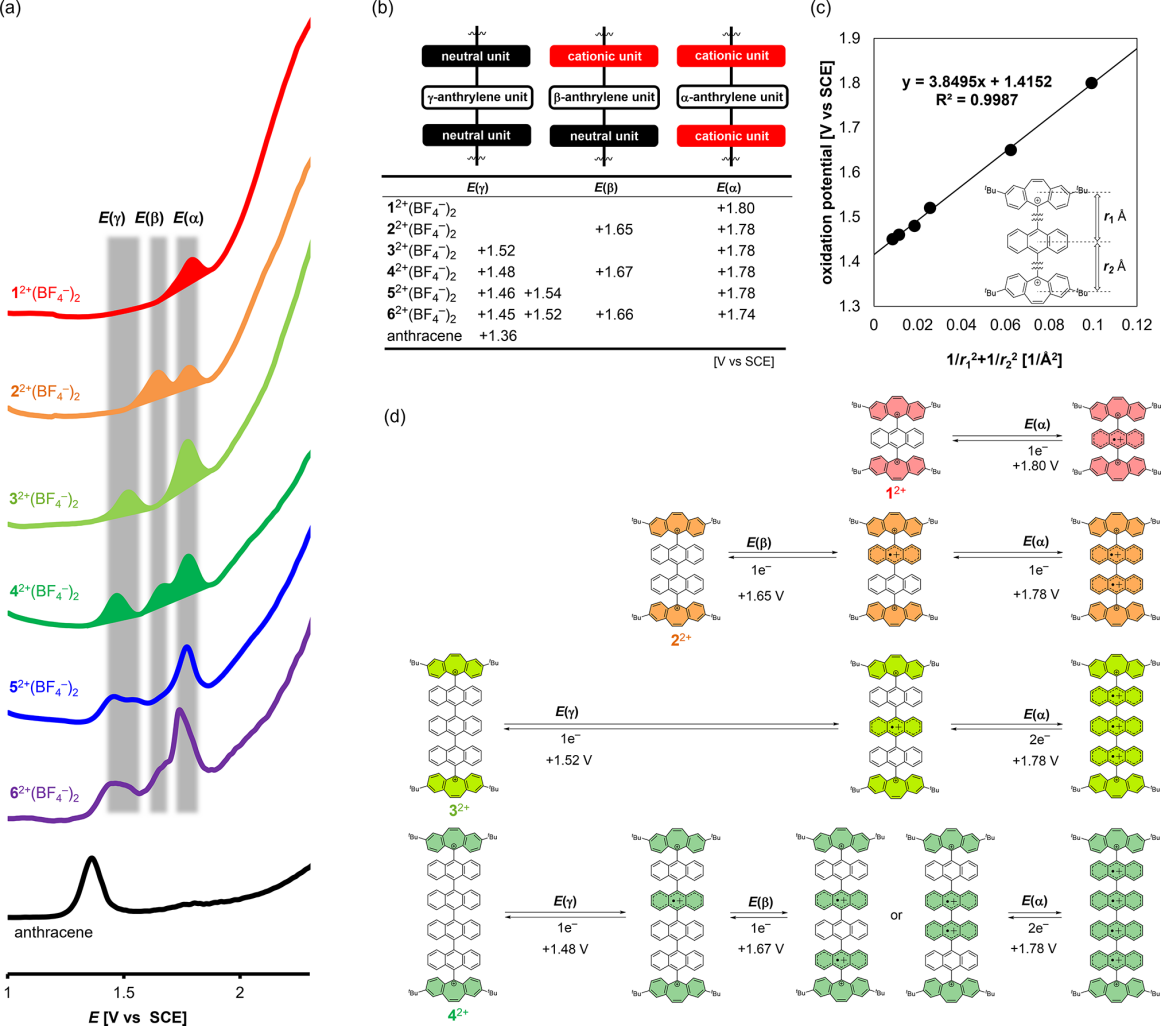
(a) Differential pulse voltammograms of dications **1**^2+^(BF_4_^–^)_2_, **2**^2+^(BF_4_^–^)_2_, **3**^2+^(BF_4_^–^)_2_, **4**^2+^(BF_4_^–^)_2_, **5**^2+^(BF_4_^–^)_2_, **6**^2+^(BF_4_^–^)_2_, and anthracene in 0.2 mM CH_2_Cl_2_ solution containing 0.1 M Bu_4_NBF_4_ as a supporting electrolyte (Pt electrode). (b) All oxidation potentials of dications assignable to the release of electrons from the α-anthrylene unit, β-anthrylene unit, or γ-anthrylene unit. (c) Plot of the oxidation potential (corresponding to first oxidation waves) for dications **1**^2+^(BF_4_^–^)_2_–**6**^2+^(BF_4_^–^)_2_ in CH_2_Cl_2_ against the sum of 1/*r*^2^ where *r* is the distance between the center of gravity of the dibenzotropylium unit and the central anthrylene unit(s) in the optimized structures. (d) Schematic diagram of the stepwise oxidation process of dications **1**^2+^(BF_4_^–^)_2_–**4**^2+^(BF_4_^–^)_2_. Cationic units were colored in the scheme.

Next, we conducted voltammetric analyses of the BF_4_^–^ salts of dications by differential pulse voltammetry (DPV) in CH_2_Cl_2_ (Figure 3a,b) to elucidate the electron-donating properties of oligo(9,10-anthrylene)s end-capped with two dibenzotropyliums (Figures S39 and S40). This should give insight into the oxidative properties of the oligo(9,10-anthrylene)s. These analyses revealed that each dication underwent stepwise oxidation in a reversible manner, where the maximum number of electron(s) released was in accordance with the number of orthogonally connected anthrylene units, resulting in the formation of multivalent cations.

The first oxidation wave, measured as a reversible process for all derivatives, shifted to a less positive potential with an increase in the number of anthrylene units [+1.80 V vs SCE for **1**^2+^(BF_4_^–^)_2_, +1.65 V for **2**^2+^(BF_4_^–^)_2_, +1.52 V for **3**^2+^(BF_4_^–^)_2_, +1.48 V for **4**^2+^(BF_4_^–^)_2_, +1.46 V for **5**^2+^(BF_4_^–^)_2_, and + 1.45 V for **6**^2+^(BF_4_^–^)_2_], suggesting an increase in the HOMO level of dications **1**^2+^–**6**^2+^ in the order of the number of anthrylene units. To clarify this point, the distributions of HOMO for the dications were estimated by DFT calculations at the CAM-B3LYP/6-31G(d) level (Figures S26–S28). For **1**^2+^, **3**^2+^, and **5**^2+^, with an odd number of anthrylene unit(s), the HOMO was located on the anthrylene unit at the very center, which is the furthest away from the two terminal dibenzotropylium units. For **2**^2+^, **4**^2+^, and **6**^2+^, with an even number of anthrylene units, the HOMO and HOMO-1 are degenerated, and both orbitals are distributed on the two central anthrylene units, which are apart from the dibenzotropylium units at both termini. Therefore, the first oxidation wave in the voltammogram can be accounted for by the release of an electron from the anthrylene unit(s) located in the center of the molecule. A linear correlation (*R*^2^ = 0.9987) was observed when the values of the oxidation potential for these dications were plotted against the sum of 1/*r*^2^ where *r* is the distance between the center of gravity of the dibenzotropylium unit and the central anthrylene unit(s) in the optimized structures. As shown in Figure 3c, the value of the intercept (+1.42) indicates the oxidation potential for a compound with an infinite number of anthrylene units. This intercept is very close to the experimentally measured oxidation potential of the parent anthracene (+1.36 V), with a difference of only 0.06 V, demonstrating that the change in HOMO levels for anthrylene-based dications **1**^2+^–**6**^2+^ follows Coulomb’s law.^60,61^ A similar behavior was observed when the voltammetric analyses were conducted in more polar solvents such as CH_3_CN or 1,1,1,3,3,3-hexafluoro-2-propanol (HFIP) (Figures S39 and S40). The differences between the value of the intercept and the oxidation potential of the parent anthracene are only 0.05 and 0.02 V, respectively, in CH_3_CN and HFIP (Figure S41). These results show that neither significant overlap of orbitals nor delocalization of electrons occurs between the neighboring anthrylene units, which is in accord with the results of the UV–vis–NIR absorption measurements (*vide supra*).

Furthermore, if the orthogonally twisted structures are preserved even after the one-electron oxidation of dications, Coulombic considerations can also be applied to further oxidation processes during the formation of higher multivalent cations. Focusing on the relationship between the oxidation potentials and the anthrylene units involved in the next oxidation, the anthrylene unit undergoing the next oxidation can be classified into three types by considering the charge state of the adjacent tricyclic units: (α) anthrylene between two cationic units, (β) anthrylene between cationic and neutral units, and (γ) anthrylene between two neutral units (Figure 3b). Based on the Coulombic effects for the three anthrylene units of (α), (β), or (γ) from the adjacent units, the oxidation potential should be more positive in the order *E*(α) > *E*(β) > *E*(γ). In fact, in terms of the observed values of the first oxidation wave for **1**^2+^(BF_4_^–^)_2_–**6**^2+^(BF_4_^–^)_2_, the first oxidation process of **1**^2+^(BF_4_^–^)_2_ [*E*(α) = +1.80 V], **2**^2+^(BF_4_^–^)_2_ [*E*(β) = +1.65 V], and **3**^2+^(BF_4_^–^)_2_ [*E*(γ) = +1.52 V] can be explained in terms of the oxidation of α-, β-, and γ-anthrylene units, respectively (Figure 3d). The fact that the first oxidation of **3**^2+^(BF_4_^–^)_2_–**6**^2+^(BF_4_^–^)_2_ occurs at almost the same potential region (+1.52 to +1.45 V) due to the oxidation of γ-anthrylene units also indicates that the above three classifications are reasonable.

In this way, the oxidation potentials after the first oxidation wave can be estimated just by considering the position of an anthrylene unit that would be involved in the next oxidation while considering that an α-, β-, or γ-anthrylene unit is oxidized at its unique potential region [*E*(α) = +1.74 to +1.80 V, *E*(β) = +1.65 to +1.67 V, and *E*(γ) = +1.45 to +1.52 V] (Figure 3d). In fact, the second oxidation wave of **2**^2+^(BF_4_^–^)_2_ and **3**^2+^(BF_4_^–^)_2_ appeared at almost the same potential because the second wave in each corresponds to the oxidation of the α-anthrylene unit, while that of **4**^2+^(BF_4_^–^)_2_ appeared at a less positive region corresponding to oxidation of the β-anthrylene unit. The third oxidation wave of **4**^2+^(BF_4_^–^)_2_ was observed at a potential similar to the second one of **2**^2+^(BF_4_^–^)_2_ and **3**^2+^(BF_4_^–^)_2_ due to involvement of an α-anthrylene unit in these oxidation processes. This explanation allows us to understand the subsequent oxidative behavior after the first oxidation of longer dications **5**^2+^(BF_4_^–^)_2_ and **6**^2+^(BF_4_^–^)_2_. These results indicated that the orthogonally twisted structure is preserved in multivalent cations as well as in dications. We have found a previously unreported simple and straightforward rule for orthogonally connected oligo(9,10-anthrylene)s, which is based solely on Coulombic considerations.

In terms of the number of electrons released upon oxidation with the formation of multivalent cations, the second oxidation wave of **3**^2+^(BF_4_^–^)_2_ and the third oxidation wave of **4**^2+^(BF_4_^–^)_2_ exhibited a larger peak area than others (Figure 3a). Thus, these oxidation processes should correspond to a one-wave 2e-oxidation. Due to the similar one-wave multi-electron-oxidation process, some oxidation peaks get broad in **5**^2+^(BF_4_^–^)_2_ and **6**^2+^(BF_4_^–^)_2_. Still, it is highly likely that **5**^2+^and **6**^2+^ are oxidized up to **5**^7+^ and **6**^8+^ based on the systematic voltammetric analyses of **1**^2+^–**6**^2+^. Therefore, all anthrylene units were oxidized in CH_2_Cl_2_ in all of the derivatives with an increase in the oxidation number by *n* at most. In addition, almost the same behavior was observed in polar CH_3_CN (Figure S39c). Furthermore, voltammetric analyses revealed that all of the oxidation processes are reversible. No further oxidation wave was observed while the potential was swept up to 2.5 V beyond the *E*(α) region.

Particularly noteworthy is the finding that the reversible formation of multivalent cations can be observed by end-capping with dibenzotropylium units because many papers to date have reported that condensation reactions easily proceeded on anthrylene skeletons upon oxidation of bianthracenes or higher analogues.^38,43,51−56,62,63^ This work is the first to demonstrate the relationship between the number of π units and their redox behaviors for a series of linearly connected π-compounds. This study demonstrated that multivalent cations can be effectively stabilized by the use of orthogonally twisted structures between tricyclic units under a “cation-capping approach”.

### Reduction Behavior of Dications and Formation of Two Types of Neutral Species

We next investigated the reduction behavior of dications **1**^2+^–**6**^2+^ to gain insight into the structure of 2e-reduced species. Based on the unique rigidity of the seven-membered carbon ring, the dibenzotropylium moiety can be reduced to give a planar dibenzocycloheptatrienyl radical or a butterfly-shaped dibenzoheptafulvene structure as an open-shell or closed-shell species, respectively. Accordingly, 2e-reduction of **1**^2+^–**6**^2+^ with two dibenzotropylium moieties would produce an open-shell twisted (**T**) or closed-shell folded (**F**) form with anthrylene(s) or anthraquinodimethane(s) in the center of the molecules. To estimate which form is the most thermodynamically stable structure as a reduced product from **1**^2+^ to **6**^2+^, we performed DFT calculations at the (U)B3LYP/6-31G(d) level (Figures S29 and S30). Although there are many isomers for the **F** form by adopting either *anti*- or *syn*-type configurations, since closed-shell **1**–**6** have two or more overcrowded alkene units, only the most favorable all-*anti*-type folded forms among the configurational isomers were chosen to be calculated. As can be seen from the relative energies of the **F** and **T** forms, the **F** form was predicted to be more stable than the **T** form for **1** and **2** with one and two anthrylene units, respectively, while the **T** form was estimated to be more stable than the **F** form for **3**–**6** with three to six anthrylene units (Table S1). For **2** and **3**, both forms would be observed by considering the small energy difference, and thus we sought to clarify the structures of the corresponding reduced species.

**Figure 4:**
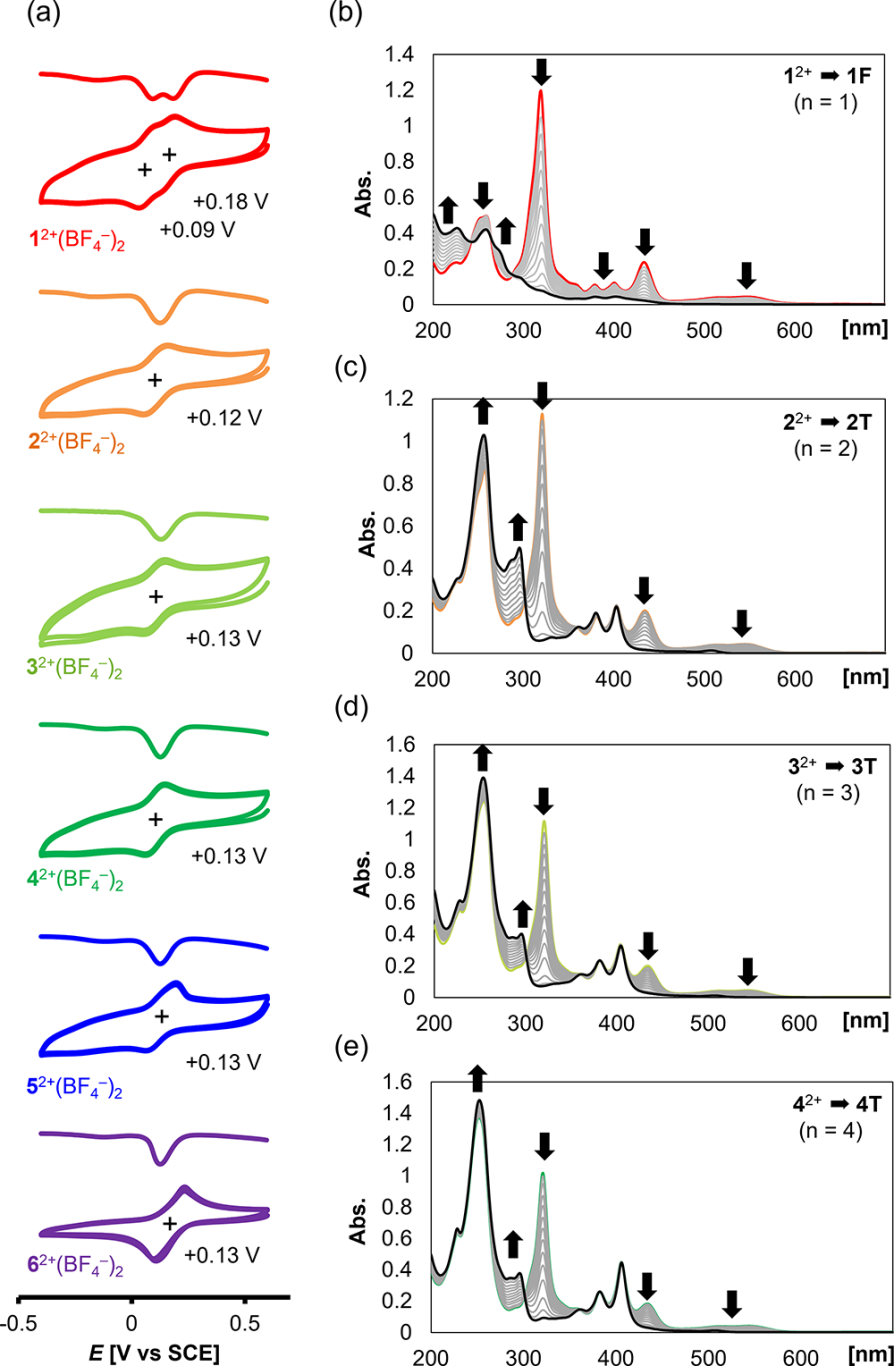
(a) Cyclic voltammograms (scan rate 100 mVs^–1^) and differential pulse voltammograms of 0.2 mM solution of dications **1**^2+^(BF_4_^–^)_2_, **2**^2+^(BF_4_^–^)_2_, **3**^2+^(BF_4_^–^)_2_, **4**^2+^(BF_4_^–^)_2_, **5**^2+^(BF_4_^–^)_2_, and **6**^2+^(BF_4_^–^)_2_ in CH_2_Cl_2_. (b–e) Changes in UV–vis spectra upon electrochemical reduction (20 μA) of (b) **1**^2+^(BF_4_^–^)_2_ (5.96 μM), (c) **2**^2+^(BF_4_^–^)_2_ (5.59 μM), (d) **3**^2+^(BF_4_^–^)_2_ (5.81 μM), and (e) **4**^2+^(BF_4_^–^)_2_ (5.28 μM) in CH_3_CN containing 0.05 M Et_4_NClO_4_ as a supporting electrolyte (every 30 s).

The reduction behavior of the dications was first investigated by voltammetric analyses in CH_2_Cl_2_ (Figure 4a). Reversible 2e-reduction waves were observed in the range +0.09 to +0.18 V (vs SCE) for all BF_4_^–^ salts of dications. These results indicate that the perpendicular geometries of not only **3**^2+^–**6**^2+^ but also **1**^2+^ and **2**^2+^ do not change under the measurement conditions. Thus, **1T** and **2T** with a twisted geometry similar to the structure of dications should be kinetically produced with a longer lifetime than a few seconds at least.

**Scheme 3:**
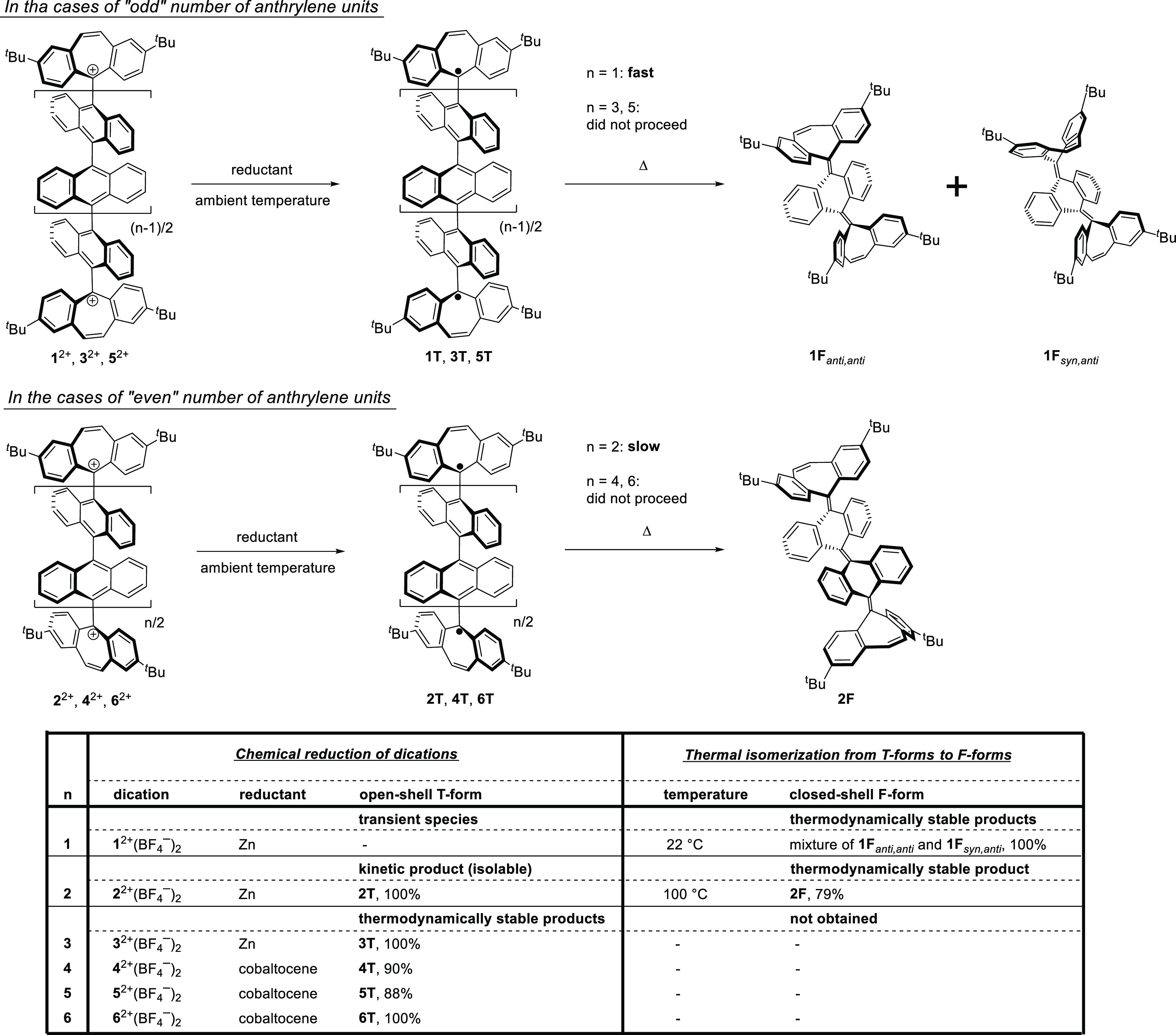
Two-Electron Reduction of Dications to Give Neutral **T** Forms and Thermal Isomerization from **T** Forms to **F** Forms

To clarify the properties and identities of 2e-reduced species, a series of electrochemical reductions were conducted for **3**^2+^(BF_4_^–^)_2_, **4**^2+^(BF_4_^–^)_2_, **5**^2+^(BF_4_^–^)_2_, and **6**^2+^(BF_4_^–^)_2_ in CH_3_CN, and the results were monitored by UV–vis spectroscopy (Figure 4d,e and Figure S45). According to the calculation, the 2e-reduced state for each compound prefers to adopt the **T** form rather than the **F** form. For **3**^2+^(BF_4_^–^)_2_ and **4**^2+^(BF_4_^–^)_2_, only the absorptions that originated from the dibenzotropylium skeletons disappeared upon electrochemical reduction and clean conversion was observed with isosbestic points (Figure 4d,e). In addition, the vibrational structure in the region of 350–410 nm assigned to the electronic transition of the anthrylene skeletons exhibited almost no change. These results indicated that the structures of the oligo(anthrylene)s in the molecules were maintained upon 2e-reduction, meaning that open-shell **3T** and **4T** with orthogonally connected anthrylene units were certainly produced. For **5**^2+^(BF_4_^–^)_2_ and **6**^2+^(BF_4_^–^)_2_, a similar behavior was observed upon electrochemical reduction, even though there was a sign that a reduced species was deposited on an electrode surface (Figure S45). Upon chemical reduction of **3**^2+^(BF_4_^–^)_2_ with zinc powder and **4**^2+^(BF_4_^–^)_2_, **5**^2+^(BF_4_^–^)_2_, and **6**^2+^(BF_4_^–^)_2_ with cobaltocene in preparative-scale experiments, the resulting species were completely NMR-silent. In fact, ESR measurements of the solids showed signals characteristic of the presence of a dibenzocycloheptatrienyl radical (Scheme 3 and Figure S44b–e). According to DFT calculations at the UB3LYP/6-31G(d) level, the energy difference between the singlet and triplet biradicals was found to be zero for all derivatives (Table S1), which is in good agreement with previously reported orthogonally twisted biradicals.^64^ Formation of the neutral species was also confirmed by IR spectroscopy, which showed the disappearance of absorptions of BF_4_^–^ ions in the reduction products (Figure S50c–f). The UV–vis spectra of the isolated solids by chemical reduction are almost identical to those obtained by electrochemical reduction (Figures S48b–d and S49b–e). In particular, the intensity ratio of the strong peak around 250 nm and the absorption showing the vibrational structure of anthrylenes around 400 nm are almost the same, showing that electrolytic and chemical reduction of dications gave the same species. These results demonstrated that the biradical species **3T**, **4T**, **5T**, and **6T**, all of which were predicted to be the most stable configurational isomers by theoretical studies, were cleanly obtained upon 2e-reduction of dications. Notably, these open-shell **T** forms are stable enough to be easily manipulated under air at ambient temperature. Furthermore, there was no change in the UV–vis absorption spectrum of **3T** upon heating at 100 °C for 10 min in a toluene solution, indicating that **3T** has high thermal stability despite being an open-shell species composed of pure hydrocarbons (Figure S51f).

**Figure 5:**
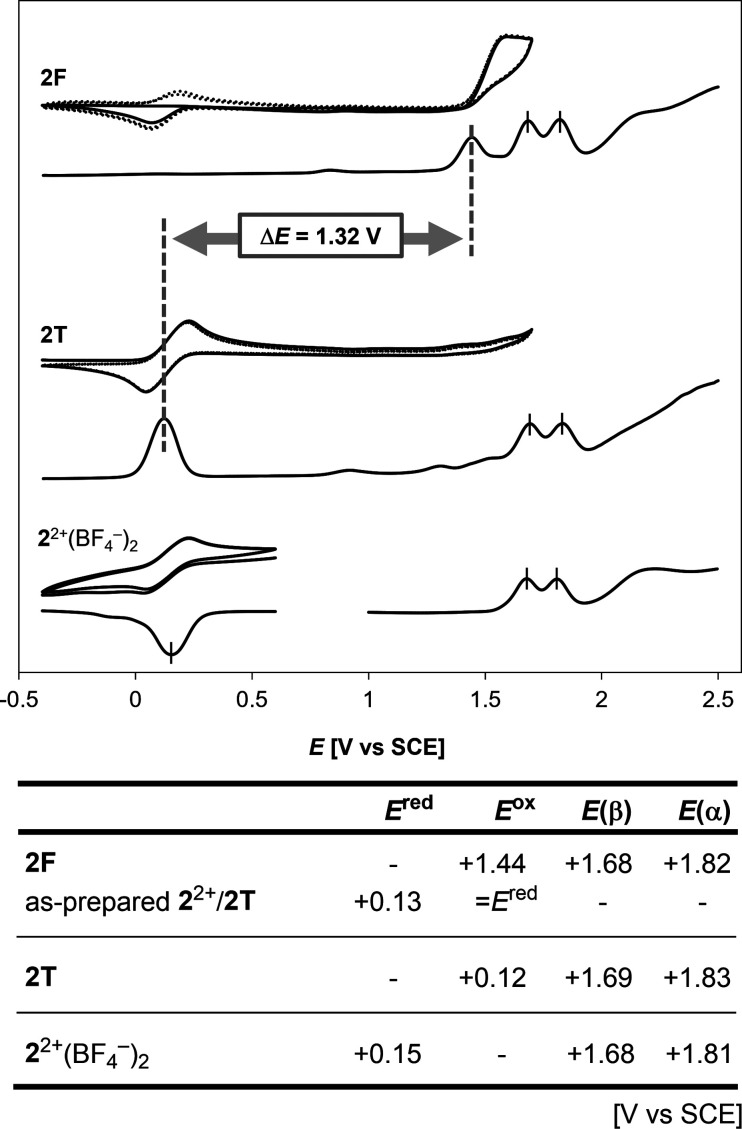
Cyclic voltammograms (scan rate 500 mVs^–1^) and differential pulse voltammograms of 1.0 mM solution of **2F**, **2T**, and **2**^2+^(BF_4_^–^)_2_ in CH_2_Cl_2_ containing 0.1 M Bu_4_NBF_4_ as a supporting electrolyte (Pt electrode). Backgrounds were subtracted for all cyclic voltammograms. The second and third cycles are shown by dotted lines in cyclic voltammograms of **2F** and **2T**.

When electrochemical reduction was conducted for **2**^2+^(BF_4_^–^)_2_, behavior similar to those for **3**^2+^(BF_4_^–^)_2_–**6**^2+^(BF_4_^–^)_2_ was observed, suggesting that open-shell **2T** was generated as a kinetically stable isomer even though the **F** form was predicted to be more stable than the **T** form for the neutral state of **2** (Figure 4c). Accordingly, reduction of **2**^2+^(BF_4_^–^)_2_ with zinc powder quantitatively gave **2T** as a deep-green solid, indicating the formation of biradical species (Scheme 3 and Figures S44a, S48a, S49a, and S50b). In contrast to the previous report on the similar biradical,^37^ we found that biradical **2T** was converted to closed-shell species **2F** upon heating at 100 °C for 20 min in toluene, and **2F** was isolated in 79% yield (Scheme 3). The closed-shell **2F** adopts an all-*anti* configuration, as determined by single-crystal X-ray structure analysis (Figure S38). To gain further insight into the thermal isomerization process, isomerization from **2T** to **2F** was monitored by UV–vis spectroscopy (Figure S51a–e). The absorption band assignable to the electronic transitions of anthrylenes for **2T** rapidly decayed upon heating at 100 °C, and the spectral pattern changed to that of isolated **2F**. By supposing first-order reaction kinetics, the rate constant *k* of isomerization was determined to be 6.08 × 10^–4^, 1.34 × 10^–3^, 4.02 × 10^–3^, and 8.67 × 10^–3^ s^–1^ at 70, 80, 90, and 100 °C, respectively, based on the molar absorption coefficient at 297 nm showing the largest change (Figure S52). According to an Arrhenius plot, the activation energy for thermal isomerization was estimated to be 23.1 kcal mol^–1^, suggesting that **2T** has a long half-life at ambient temperature (20 °C, 100 h). The persistence and kinetic stability of **2T** were also confirmed by measuring the cyclic voltammogram of the isolated solid of **2T**, which showed a reversible oxidation wave, which is similar to the oxidation process of twisted biradical species, as in **3T**–**6T** (Figure 5 and Figure S43). There have been several reports for bianthracene derivatives exhibiting a change in conformation from the **T** form to the **F** form; however, it is difficult to isolate the metastable open-shell **T** form due to its very short lifetime at ambient temperature under air. In contrast, we revealed that all three states, the dication **2**^2+^, the open-shell biradical **2T**, and the closed-shell folded form **2F**, can be isolated as stable entities and mutually interconvert even under ambient conditions. These observations rely on the moderate rigidity of the seven-membered fused-ring structure and the orthogonally twisted structure of the oligoanthrylenes, which play important roles in both raising the activation energy for the change in configuration and kinetically stabilizing carbocations/radicals, which are typically considered to be unstable and reactive.

Upon electrochemical reduction of **1**^2+^(BF_4_^–^)_2_, a continuous decrease not only in the absorptions assigned to the electronic transitions of dibenzotropyliums but also in the vibrational structure of the anthrylene skeleton was observed, which reflected the rapid formation of a closed-shell **F** form, as expected based on the result of DFT calculations (Figure 4b). Although the generation of **1T** with a twisted geometry could be observed in the cyclic voltammogram (Figure 4a), the conversion of **1T** to **1F** is a much faster process than that of **2T** to **2F**. Thus, reduction of **1**^2+^(BF_4_^–^)_2_ with zinc powder did not give **1T** but rather a mixture of two isomers of **F** forms quantitatively, **1F_*anti,anti*_** and **1F_*syn,anti*_**, both of which are thermodynamically more stable than open-shell **1T** (Scheme 3). Upon heating a mixture of two isomers under reflux conditions in DMSO, the *C*_2*v*_-symmetric **1F_*anti,anti*_** was quantitatively obtained as the most stable isomer. Photoirradiation (λ > 360 nm) of two **F** forms quantitatively produces the *C_s_*-symmetric **1F_*syn,anti*_** as a metastable isomer but not **1T**. This behavior is similar to what we previously reported as a molecular switch in response to heat and light to realize selective oxidation (Figure S42).^57^

As mentioned above, the 2e-reduction of dications **1**^2+^–**6**^2+^ exhibited characteristic behavior depending on the number of anthrylene units. For **3**^2+^, **4**^2+^, **5**^2+^, and **6**^2+^, open-shell twisted **3T**, **4T**, **5T**, and **6T** were obtained as the thermodynamically most stable isomers. Despite the absence of bulky substituents such as mesityl groups, the 2e-reductions that generated these open-shell species proceeded almost quantitatively, and all neutral species could be handled as stable entities. In these biradicals, the oligoanthrylene skeleton acts as a rigid spacer, which separates two radical centers with a discrete increase in the distance of separation with an increase in the number of anthrylene units. On the other hand, during the reduction of dications **1**^2+^ and **2**^2+^, the biradical species **1T** and **2T** were generated as kinetic products in voltammetric analyses. Open-shell **1T** was quickly converted to the thermodynamically stable closed-shell **1F_*anti,anti*_** and **1F_*syn,anti*_** even at ambient temperature, whereas **2T** was isolated as a stable entity with an energy of 23.1 kcal mol^–1^ for isomerization. Upon heating of **2T** in a toluene solution, the most stable closed-shell isomer **2F** was obtained, and thus three states can be isolated for bianthrylene-type derivative **2**. In this way, oligoanthrylenes designed under the “cation-capping approach” can lead to new functional materials.

### Switching Behavior between a Dicationic State and a 2e-Reduced State of Oligoanthrylenes

**Scheme 4:**
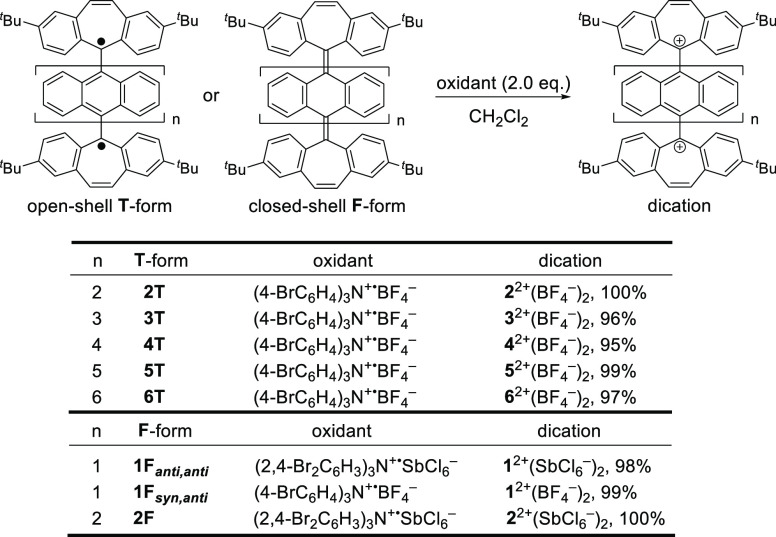
Two-Electron Oxidation of Isolated Neutral Species to Reproduce Original Dications

Due to the persistence of biradical species **2T–6T**, they could be used to construct spectral and magnetic switching systems when reversible interconversion with **2**^2+^–**6**^2+^ is possible. Upon electrochemical oxidation of as-prepared **2T** and **3T** in solution, regeneration of dications **2**^2+^ and **3**^2+^ was confirmed with the appearance of absorption assignable to the formation of dibenzotropiums with isosbestic points (Figure S46a,b). Based on the reversible redox interconversion between dications and biradicals, **2T** and **3T** exhibit clean electrochromism. For the electrochemical oxidation of as-prepared **4T**, **5T**, and **6T** in solution, almost the same spectral change was observed as in **2T** and **3T**, although conversion did not proceed completely probably due to deposition on the electrode surface (Figure S46c–e). In preparative-scale experiments, upon treatment of isolated open-shell **2T**–**6T** with two equivalents of (4-BrC_6_H_4_)_3_N^+•^BF_4_^–^, original dications **2**^2+^(BF_4_^–^)_2_–**6**^2+^(BF_4_^–^)_2_ were obtained quantitatively (Scheme 4). These results reveled that a family of dications **2**^2+^–**6**^2+^ and **2T**–**6T** show high reversibility in terms of redox interconversion and thus are potential candidates for the development of molecular switches with which color and magnetic properties can be controlled by applying an electric potential. In addition, treatment of closed-shell neutral species **1F_*anti,anti*_**/**1F_*syn,anti*_** and **2F** with two equivalents of an appropriate oxidant, (4-BrC_6_H_4_)_3_N^+•^BF_4_^–^ or (2,4-Br_2_C_6_H_3_)_3_N^+•^SbCl_6_^–^, quantitatively gave dications ****1****^2+^(BF_4_^–^)_2_, **1**^2+^(SbCl_6_^–^)_2_ and **2**^2+^(SbCl_6_^–^)_2_, respectively.

**Scheme 5:**
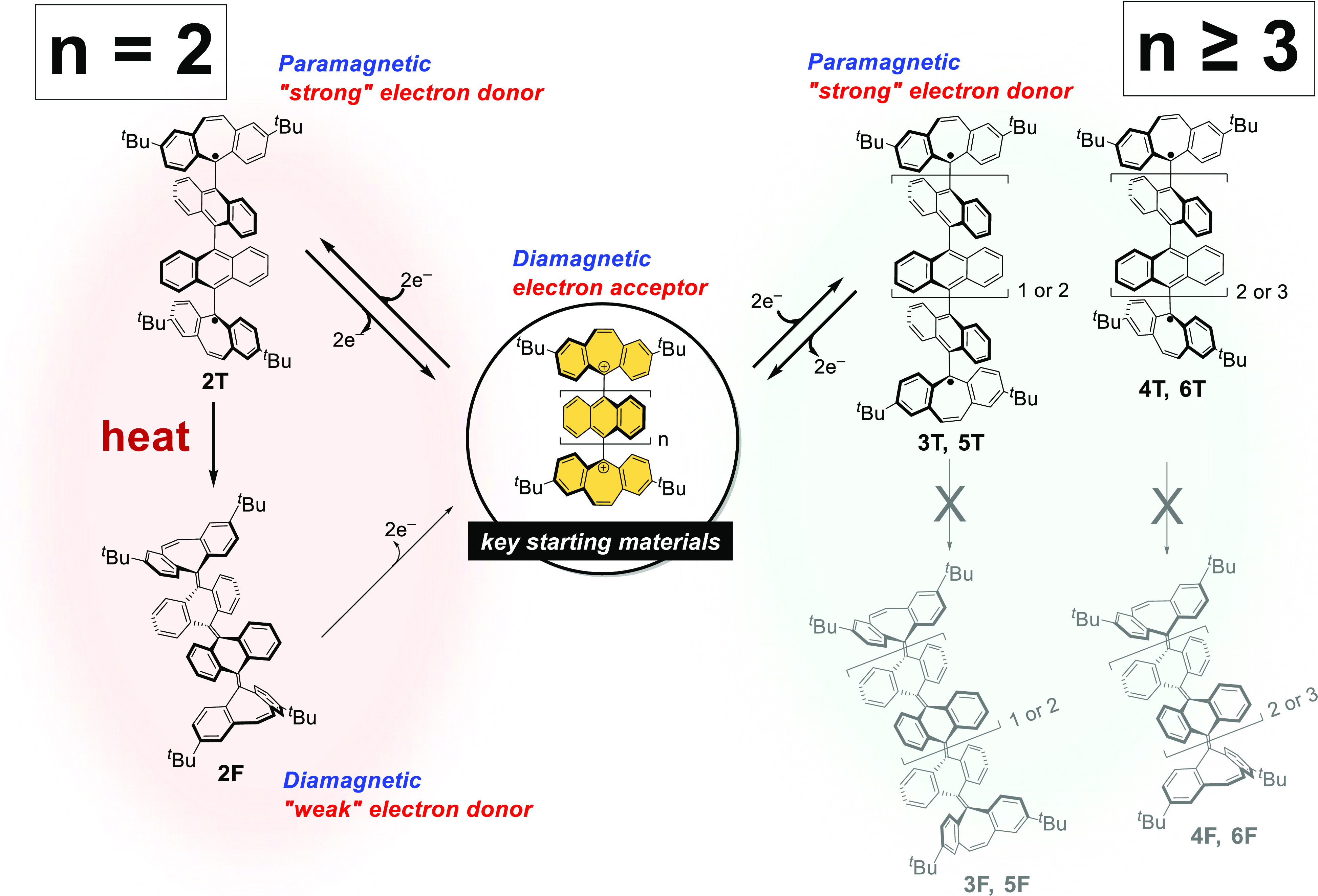
Family of Molecular Switches Composed of 9,10-Anthrylene(s), in which the Switching Behavior Can be Precisely Tuned by Selecting the Number of Anthrylene Unit(s) between Two Dibenzotropyliums In the case of *n* = 1, photo- and thermally controlled redox interconversion with **1**^2+^ occurs without magnetic switching, which is similar to previously reported behavior.

Based on the above results, the dications synthesized in this study can serve as key starting materials for making a series of unique molecular switches (Scheme 5). Dications **2**^2+^, **3**^2+^, **4**^2+^, **5**^2+^, and **6**^2+^ are suitable for ON/OFF switching of magnetic properties by redox interconversion between the dications and their corresponding open-shell **T** forms. In the case of **2**^2+^, both the most stable closed-shell **2F** and metastable open-shell **2T** in the neutral state can be isolated and are stable enough even under ambient conditions so that the switching behavior among three states, **2**^2+^, **2T**, and **2F**, is completely controllable and a magnetic property can be changed not only by the redox interconversion between **2**^2+^ and **2T** but also by thermal conversion between **2T** and **2F**. Moreover, the difference between the oxidation potentials of **2T** and **2F** (Δ*E* = 1.32 V in CH_2_Cl_2_) is the largest change among the values reported to date (Figure 5).^58,65^

In this way, we have constructed a family of molecular switches composed of 9,10-anthrylene(s), in which the switching behavior can be precisely tuned by selecting the number of anthrylene unit(s) between two dibenzotropyliums. The “cation-capped orthogonal geometry” should provide valuable guidelines for the molecular design of arylene-based response systems because preliminary DFT calculations indicated that the number of arylene units, which would be suitable for realizing three-state switching, could be tuned by modifying the anthrylene skeletons or changing the cationic moieties for end-capping while maintaining the orthogonally twisted structure.

## Conclusions

We have designed and synthesized a family of dications with one to six anthrylene unit(s) composed of pure hydrocarbons by end-capping with dibenzotropylium skeletons as key building blocks. Our approach enabled isolation of the non-substituted oligo(9,10-anthrylene) derivatives even in the case of six anthrylene units, which is the largest number ever reported. Furthermore, they are soluble enough to perform various measurements due to the scarcity of intermolecular interactions in the crystal form, and thus we were able to elucidate their orthogonally twisted structures and unique redox properties.

Based on voltammetric analyses of these dications, all derivatives exhibited reversible oxidation wave(s), where each anthrylene unit underwent one-by-one oxidation resulting in the formation of multivalent cations. These dications are appropriate systems for investigating the electronic properties of individual anthrylene units in oligoanthrylenes because the electronic interaction between each 14π-aromatic unit is very weak due to the orthogonally twisted structure. In fact, the relationship between the oxidation potentials and the number of anthrylene unit(s) in each oxidation process was clarified in detail. Therefore, this study should provide important insights into applications for molecular electronics such as single-molecule memory or transistors because these dications can be considered a suitable model, in which individual molecules are forced to be close to each other.

These dications undergo 2e-reduction to give the corresponding closed-shell and/or open-shell neutral species as stable entities depending on the number of anthrylene unit(s), which can be handled under air at ambient temperature. Meanwhile, switching between the dication and closed-shell folded species was observed for monoanthrylene derivative **1** and that between the dication and open-shell biradical species was demonstrated for longer derivatives **3**–**6** upon redox interconversion. Particularly in the case of bianthrylene derivative **2**, since neutral species were isolated as both a kinetically produced biradical and thermodynamically stable folded structure, changes in the color, oxidation properties, and magnetic properties were demonstrated based on three-state interconversion. Although some studies have sought to construct switching systems based on interconversion between anthrylene-based open-shell species and anthraquinodimethane-based closed-shell species,^64−70^ the isolation of both structures is still challenging, especially under air at ambient temperature, and thus, a series of the molecular switches in this study could pave the way for the development of functional π-conjugated molecules.

In conclusion, we demonstrated that molecular design under the “cation-capping approach” could be a versatile strategy for stabilizing intrinsically unstable molecules and overcoming the solubility problem so that this approach could provide valuable guidelines for constructing and investigating unexploited molecular skeletons.

## Supporting Information Available

Synthetic details, characterization data, theoretical study, spectra, voltammetric analyses, and crystallographic details (PDF)
