# Supplementary material for: Dibenzotropylium-Capped Orthogonal Geometry Enabling Isolation and Examination of a Series of Hydrocarbons with Multiple 14π-Aromatic Units
Source: J Am Chem Soc. 2023 Jan 6;145(4):2596–608. doi: 10.1021/jacs.2c12574 (PMC9896550; doi:10.1021/jacs.2c12574)
Supplement: Supplementary file 1 — ja2c12574_si_001.pdf [file ja2c12574_si_001.pdf]

Supporting Information

Dibenzotropylium-Capped Orthogonal Geometry Enabling Isolation and Examination of a Series of Hydrocarbons with Multiple 14π-Aromatic Units

Yuki Hayashi[a], Shuichi Suzuki[b], Takanori Suzuki[a], and Yusuke Ishigaki[a]*

[a] Department of Chemistry, Faculty of Science, Hokkaido University, Sapporo 060-0810, Japan
[b] Graduate School of Engineering Science, Osaka University, Osaka 560-8531, Japan

*Correspondence to: yishigaki@sci.hokudai.ac.jp

Table of Contents

General S2
Experimental Section (Scheme S1, Figures S1-S24) S3
  Synthetic procedures S3
  NMR spectra of new compounds S21
Theoretical Study (Figures S25-S36, Table S1) S45
  DFT calculations of dications at the CAM-B3LYP/6-31G(d) level S45
  TD-DFT calculations of dications at the CAM-B3LYP/6-31G(d) level S49
  DFT calculations of neutral species at the (U)B3LYP/6-31G(d) level S55
Single-Crystal X-ray Structure Analyses (Tables S2 and S3, Figures S37 and S38) S64
  Crystal data S66
Voltammetric Analyses (Figures S39-S43) S67
Spectroscopic Analyses (Table S4, Figures S44-S52) S71
  UV-Vis-NIR spectra of dications S71
  ESR spectra of biradicals S71
  Electrochemical reduction and oxidation S72
  UV-Vis spectra of isolated neutral species S73
  Comparison of UV-Vis spectra of neutral species S74
  IR spectra of dications and corresponding neutral species S75
  Thermal isomerization from 2T to 2F S76
Optimized Coordinates S77
References S127

General

All reactions were carried out under an argon atmosphere. All commercially available compounds were used without further purification unless otherwise indicated. Dry MeCN was obtained by distillation from CaH_2 prior to use. Column chromatography was performed on silica gel 60N (KANTO KAGAKU, spherical neutral) of particle size 40–50 μm or Wakogel® 60N (neutral) of particle size 38-100 μm. ^{1}H and ^{13}C NMR spectra were recorded on a BRUKER Ascend™ 400 (^{1}H/400 MHz and ^{13}C/100 MHz) spectrometer. ESR spectra were recorded on a JEOL JES-FE2XG spectrometer. IR spectra were measured on a Shimadzu IRAffinity-1S spectrophotometer using the attenuated total reflection (ATR) mode. Mass spectra were recorded on a JEOL JMS-T100GCV spectrometer in FD mode, on a Thermo Fisher Scientific Q Exactive Plus spectrometer in ESI positive mode or on a BRUKER UltrafleXtreme-DHS2 TOF/TOF in MALDI positive mode with α-cyano-4-hydroxycinnamic acid (CHCA) as matrix by Dr. Eri Fukushi and Mr. Yusuke Takata (GC-MS&NMR Laboratory, Research Faculty of Agriculture, Hokkaido University). Melting points were measured on a Stanford Research Systems MPA100 Optimelt and are uncorrected. UV-Vis-NIR spectra were recorded on a JASCO V-770 spectrophotometer. Fluorescence spectra were measured on a Hitachi F-7000 spectrofluorometer. Fluorescence quantum yields were determined by using 9,10-diphenylanthracene (Φ_F = 0.97) as an external standard.[1] Redox potentials (E^{ox} and E^{red}) were measured on a BAS ALS-612EX by differential pulse voltammetry and cyclic voltammetry. Pt electrodes were used as the working (disk) and counter electrodes. The working electrode was polished using a water suspension of aluminum oxide (0.05 μm) before use. DFT calculations were performed with the Gaussian 16 W program package.[2] The geometries of the compounds were optimized by using the (U)B3LYP or (U)CAM-B3LYP method in combination with the 6–31G(d) basis set. Single-crystal X-ray structure analyses were performed by a Rigaku XtaLAB Synergy (Cu-Kα radiation, λ = 1.54184 Å) with HyPix diffractometer. Using Olex2,[3] the structure was solved with the SHELXT[4] structure solution program using Intrinsic Phasing and refined with the SHELXL[5] refinement package using Least Squares minimization. All the hydrogen atoms were located at the calculated positions and refined with riding.

Experimental Section (Scheme S1, Figures S1-S24)

Synthetic procedures

Scheme S1. Synthetic route for preparing ketones **S4** and **S7**.

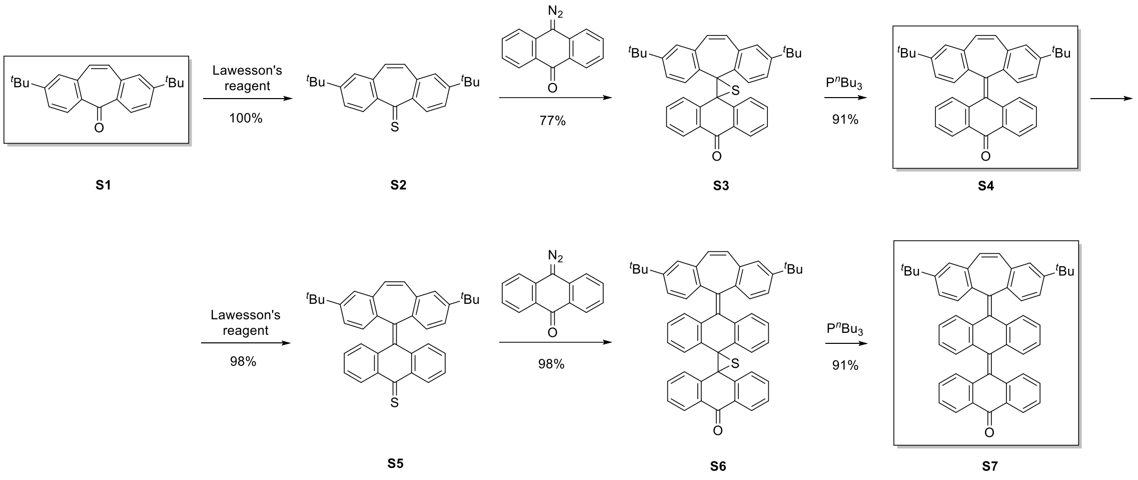

2,8-Di-*tert*-butyl-5*H*-dibenzo[*a,d*]cycloheptatriene-5-thione S2.

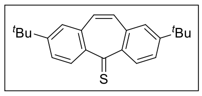

A solution of ketone **S1**[6] (1.10 g, 3.46 mmol) and 2,4-bis(4-methoxyphenyl)-1,3-dithia-2,4-diphosphetan-2,4-disulfide (Lawesson's reagent) (770 mg, 1.90 mmol) in dry toluene (35 mL) was heated to reflux for 4 h. After cooling to 25 °C, the resulting green solution was concentrated under reduced pressure. The crude product was purified by column chromatography on silica gel (CH_2Cl_2/hexane = 1/1, Rf = 0.60) to give thione **S2** (1.21 g) as a green solid in 100% yield.

S2; Mp: 124.0 °C-126.3 °C; ^{1}H NMR ($CDCl_3$): δ /ppm 8.04 (2H, d, J= 8.4 Hz), 7.43 (2H, dd, J= 2.0 Hz, 8.4 Hz), 7.33 (2H, d, J= 2.0 Hz), 7.00 (2H, s), 1.34 (18H, s); ^{13}C NMR ($CDCl_3$): δ/ppm 238.15, 154.03, 146.92, 131.82, 130.63, 129.71, 126.21, 125.22, 34.82, 31.02; IR (ATR): ν/cm^{-1} 3051, 3025, 2960, 2901, 2864, 1917, 1772, 1668, 1641, 1597, 1539, 1476, 1458, 1381, 1361, 1311, 1294, 1271, 1253, 1235, 1196, 1177, 1149, 1112, 1020, 977, 961, 944, 900, 887, 843, 834, 799, 793, 740, 703, 694, 677, 661, 622, 539, 502, 456; LR-MS (FD) m/z (%): 669.39 (5), 668.38 (9), 336.19 (8) , 335.20 (25), 334.19 (M^+, bp), 318.22 (6); HR-MS (FD) Calcd. for $C_{23}H_{26}S$: 334.17552; Found: 334.17504.

2'',8''-Di-*tert*-butyl-10*H*-dispiro[anthracene-9,2'-thiirane-3',5''-dibenzo[*a,d*]cycloheptatrien]-10-one S3

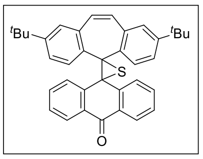

A solution of thione **S2** (1.16 g, 3.46 mmol) and 10-diazoanthracen-9(10*H*)-one[7] (763 mg, 3.46 mmol) in dry THF (35 mL) was heated to reflux for 14 h. After cooling to 25 °C, the precipitates were collected and washed with

ethanol three times. The resulting solid was dried in vacuo to give **S3** (1.40 g) as a yellow solid in 77% yield.

S3; Mp: 204.0 °C-208.2 °C (decomp.); ^{1}H NMR ($CDCl_3$): δ /ppm 8.06 (2H, dd, J= 0.8 Hz, 7.2 Hz), 7.64 (2H, d, J= 8.0 Hz), 7.27 (2H, dd, J= 2.0 Hz, 8.0 Hz), 7.15 (2H, ddd, J= 0.8 Hz, 7.2 Hz, 7.2 Hz), 7.14 (2H, d, J= 7.2 Hz), 6.90 (2H, ddd, J= 0.8 Hz, 7.2 Hz, 7.2 Hz), 6.70 (2H, d, J= 2.0 Hz), 6.23 (2H, s), 1.16 (18H, s); ^{13}C NMR ($CDCl_3$): δ/ppm 184.90, 149.94, 139.84, 134.78, 134.09, 133.99, 132.03, 130.39, 129.17, 128.31, 127.11, 126.53, 124.86, 123.88, 68.53, 56.84, 34.28, 31.22; IR (ATR): ν/cm^{-1} 3062, 3028, 2957, 2901, 2866, 1667, 1595, 1496, 1477, 1457, 1387, 1361, 1317, 1278, 1244, 1198, 1171, 1161, 1112, 1091, 1044, 963, 950, 933, 910, 893, 878, 829, 821, 814, 785, 763, 717, 689, 678, 662, 646, 620, 613, 571, 535, 520, 497, 474, 452; LR-MS (FD) m/z (%): 528.25 (14), 527.25 (43), 526.25 (M^+, bp); HR-MS (FD) Calcd. for $C_{37}H_{34}OS$: 526.23304; Found: 526.23141.

10-(2,8-Di-*tert*-butyl-5*H*-dibenzo[*a,d*]cycloheptatrien-5-ylidene)anthracen-9(10*H*)-one S4

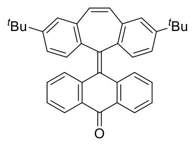

A solution of **S3** (1.36 g, 2.58 mmol) and tri-*n*-butylphosphine (638 mg, 3.15 mmol) in dry toluene (30 mL) was heated to reflux for 15 h. After cooling to 25 °C, the precipitates were collected and washed with hexane three times. The resulting solid was dried in vacuo to give ketone **S4** (1.16 g) as a pale-yellow solid in 91% yield.

S4; Mp: 305.8 °C-309.1 °C (decomp.); ^{1}H NMR ($CDCl_3$): δ /ppm 8.10 (2H, dd, J= 1.2 Hz, 7.6 Hz), 7.44 (2H, d, J= 2.0 Hz), 7.26 (2H, ddd, J= 1.2 Hz, 7.6 Hz, 7.6 Hz), 7.20 (2H, s), 7.14 (2H, dd, J= 2.0 Hz, 8.4 Hz), 6.96 (2H, ddd, J= 1.2 Hz, 7.6 Hz, 8.0 Hz), 6.70 (2H, d, J= 8.4 Hz), 6.52 (2H, dd, J= 1.2 Hz, 8.0 Hz), 1.31 (18H, s); ^{13}C NMR ($CDCl_3$): δ/ppm 186.26, 150.37, 142.49, 139.24, 135.89, 134.51, 133.46, 131.55, 129.86, 129.43, 128.88, 127.76, 127.01, 126.31, 126.16, 124.59, 34.57, 31.38; IR (ATR): ν/cm^{-1} 3062, 3027, 2952, 2902, 2864, 1772, 1659, 1597, 1465, 1457, 1384, 1360, 1306, 1282, 1202, 1173, 1162, 1151, 1135, 1114, 1092, 1037, 984, 963, 930, 908, 889, 858, 830, 822, 798, 779, 749, 717, 691, 679, 654, 642, 620, 603, 546, 509, 502, 458, 419; LR-MS (FD) m/z (%): 496.27 (9), 495.27 (42), 494.27 (M^+, bp); HR-MS (FD) Calcd. for $C_{37}H_{34}O$: 494.26096; Found: 494.26056.

10-(2,8-Di-*tert*-butyl-5*H*-dibenzo[*a,d*]cycloheptatrien-5-ylidene)anthracene-9(10*H*)-thione S5

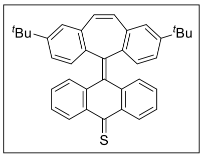

A solution of ketone **S4** (1.27 g, 2.57 mmol) and 2,4-bis(4-methoxyphenyl)-1,3-dithia-2,4-diphosphetan-2,4-disulfide (Lawesson's reagent) (571 mg, 1.41 mmol) in dry toluene (27 mL) was heated to reflux for 4 h. After cooling to 26 °C, the resulting green solution was concentrated under reduced pressure.

The crude product was purified by column chromatography on silica gel (CH_2Cl_2/hexane = 1/1, Rf = 0.65) to give thione **S5** (1.29 g) as a green solid in 98% yield.

S5; Mp: 273.5 °C-274.3 °C (decomp.); ^{1}H NMR ($CDCl_3$): δ /ppm 8.31 (2H, dd, J= 1.2 Hz, 8.0 Hz), 7.44 (2H, d, J= 2.0 Hz), 7.20 (2H, s), 7.18 (2H, ddd, J= 1.2 Hz, 7.2 Hz, 8.0 Hz), 7.11 (2H, dd, J= 2.0 Hz, 8.0 Hz), 6.98 (2H, ddd, J= 1.2 Hz, 7.2 Hz, 8.0 Hz), 6.71 (2H, d, J= 8.0 Hz), 6.50 (2H, dd, J= 1.2 Hz, 8.0 Hz), 1.31 (18H, s); ^{13}C NMR ($CDCl_3$): δ/ppm 223.11, 150.40, 142.71, 141.64, 135.96, 134.34, 132.89, 131.55, 130.53, 129.44, 128.67, 128.12, 127.50, 126.89, 126.24, 124.60, 34.55, 31.34; IR (ATR): ν/cm^{-1} 3060, 3022, 2960, 2902, 2865, 1599, 1487, 1465, 1458, 1383, 1361, 1310, 1297, 1275, 1254, 1225, 1212, 1174, 1154, 1137, 1122, 1098, 1030, 959, 907, 886, 854, 829, 822, 796, 788, 771, 737, 705, 687, 656, 635, 621, 613, 607, 601, 505, 500, 452, 430; LR-MS (FD) m/z (%): 512.21 (15), 511.21 (43), 510.21 (M$^+$, bp); HR-MS (FD) Calcd. for $C_{37}H_{34}S$: 510.23812; Found: 510.23846.

10''-(2,8-Di-*tert*-butyl-5*H*-dibenzo[*a,d*]cycloheptatrien-5-ylidene)-10*H*,10''*H*-dispiro[anthracene-9,2'-thiirane-3',9''-anthracen]-10-one S6

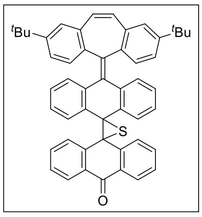

A solution of thione **S5** (1.15 g, 2.25 mmol) and 10-diazoanthracen-9(10*H*)-one[7] (496 mg, 2.25 mmol) in dry THF (23 mL) was heated to reflux for 14 h. After cooling to 25 °C, the precipitates were collected and washed with ethanol three times. The resulting solid was dried in vacuo to give **S6** (1.56 g) as a yellow solid in 98% yield.

S6; Mp: 224.1 °C-229.3 °C (decomp.); ^{1}H NMR ($CDCl_3$): δ /ppm 8.24-8.21 (2H, m), 7.98-7.95 (2H, m), 7.66 (2H, dd, J= 1.2 Hz, 8.0 Hz), 7.46-7.40 (4H, m), 7.25 (2H, d, J= 2.0 Hz), 6.96 (2H, s), 6.89 (2H, ddd, J= 1.2 Hz, 8.0 Hz, 8.0 Hz), 6.80 (2H, dd, J= 2.0 Hz, 8.0 Hz), 6.52 (2H, ddd, J= 1.2 Hz, 8.0 Hz, 8.0 Hz), 6.16 (2H, d, J= 8.0 Hz), 6.07 (2H, dd, J= 0.8 Hz, 8.0 Hz), 1.26 (18H, s); ^{13}C NMR ($CDCl_3$): δ/ppm 184.11, 149.48, 139.43 (2C), 138.06, 135.94, 135.48, 134.97, 134.45, 131.75, 131.58, 131.43, 129.45, 128.56, 128.27, 128.01, 127.69, 127.06, 125.65, 124.98, 124.58, 124.51, 66.63, 59.77, 34.33, 31.35; IR (ATR): ν/cm^{-1} 3056, 3025, 2962, 2902, 2866, 1667, 1596, 1466, 1457, 1383, 1362, 1312, 1267, 1204, 1172, 1159, 1135, 1116, 1092, 1042, 932, 900, 886, 851, 821, 801, 792, 782, 768, 750, 731, 704, 694, 688, 664, 649, 632, 610, 575, 519, 498, 475, 452, 436, 418; LR-MS (FD) m/z (%): 705.30 (9), 704.30 (24), 703.30 (60), 702.30 (M$^+$, bp); HR-MS (FD) Calcd. for $C_{51}H_{42}OS$: 702.29564; Found: 702.29704.

10'-(2,8-Di-*tert*-butyl-5*H*-dibenzo[*a,d*]cycloheptatrien-5-ylidene)-10*H*, 10'*H*-[9,9'-bianthracenylidene]-10-one S7 (X-ray analysis in Figure S37)

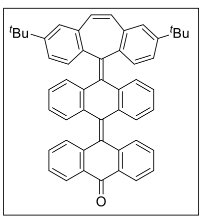

A solution of **S6** (1.49 g, 2.12 mmol) and tri-*n*-butylphosphine (693 mg, 3.43 mmol) in dry toluene (20 mL) was heated to reflux for 14 h. After cooling

to 25 °C, the precipitates were collected and washed with hexane three times. The resulting solid was dried in vacuo to give ketone **S7** (1.29 g) as a pale yellow solid in 91% yield.

S7; Mp: 196.9 °C-206.3 °C (decomp.); ^{1}H NMR ($CDCl_3$): δ /ppm 8.19 (2H, dd, J= 1.2 Hz, 8.0 Hz), 7.46 (2H, d, J= 2.0 Hz), 7.45-7.41 (4H, m), 7.31 (2H, ddd, J= 1.2 Hz, 7.6 Hz, 9.2 Hz), 7.28 (2H, ddd, J= 2.0 Hz, 8.0 Hz), 7.19 (2H, s), 7.16 (2H, d, J= 8.0 Hz), 6.87-6.83 (2H, m), 6.77-6.72 (4H, m), 6.55-6.50 (2H, m), 1.36 (18H, s); ^{13}C NMR ($CDCl_3$): δ/ppm 186.61, 150.16, 139.37, 138.67, 137.96, 137.84, 137.81, 136.27, 135.01, 134.29, 134.11, 131.76, 130.04, 129.87, 128.97, 128.79, 127.89, 127.69, 126.79, 126.64, 125.90, 125.86, 125.00, 124.89, 34.58, 31.43; IR (ATR): ν/cm^{-1} 3064, 3022, 2955, 2903, 2867, 1663, 1598, 1496, 1457, 1450, 1383, 1361, 1305, 1275, 1202, 1170, 1144, 1092, 1038, 950, 931, 908, 896, 866, 844, 819, 798, 778, 766, 724,699, 688, 679, 657, 636, 590, 517, 499, 494, 454; LR-MS (FD) m/z (%): 673.35 (5), 672.35 (19), 671.34 (58), 670.34 (M^+, bp); HR-MS (FD) Calcd. for $C_{51}H_{42}O$: 670.32356; Found: 670.32242.

5,5'-(Anthracene-9,10-diyl)-bis(2,8-di-*tert*-butyl-5*H*-dibenzo[*a,d*]cycloheptatrien-5-ol) 1-OH

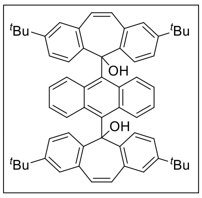

To a solution of 9,10-dibromoanthracene (233 mg, 0.694 mmol) in dry Et_2O (10 mL) was added ^{n}BuLi (1.58 M in hexane, 1.05 mL, 1.67 mmol) dropwise over 2 min at –20 °C. After stirring at –20 °C for 1 h, **S1** (530 mg, 1.67 mmol) was added to the suspension and the mixture was warmed to 22 °C. The resulting solution was stirred at 22 °C for 1 h, and then diluted with water. The precipitates were collected and washed with water three times and with methanol three times. The resulting solid was washed with $CHCl_3$ dried in vacuo to give diol **1-OH** (241 mg) as a yellow solid in 43% yield.

1-OH; Mp: 233.0 °C-251.2 °C (decomp.); ^{1}H NMR (DMSO-d_6): δ /ppm 8.23 (4H, d, J= 8.8 Hz), 7.58-7.54 (8H, m), 7.14 (2H, s), 7.06 (4H, d, J= 1.6 Hz), 6.41 (4H, s), 6.36 (4H, dd, J= 3.2 Hz, 7.2 Hz), 1.27 (36H, s); ^{13}C NMR (DMSO-d_6): δ/ppm 147.42, 144.74, 138.11, 131.68, 131.38, 130.16, 126.40, 125.67, 124.04, 122.40, 120.87, 78.39, 34.53, 31.72; IR (ATR): ν/cm^{-1} 3544, 3119, 3072, 3033, 3019, 2958, 2902, 2866, 1603, 1559, 1527, 1490, 1461, 1444, 1387, 1363, 1299, 1266, 1202, 1188, 1163, 1132, 1096, 1063, 1041, 990, 942, 905, 893, 829, 816, 801, 758, 736, 704, 699, 683, 653, 640, 622, 615, 547, 506, 482, 419; LR-MS (FD) m/z (%): 816.48 (20), 815.48 (66), 814.48 (M^+, bp), 576.19 (6), 574.19 (6), 497.28 (6), 496.28 (11), 318.20 (22); HR-MS (FD) Calcd. for $C_{60}H_{62}O_2$: 814.47498; Found: 814.47532.

5,5'-([9,9'-Bianthracene]-10,10'-diyl)-bis(2,8-di-*tert*-butyl-5*H*-dibenzo[*a,d*]cycloheptatrien-5-ol) 2-OH

To a solution of 10,10'-dibromo-9,9'-bianthracene (362 mg, 0.706 mmol) in dry THF (8 mL) was added ^{n}BuLi (1.58 M in hexane, 1.07 mL, 1.69 mmol) dropwise over 2 min at –78 °C. After

stirring at –78 °C for 1 h, **S1** (538 mg, 1.69 mmol) was added to the suspension and the mixture was warmed to 21 °C. The resulting solution was stirred at 21 °C for 1 h, and then diluted with water. The whole mixture was extracted with EtOAc three times. The combined organic layers were washed with water and brine, and dried over anhydrous Na_2SO_4. After filtration, the solvent was concentrated under reduced pressure. The crude product was purified by column chromatography on silica gel (CH_2Cl_2/hexane = 1/1, Rf = 0.27) to give diol **2-OH** (340 mg) as a yellow solid in 49% yield.

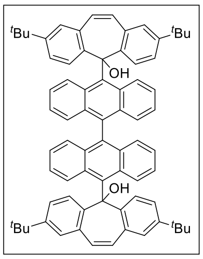

2-OH; Mp: 221.9 °C-225.4 °C (decomp.); ^{1}H NMR ($CDCl_3$): δ /ppm 8.43 (4H, d, J= 8.8 Hz), 7.66 (2H, dd, J= 0.8 Hz, 9.2 Hz), 7.62 (4H, dd, J= 1.6 Hz, 8.8 Hz), 7.42 (2H, dd, J= 0.8 Hz, 9.2 Hz), 6.98-6.88 (10H, m), 6.76 (2H, ddd, J= 0.8 Hz, 6.4 Hz, 8.4 Hz), 6.69 (2H, ddd, J= 2.4 Hz, 5.6 Hz, 9.2 Hz), 6.63 (2H, ddd, J= 1.6 Hz, 6.4 Hz, 9.2 Hz), 6.17 (4H, s), 3.70 (2H, s), 1.29 (18H, s), 1.28 (18H, s); ^{13}C NMR ($CDCl_3$): δ/ppm 148.42, 148.38, 143.77, 143.66, 136.37, 135.30, 131.61, 131.55, 131.24, 131.19, 130.93, 130.88, 130.18, 129.78, 126.67, 126.26, 125.81, 125.62, 125.56, 125.46, 124.78, 124.70, 124.37, 124.00, 122.52, 122.47, 121.66, 121.58, 79.87, 34.35, 31.43; IR (ATR): ν/cm^{-1} 3559, 3062, 3024, 2902, 2866, 1918, 1602, 1555, 1521, 1489, 1458, 1444, 1387, 1362, 1315, 1266, 1209, 1186, 1165, 1134, 1095, 1058, 1034, 991, 949, 906, 889, 883, 836, 821, 798, 756, 732, 699, 684, 676, 662, 633, 611, 578, 548, 507, 477, 449, 420; LR-MS (FD) m/z (%): 993.55 (10), 992.55 (36), 991.55 (83), 990.54 (M^+, bp),; HR-MS (FD) Calcd. for $C_{74}H_{70}O_2$: 990.53758; Found: 990.53711.

10,10''-Bis(2,8-di-*tert*-butyl-5*H*-dibenzo[*a,d*]cycloheptatrien-5-ylidene)-[9,9':10',9''-teranthracene]-9,9''(10*H*,10''*H*)-diol 3-OH

To a solution of 9,10-dibromoanthracene (300 mg, 0.894 mmol) in dry Et_2O (15 mL) was added ^{n}BuLi (1.58 M in hexane, 1.36 mL, 2.15 mmol) dropwise over 2 min at –20 °C. After stirring at –20 °C for 1 h, **S4** (1.06 g, 2.15 mmol) was added to the suspension and the mixture was warmed to 21 °C. The resulting solution was stirred at 21 °C for 1 h, and then diluted with water. The whole mixture was extracted with CH_2Cl_2 three times. The combined organic layers were washed with water and brine, and dried over anhydrous Na_2SO_4. After filtration, the solvent was concentrated under reduced pressure. The crude product was purified by column chromatography on silica gel (toluene/hexane = 2/1, Rf = 0.067) to give diol **3-OH** (432 mg) as a yellow solid in 41% yield as a mixture of diastereomers, which were identified by FD-MS.

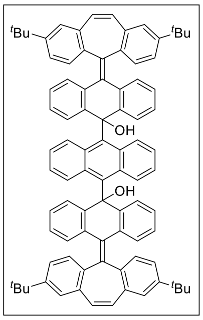

3-OH; Mp: 219.2 °C-229.6 °C (decomp.); IR (ATR): ν/cm^{-1} 3531, 3060, 3022, 2954, 2902, 2866, 2367, 1675, 1601, 1495, 1458, 1444, 1383, 1361, 1304, 1284, 1269, 1252, 1202, 1169, 1135, 1116, 1079, 1030, 992, 951, 907, 890, 856, 838, 822, 795, 780, 758, 729, 700, 654, 631, 619, 501, 452, 425, 419; LR-MS (FD) m/z (%): 1170.61 (6), 1169.61 (18), 1168.61 (49), 1167.60

(99), 1166.60 (M^+, bp), 1151.61 (8), 1150.60 (12), 1149.59 (12), 673.35 (6), 672.34 (10), 583.80 (7), 495.27 (6), 494.26 (13); HR-MS (FD) Calcd. for $C_{88}H_{78}O_2$: 1166.60018; Found: 1166.60194.

10,10'''-Bis(2,8-di-*tert*-butyl-5*H*-dibenzo[*a,d*]cycloheptatrien-5-ylidene)-[9,9':10',9'':10'',9'''-quateranthracene]-9,9'''(10*H*,10'''*H*)-diol 4-OH

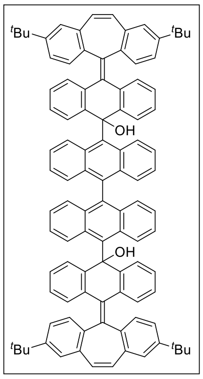

To a solution of 10,10'-dibromo-9,9'-bianthracene (393 mg, 0.767 mmol) in dry THF (10 mL) was added ^{n}BuLi (1.58 M in hexane, 1.16 mL, 1.84 mmol) dropwise over 2 min at –78 °C. After stirring at –78 °C for 1 h, **S4** (911 mg, 1.84 mmol) was added to the suspension and the mixture was warmed to 26 °C. The resulting solution was stirred at 26 °C for 1 h, and then diluted with water. The whole mixture was extracted with CH_2Cl_2 three times. The combined organic layers were washed with water and brine, and dried over anhydrous Na_2SO_4. After filtration, the solvent was concentrated under reduced pressure. The crude product was purified by column chromatography on silica gel (CH_2Cl_2/hexane = 2/1, Rf = 0.13) to give diol **4-OH** (378 mg) as a yellow solid in 37% yield as a mixture of diastereomers, which were identified by FD-MS.

4-OH; Mp: 271.0 °C-281.1 °C (decomp.); IR (ATR): ν/cm^{-1} 3537, 3062, 3019, 2959, 2903, 2867, 1601, 1521, 1490, 1458, 1442, 1383, 1361, 1306, 1269, 1252, 1169, 1135, 1116, 1079, 1034, 992, 951, 908, 894, 871, 854, 838, 823, 796, 780, 761, 730, 679, 655, 627, 617, 605, 566, 536, 502, 451, 431, 419; LR-MS (FD) m/z (%): 1346.67 (8), 1345.67 (22), 1344.66 (56), 1343.66 (bp), 1342.66 (M^+, 89), 1327.66 (6), 1326.65 (9), 1325.65 (8), 671.83 (7), 671.33 (M^{2+}, 7), 496.28 (6), 495.27 (5), 494.26 (9); HR-MS (FD) Calcd. for $C_{102}H_{86}O_2$: 1342.66278; Found: 1342.66472.

10,10''''-Bis(2,8-di-*tert*-butyl-5*H*-dibenzo[*a,d*]cycloheptatrien-5-ylidene)-9'''*H*,10*H*,10'*H*, 10''''*H*-[9,9':10',9'':10'',9''':10''',9''''-quinqueanthracene]-9''',10'-diol 5-OH

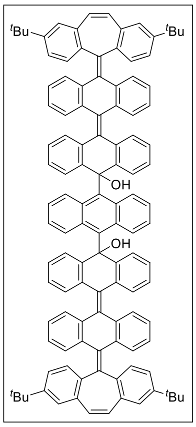

To a solution of 9,10-dibromoanthracene (236 mg, 0.702 mmol) in dry Et_2O (10 mL) was added ^{n}BuLi (1.58 M in hexane, 1.07 mL, 1.68 mmol) dropwise over 2 min at –20 °C. After stirring at –20 °C for 1 h, **S7** (1.13 g, 1.68 mmol) was added to the suspension and the mixture was warmed to 24 °C. The resulting solution was stirred at 24 °C for 1 h, and then diluted with water. The whole mixture was extracted with CH_2Cl_2 three times. The combined organic layers were washed with water and brine, and dried over anhydrous Na_2SO_4. After filtration, the solvent was concentrated under reduced pressure. The crude product was purified by column chromatography on silica gel (toluene/hexane = 3/1, Rf = 0.089) to give diol **5-OH** (482 mg) as a yellow solid in 45% yield as a mixture of diastereomers, which were identified by FD-MS.

5-OH; Mp: 241.2 °C-251.3 °C (decomp.); IR (ATR): ν/cm^{-1} 3567, 3061, 3021, 2960, 2903, 2865, 1675, 1599, 1490, 1457, 1448, 1384, 1361, 1304, 1284, 1264, 1252, 1203, 1170, 1118, 1093, 1039, 992, 951, 908, 891, 836, 818, 795, 759, 732, 700, 677, 664, 657, 633, 611, 582, 455; LR-MS (FD) m/z (%): 1522.71 (9), 1521.71 (28), 1520.70 (63), 1519.71 (bp), 1518.70 (M^+, 78), 1505.78 (6), 1504.71 (11), 1503.71 (18), 1502.70 (23), 1501.70 (18), 848.38 (6), 760.85 (5), 760.35 (9), 759.85 (M^{2+}, 14), 759.35 (11), 751.87 (6), 751.35 (7), 750.85 (5), 672.33 (5), 671.32 (6), 670.31 (7), 208.05 (6); HR-MS (FD) Calcd. for $C_{116}H_{94}O_2$: 1518.72538; Found: 1518.72590.

10,10'''''-Bis(2,8-di-*tert*-butyl-5*H*-dibenzo[*a,d*]cycloheptatrien-5-ylidene)-9''''*H*,10*H*,10'*H*, 10'''''*H*-[9,9':10',9'':10'',9''':10''',9'''':10'''',9'''''-sexianthracene]-9'''',10'-diol 6-OH

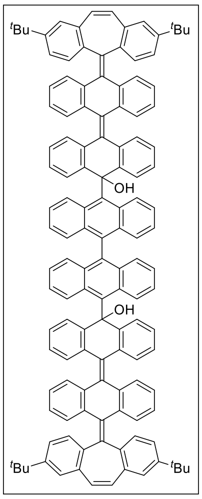

To a solution of 10,10'-dibromo-9,9'-bianthracene (354 mg, 0.691 mmol) in dry THF (8 mL) was added ^{n}BuLi (1.58 M in hexane, 1.05 mL, 1.66 mmol) dropwise over 2 min at –78 °C. After stirring at –78 °C for 1 h, **S7** (1.11 g, 1.66 mmol) was added to the suspension and the mixture was warmed to 26 °C. The resulting solution was stirred at 26 °C for 1 h, and then diluted with water. The whole mixture was extracted with CH_2Cl_2 three times. The combined organic layers were washed with water and brine, and dried over anhydrous Na_2SO_4. After filtration, the solvent was concentrated under reduced pressure. The crude product was purified by column chromatography on silica gel (toluene/hexane = 3/1, Rf = 0.022) to give diol **6-OH** (682 mg) as a yellow solid in 58% yield as a mixture of diastereomers, which were identified by FD-MS.

6-OH; Mp: 283.5 °C-296.7 °C (decomp.); IR (ATR): ν/cm^{-1} 3566, 3061, 3022, 2958, 2902, 2866, 1671, 1599, 1457, 1448, 1384, 1361, 1305, 1270, 1252, 1170, 1119, 1093, 1034, 993, 951, 932, 908, 890, 839, 818, 795, 760, 732, 711, 694, 676, 659, 637, 632, 606, 592, 583, 452; LR-MS (FD) m/z (%): 1698.74 (11), 1697.74 (38), 1696.74 (73), 1695.73 (bp), 1694.73 (M^+, 77), 1693.73 (8), 1682.78 (5), 1681.74 (10), 1680.75 (18), 1679.74 (31), 1678.74 (39), 1677.73 (29), 848.87 (5), 848.36 (8), 847.86 (11), 847.36 (M^{2+}, 8), 840.95 (5), 840.40 (10), 839.86 (14), 839.36 (15), 838.86 (10), 831.90 (6), 831.37 (8), 830.85 (8), 830.36 (6), 671.30 (11), 670.30 (16); HR-MS (FD) Calcd. for $C_{130}H_{102}O_2$: 1694.78798; Found: 1694.78725.

5,5'-(Anthracene-9,10-diyl)-bis(2,8-di-*tert*-butyl-5*H*-dibenzo[*a,d*]cycloheptatrien-5-ylium) bis(tetrafluoroborate) $1^{2+}(BF_4^-)_2$

<u>Dehydration of **1-OH** with HBF_4:</u>

To a solution of diol **1-OH** (34.8 mg, 42.7 μmol) in trifluoroacetic anhydride (TFAA, 2.0 mL) was added 42% HBF_4 aq. (64 μL, 427 μmol) at 0 °C to give a deep red solution, and the mixture

was stirred at 25 °C for 2 h. The addition of dry Et_2O led to precipitation of the dication salt. The supernatant solution was removed by decantation, and the precipitates were washed with dry Et_2O three times, and dried in vacuo to give $\mathbf{1}^{2+}(BF_4^-)_2$ (38.0 mg) as a red powder in 93% yield.

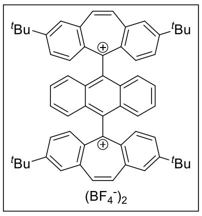

Oxidation of $\mathbf{1F}_{syn,anti}$ with $(4\text{-}BrC_6H_4)_3N^{+\bullet}BF_4^-$:

To a solution of $\mathbf{1F}_{syn,anti}$ (12.6 mg, 17.5 μmol) in dry CH_2Cl_2 (1 mL) was added $(4\text{-}BrC_6H_4)_3N^{+\bullet}BF_4^-$ (20.0 mg, 35.1 μmol) at 20 °C, and the mixture was stirred for 2 h. The addition of dry hexane led to precipitation of the dication salt. The supernatant solution was removed by decantation, and the precipitates were washed with dry hexane three times, and dried in vacuo to give $\mathbf{1}^{2+}(BF_4^-)_2$ (16.6 mg) as a red powder in 99% yield.

$\mathbf{1}^{2+}(BF_4^-)_2$; Mp: 302.3 °C~308.8 °C (decomp.); ^{1}H NMR (CD_3CN): δ /ppm 9.48 (4H, s), 8.95 (4H, dd, J= 1.2 Hz, 1.2 Hz), 8.22 (8H, d, J= 1.2 Hz), 7.32 (4H, dd, J= 3.2 Hz, 7.2 Hz), 7.16 (4H, dd, J= 3.2 Hz, 7.2 Hz), 1.58 (36H, s); ^{13}C NMR (CD_3CN): δ/ppm 177.47, 167.48, 147.67, 145.93, 139.06, 138.18, 137.85, 133.41, 133.22, 130.72, 128.82, 127.22, 37.34, 30.24; IR (ATR): ν/cm^{-1} 3061, 2969, 2908, 2872, 1792, 1761, 1601, 1505, 1478, 1412, 1380, 1363, 1322, 1277, 1194, 1147, 1120, 1088, 1065, 1027, 914, 851, 827, 780, 755, 693, 676, 667, 659, 615, 608, 531, 514, 442, 431, 419; LR-MS (FD) m/z (%): 870.55 (6), 869.55 (24), 868.54 (67), 867.54 ($M^{2+}BF_4^-$, bp), 866.54 (23), 851.53 (5), 850.53 (16), 849.53 (21), 782.53 (12), 781.53 (30), 780.53 (M^+, 43); HR-MS (FD) Calcd. for $C_{60}H_{60}$: 780.46950; Found: 780.46943.

5,5'-(Anthracene-9,10-diyl)-bis(2,8-di-*tert*-butyl-5*H*-dibenzo[*a,d*]cycloheptatrien-5-ylium) bis(hexachloroantimonate) $\mathbf{1}^{2+}(SbCl_6^-)_2$

To a solution of $\mathbf{1F}_{anti,anti}$ (22.1 mg, 28.3 μmol) in dry CH_2Cl_2 (2 mL) was added $(2,4\text{-}Br_2C_6H_3)_3N^{+\bullet}SbCl_6^-$ (59.5 mg, 56.6 μmol) at 20 °C, and the mixture was stirred for 2 h. The addition of dry hexane led to precipitation of the dication salt. The supernatant solution was removed by decantation, and the precipitates were washed with dry hexane three times, and dried in vacuo to give $\mathbf{1}^{2+}(SbCl_6^-)_2$ (40.0 mg) as a red powder in 98% yield.

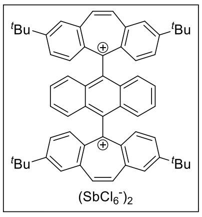

$\mathbf{1}^{2+}(SbCl_6^-)_2$; Mp: 247.8 °C~250.4 °C (decomp.); ^{1}H NMR and ^{13}C NMR spectra are identical to those of $\mathbf{1}^{2+}(BF_4^-)_2$; IR (ATR): ν/cm^{-1} 3060, 2964, 2929, 2902, 2868, 2365, 1607, 1506, 1476, 1444, 1410, 1380, 1367, 1359, 1345, 1321, 1271, 1261, 1211, 1116, 1022, 957, 918, 909, 887, 855, 823, 778, 763, 731, 702, 694, 676, 669, 656, 613, 513, 436, 429; LR-MS (FD) m/z (%): 852.34 (7), 851.34 (8), 850.34 (9), 849.33 (6), 817.37 (5), 816.37 (10), 815.37 (12), 814.37 (14), 810.38 (6), 796.40 (8), 795.39 (9), 784.39 (6), 783.40 (16), 782.40 (38), 781.41 (69), 780.41 (M^+, bp), 768.40 (7), 767.40 (10), 755.40 (6), 754.39 (9), 391.20 (5), 390.70 (12), 390.20 (M^{2+}, 18); HR-MS (FD) Calcd. for $C_{60}H_{60}$: 780.46950; Found: 780.46775.

5,5'-([9,9'-Bianthracene]-10,10'-diyl)-bis(2,8-di-*tert*-butyl-5*H*-dibenzo[*a,d*]cycloheptatrien-5-ylium) bis(tetrafluoroborate) $2^{2+}(BF_4^-)_2$

Dehydration of **2-OH** with HBF_4:

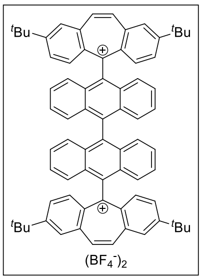

To a solution of diol **2-OH** (67.2 mg, 67.8 μmol) in trifluoroacetic anhydride (TFAA, 2.0 mL) was added 42% HBF_4 aq. (100 μL, 678 μmol) at 0 °C to give a deep red solution, and the mixture was stirred at 25 °C for 2 h. The addition of dry Et_2O led to precipitation of the dication salt. The supernatant solution was removed by decantation, and the precipitates were washed with dry Et_2O three times, and dried in vacuo to give $\mathbf{2}^{2+}(BF_4^-)_2$ (77.5 mg) as a red powder in 100% yield.

Oxidation of **2T** with $(4\text{-}BrC_6H_4)_3N^{+\bullet}BF_4^-$:

To a solution of **2T** (20.5 mg, 21.4 μmol) in dry CH_2Cl_2 (1 mL) was added $(4\text{-}BrC_6H_4)_3N^{+\bullet}BF_4^-$ (24.3 mg, 42.8 μmol) at 20 °C, and the mixture was stirred for 2 h. The addition of dry hexane led to precipitation of the dication salt. The supernatant solution was removed by decantation, and the precipitates were washed with dry hexane three times, and dried in vacuo to give $\mathbf{2}^{2+}(BF_4^-)_2$ (24.3 mg) as a red powder in 100% yield.

$\mathbf{2}^{2+}(BF_4^-)_2$; Mp: 299.1 °C~306.5 °C (decomp.); ^{1}H NMR (CD_3CN): δ /ppm 9.48 (4H, s), 8.96 (4H, d, J= 1.6 Hz), 8.27 (4H, d, J= 9.6 Hz), 8.23 (4H, dd, J= 1.6 Hz, 9.6 Hz), 7.70 (4H, dd, J= 1.2 Hz, 8.8 Hz), 7.46 (4H, ddd, J= 0.8 Hz, 6.4 Hz, 8.8 Hz),7.38 (4H, ddd, J= 1.2 Hz, 6.4 Hz, 8.8 Hz), 7.18 (4H, dd, J= 1.2 Hz, 8.8 Hz), 1.59 (36H, s); ^{13}C NMR (CD_3CN): δ/ppm 179.29, 167.40, 147.60, 145.75, 139.14, 138.52, 136.51, 136.09, 133.30, 133.20, 131.53, 131.30, 128.65, 127.83, 127.61, 127.17, 37.31, 30.26; IR (ATR): ν/cm^{-1} 3072, 2963, 2868, 1790, 1761, 1606, 1507, 1478, 1443, 1414, 1379, 1364, 1346, 1321, 1274, 1262, 1211, 1167, 1152, 1119, 1086, 1049, 1037, 1024, 957, 913, 878, 825, 780, 774, 760, 694, 685, 676, 660, 616, 593, 518, 440; LR-MS (FD) m/z (%): 959.60 (10), 958.60 (40), 957.59 (87), 956.59 (M^+, bp); HR-MS (FD) Calcd. for $C_{74}H_{68}$: 956.53210; Found: 956.53289.

5,5'-([9,9'-Bianthracene]-10,10'-diyl)-bis(2,8-di-*tert*-butyl-5*H*-dibenzo[*a,d*]cycloheptatrien-5-ylium) bis(hexafluorophosphate) $2^{2+}(PF_6^-)_2$

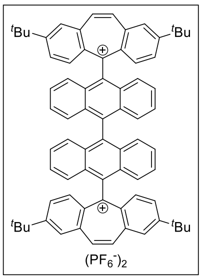

To a solution of diol **2-OH** (21.8 mg, 22.0 μmol) in trifluoroacetic anhydride (TFAA, 2.0 mL) was added 60% HPF_6 aq. (30 μL, 220 μmol) at 0 °C to give a deep red solution, and the mixture was stirred at 25 °C for 2 h. The addition of dry Et_2O led to precipitation of the dication salt. The supernatant solution was removed by decantation, and the precipitates were washed with dry Et_2O three times, and dried in vacuo to give $\mathbf{2}^{2+}(PF_6^-)_2$ (22.1 mg) as a red powder in 81% yield.

$\mathbf{2}^{2+}(PF_6^-)_2$; Mp: 223.6 °C~242.9 °C (decomp.); ^{1}H NMR and ^{13}C NMR spectra are identical to those of $\mathbf{2}^{2+}(BF_4^-)_2$; IR (ATR): ν/cm^{-1} 3066, 2964, 2871, 1790, 1757, 1605, 1507, 1479, 1443, 1411, 1381, 1363, 1346, 1320, 1274, 1212, 1169, 1118, 1026, 957, 916, 877, 838, 831, 775, 762, 694, 675, 658, 615, 593, 556, 517, 495, 440; LR-MS (FD) m/z (%): 1105.58 (5), 1104.57 (11), 1103.57 (40), 1102.57 (88), 1101.57 ($M^{2+}PF_6^-$, bp), 973.59 (6), 972.58 (15), 971.58 (18), 959.59 (7), 958.60 (19), 957.59 (41), 956.59 (M^+, 47), 945.59 (5), 944.59 (12), 943.59 (13), 480.30 (6), 479.80 (12), 479.30 (28), 478.80 (51), 478.30 (M^{2+}, 55); HR-MS (FD) Calcd. for $C_{74}H_{68}$: 956.53210; Found: 956.53501.

5,5'-([9,9'-Bianthracene]-10,10'-diyl)-bis(2,8-di-*tert*-butyl-5*H*-dibenzo[*a,d*]cycloheptatrien-5-ylium) bis(hexachloroantimonate) $\mathbf{2}^{2+}(SbCl_6^-)_2$

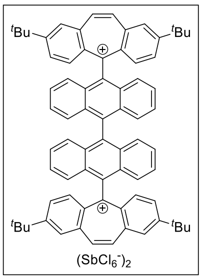

To a solution of **2F** (17.9 mg, 18.7 μmol) in dry CH_2Cl_2 (2 mL) was added $(2,4\text{-}Br_2C_6H_3)_3N^{+\bullet}SbCl_6^-$ (39.4 mg, 37.4 μmol) at 20 °C, and the mixture was stirred for 2 h. The addition of dry hexane led to precipitation of the dication salt. The supernatant solution was removed by decantation, and the precipitates were washed with dry hexane three times, and dried in vacuo to give $\mathbf{2}^{2+}(SbCl_6^-)_2$ (30.3 mg) as a red powder in 100% yield.

$\mathbf{2}^{2+}(SbCl_6^-)_2$; Mp: 215.7 °C~219.1 °C (decomp.); ^{1}H NMR and ^{13}C NMR spectra are identical to those of $\mathbf{2}^{2+}(BF_4^-)_2$; IR (ATR): ν/cm^{-1} 3077, 3064, 2955, 2926, 2868, 2854, 1602, 1505, 1478, 1464, 1443, 1409, 1378, 1362, 1344, 1319, 1272, 1261, 1223, 1212, 1169, 1118, 1024, 957, 910, 893, 876, 822, 778, 773, 757, 694, 675, 657, 615, 588, 553, 518, 438, 426; LR-MS (FD) m/z (%): 972.46 (5), 960.47 (6), 959.47 (15), 958.48 (42), 957.48 (83), 956.47 (M^+, bp), 931.46 (6), 930.46 (7), 478.73 (6), 478.23 (M^{2+}, 7), 41.02 (5); HR-MS (FD) Calcd. for $C_{74}H_{68}$: 956.53210; Found: 956.53416.

5,5'-([9,9':10',9''-Teranthracene]-10,10''-diyl)-bis(2,8-di-*tert*-butyl-5*H*-dibenzo[*a,d*]cycloheptatrien-5-ylium) bis(tetrafluoroborate) $\mathbf{3}^{2+}(BF_4^-)_2$

Dehydration of **3-OH** with HBF_4:

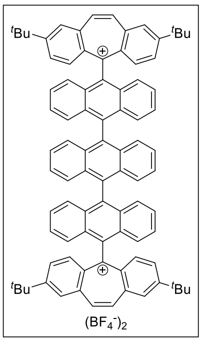

To a solution of diol **3-OH** (49.7 mg, 42.6 μmol) in trifluoroacetic anhydride (TFAA, 2 mL) was added 42% HBF_4 aq. (64 μL, 0.426 mmol) at 0 °C to give a deep red solution, and the mixture was stirred at 26 °C for 2 h. The addition of dry Et_2O led to precipitation of the dication salt. The supernatant solution was removed by decantation, and the precipitates were washed with dry Et_2O three times, and dried in vacuo to give $\mathbf{3}^{2+}(BF_4^-)_2$ (55.2 mg) as a red powder in 99% yield.

Oxidation of **3T** with $(4\text{-BrC}_6H_4)_3N^{+\bullet}BF_4^-$:

To a solution of **3T** (14.0 mg, 12.4 μmol) in dry CH_2Cl_2 (1 mL) was added $(4\text{-BrC}_6H_4)_3N^{+\bullet}BF_4^-$ (14.0 mg, 24.7 μmol) at 19 °C, and the mixture was stirred for 2 h. The addition of dry hexane led to precipitation of the dication salt. The supernatant solution was removed by decantation, and the precipitates were washed with dry hexane three times, and dried in vacuo to give **3**$^{2+}(BF_4^-)_2$ (15.5 mg) as a red powder in 96% yield.

3$^{2+}(BF_4^-)_2$; Mp: 310.5 °C~336.4 °C (decomp.); ^{1}H NMR (CD_3CN): δ /ppm 9.47 (4H, s), 8.96 (4H, d, J= 1.6 Hz), 8.28 (4H, d, J= 9.2 Hz), 8.24 (4H, dd, J= 1.6 Hz, 9.2 Hz), 7.69 (4H, dd, J= 3.6 Hz, 7.2 Hz), 7.67 (4H, dd, J= 1.2 Hz, 8.4 Hz), 7.51 (4H, dd, J= 3.6 Hz, 7.2 Hz), 7.44 (4H, ddd, J= 1.2 Hz, 6.4 Hz, 8.4 Hz),7.38 (4H, ddd, J= 1.2 Hz, 6.4 Hz, 8.4 Hz), 7.18 (4H, dd, J= 1.2 Hz, 8.4 Hz), 1.59 (36H, s); ^{13}C NMR (CD_3CN): δ/ppm 179.56, 167.41, 147.59, 145.72, 139.17, 138.59, 137.66, 135.73, 134.23, 133.32, 133.19, 132.11, 131.70, 131.37, 128.62, 127.94, 127.80, 127.44, 127.37, 127.13, 37.32, 30.28; IR (ATR): ν/cm^{-1} 3060, 2966, 2907, 2870, 1759, 1604, 1506, 1479, 1441, 1412, 1379, 1363, 1319, 1275, 1197, 1148, 1117, 1056, 1025, 957, 914, 889, 850, 825, 778, 757, 694, 675, 654, 617, 599, 592, 520, 441, 420; LR-MS (FD) m/z (%): 1245.65 (8), 1220.67 (9), 1219.66($M^{2+}BF_4^-$, 9), 1203.68 (6), 1202.66 (13), 1201.66 (11), 1149.65 (8), 1148.65 (13), 1147.65 (17), 1136.66 (6), 1135.66 (22), 1134.67 (51), 1133.66 (bp), 1132.67 (M^+, 93), 1120.66 (9), 1119.66 (10), 780.51 (5), 711.44 (6), 670.37 (6), 656.39 (25), 655.38 (37), 567.83 (13), 567.33 (22), 566.84 (34), 566.33 (30); HR-MS (FD) Calcd. for $C_{88}H_{76}$: 1132.59470; Found: 1132.59360.

5,5'-([9,9':10',9'':10'',9'''-Quateranthracene]-10,10'''-diyl)-bis(2,8-di-*tert*-butyl-5*H*-dibenzo[*a,d*]cycloheptatrien-5-ylium) bis(tetrafluoroborate) 4$^{2+}(BF_4^-)_2$

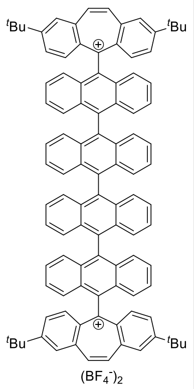

Dehydration of **4-OH** with HBF_4:

To a solution of diol **4-OH** (43.4 mg, 32.3 μmol) in trifluoroacetic anhydride (TFAA, 2 mL) was added 42% HBF_4 aq. (50 μL, 0.323 mmol) at 0 °C to give a deep red solution, and the mixture was stirred at 27 °C for 2 h. The addition of dry Et_2O led to precipitation of the dication salt. The supernatant solution was removed by decantation, and the precipitates were washed with dry Et_2O three times, and dried in vacuo to give **4**$^{2+}(BF_4^-)_2$ (48.0 mg) as a red powder in 100% yield.

Oxidation of **4T** with $(4\text{-BrC}_6H_4)_3N^{+\bullet}BF_4^-$:

To a solution of **4T** (11.6 mg, 8.86 μmol) in dry CH_2Cl_2 (1 mL) was added $(4\text{-BrC}_6H_4)_3N^{+\bullet}BF_4^-$ (10.0 mg, 17.7 μmol) at 20 °C, and the mixture was stirred for 2 h. The addition of dry hexane led to precipitation of the dication salt. The supernatant solution was removed by decantation, and the precipitates were washed with dry hexane three times, and dried in vacuo to give **4**$^{2+}(BF_4^-)_2$ (12.5 mg) as a red powder in 95% yield.

$4^{2+}(BF_4^-)_2$; Mp: 292.9 °C~306.0 °C (decomp.); ^{1}H NMR (CD_3CN): δ /ppm 9.48 (4H, s), 8.96 (4H, d, J= 2.0 Hz), 8.29 (4H, d, J= 9.2 Hz), 8.25 (4H, dd, J= 2.0 Hz, 9.2 Hz), 7.72-7.65 (12H, m), 7.53-7.48 (8H, m), 7.47 (4H, ddd, J= 1.2 Hz, 6.4 Hz, 8.4 Hz), 7.39 (4H, ddd, J= 1.2 Hz, 6.4 Hz, 8.4 Hz), 7.19 (4H, dd, J= 1.2 Hz, 8.4 Hz), 1.59 (36H, s); ^{13}C NMR (CD_3CN): δ/ppm 179.62, 167.40, 147.59, 145.71, 139.18, 138.60, 137.82, 135.68, 135.34, 133.79, 133.33, 133.19, 132.28, 132.15, 131.73, 131.38, 128.61, 127.97, 127.92, 127.76, 127.44, 127.33, 127.20, 127.13, 37.32, 30.28; IR (ATR): ν/cm^{-1} 3060, 2963, 2907, 2872, 1792, 1756, 1603, 1506, 1479, 1441, 1410, 1379, 1346, 1318, 1275, 1204, 1159, 1150, 1117, 1024, 956, 914, 896, 852, 826, 777, 756, 694, 675, 653, 616, 610, 599, 593, 519, 440, 420; LR-MS (FD) m/z (%): 1424.70 (8), 1423.71 (12), 1422.72 (18), 1421.71 (15), 1397.73 (6), 1396.72 (10), 1395.74 ($M^{2+}BF_4^-$, 11), 1380.71 (8), 1379.71 (15), 1378.73 (27), 1377.72 (23), 1324.74 (6), 1312.69 (9), 1311.71 (28), 1310.72 (64), 1309.72 (bp), 1308.72 (M^+, 83), 1296.71 (5), 1295.70 (5), 1133.67 (5), 944.44 (9), 943.42 (10), 847.43 (9), 846.44 (8), 833.47 (6), 832.45 (15), 831.45 (22), 655.87 (18), 655.38 (23), 654.87 (32), 654.37 (M^{2+}, 26), 479.31 (5); HR-MS (FD) Calcd. for $C_{102}H_{84}$: 1308.65730; Found: 1308.65617.

5,5'-([9,9':10',9'':10'',9''':10''',9''''-Quinqueanthracene]-10,10''''-diyl)-bis(2,8-di-*tert*-butyl-5*H*-dibenzo[*a,d*]cycloheptatrien-5-ylium) bis(tetrafluoroborate) $5^{2+}(BF_4^-)_2$

<u>Dehydration of **5-OH** with HBF_4:</u>

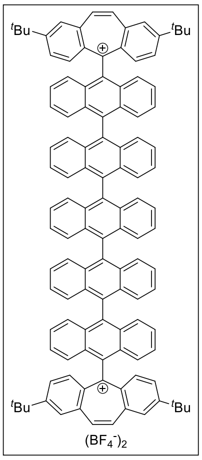

To a solution of diol **5-OH** (56.6 mg, 37.2 μmol) in trifluoroacetic anhydride (TFAA, 2 mL) was added 42% HBF_4 aq. (56 μL, 0.362 mmol) at 0 °C to give a deep red solution, and the mixture was stirred at 25 °C for 2 h. The addition of dry Et_2O led to precipitation of the dication salt. The supernatant solution was removed by decantation, and the precipitates were washed with dry Et_2O three times, and dried in vacuo to give $5^{2+}(BF_4^-)_2$ (62.0 mg) as a red powder in 100% yield.

<u>Oxidation of **5T** with $(4\text{-}BrC_6H_4)_3N^{+\bullet}BF_4^-$:</u>

To a solution of **5T** (11.4 mg, 7.67 μmol) in dry CH_2Cl_2 (1 mL) was added $(4\text{-}BrC_6H_4)_3N^{+\bullet}BF_4^-$ (8.7 mg, 15.3 μmol) at 19 °C, and the mixture was stirred for 2 h. The addition of dry hexane led to precipitation of the dication salt. The supernatant solution was removed by decantation, and the precipitates were washed with dry hexane three times, and dried in vacuo to give $5^{2+}(BF_4^-)_2$ (12.6 mg) as a red powder in 99% yield.

$5^{2+}(BF_4^-)_2$; Mp: 285.9 °C~288.7 °C (decomp.); ^{1}H NMR (CD_3CN): δ /ppm 9.48 (4H, s), 8.97 (4H, d, J= 1.6 Hz), 8.30 (4H, d, J= 9.6 Hz), 8.26 (4H, dd, J= 1.6 Hz, 9.6 Hz), 7.73-7.68 (16H, m), 7.53-7.48 (12H, m), 7.47 (4H, ddd, J= 1.2 Hz, 6.4 Hz, 8.4 Hz), 7.40 (4H, ddd, J= 1.2 Hz, 6.4 Hz, 8.4 Hz), 7.19 (4H, dd, J= 1.2 Hz, 8.4 Hz), 1.60 (36H, s); ^{13}C NMR (CD_3CN): δ/ppm 179.64,

167.41, 147.58, 145.70, 139.19, 138.60, 137.85, 135.67, 135.49, 134.89, 133.73, 133.33, 133.18, 132.33, 132.31, 132.17, 131.74, 131.38, 128.62, 127.98, 127.95, 127.88, 127.76, 127.45, 127.33, 127.20, 127.15, 127.13, 37.32, 30.28; IR (ATR): ν/cm^{-1} 3197, 3060, 2965, 2870, 1761, 1603, 1505, 1479, 1440, 1411, 1379, 1363, 1316, 1275, 1199, 1149, 1117, 1024, 956, 913, 900, 851, 826, 756, 694, 675, 652, 615, 599, 592, 519, 431, 416; LR-MS (ESI) m/z (%): 1486.72 (1), 1485.72 (1), 1484.72 (M$^+$, 1), 744.36 (8), 743.86 (27), 743.36 (61), 742.86 (bp), 742.36 (M^{2+}, 83), 655.34 (5), 453.17 (10), 415.21 (5), 316.21 (7); HR-MS (ESI) Calcd. for $C_{116}H_{92}$: 742.35940 (M^{2+}); Found: 742.35889.

5,5'-([9,9':10',9'':10'',9''':10''',9'''':10'''',9'''''-Sexianthracene]-10,10'''''-diyl)-bis(2,8-di-*tert*-butyl-5*H*-dibenzo[*a,d*]cycloheptatrien-5-ylium) bis(tetrafluoroborate) 6^{2+}(BF$_4^-$)$_2$

<u>Dehydration of **6-OH** with HBF$_4$:</u>

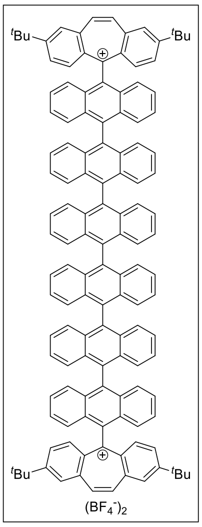

To a solution of diol **6-OH** (60.7 mg, 35.8 μmol) in trifluoroacetic anhydride (TFAA, 2 mL) was added 42% HBF_4 aq. (54 μL, 0.358 mmol) at 0 °C to give a deep red solution, and the mixture was stirred at 25 °C for 2 h. The addition of dry Et_2O led to precipitation of the dication salt. The supernatant solution was removed by decantation, and the precipitates were washed with dry Et_2O three times, and dried in vacuo to give **6**$^{2+}$(BF$_4^-$)$_2$ (65.8 mg) as a red powder in 100% yield.

<u>Oxidation of **6T** with (4-BrC$_6$H$_4$)$_3$N$^{+\bullet}$BF$_4^-$:</u>

To a solution of **6T** (13.1 mg, 7.88 μmol) in dry CH_2Cl_2 (1 mL) was added (4-BrC$_6$H$_4$)$_3$N$^{+\bullet}$BF$_4^-$ (9.0 mg, 15.8 μmol) at 20 °C, and the mixture was stirred for 2 h. The addition of dry hexane led to precipitation of the dication salt. The supernatant solution was removed by decantation, and the precipitates were washed with dry hexane three times, and dried in vacuo to give **6**$^{2+}$(BF$_4^-$)$_2$ (14.0 mg) as a red powder in 97% yield.

6^{2+}(BF$_4^-$)$_2$; Mp: 287.9 °C~296.8 °C (decomp.); ^{1}H NMR (CD$_3$CN): δ /ppm 9.48 (4H, s), 8.96 (4H, d, J= 2.0 Hz), 8.30 (4H, d, J= 9.26 Hz), 8.26 (4H, dd, J= 2.0 Hz, 9.6 Hz), 7.74-7.68 (20H, m), 7.54-7.50 (16H, m), 7.48 (4H, ddd, J= 0.8 Hz, 6.4 Hz, 8.8 Hz), 7.40 (4H, ddd, J= 0.8 Hz, 6.4 Hz, 8.4 Hz), 7.20 (4H, dd, J= 0.8 Hz, 8.4 Hz), 1.60 (36H, s); ^{13}C NMR (CD$_3$CN): δ/ppm 179.61, 167.43, 147.58, 145.67, 139.22, 138.63, 137.89, 137.43, 135.54, 135.05, 134.85, 133.73, 133.36, 133.20, 132.38, 132.36, 132.33, 132.19, 131.76, 131.38, 128.63, 128.01, 127.98, 127.91, 127.89, 127.76, 127.46, 127.34, 127.21, 127.16 (3C), 37.34, 30.29; IR (ATR): ν/cm^{-1} 3206, 3060, 2968, 2909, 2872, 1784, 1761, 1603, 1506, 1479, 1439, 1412, 1378, 1363, 1316, 1275, 1206, 1156, 1117, 1024, 956, 913, 903, 851, 823, 755, 700, 675, 651, 615, 599, 519, 440, 431, 419; LR-MS (ESI) m/z (%): 1662.79 (2), 1661.78 (3), 1660.78 (M$^+$, 2), 1023.45 (8), 832.40 (12), 831.89 (33),

831.39 (70), 830.89 (bp), 830.39 (M^{2+}, 66), 671.33 (11), 479.28 (5); HR-MS (ESI) Calcd. for $C_{130}H_{100}$: 830.39070 (M^{2+}); Found: 830.39020.

5,5'-([9,9':10',9'':10'',9''':10''',9'''':10'''',9'''''-Sexianthracene]-10,10'''''-diyl)-bis(2,8-di-*tert*-butyl-5*H*-dibenzo[*a*,*d*]cycloheptatrien-5-ylium) bis(trifluoromethanesulfonyl)imide $6^{2+}(NTf_2^-)_2$

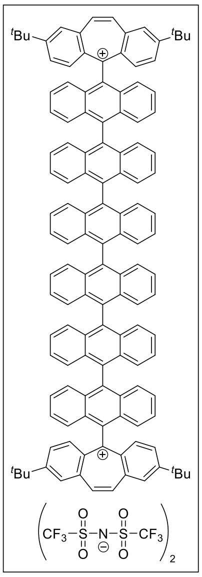

To a solution of diol **6-OH** (33.7 mg, 19.9 μmol) in CH_2Cl_2 (2 mL) was added bis(trifluoromethanesulfonyl)imide ($HNTf_2$) (36.0 mg, 0.128 mmol) at 0 °C to give a deep red solution, and the mixture was stirred at 25 °C for 2 h. The addition of dry Et_2O led to precipitation of the dication salt. The supernatant solution was removed by decantation, and the precipitates were washed with dry Et_2O three times, and dried in vacuo to give $\mathbf{6}^{2+}(NTf_2^-)_2$ (39.4 mg) as a red powder in 89% yield.

$\mathbf{6}^{2+}(NTf_2^-)_2$; Mp: 244.5 °C~254.3 °C (decomp.); ^{1}H NMR and ^{13}C NMR spectra are identical to those of $\mathbf{6}^{2+}(BF_4^-)_2$; IR (ATR): ν/cm^{-1} 3060, 2967, 2908, 2870, 1799, 1706, 1603, 1506, 1479, 1439, 1412, 1379, 1345, 1316, 1275, 1224, 1181, 1132, 1117, 1054, 1024, 957, 914, 903, 825, 789, 779, 756, 738, 674, 651, 613, 598, 569, 507, 431, 419; LR-MS (FD) m/z (%): 1945.84 (5), 1944.88 (17), 1943.88 (38), 1942.87 (59), 1941.87 (73), 1940.87 ($M^{2+}NTf_2^-$, 42), 1677.91 (6), 1676.91 (5), 1675.90 (5), 1664.89 (5), 1663.96 (8), 1662.93 (11), 1661.95 (12), 1660.96 (M^+, 5), 1649.93 (5), 1647.92 (6), 1108.66 (5), 1057.55 (8), 1056.55 (19), 1055.54 (23), 1054.52 (20), 1053.52 (19), 1039.54 (5), 1038.54 (7), 1037.53 (6), 1026.45 (7), 1025.54 (14), 1024.55 (44), 1023.55 (88), 1022.54 (bp), 832.45 (20), 831.96 (28), 831.47 (48), 830.97 (62), 830.48 (M^{2+}, 40), 671.39 (9), 670.38 (14); HR-MS (FD) Calcd. for $C_{130}H_{100}$: 1660.78250; Found: 1660.78505.

9,10-Bis(2,8-di-*tert*-butyl-5*H*-dibenzo[*a*,*d*]cycloheptatrien-5-ylidene)-9,10-dihydroanthracene $1F_{anti,anti}$

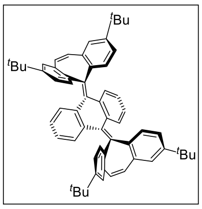

To a solution of $\mathbf{1}^{2+}(BF_4^-)_2$ (79.0 mg, 82.7 μmol) in dry CH_3CN (5 mL) was added activated zinc powder (108 mg, 1.65 mmol) at 22 °C. The mixture was stirred at 22 °C for 5 min under sonication, and then diluted with water. The whole mixture was extracted with CH_2Cl_2 three times. The combined organic layers were washed with water and brine, and dried over anhydrous Na_2SO_4. After filtration through silica gel, the solvent was concentrated under reduced pressure to give ca. 2:1 mixture of $\mathbf{1F}_{anti,anti}$ and $\mathbf{1F}_{syn,anti}$ as a white solid. Then, a solution of the whole mixture in dimethylsulfoxide (DMSO, 8 mL) was refluxed for 1.5 h. After cooling to 22 °C, the resulting solution was diluted with water. The whole mixture was extracted with CH_2Cl_2 three times. The

combined organic layers were washed with water and brine, and dried over anhydrous Na_2SO_4. After filtration through silica gel, the solvent was concentrated under reduced pressure to give **1F***anti,anti* (64.5 mg) as a white solid in 100% yield.

1F*anti,anti*; Mp: 310.3-317.6 °C (decomp.); ^{1}H NMR ($CDCl_3$): δ /ppm 7.42 (4H, d, J= 1.6 Hz), 7.24 (4H, dd, J= 1.6 Hz, 8.0 Hz), 7.16 (4H, s), 7.05 (4H, d, J= 8.0 Hz), 6.57 (4H, dd, J= 3.2 Hz, 5.6 Hz), 6.42 (4H, dd, J= 3.2 Hz, 5.6 Hz), 1.34 (36H, s); ^{13}C NMR ($CDCl_3$): δ/ppm 149.66, 137.72, 136.83, 136.78, 135.11, 134.51, 131.78, 128.96, 127.96, 125.77, 124.85, 124.53, 34.52, 31.43; IR (ATR): ν/cm^{-1} 3065, 3014, 2958, 2928, 2902, 2866, 1601, 1495, 1462, 1386, 1361, 1282, 1265, 1251, 1204, 1193, 1173, 1163, 1125, 1112, 1042, 1026, 948, 906, 888, 846, 824, 818, 793, 785, 762, 749, 734, 723, 714, 672, 651, 601, 557, 545, 498, 460, 457, 423, 419; LR-MS (FD) m/z (%): 783.55 (5), 782.55 (25), 781.54 (68), 780.54 (M^+, bp); HR-MS (FD) Calcd. for $C_{60}H_{60}$: 780.46950; Found: 780.46835; Fluorescence (CH_2Cl_2, λ_{ex} = 290 nm): λ_{em}/nm (Φ_F) 429 (0.21).

9,10-Bis(2,8-di-*tert*-butyl-5*H*-dibenzo[*a,d*]cycloheptatrien-5-ylidene)-9,10-dihydroanthracene 1F*syn,anti*

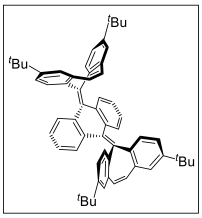

A solution of **1F***anti,anti* (39.1 mg, 50.1 μmol, 2.5×10^{-3} mol L^{-1}) in CH_2Cl_2 (20 mL) was degassed by Ar bubbling, and then photoirradiated with an Ushiospax SX-UID501XAMQ light source device and a CORNING COLOR FILTER (No. O-51) at 24 °C for 5 h (λ> 360 nm). The solvent was concentrated under reduced pressure to give **1F***syn,anti* (38.4 mg) as a white solid in 98% yield.

1F*syn,anti*; Mp: 289.0 °C-298.6 °C (decomp.); ^{1}H NMR ($CDCl_3$): δ /ppm 7.74 (2H, d, J= 8.0 Hz), 7.49 (2H, dd, J= 1.2 Hz, 7.6 Hz), 7.43 (2H, dd, J= 2.0 Hz, 8.0 Hz), 7.30 (2H, d, J= 2.0 Hz), 7.26 (2H, d, J= 2.0 Hz), 7.05 (2H, s), 6.93 (2H, dd, J= 2.0 Hz, 8.0 Hz), 6.91 (2H, s), 6.77 (2H, ddd, J= 1.2 Hz, 7.6 Hz, 7.6 Hz), 6.63 (2H, d, J= 8.0 Hz), 6.55 (2H, ddd, J= 1.2 Hz, 7.6 Hz, 7.6 Hz), 6.28 (2H, dd, J= 1.2 Hz, 7.6 Hz), 1.32 (18H, s), 1.26 (18H, s); ^{13}C NMR ($CDCl_3$): δ/ppm 149.34, 148.93, 138.26, 137.49, 137.36, 136.65, 136.59, 136.26, 135.75, 135.01, 135.01, 133.25, 131.62, 131.14, 129.40, 129.09, 127.38, 127.34, 125.04, 124.62, 124.54, 124.51, 123.95, 123.84, 34.47, 34.36, 31.42, 31.34; IR (ATR): ν/cm^{-1} 3062, 3016, 2958, 2902, 2865, 1601, 1540, 1490, 1478, 1458, 1384, 1361, 1266, 1252, 1203, 1168, 1122, 1025, 944, 910, 904, 888, 848, 827, 818, 795, 781, 763, 726, 714, 706, 693, 675, 664, 653, 632, 599, 559, 500, 452, 446, 429; LR-MS (FD) m/z (%): 783.56 (7), 782.56 (25), 781.56 (69),780.56 (M^+, bp); HR-MS (FD) Calcd. for $C_{60}H_{60}$: 780.46950; Found: 780.47042; Fluorescence (CH_2Cl_2, λ_{ex} = 290 nm): λ_{em}/nm (Φ_F) 443 (0.17).

5,5'-([9,9'-Bianthracene]-10,10'-diyl)-bis(2,8-di-*tert*-butyl-5*H*-dibenzo[*a,d*]cycloheptatrien-5-yl) 2T

To a solution of **2**$^{2+}$(BF_4^-)$_2$ (64.0 mg, 56.6 μmol) in dry CH_3CN (6 mL) was added activated

zinc powder (74.2 mg, 1.13 mmol) at 26 °C. The mixture was stirred at 26 °C for 5 min under sonication, and then diluted with water. The whole mixture was extracted with CH_2Cl_2 three times. The combined organic layers were washed with water and brine, and dried over anhydrous Na_2SO_4. After filtration, the solvent was concentrated under reduced pressure to give **2T** (54.2 g) as a deep green solid in 100% yield.

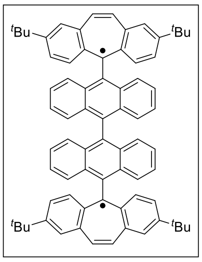

2T; Mp: 240.7 °C-274.1 °C (decomp.); IR (ATR): ν/cm^{-1} 3059, 3027, 2956, 2901, 2864, 1950, 1914, 1772, 1714, 1582, 1527, 1517, 1476, 1454, 1438, 1383, 1359, 1301, 1279, 1265, 1247, 1200, 1162, 1146, 1126, 1117, 1025, 954, 912, 894, 877, 854, 832, 824, 794, 767, 760, 734, 703, 685, 643, 608, 582, 436, 409; LR-MS (FD) m/z (%): 972.57 (5), 959.58 (12), 958.57 (37), 957.57 (84), 956.57 (M^+, bp); HR-MS (FD) Calcd. for $C_{74}H_{68}$: 956.53210; Found: 956.53321.

10,10'-Bis(2,8-di-*tert*-butyl-5*H*-dibenzo[a,d]cycloheptatrien-5-ylidene)-10*H*,10'*H*-9,9'-bianthracenylidene 2F

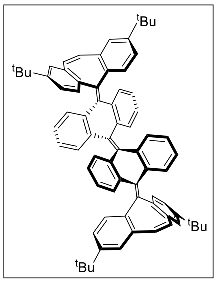

A solution of **2T** (19.0 mg, 19.8 μmol) in toluene (5 mL) was degassed by Ar bubbling, and then heated at 100 °C for 20 min. After cooling to 25 °C, the resulting solution was concentrated under reduced pressure. The resulting residue was washed with hexane three times, and dried in vacuo to give **2F** (15.0 mg) as a white solid in 79% yield.

2F; Mp: 333.5-350.5 °C (decomp.); ^{1}H NMR ($CDCl_3$): δ /ppm 7.45 (4H, d, J= 1.6 Hz), 7.25 (4H, dd, J= 1.6 Hz, 8.0 Hz), 7.21 (4H, dd, J= 1.2 Hz, 7.6 Hz), 7.20 (4H, s), 7.12 (4H, d, J= 8.0 Hz), 6.89 (4H, ddd, J= 1.2Hz, 7.6 Hz, 7.6 Hz), 6.75 (4H, dd, J= 1.2 Hz, 7.6 Hz, 7.6 Hz), 6.55 (4H, dd, J= 1.2 Hz, 7.6 Hz), 1.35 (36H, s); ^{13}C NMR ($CDCl_3$): δ/ppm 149.85, 138.30, 138.01, 137.18, 136.62, 135.06, 134.53, 132.09, 131.78, 129.06, 128.64, 128.30, 125.90, 125.25, 124.84, 124.81, 34.54, 31.43; IR (ATR): ν/cm^{-1} 3060, 3022, 2954, 2930, 2903, 2866, 1598, 1496, 1458, 1449, 1394, 1384, 1361, 1271, 1252, 1202, 1171, 1154, 1126, 1113, 1038, 1024, 948, 934, 910, 892, 835, 815, 799, 783, 758, 752, 729, 694, 678, 667, 652, 636, 583, 500, 453, 420; LR-MS (FD) m/z (%): 959.48 (9), 958.48 (36), 957.48 (83), 956.47 (M^+, bp); HR-MS (FD) Calcd. for $C_{74}H_{68}$: 956.53210; Found: 956.53410; Fluorescence (CH_2Cl_2, λ_{ex} = 290 nm): λ_{em}/nm (Φ_F) 450 (0.41).

5,5'-([9,9':10',9''-Teranthracene]-10,10''-diyl)-bis(2,8-di-*tert*-butyl-5*H*-dibenzo[*a,d*]cycloheptatrien-5-yl) 3T

To a solution of $3^{2+}(BF_4^-)_2$ (31.0 mg, 23.7 μmol) in dry CH_3CN (3 mL) was added activated zinc powder (31.0 mg, 0.474 mmol) at 23 °C. The mixture was stirred at 23 °C for 5 min under sonication, and then diluted with water. The whole mixture was extracted with CH_2Cl_2 three times. The combined organic layers were washed with water and brine, and dried over anhydrous

Na_2SO_4. After filtration, the solvent was concentrated under reduced pressure to give **3T** (26.9 mg) as a light brown solid in 100% yield.

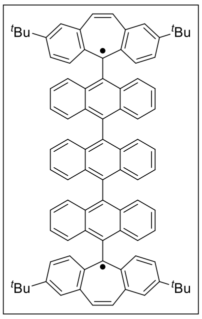

3T; Mp: 303.8 °C~327.6 °C (decomp.); IR (ATR): ν/cm^{-1} 3059, 2961, 2926, 2905, 2867, 1723, 1675, 1583, 1517, 1477, 1456, 1438, 1380, 1362, 1281, 1250, 1201, 1159, 1144, 1119, 1022, 911, 897, 890, 832, 817, 792, 759, 734, 687, 656, 634, 607, 599, 414; LR-MS (FD) m/z (%): 1182.73 (5), 1181.73 (6), 1180.73 (6), 1179.75 (6), 1178.76 (7), 1177.78 (5), 1168.73 (6), 1167.74 (9), 1166.73 (16), 1165.73 (23), 1164.73 (29), 1163.72 (27), 1162.71 (26), 1153.73 (8), 1152.74 (13), 1151.74 (24), 1150.73 (43), 1149.73 (63), 1148.73 (79), 1147.73 (57), 1139.72 (6), 1138.73 (13), 1137.73 (27), 1136.73 (51), 1135.73 (77), 1134.73 (bp), 1133.74 (66), 1132.74 (M^+, 50), 1124.72 (6), 1123.73 (12), 1122.73 (25), 1121.73 (52), 1120.73 (79), 1119.73 (56), 1109.71 (6), 1108.72 (15), 1107.72 (28), 1106.71 (29), 780.57 (6), 670.41 (6), 656.43 (8), 655.42 (9), 642.41 (7), 567.37 (6); HR-MS (FD) Calcd. for $C_{88}H_{76}$: 1132.59470; Found: 1132.59640.

5,5'-([9,9':10',9'':10'',9'''-Quateranthracene]-10,10'''-diyl)-bis(2,8-di-*tert*-butyl-5*H*-dibenzo[*a,d*]cycloheptatrien-5-yl) 4T

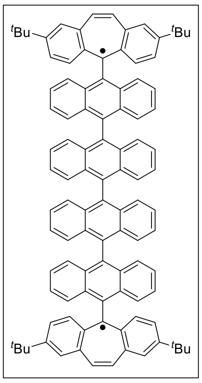

To a solution of **4**$^{2+}$(BF_4^-)$_2$ (24.9 g, 16.8 μmol) in dry CH_3CN (4 mL) was added cobaltocene (6.4 mg, 33.8 μmol) at 25 °C to give a yellow suspension, and the mixture was stirred at 25 °C for 5 min under sonication. After the supernatant solution was removed by decantation, the precipitates were washed with dry CH_3CN three times and dried in vacuo to give **4T** (19.7 mg) as a brown powder in 90% yield.

4T; Mp: >400 °C; IR (ATR): ν/cm^{-1} 3057, 2961, 2903, 2865, 1559, 1517, 1473, 1457, 1438, 1380, 1362, 1303, 1249, 1201, 1158, 1144, 1118, 1023, 954, 911, 896, 831, 817, 791, 757, 733, 670, 648, 606, 599, 442, 417; LR-MS (MALDI) m/z (%): 1341.64 (7), 1340.64 (5), 1327.66 (11), 1326.65 (22), 1325.65 (35), 1324.64 (41), 1323.64 (35), 1312.66 (9), 1311.66 (24), 1310.66 (58), 1309.65 (bp), 1308.65 (M+, 91), 1298.65 (13), 1297.65 (38), 1296.64 (72), 1295.64 (65), 877.04 (5), 862.07 (5), 861.07 (11), 845.09 (9), 672.05 (11), 657.08 (12), 656.07 (42), 650.05 (8); HR-MS (MALDI) Calcd. for $C_{102}H_{84}$: 1308.65675; Found: 1308.65967.

5,5'-([9,9':10',9'':10'',9''':10''',9''''-Quinqueanthracene]-10,10''''-diyl)-bis(2,8-di-*tert*-butyl-5*H*-dibenzo[*a,d*]cycloheptatrien-5-yl) 5T

To a solution of **5**$^{2+}$(BF_4^-)$_2$ (15.4 mg, 9.28 μmol) in dry THF (1 mL) was added cobaltocene (3.6 mg, 19.0 μmol) at 25 °C to give a yellow suspension, and the mixture was stirred at 25 °C for 5 min under sonication. After the supernatant solution was removed by decantation, the

precipitates were washed with dry CH_3CN three times and dried in vacuo to give **5T** (12.2 mg) as a deep brown powder in 88% yield.

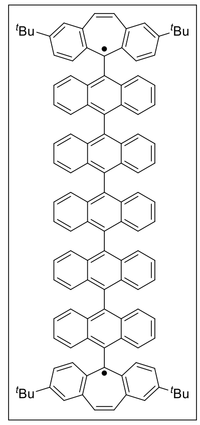

5T; Mp: >400 °C; IR (ATR): ν/cm^{-1} 3058, 2960, 2901, 2865, 1671, 1559, 1517, 1507, 1477, 1438, 1362, 1305, 1250, 1201, 1157, 1145, 1024, 953, 925, 901, 825, 819 791, 755, 735, 678, 652, 599, 468, 431, 416; LR-MS (MALDI) m/z (%): 1567.69 (6), 1566.69 (7), 1565.69 (7), 1552.71 (6), 1551.70 (13), 1550.70 (18), 1549.69 (16), 1548.68 (7), 1537.71 (5), 1536.71 (10), 1535.71 (21), 1534.70 (32), 1533.70 (30), 1532.69 (16), 1531.68 (12), 1521.70 (6), 1520.71 (9) 1519.71 (16) 1518.71 (25), 1517.70 (32) 1516.69 (26), 1515.69 (14), 1506.71 (7), 1505.71 (14), 1504.72 (29), 1503.72 (60), 1502.71 (94) 1501.71 (bp), 1500.70 (65) 1499.69 (48), 1490.70 (6) 1489.70 (13), 1488.70 (21), 1487.70 (25), 1486.71 (24), 1485.71 (35), 1484.70 (M^+, 25), 1475.71 (7), 1474.71 (19), 1473.70 (42), 1472.70 (68), 1471.69 (53), 1200.51 (6), 1199.51 (9), 1198.50 (6), 861.07 (5), 833.40 (6), 832.40 (20), 831.40 (28), 672.04 (5), 656.07 (15); HR-MS (MALDI) Calcd. for $C_{116}H_{92}$: 1484.71935; Found: 1484.72062.

5,5'-([9,9':10',9'':10'',9''':10''',9'''':10'''',9''''':10''''-Sexianthracene]-10,10'''''-diyl)-bis(2,8-di-*tert*-butyl-5*H*-dibenzo[*a*,*d*]cycloheptatrien-5-yl) 6T

To a solution of $6^{2+}(BF_4^-)_2$ (51.1 mg, 27.8 μmol) in dry THF (6 mL) was added cobaltocene (10.6 mg, 56.0 μmol) at 25 °C to give a yellow suspension, and the mixture was stirred at 25 °C for 5 min under sonication. After the supernatant solution was removed by decantation, the precipitates were washed with dry CH_3CN three times and dried in vacuo to give **6T** (46.1 mg) as a deep brown powder in 100% yield.

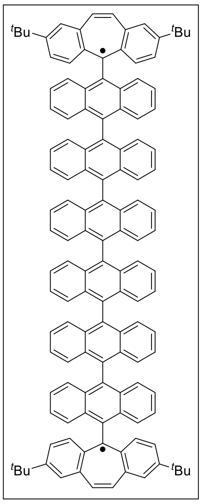

6T; Mp: 324.3 °C-340.5 °C (decomp.); IR (ATR): ν/cm^{-1} 3058, 2960, 2902, 2865, 1582, 1517, 1477, 1438, 1394, 1382, 1361, 1322, 1305, 1249, 1201, 1157, 1145, 1065, 1024, 954, 910, 903, 831, 818, 793, 756, 735, 667, 649, 607, 599, 436, 420; LR-MS (MALDI) m/z (%): 1727.89 (5), 1726.89 (6), 1725.88 (6), 1712.89 (6), 1711.89 (10), 1710.89 (14), 1709.89 (15), 1708.88 (10), 1707.87 (8), 1697.88 (5), 1696.88 (8), 1695.89 (11), 1694.89 (14), 1693.89 (15), 1692.88 (13), 1691.87 (9), 1682.89 (6), 1681.89 (12), 1680.89 (21), 1679.89 (31), 1678.89 (36), 1677.89 (44), 1676.88 (42), 1675.88 (25), 1667.88 (5), 1666.88 (8), 1665.88 (15), 1664.88 (28), 1663.89 (40), 1662.89 (43), 1661.90 (46), 1660.90 (M^+, 31), 1651.88 (8), 1650.88 (18), 1649.89 (38), 1648.89 (57), 1647.88 (36), 1636.86 (6), 1635.86 (8), 877.14 (9), 862.17 (15), 861.16 (40), 847.19 (8), 846.19 (18), 845.19 (36), 686.53 (11), 685.53 (28), 671.42 (9), 670.41 (5), 666.11 (10), 657.17 (29), 656.16 (bp), 651.14 (10), 650.14 (39), 635.16 (8), 634.16 (23); HR-MS (MALDI) Calcd. for $C_{130}H_{100}$: 1660.78190; Found: 1660.78022.

NMR spectra of new compounds

(a)

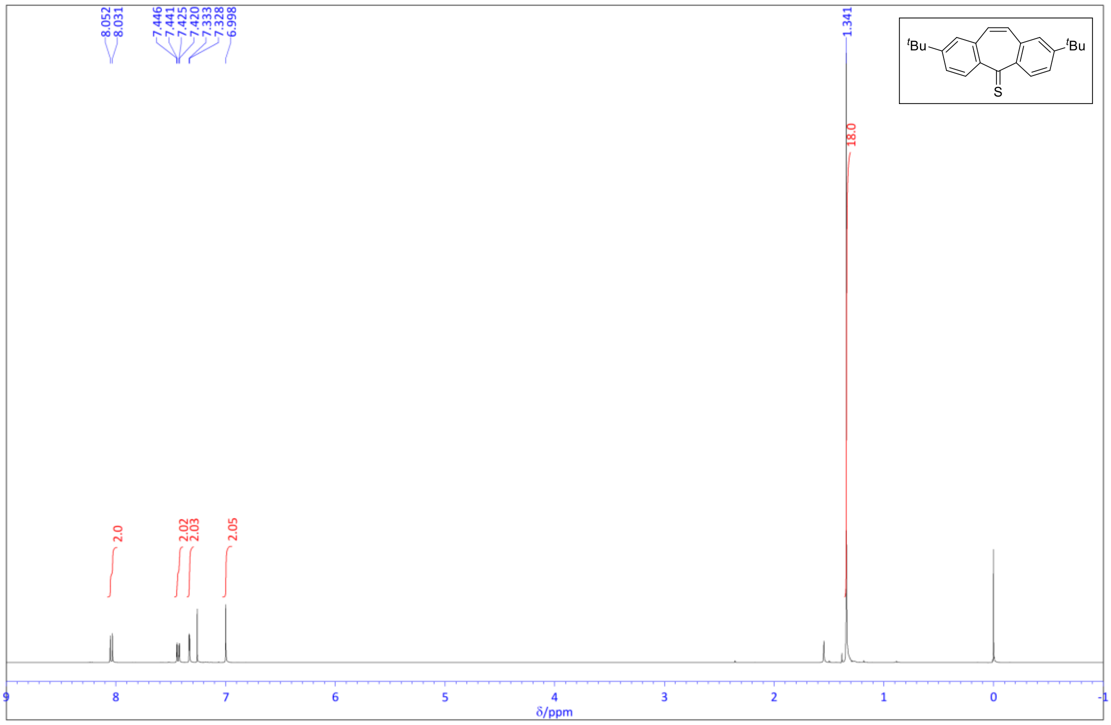

(b)

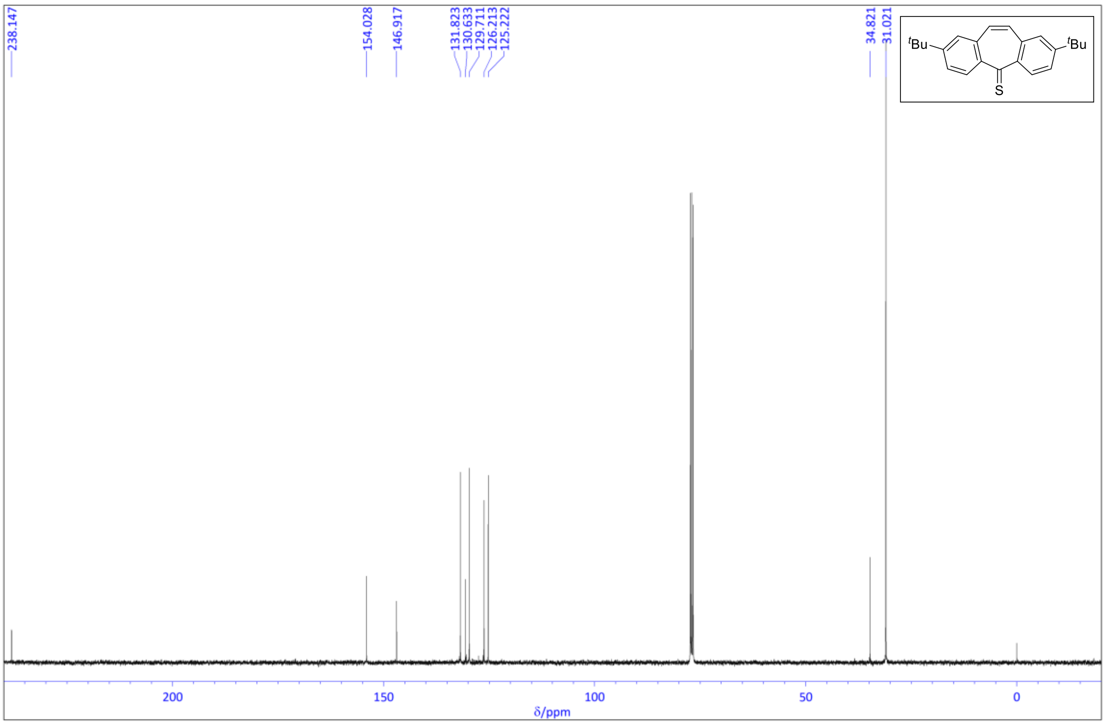

Figure S1. (a) ^{1}H NMR and (b) ^{13}C NMR spectra of **S2** in $CDCl_3$.

(a)

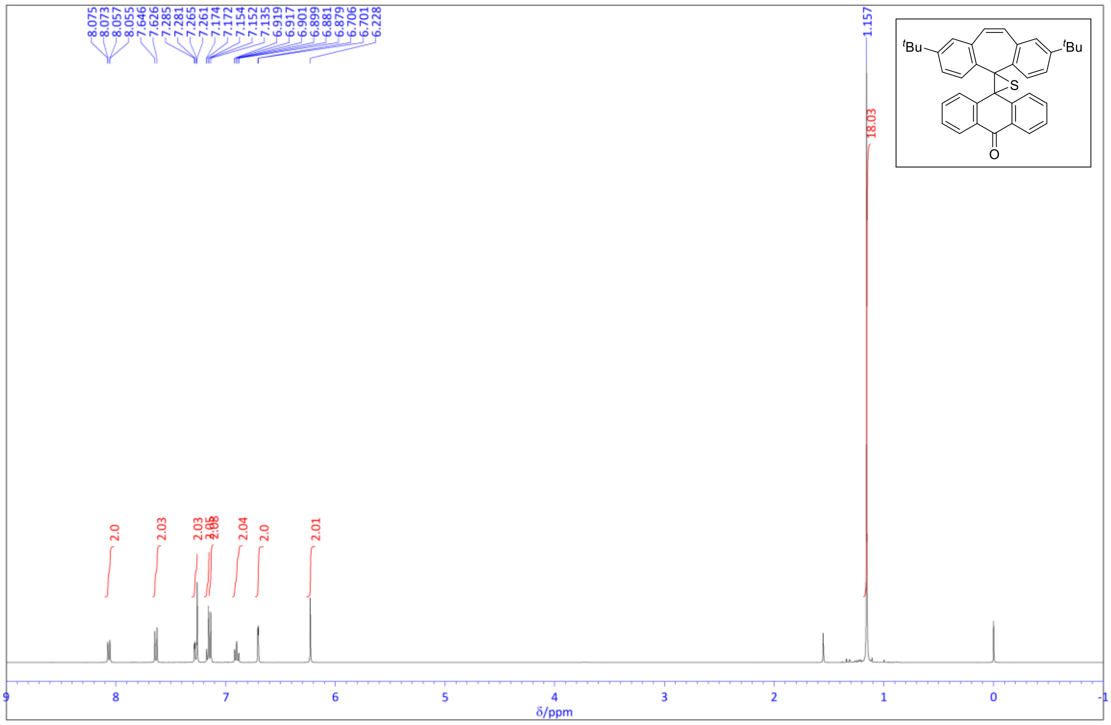

(b)

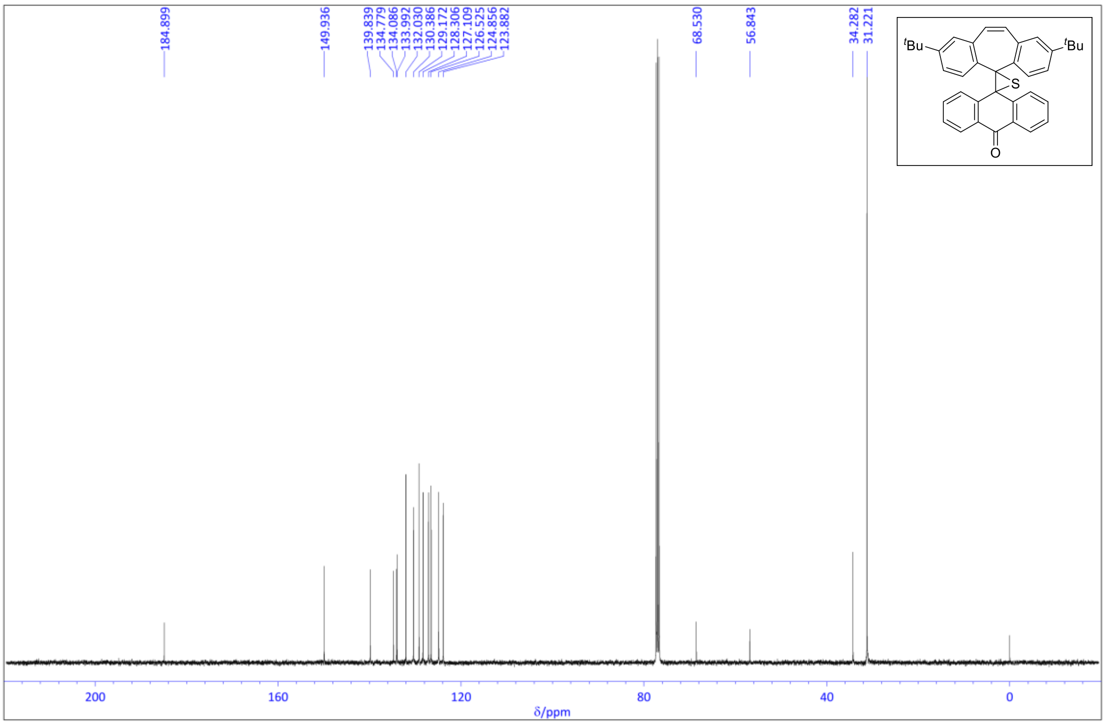

Figure S2. (a) ^{1}H NMR and (b) ^{13}C NMR spectra of **S3** in $CDCl_3$.

(a)

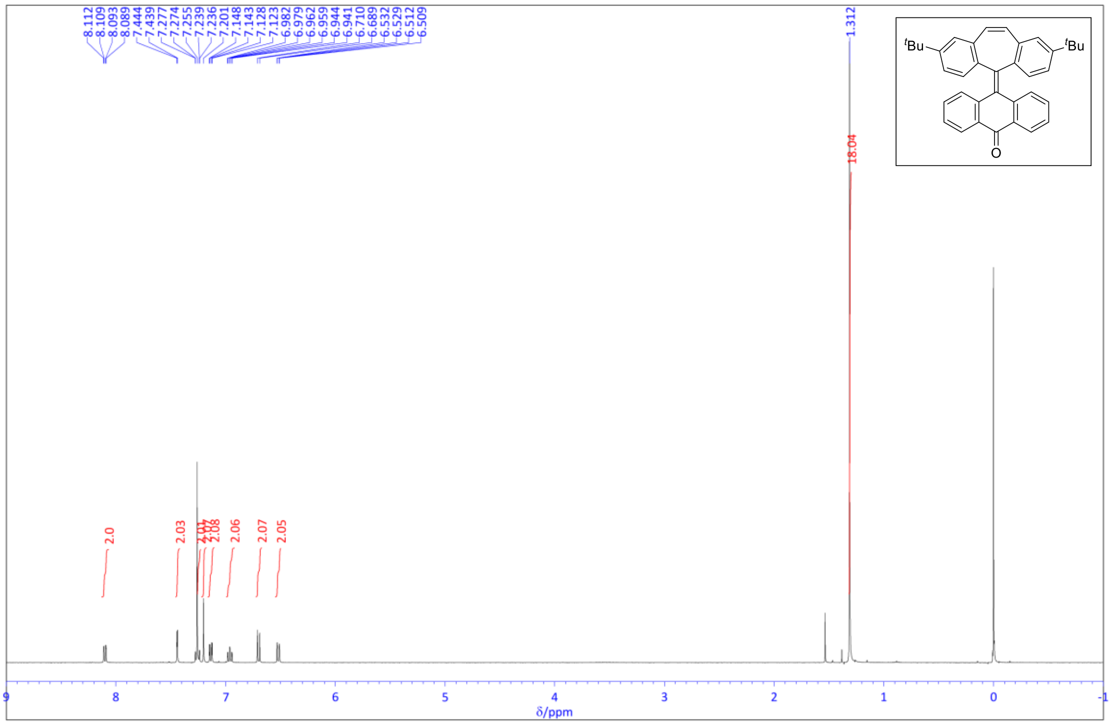

(b)

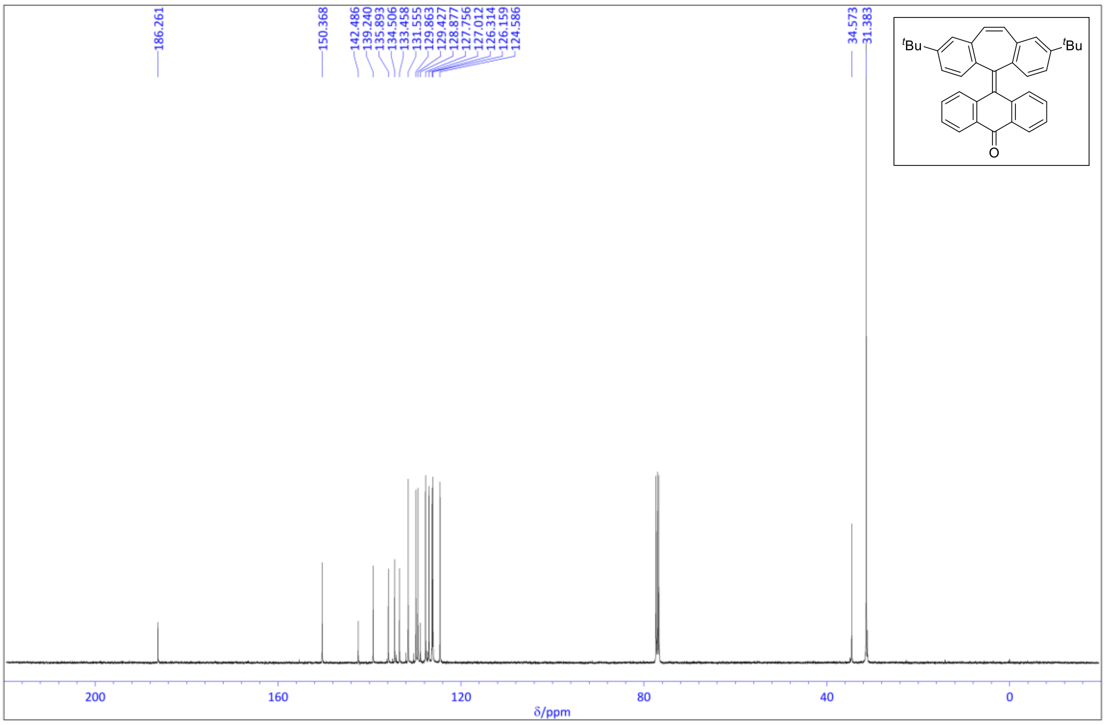

Figure S3. (a) ^{1}H NMR and (b) ^{13}C NMR spectra of **S4** in $CDCl_3$.

(a)

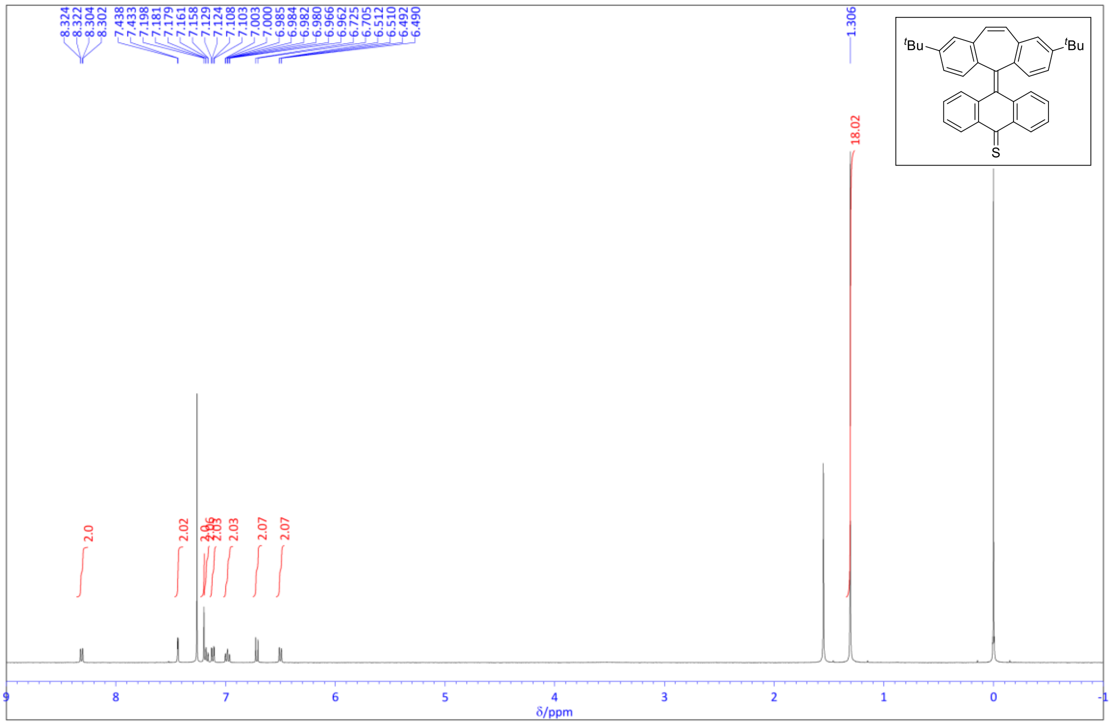

(b)

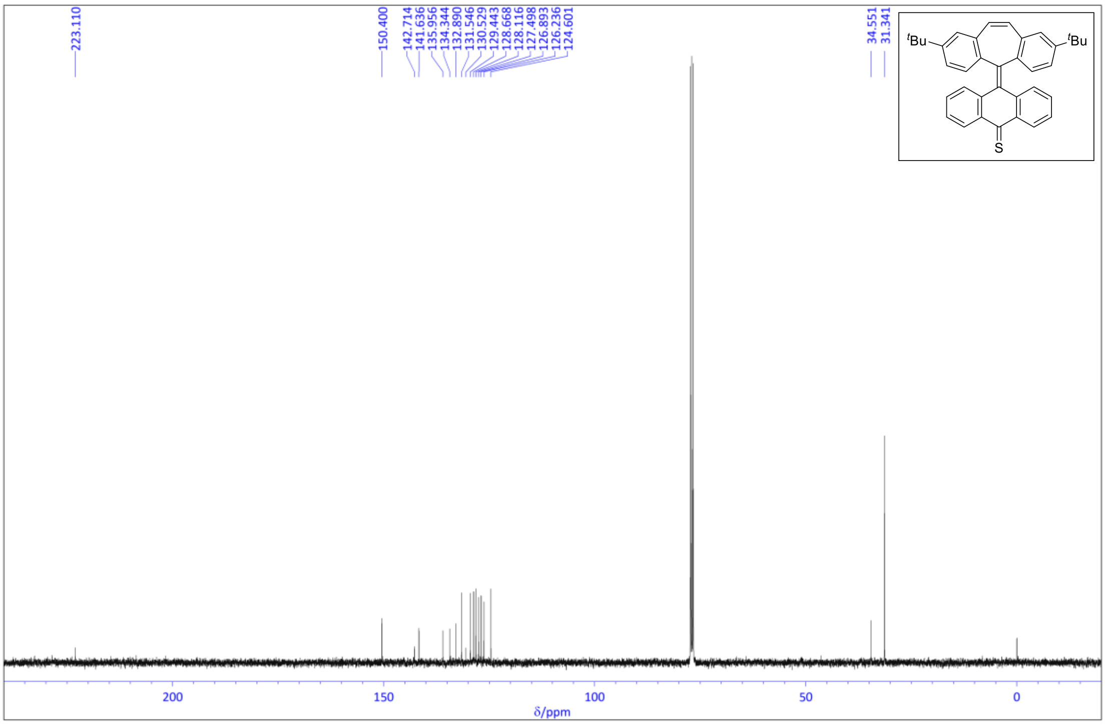

Figure S4. (a) ^{1}H NMR and (b) ^{13}C NMR spectra of **S5** in $CDCl_3$.

(a)

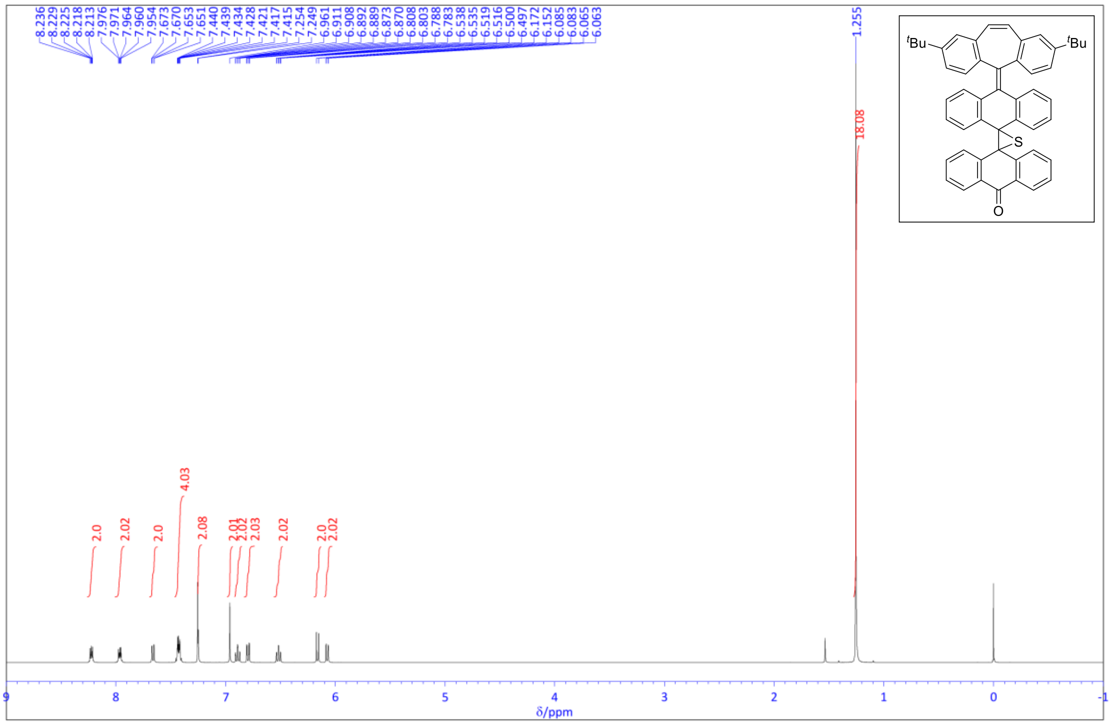

(b)

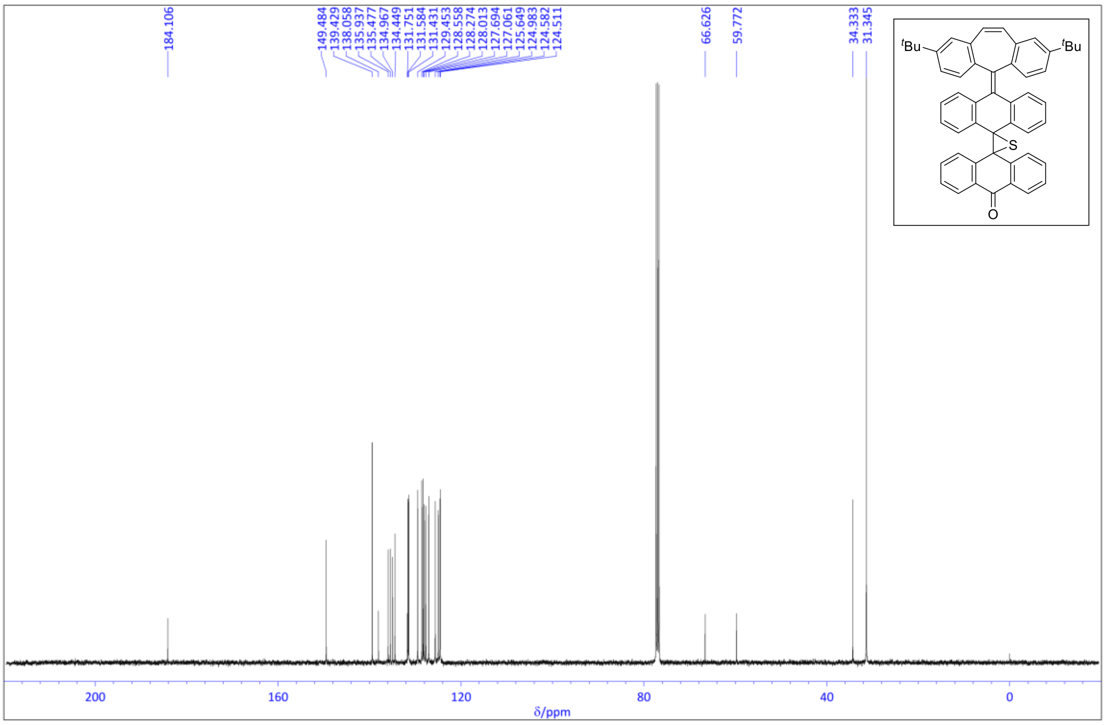

Figure S5. (a) ^{1}H NMR and (b) ^{13}C NMR spectra of **S6** in $CDCl_3$.

(a)

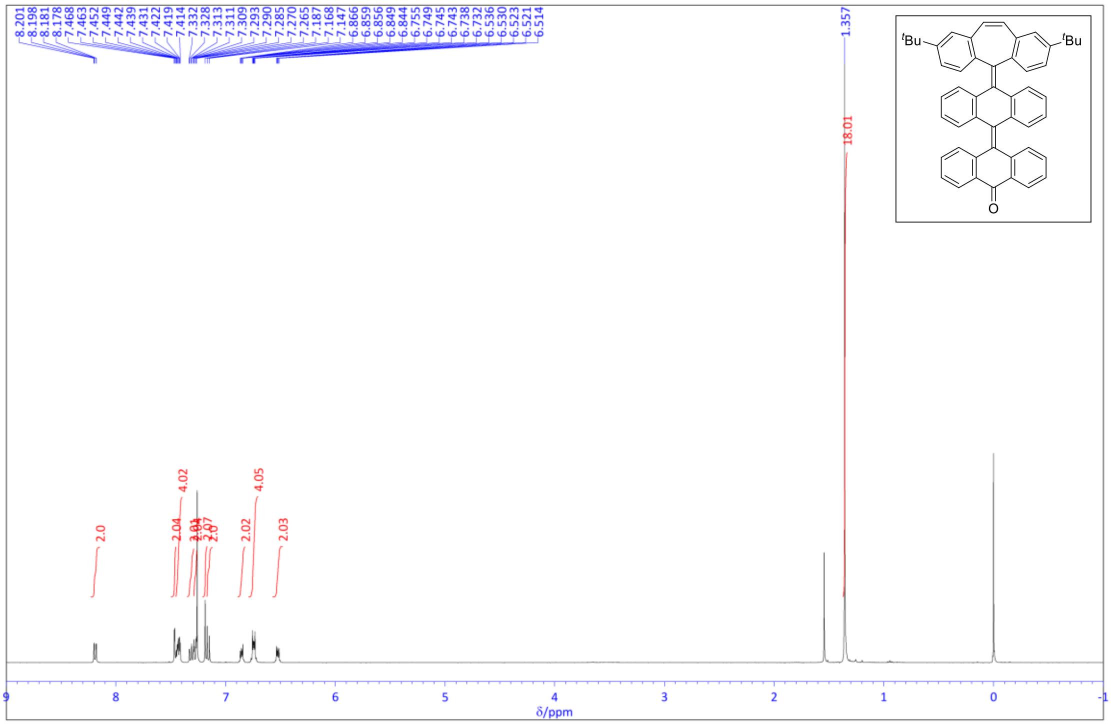

(b)

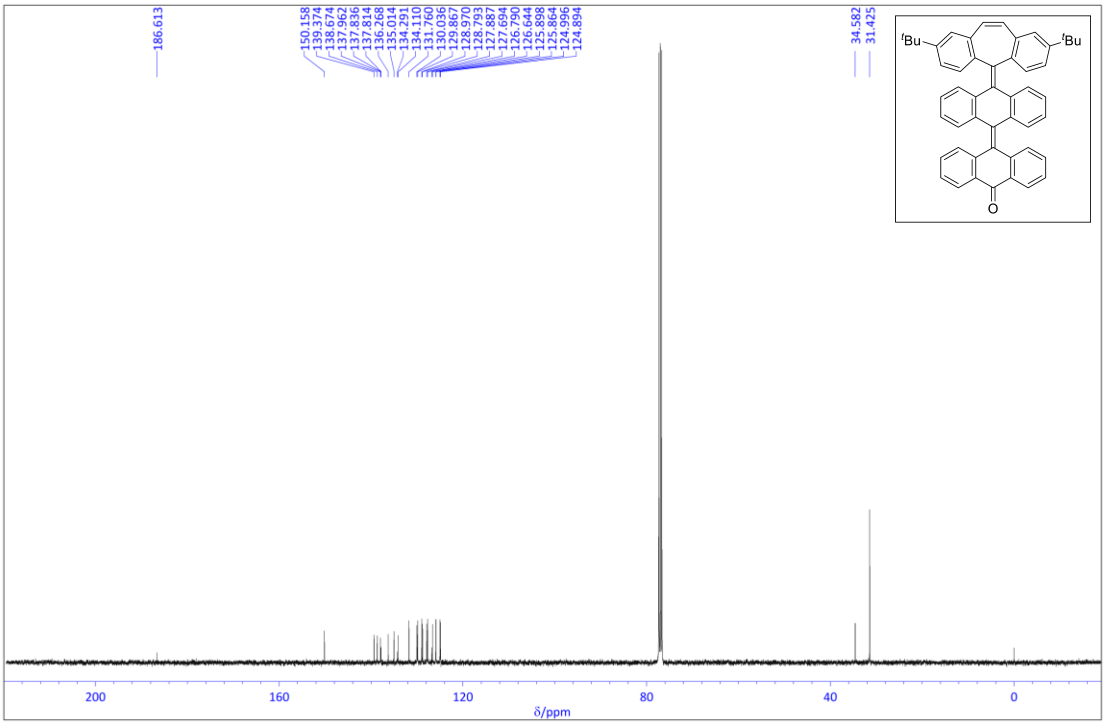

Figure S6. (a) ^{1}H NMR and (b) ^{13}C NMR spectra of **S7** in $CDCl_3$.

(a)

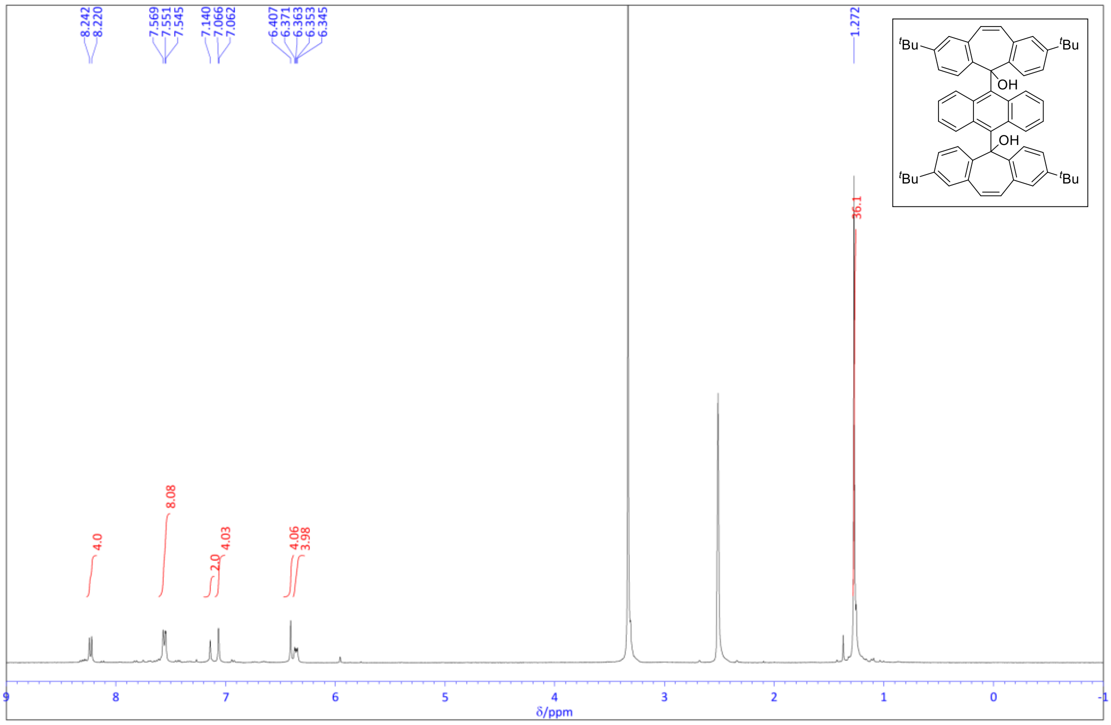

(b)

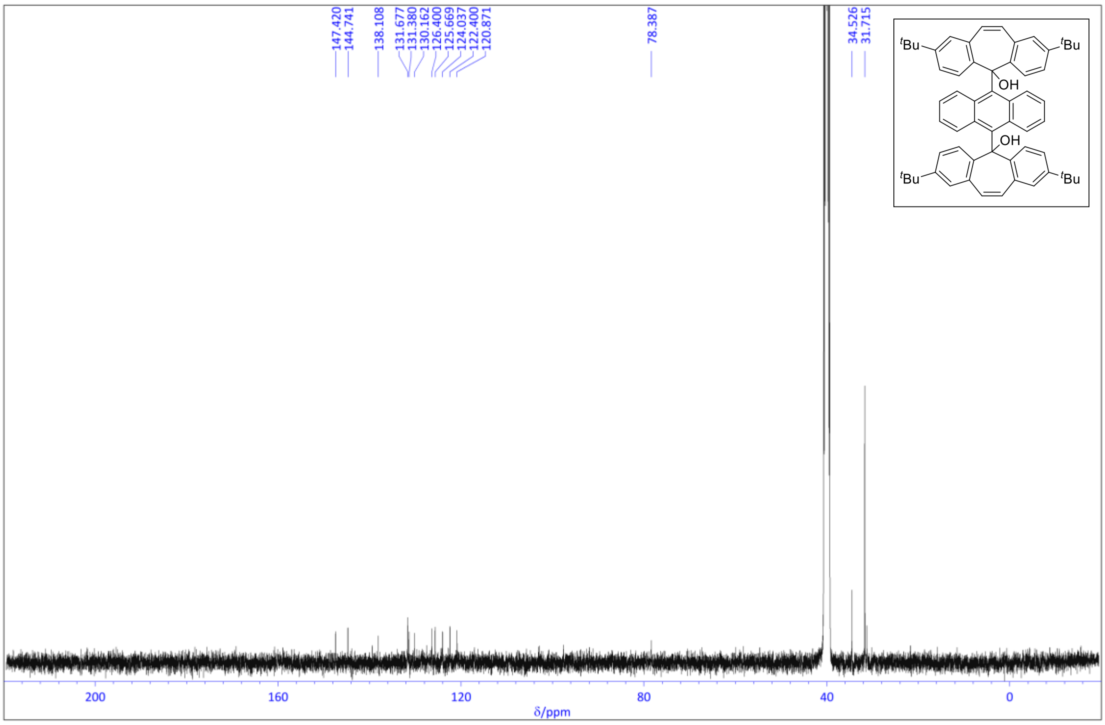

Figure S7. (a) ^{1}H NMR and (b) ^{13}C NMR spectra of **1-OH** in DMSO-d_6.

(a)

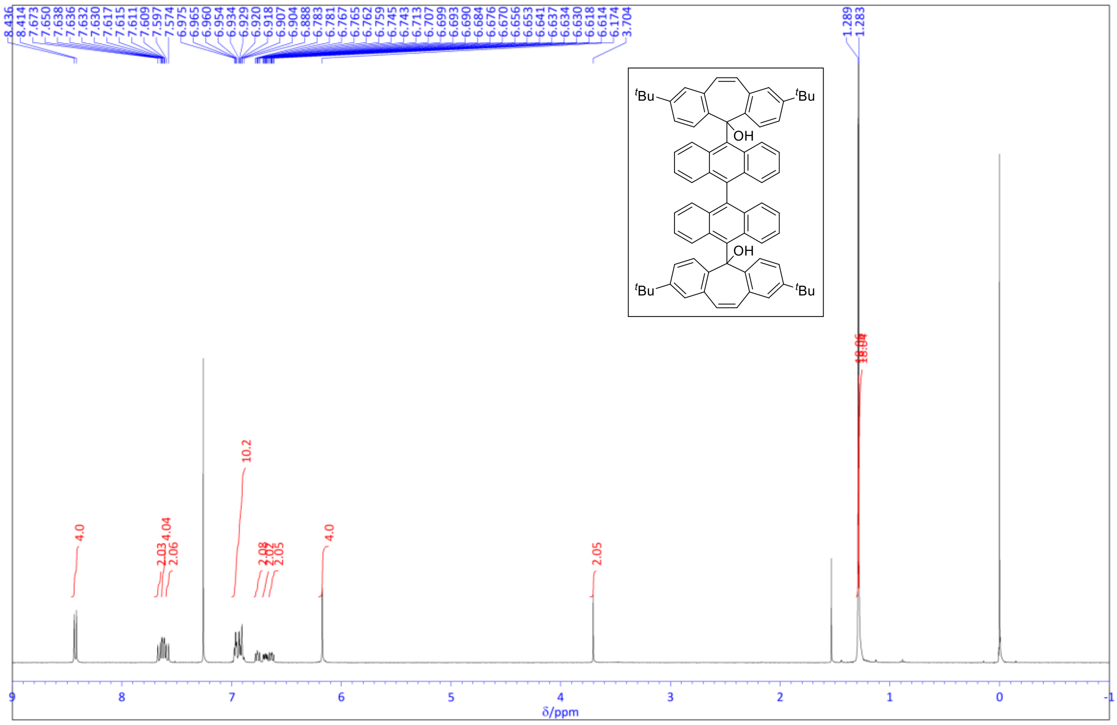

(b)

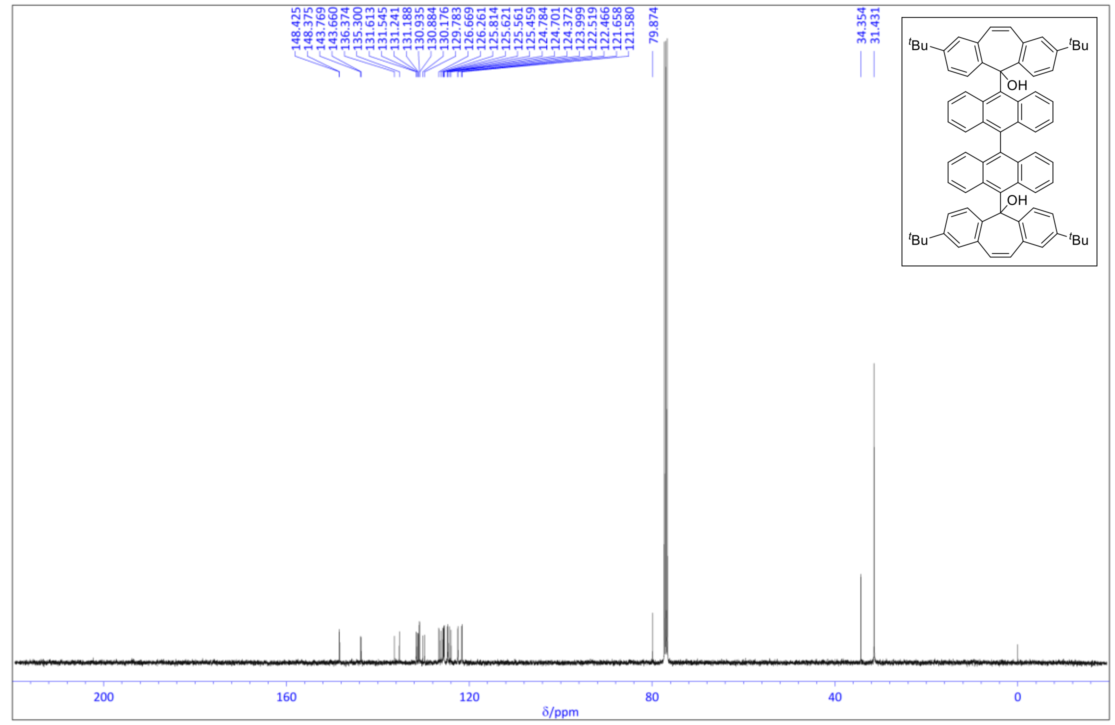

Figure S8. (a) ^{1}H NMR and (b) ^{13}C NMR spectra of **2-OH** in $CDCl_3$.

(a)

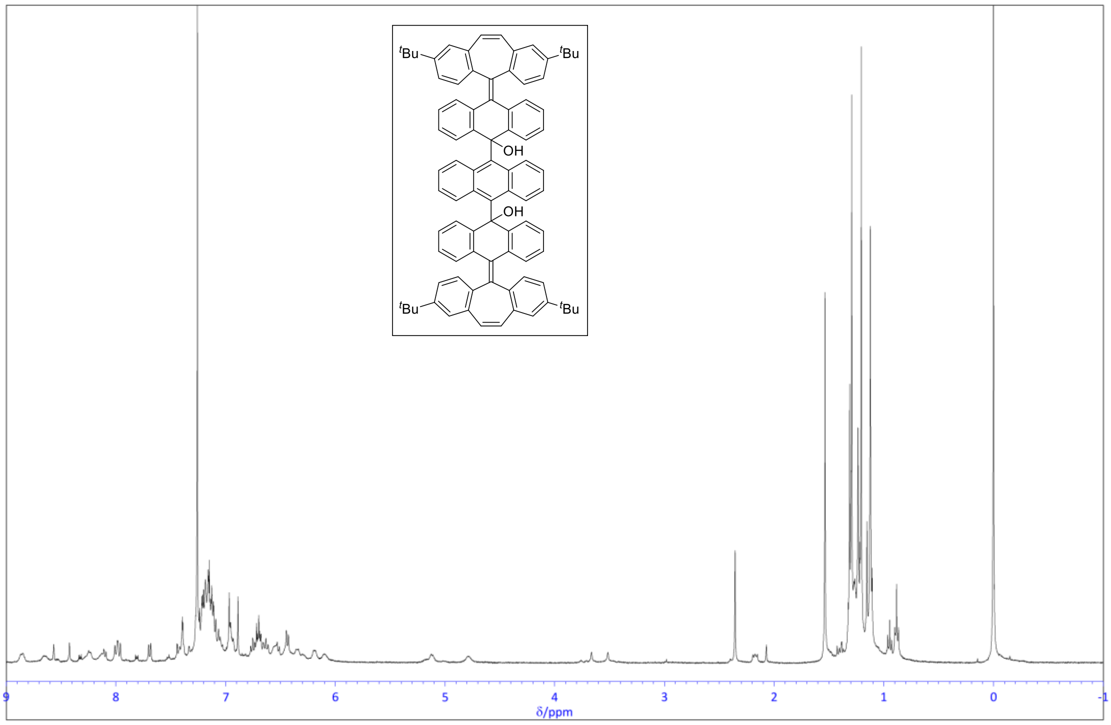

(b)

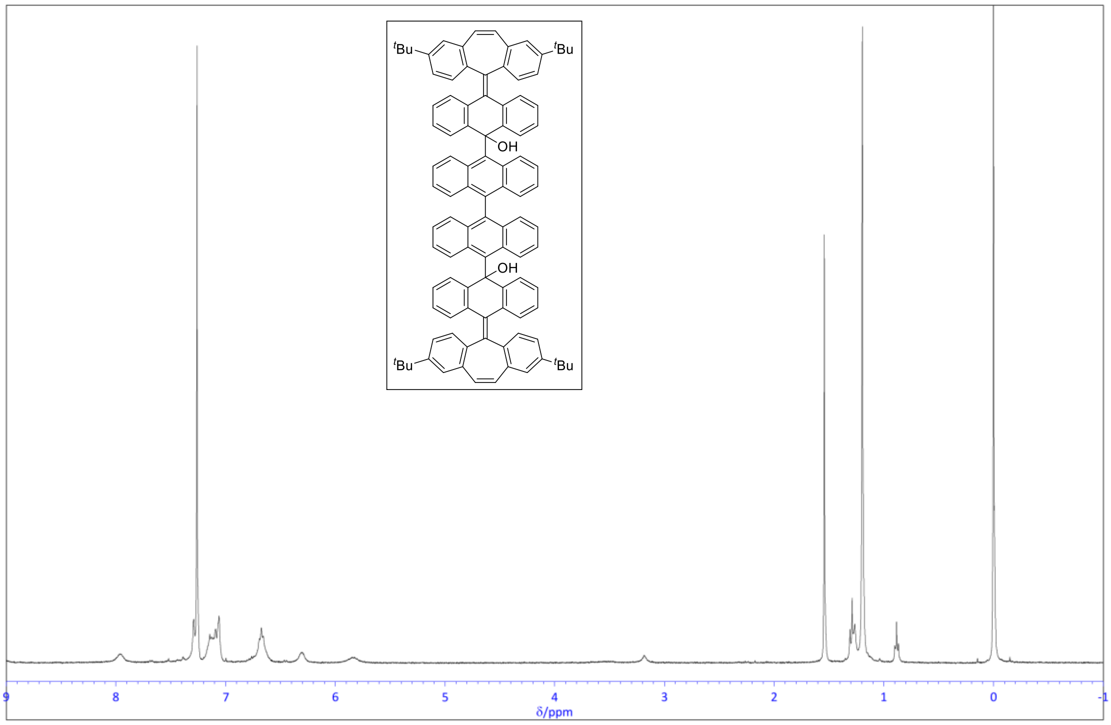

Figure S9. ^{1}H NMR of (a) **3-OH** and (b) **4-OH** in $CDCl_3$.

(a)

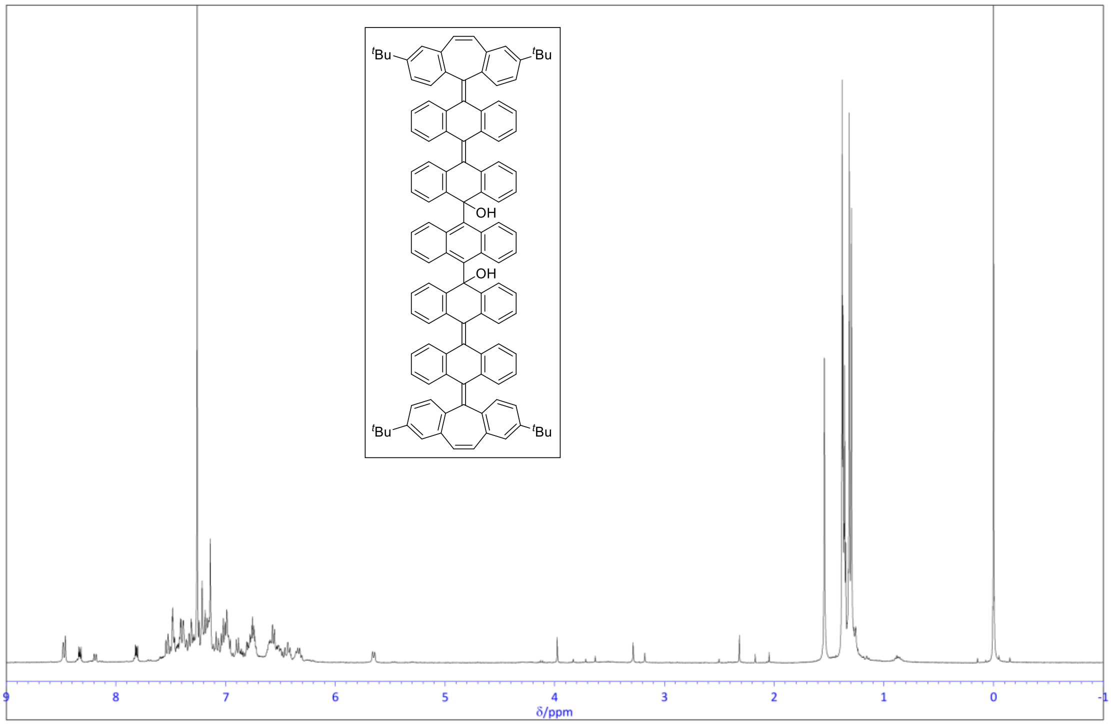

(b)

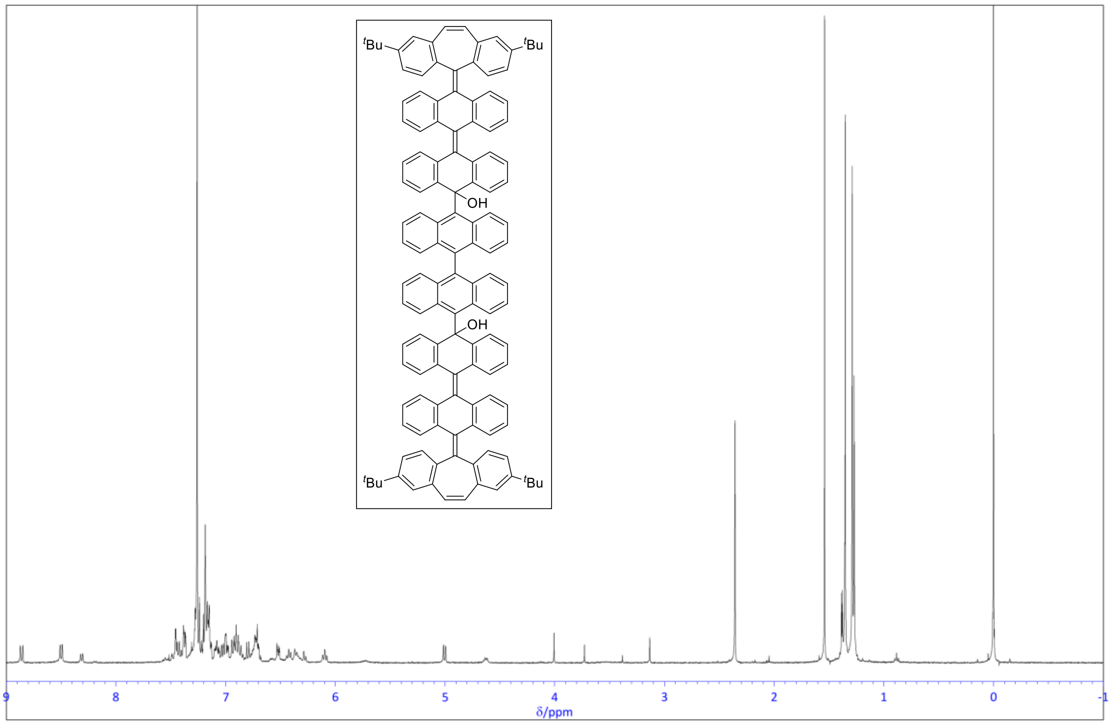

Figure S10. ^{1}H NMR of (a) **5-OH** and (b) **6-OH** in $CDCl_3$.

(a)

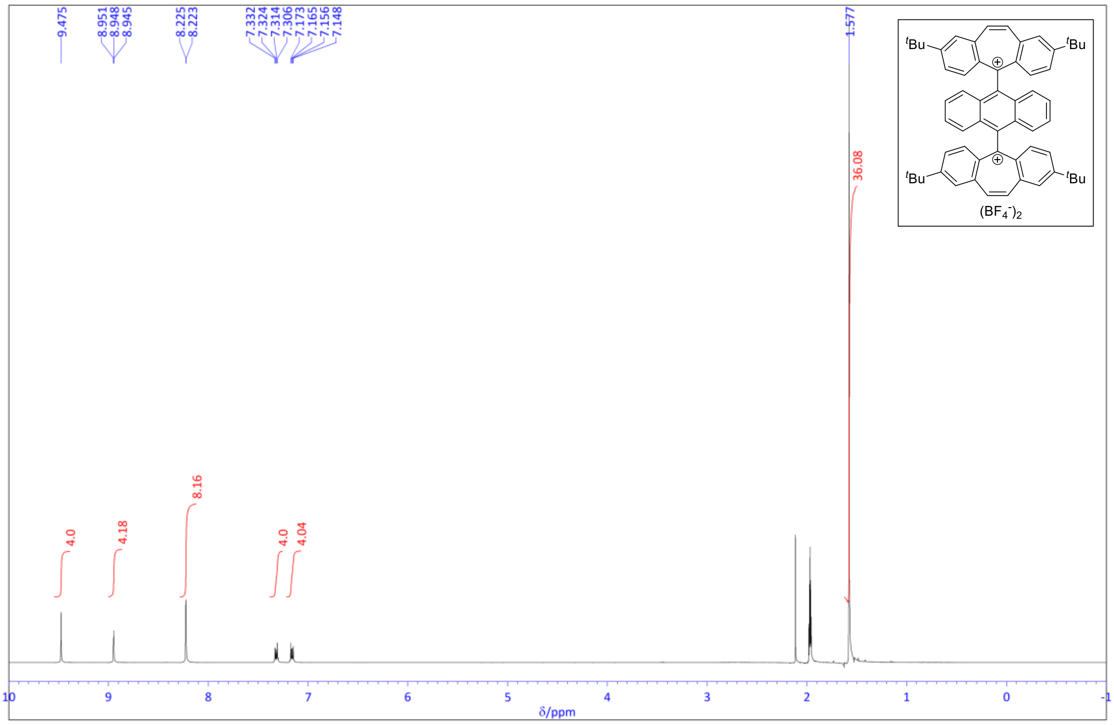

(b)

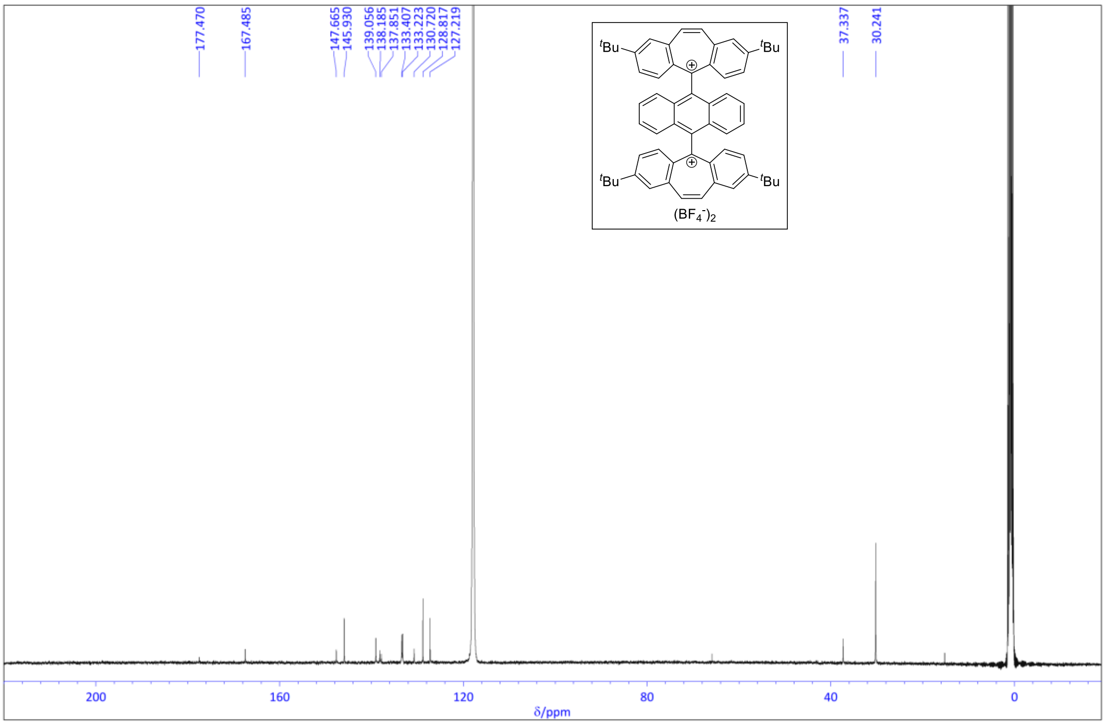

Figure S11. (a) ^{1}H NMR and (b) ^{13}C NMR spectra of $\mathbf{1}^{2+}(BF_4^-)_2$ in CD_3CN.

(a)

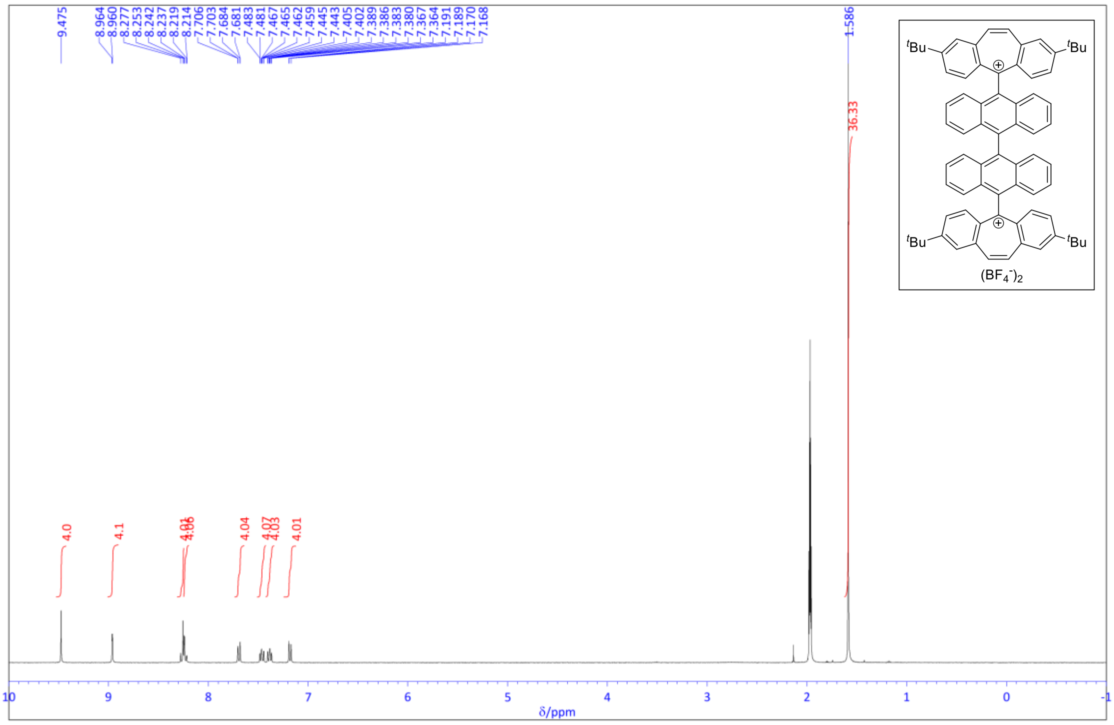

(b)

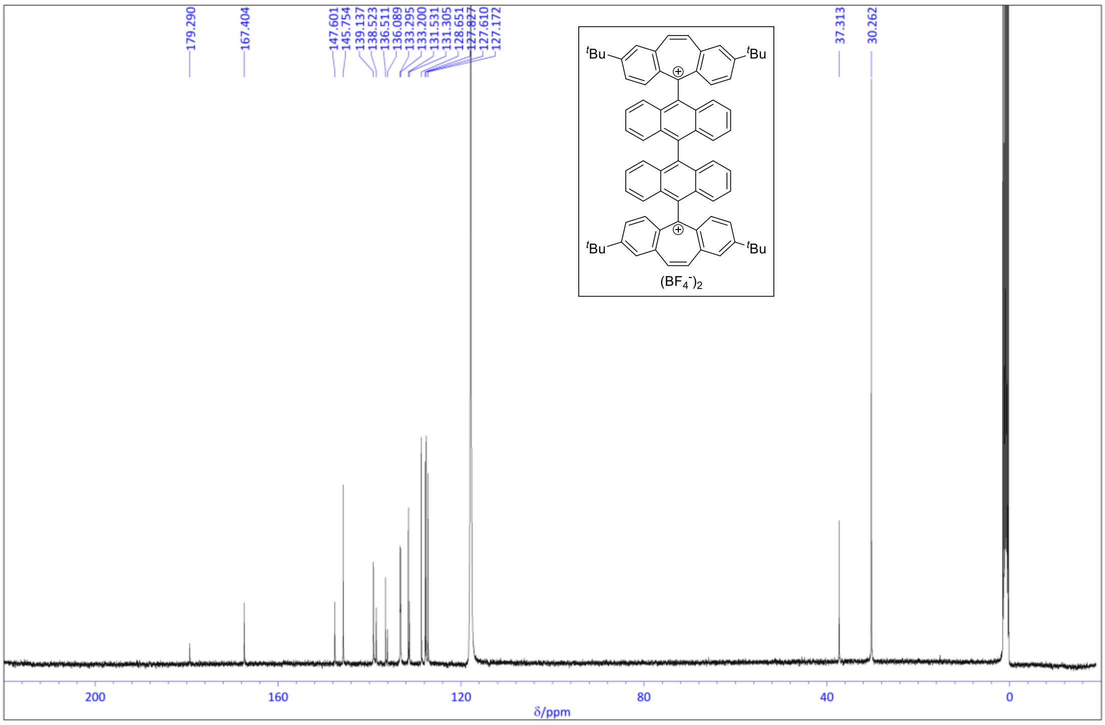

Figure S12. (a) ^{1}H NMR and (b) ^{13}C NMR spectra of **2**$^{2+}$(BF_4^-)$_2$ in CD_3CN.

(a)

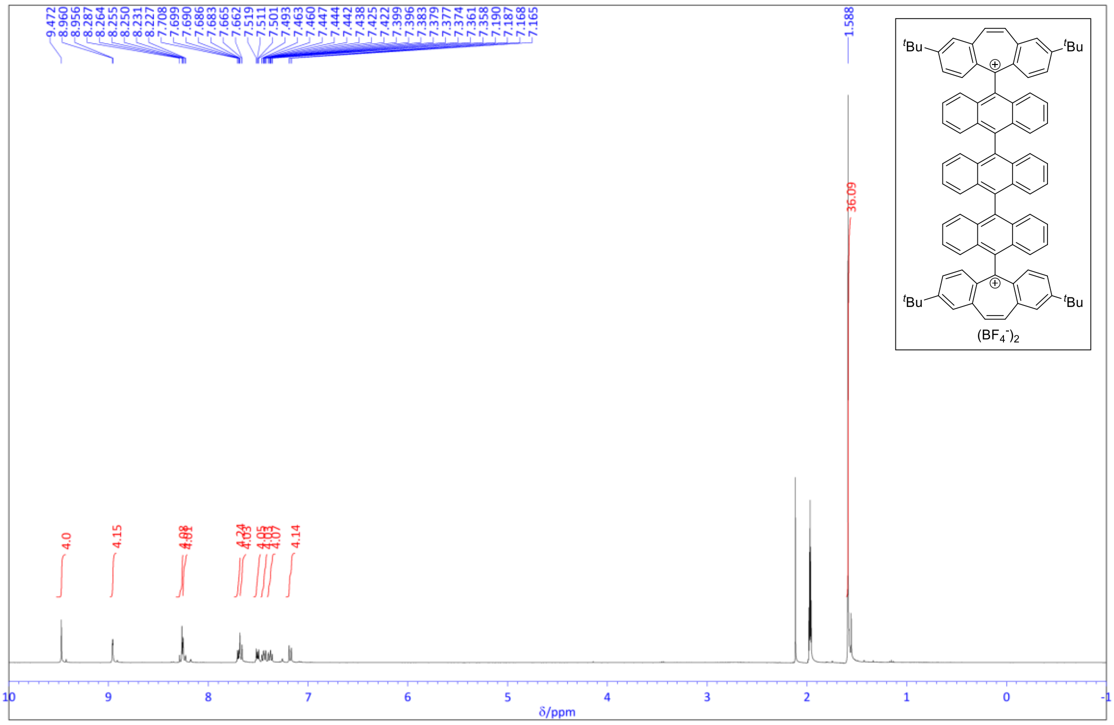

(b)

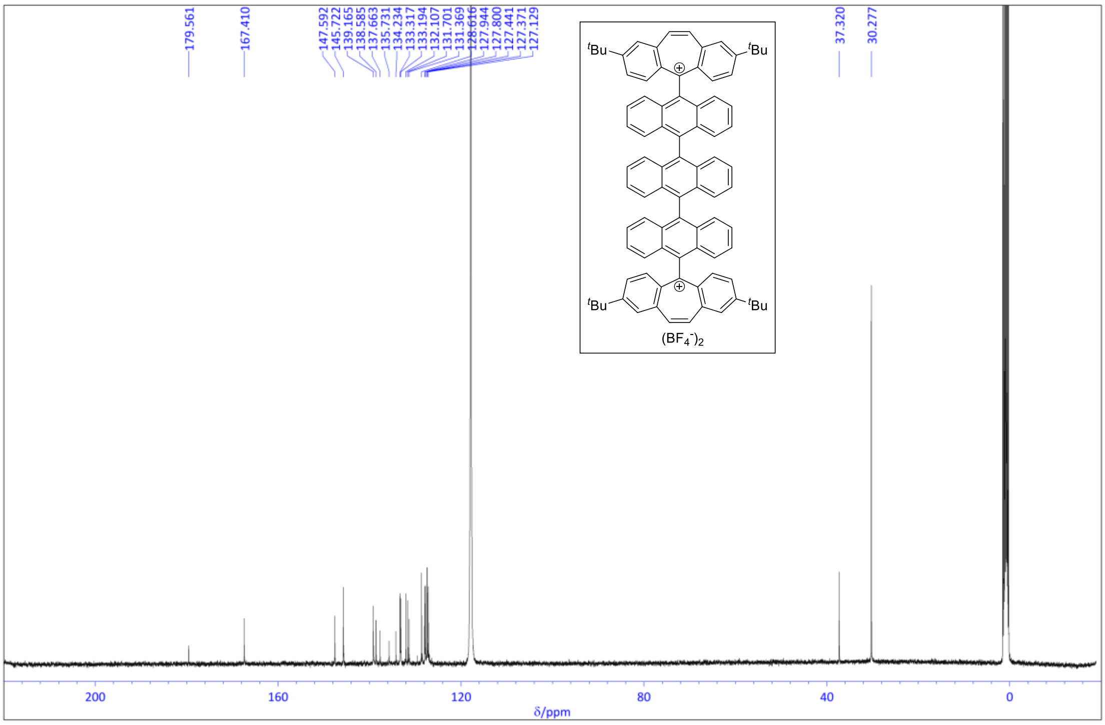

Figure S13. (a) ^{1}H NMR and (b) ^{13}C NMR spectra of $\mathbf{3}^{2+}(BF_4^-)_2$ in CD_3CN.

(a)

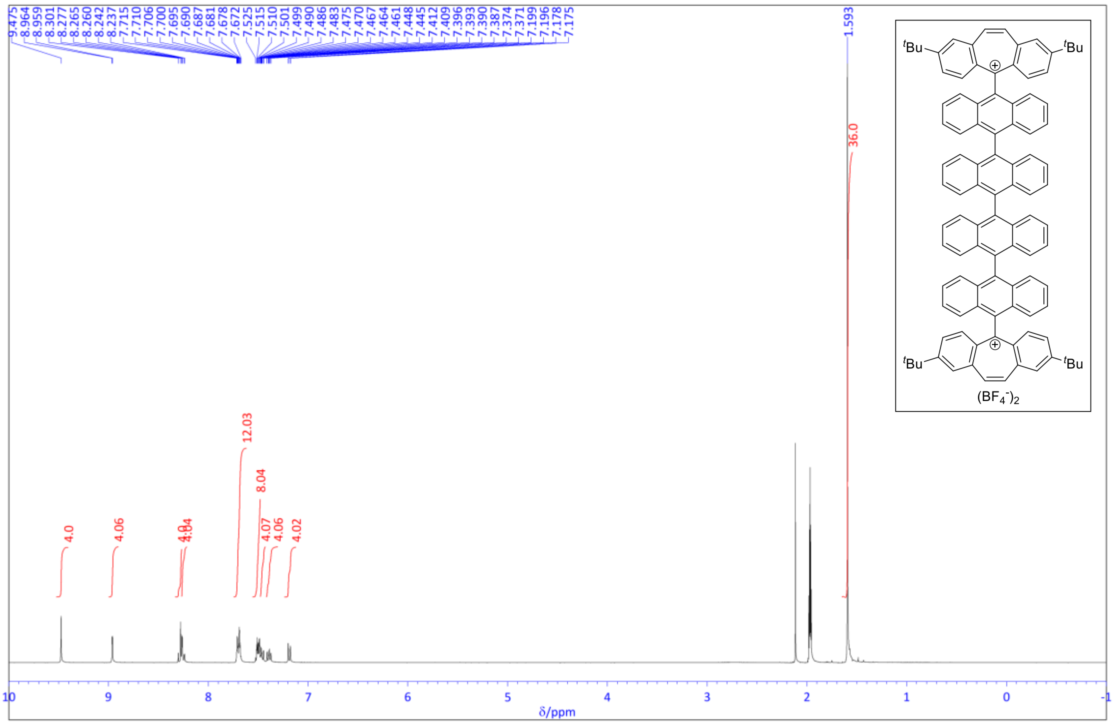

(b)

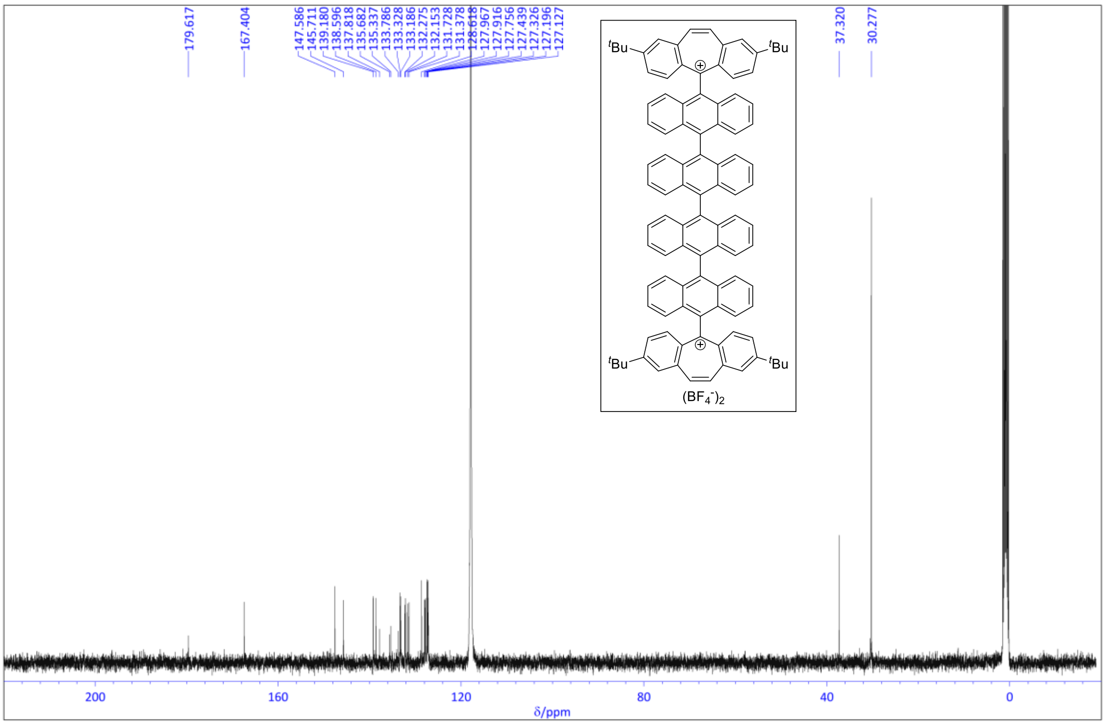

Figure S14. (a) ^{1}H NMR and (b) ^{13}C NMR spectra of $\mathbf{4}^{2+}(BF_4^-)_2$ in CD_3CN.

(a)

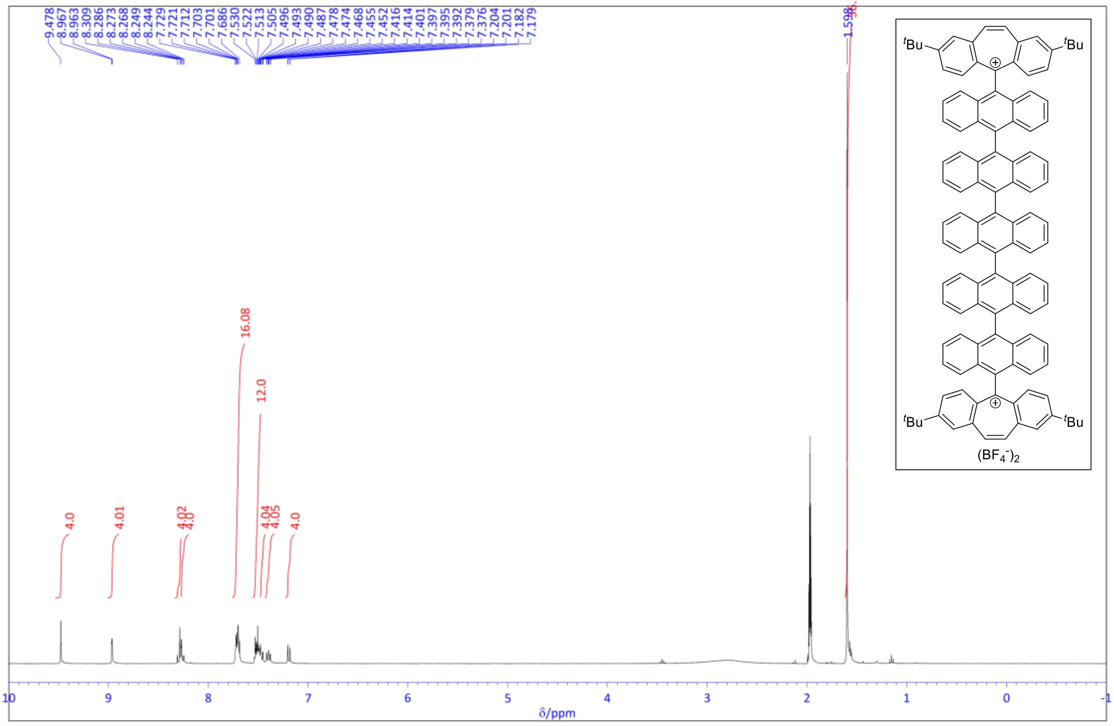

(b)

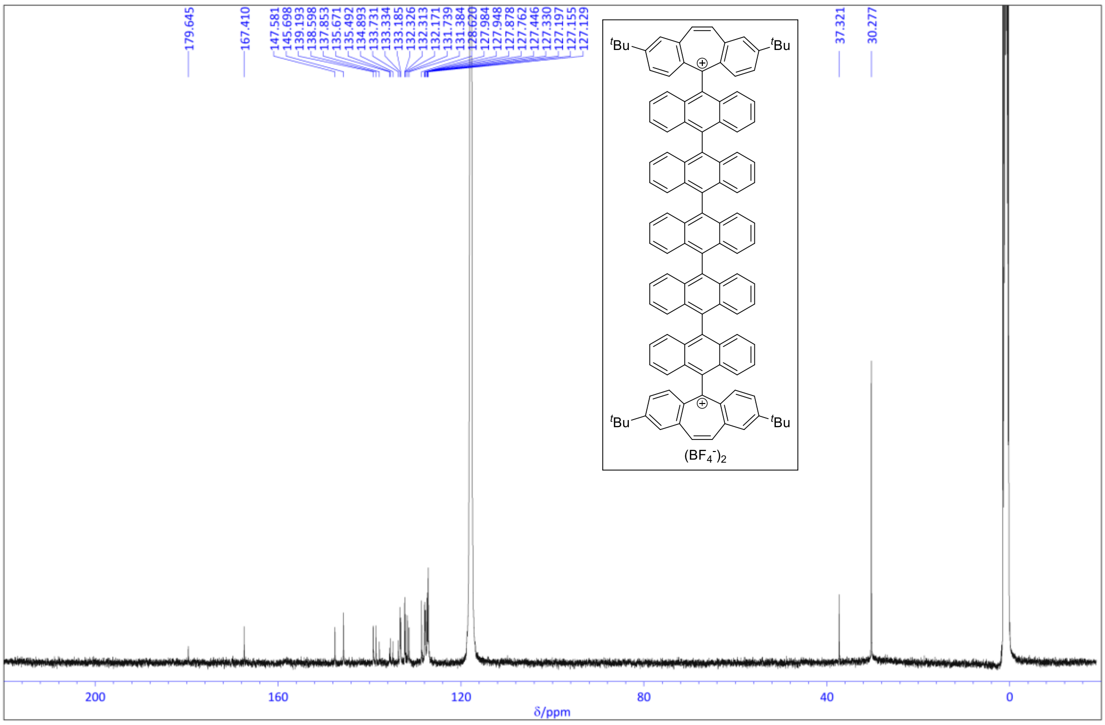

Figure S15. (a) ^{1}H NMR and (b) ^{13}C NMR spectra of **5**$^{2+}$(BF_4^-)$_2$ in CD_3CN.

(a)

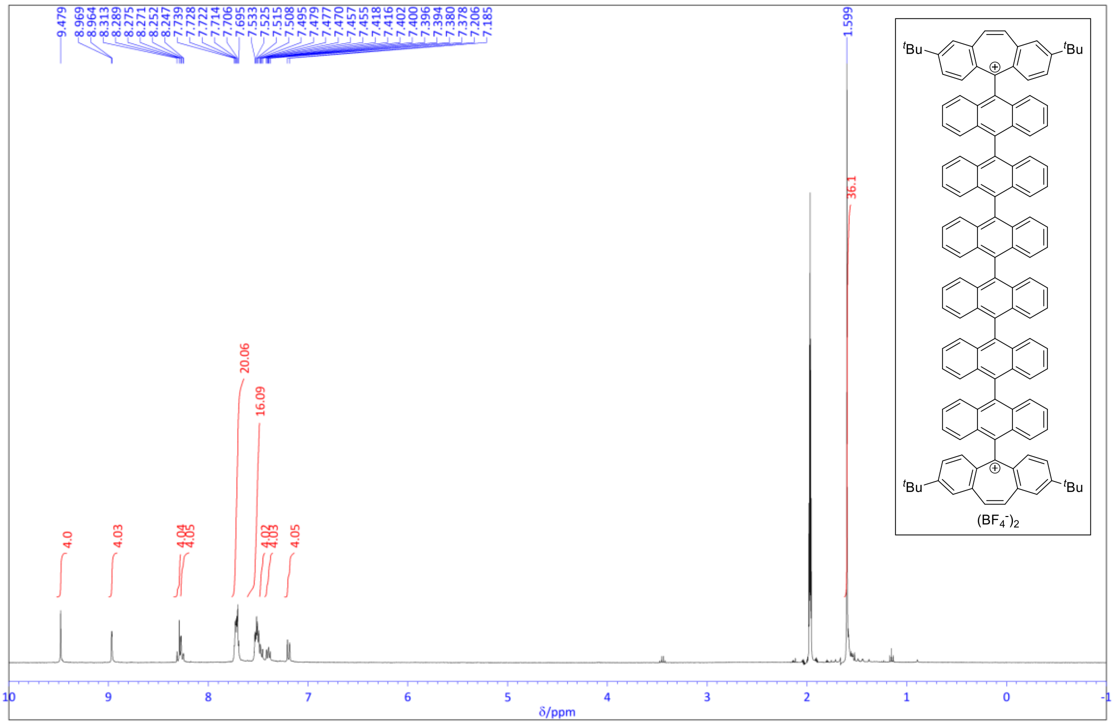

(b)

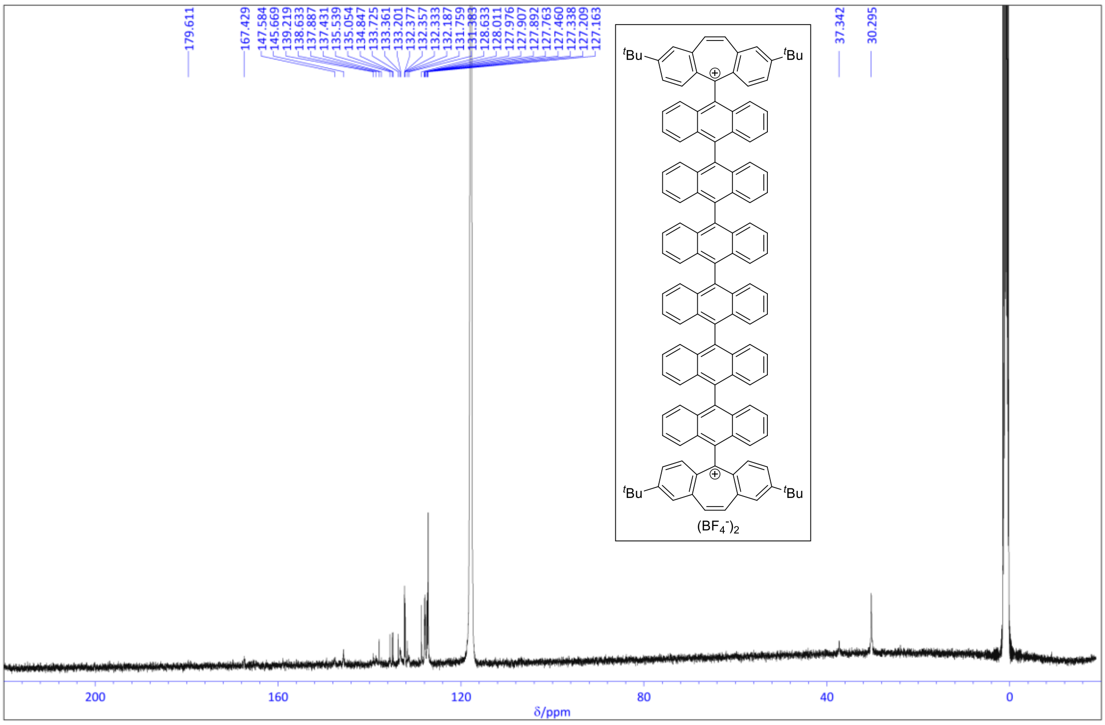

Figure S16. (a) ^{1}H NMR and (b) ^{13}C NMR spectra of **6**$^{2+}(BF_4^{-})_2$ in CD_3CN.

(a)

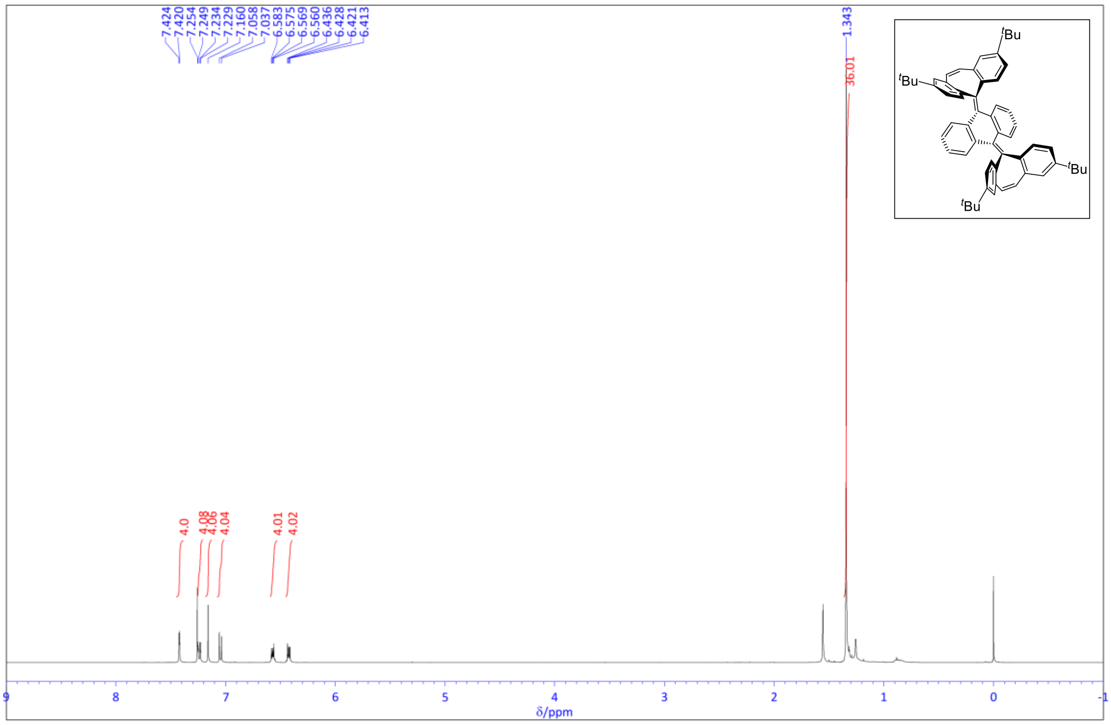

(b)

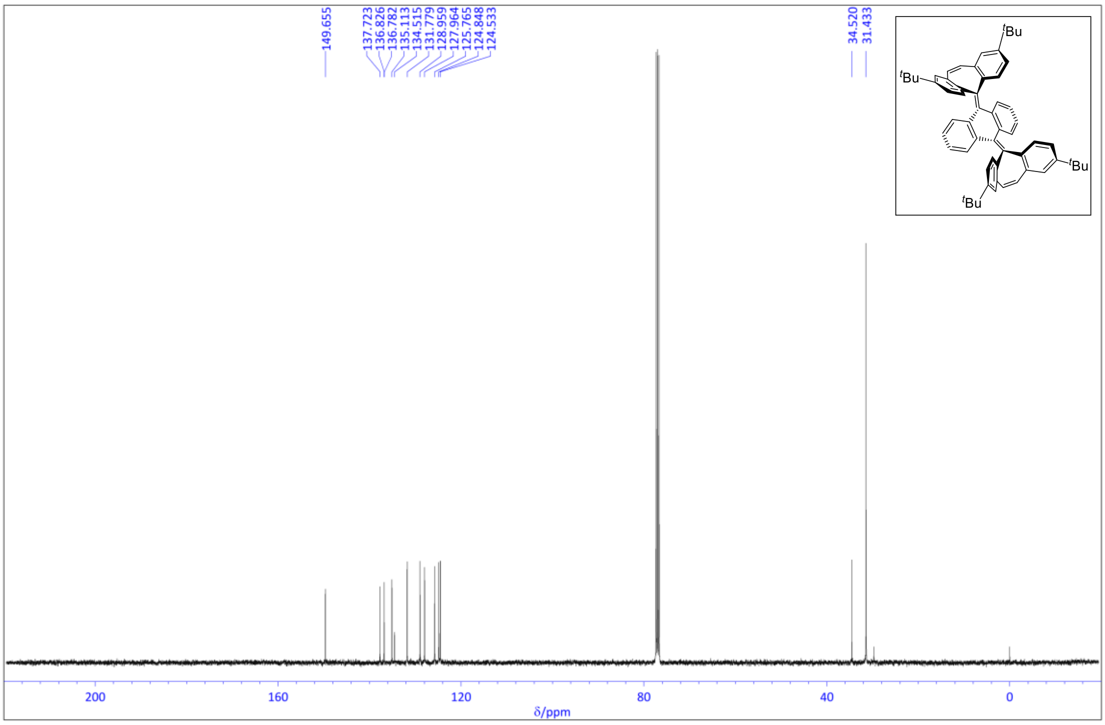

Figure S17. (a) ^{1}H NMR and (b) ^{13}C NMR spectra of **1F**$_{\boldsymbol{anti,anti}}$ in $CDCl_3$.

(a)

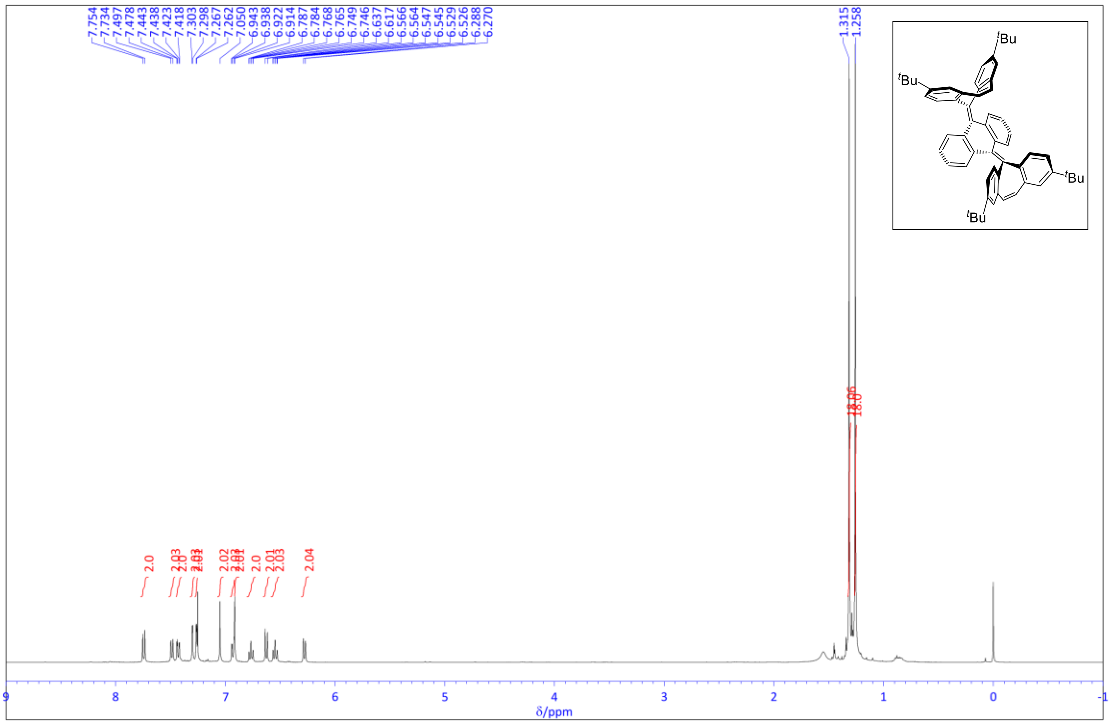

(b)

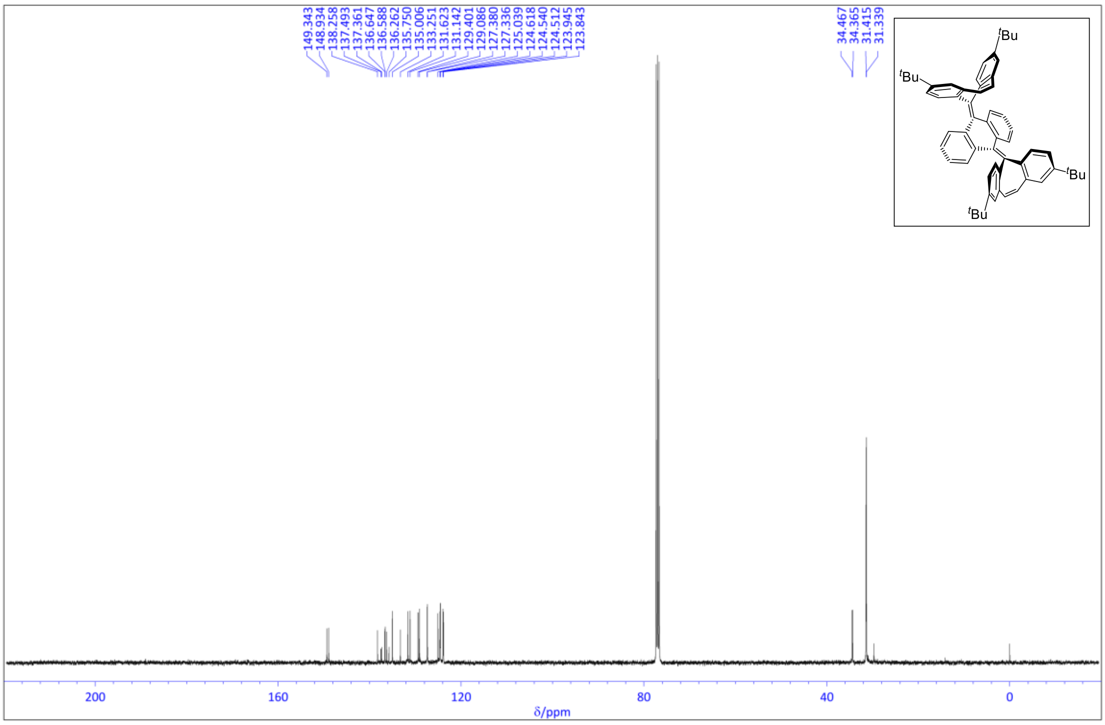

Figure S18. (a) ^{1}H NMR and (b) ^{13}C NMR spectra of **1F**$_{syn,anti}$ in $CDCl_3$.

(a)

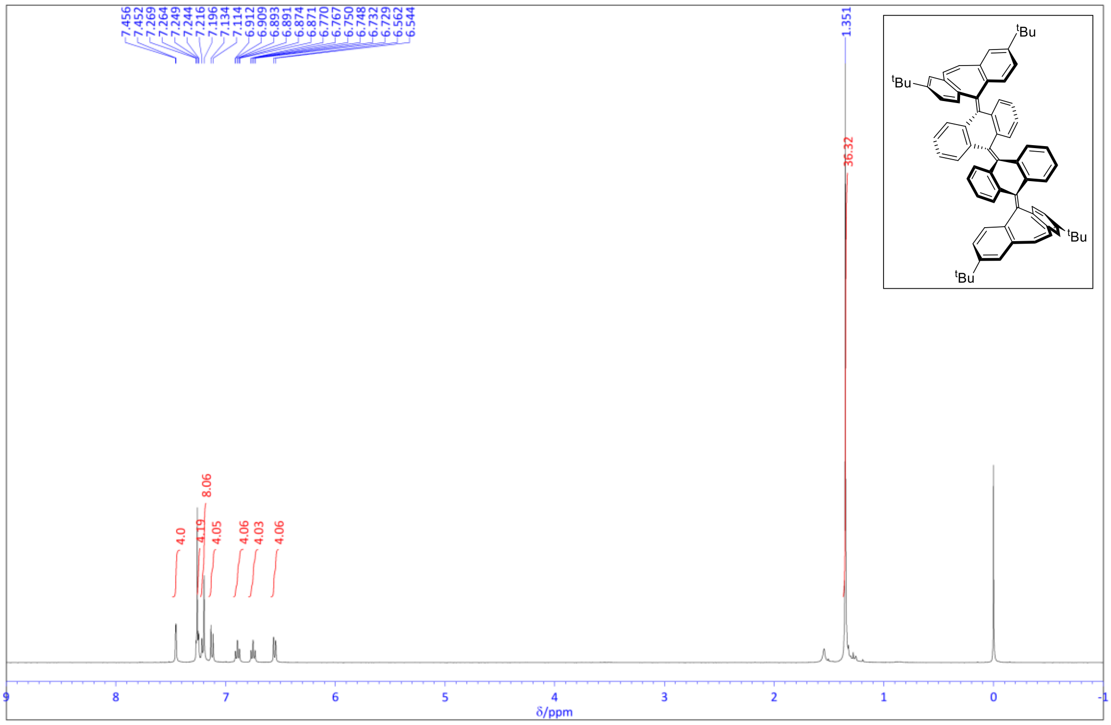

(b)

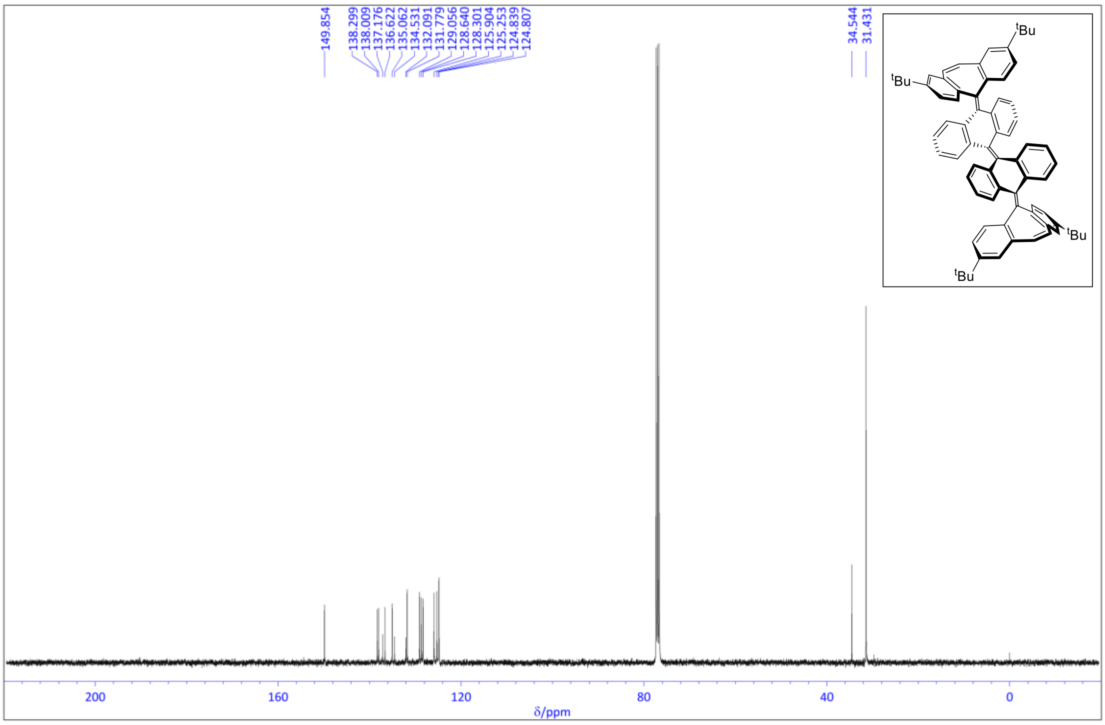

Figure S19. (a) ^{1}H NMR and (b) ^{13}C NMR spectra of **2F** in $CDCl_3$.

(a)

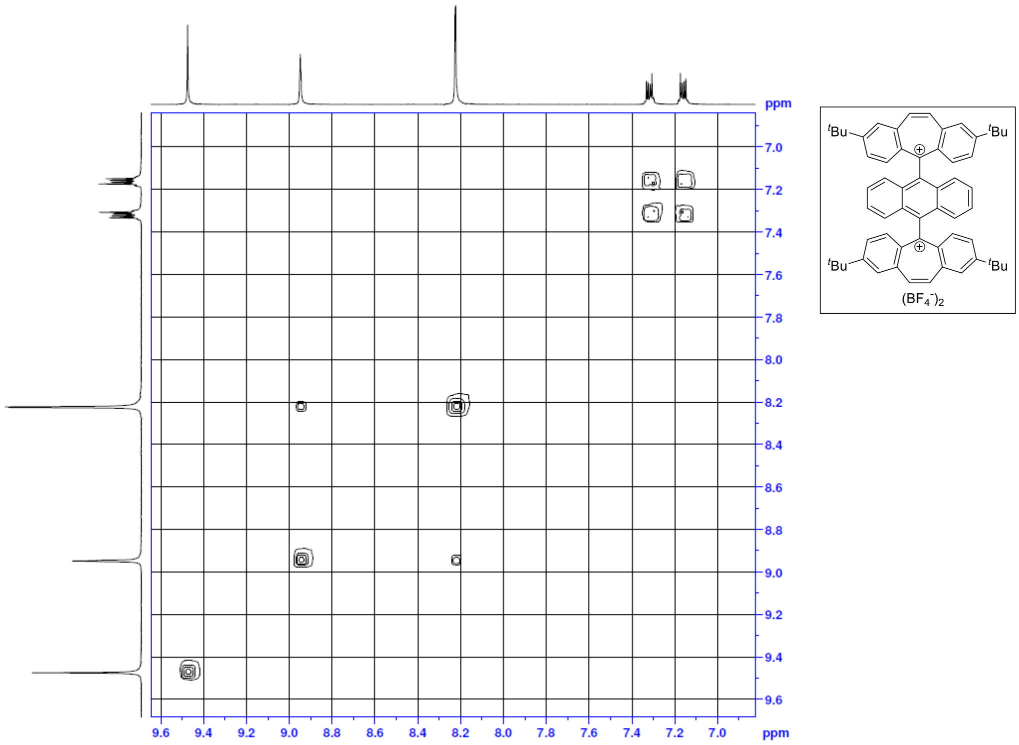

(b)

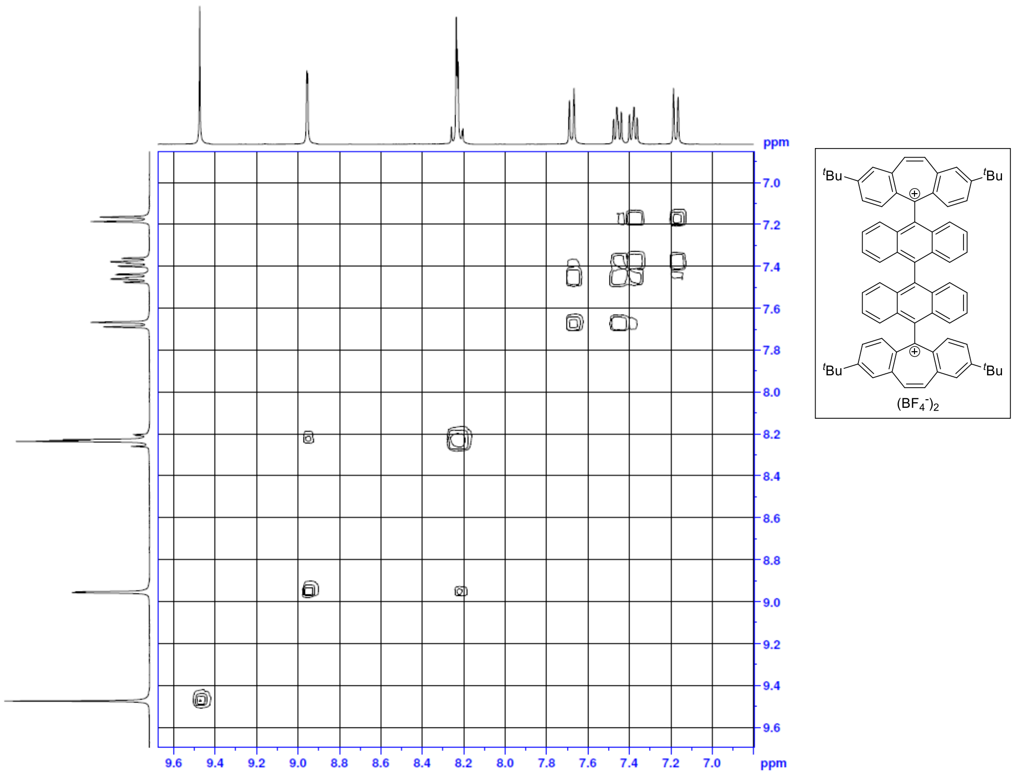

Figure S20. ^{1}H-^{1}H COSY NMR spectra of (a) $\mathbf{1}^{2+}(BF_4^-)_2$ and (b) $\mathbf{2}^{2+}(BF_4^-)_2$ in CD_3CN.

(a)

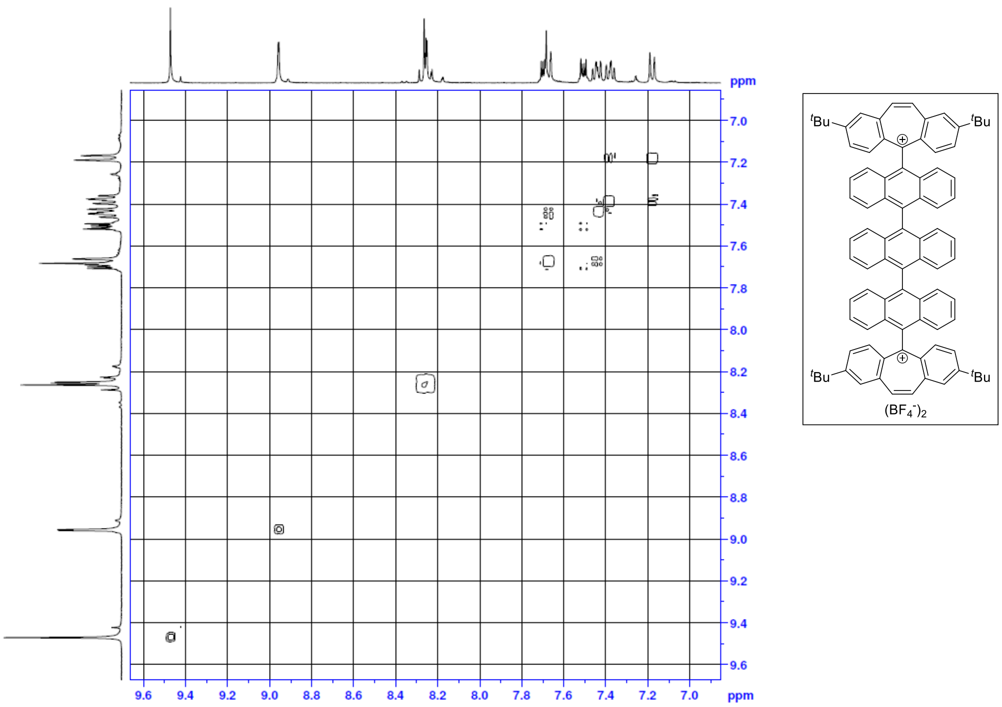

(b)

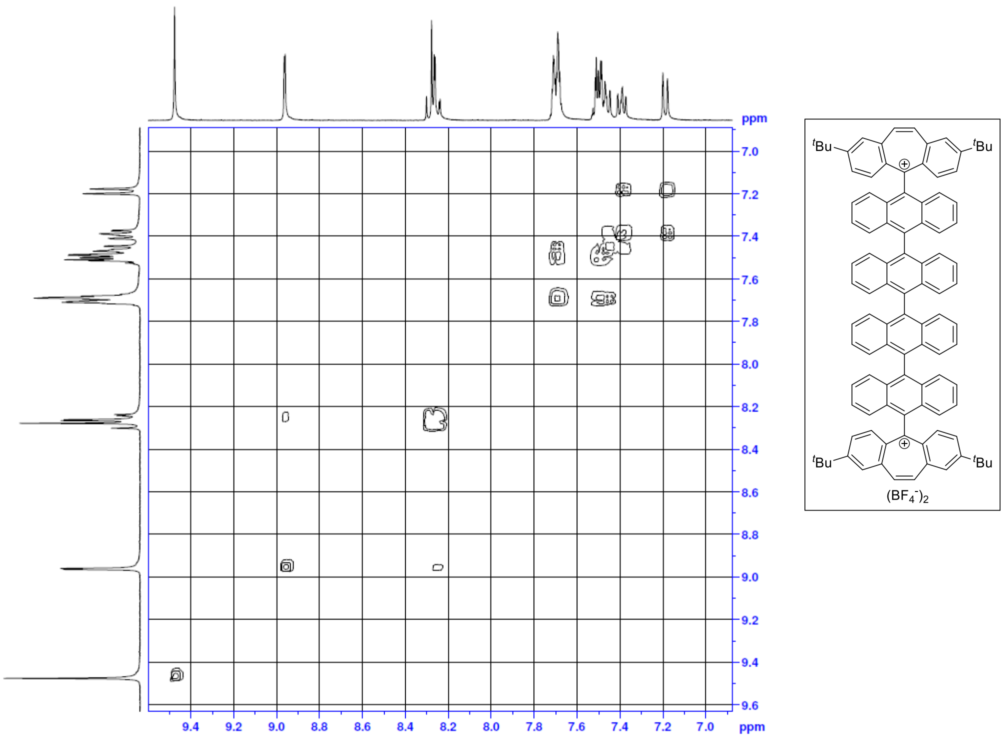

Figure S21. ^{1}H-^{1}H COSY NMR spectra of (a) $\mathbf{3}^{2+}(BF_4^-)_2$ and (b) $\mathbf{4}^{2+}(BF_4^-)_2$ in CD_3CN.

(a)

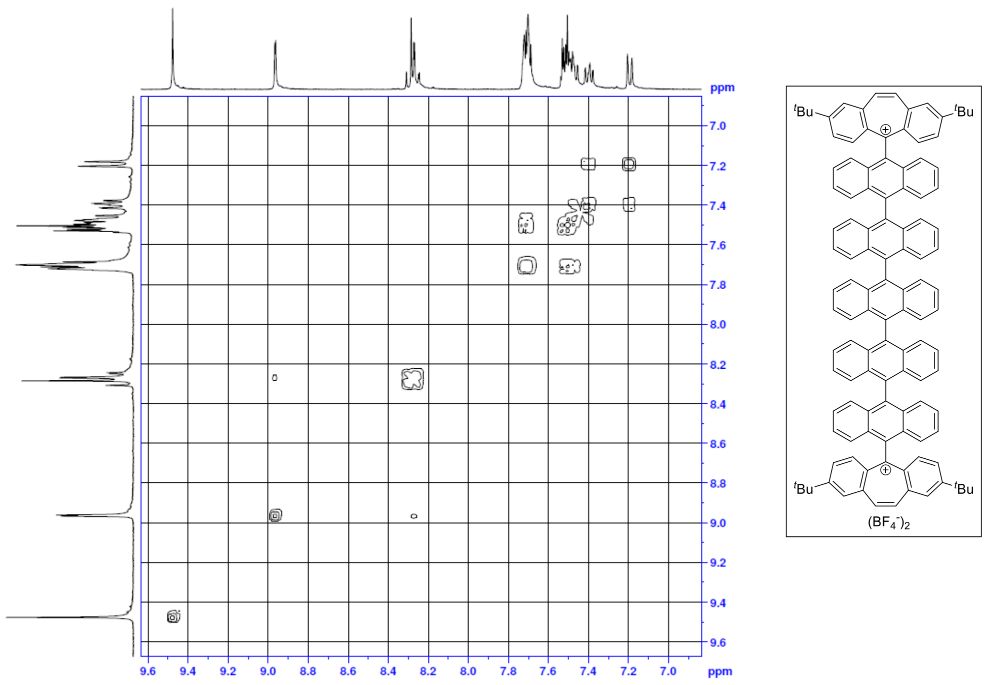

(b)

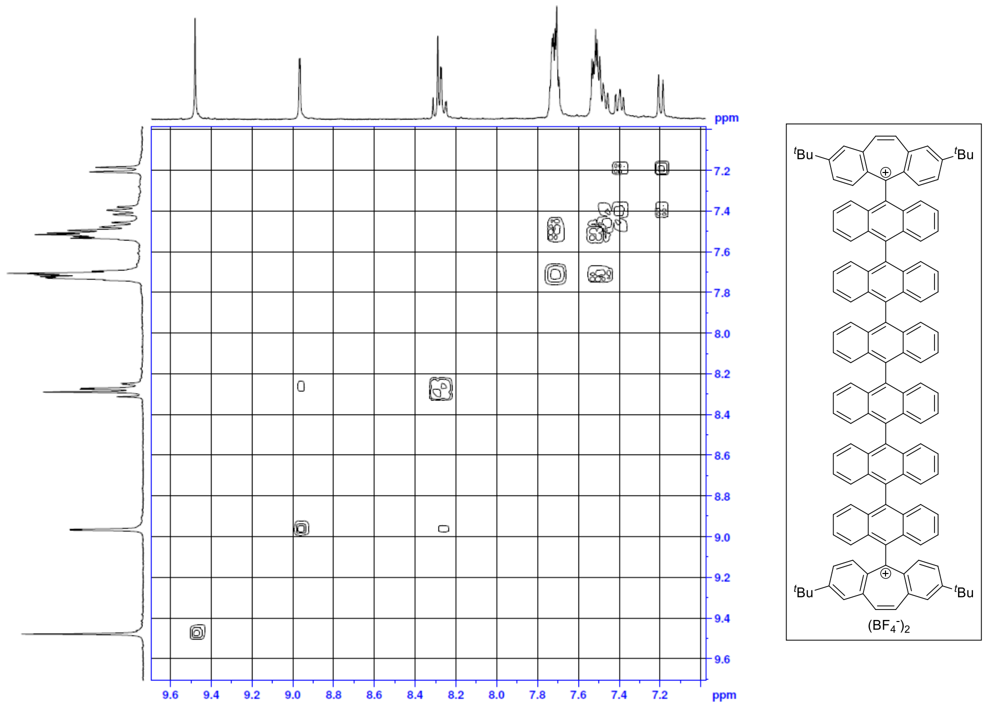

Figure S22. ^{1}H-^{1}H COSY NMR spectra of (a) **5**$^{2+}$(BF_4^-)$_2$ and (b) **6**$^{2+}$(BF_4^-)$_2$ in CD_3CN.

(a)

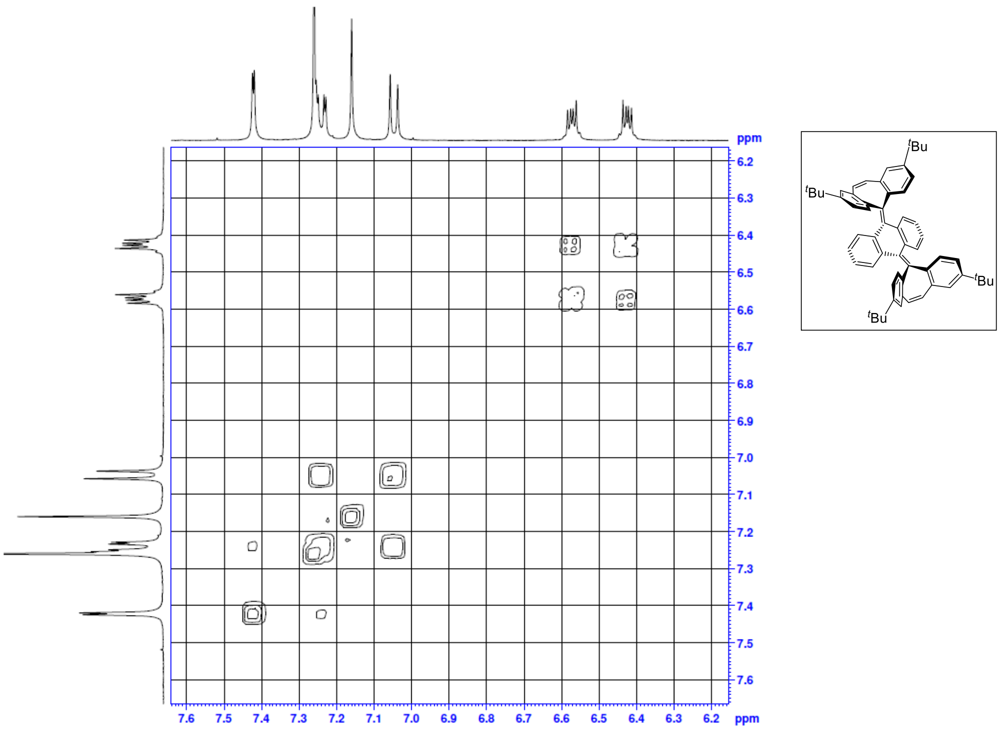

(b)

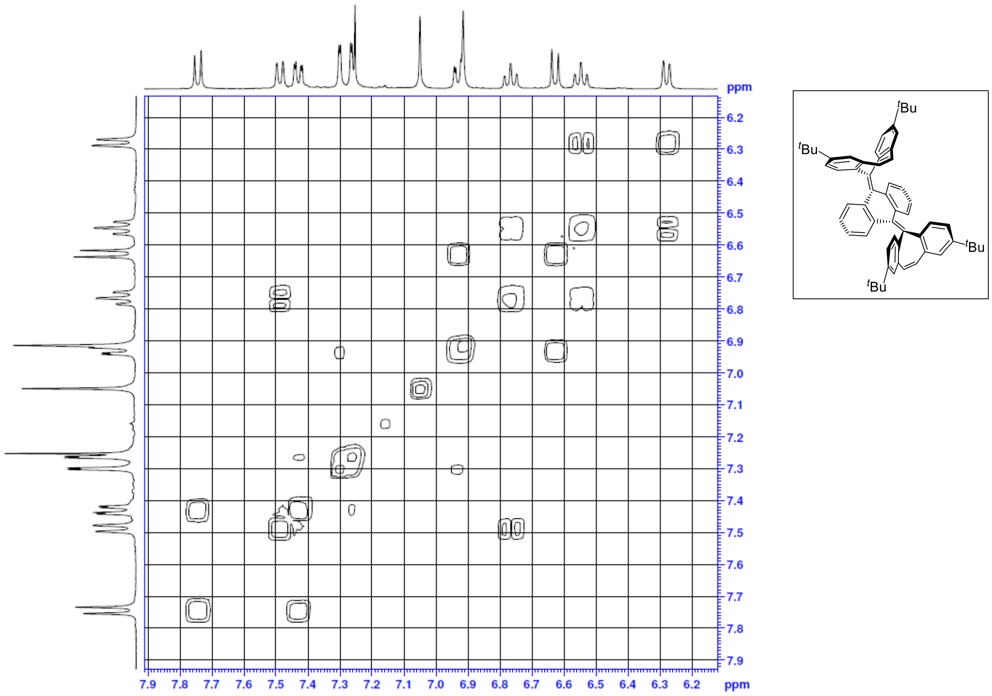

Figure S23. ^{1}H-^{1}H COSY NMR spectra of (a) **1F**$_{anti,anti}$ and (b) **1F**$_{syn,anti}$ in $CDCl_3$.

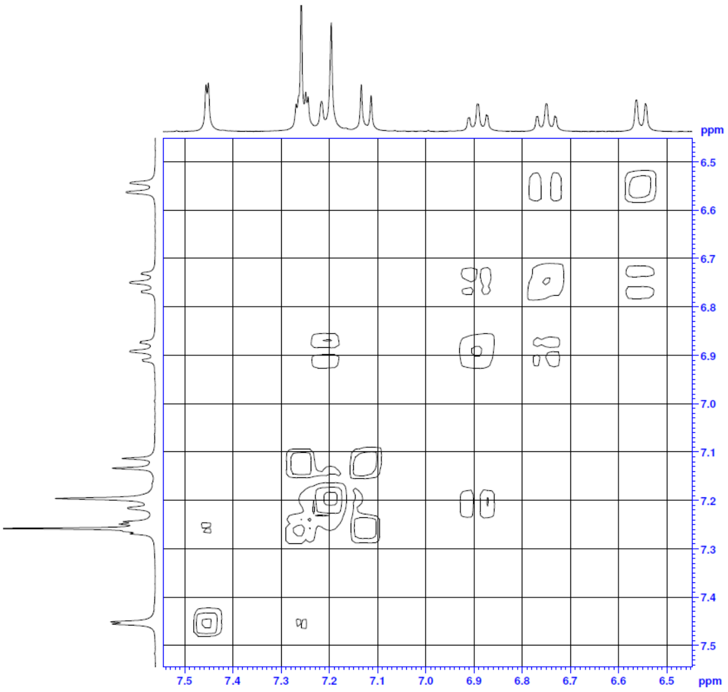

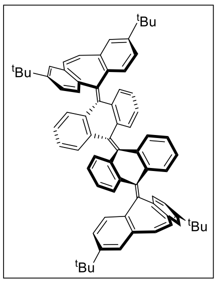

Figure S24. ^{1}H-^{1}H COSY NMR spectra of **2F** in $CDCl_3$.

Theoretical Study (Figures S25-S36, Table S1)

DFT calculations of dications at the CAM-B3LYP/6-31G(d) level

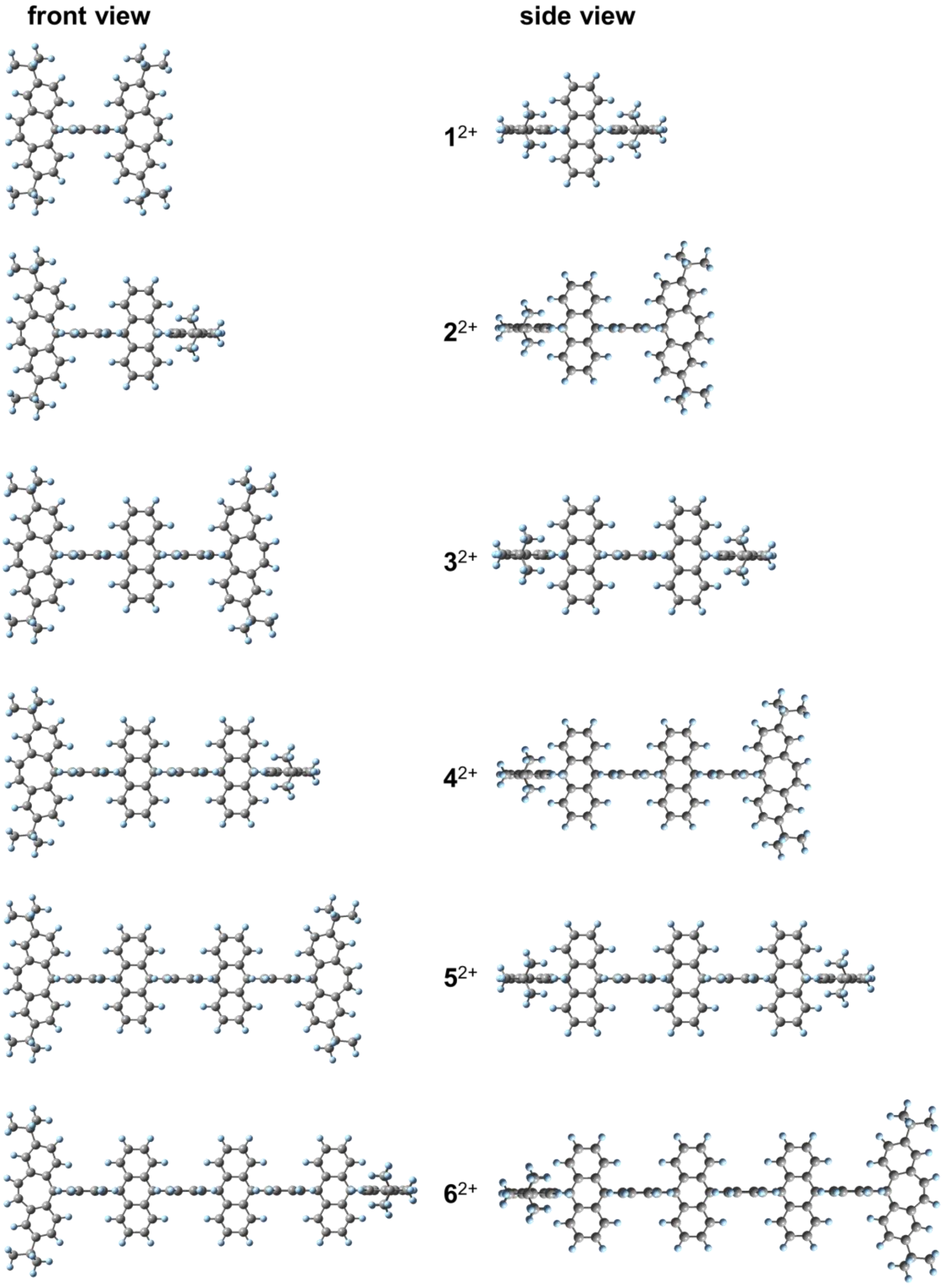

Figure S25. Optimized structures of dications $\mathbf{1}^{2+}$-$\mathbf{6}^{2+}$ based on DFT calculations at the CAM-B3LYP/6-31G(d) level.

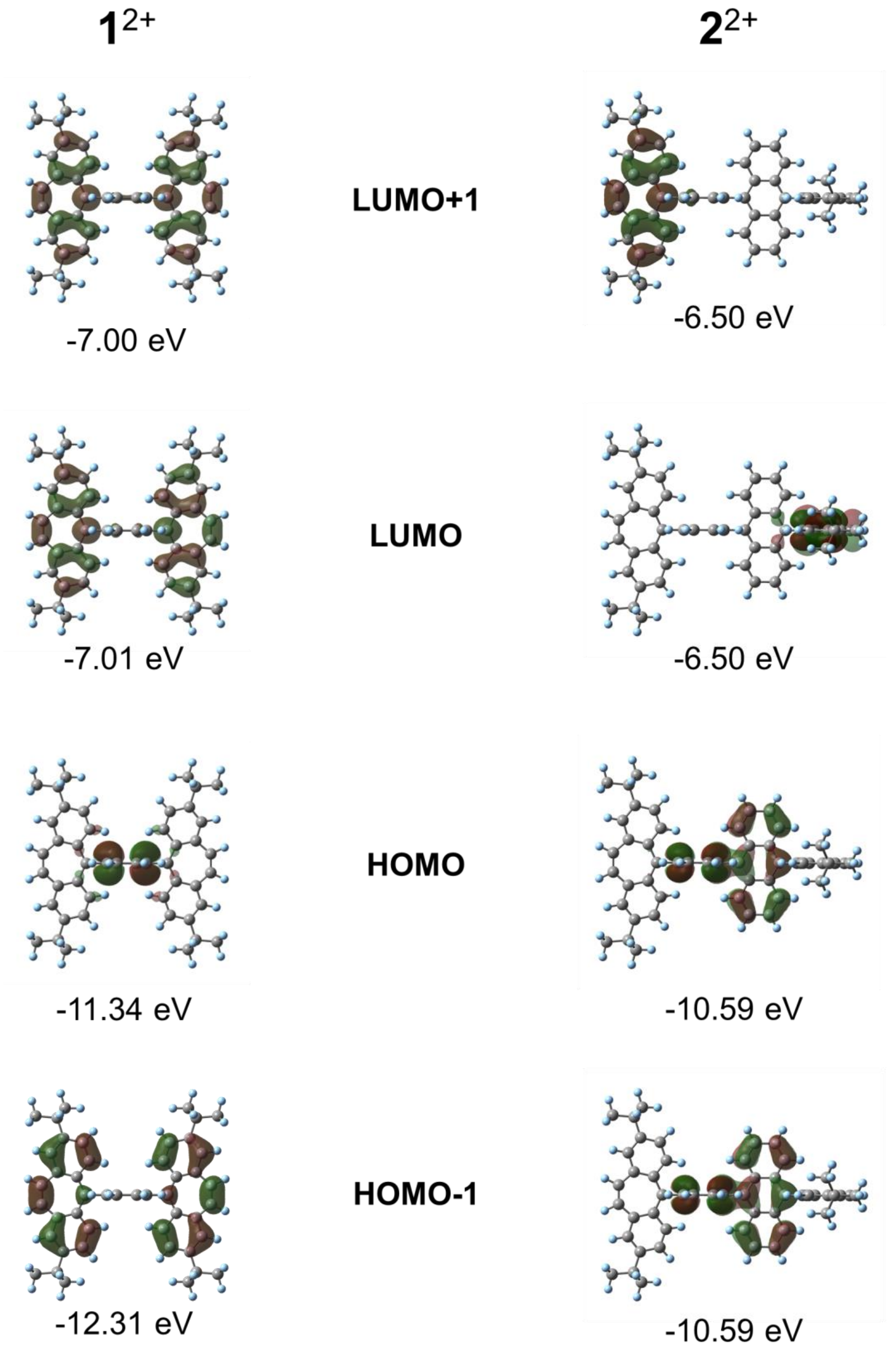

Figure S26. Molecular orbitals of dications $\mathbf{1}^{2+}$ and $\mathbf{2}^{2+}$ based on DFT calculations at the CAM-B3LYP/6-31G(d) level.

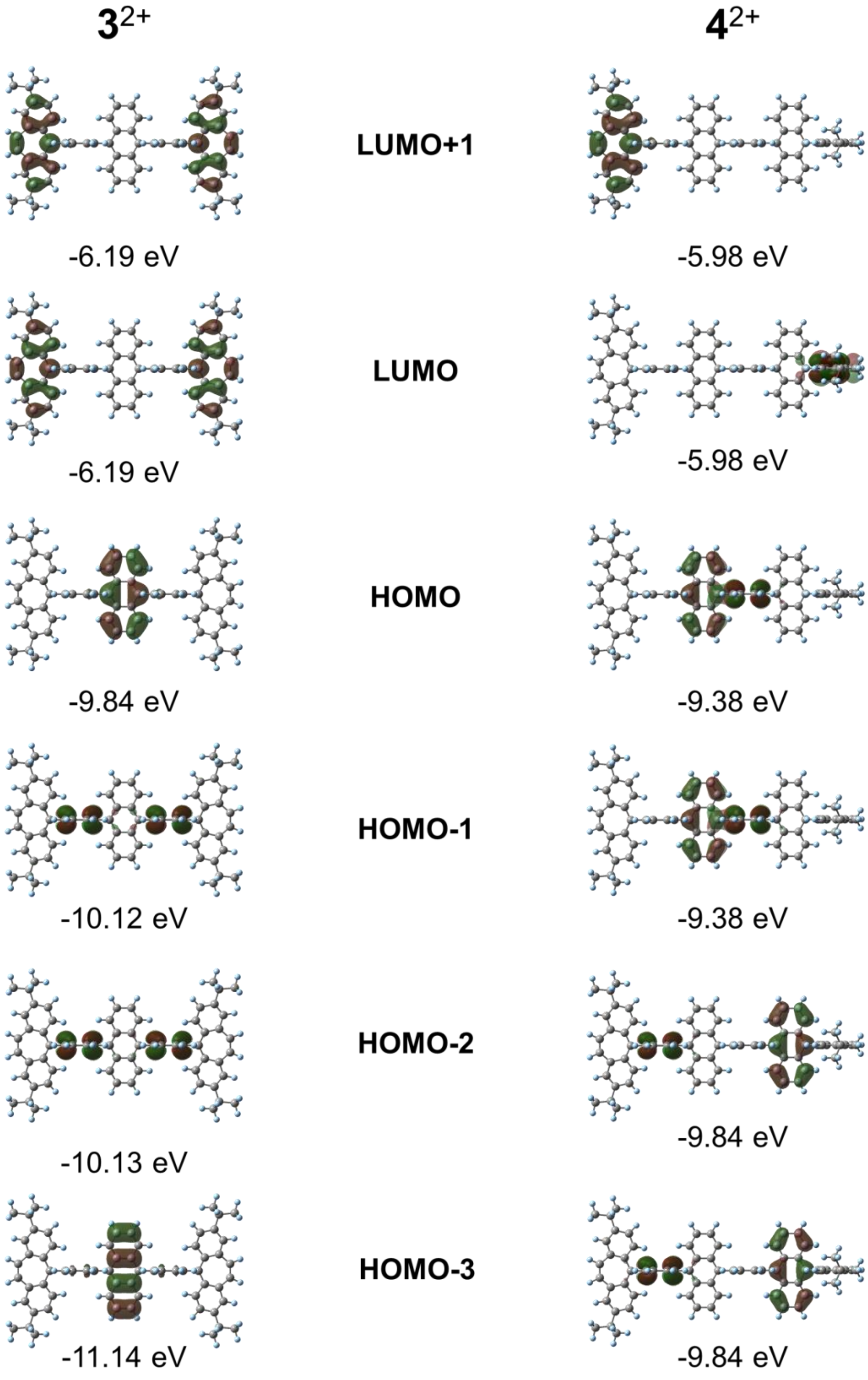

Figure S27. Molecular orbitals of dications $\mathbf{3}^{2+}$ and $\mathbf{4}^{2+}$ based on DFT calculations at the CAM-B3LYP/6-31G(d) level.

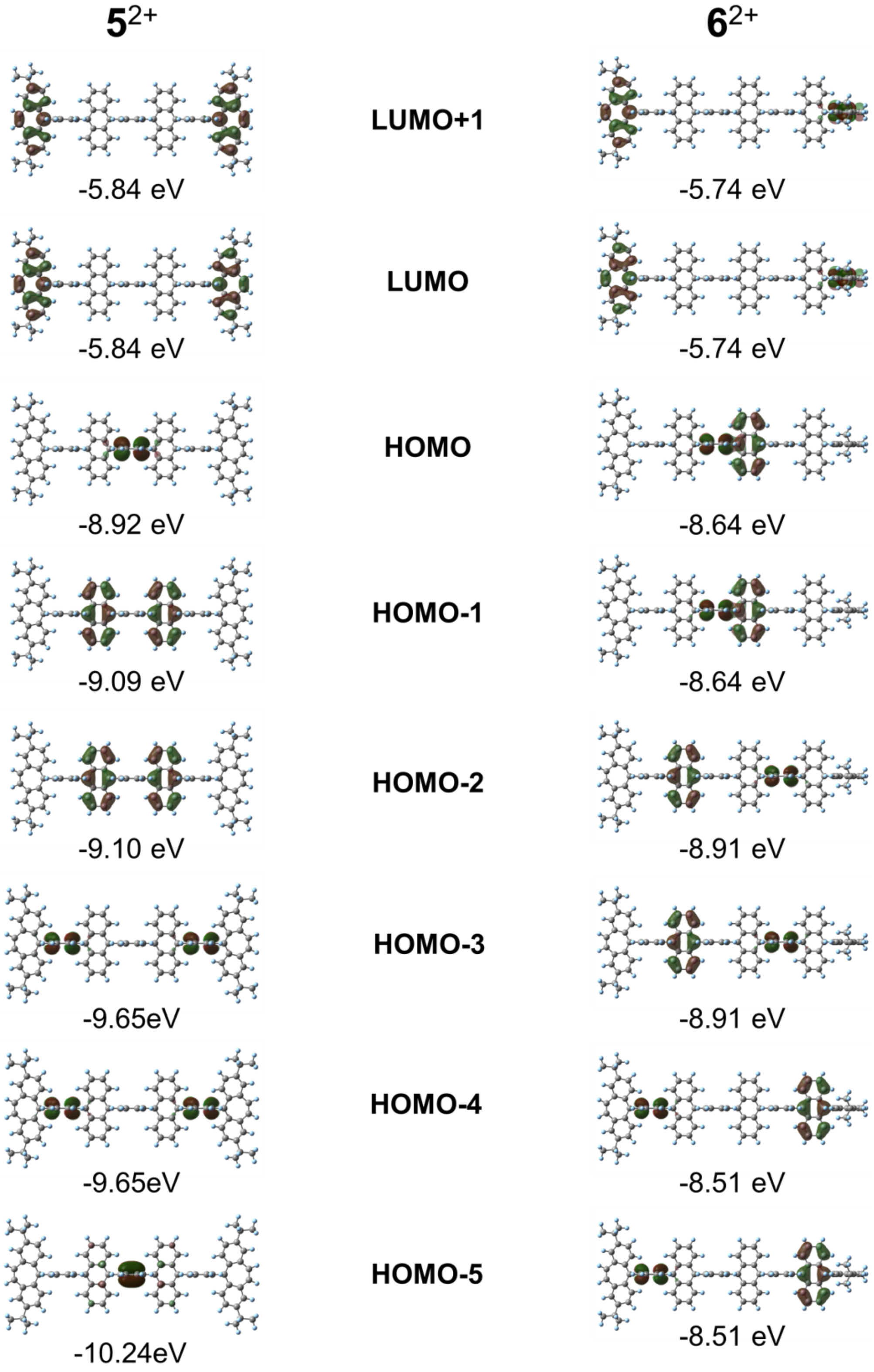

Figure S28. Molecular orbitals of dications $\mathbf{5}^{2+}$ and $\mathbf{6}^{2+}$ based on DFT calculations at the CAM-B3LYP/6-31G(d) level.

TD-DFT calculations of dications at the CAM-B3LYP/6-31G(d) level

1^{2+}
HOMO: 209, LUMO: 210
Excitation energies and oscillator strengths:

```
 Excited State   1:      Singlet-A      2.2003 eV  563.49 nm  f=0.0000  <S**2>=0.000
     209 -> 210         0.69772
 This state for optimization and/or second-order correction.
 Total Energy, E(TD-HF/TD-DFT) =  -2320.59670294
 Copying the excited state density for this state as the 1-particle RhoCI density.

 Excited State   2:      Singlet-A      2.2110 eV  560.77 nm  f=0.0000  <S**2>=0.000
     209 -> 211         0.69776

 Excited State   3:      Singlet-A      2.8027 eV  442.38 nm  f=0.1520  <S**2>=0.000
     207 -> 210         0.49095
     208 -> 211        -0.49006

 Excited State   4:      Singlet-A      2.8226 eV  439.26 nm  f=0.0000  <S**2>=0.000
     207 -> 211        -0.49017
     208 -> 210         0.49309

 Excited State   5:      Singlet-A      3.2720 eV  378.93 nm  f=0.0000  <S**2>=0.000
     204 -> 210        -0.31520
     205 -> 211         0.42910
     206 -> 210        -0.32643
     207 -> 212         0.20948
     208 -> 213         0.20994

 Excited State   6:      Singlet-A      3.2854 eV  377.38 nm  f=0.2795  <S**2>=0.000
     204 -> 211        -0.30598
     205 -> 210         0.42387
     206 -> 211        -0.32268
     207 -> 213        -0.22550
     208 -> 212        -0.22588

 Excited State   7:      Singlet-A      3.6675 eV  338.07 nm  f=0.0000  <S**2>=0.000
     203 -> 211        -0.13299
     204 -> 210        -0.30511
     206 -> 210         0.58087
     207 -> 212         0.12906
     208 -> 213         0.12954

 Excited State   8:      Singlet-A      3.6732 eV  337.54 nm  f=0.0008  <S**2>=0.000
     203 -> 210        -0.13548
     204 -> 211        -0.30334
     206 -> 211         0.58210
     207 -> 213        -0.12791
     208 -> 212        -0.12776

 Excited State   9:      Singlet-A      3.7284 eV  332.54 nm  f=0.0017  <S**2>=0.000
     206 -> 214        -0.11727
     209 -> 212         0.68658

 Excited State  10:      Singlet-A      3.7426 eV  331.28 nm  f=0.0000  <S**2>=0.000
     209 -> 213         0.70220
```

2^{2+}

HOMO: 255, LUMO: 256

Excitation energies and oscillator strengths:

```
 Excited State   1:      Singlet-A      1.9356 eV  640.55 nm  f=0.0000  <S**2>=0.000
      254 -> 256         0.33917
      255 -> 256         0.59276
      255 -> 257        -0.11289
 This state for optimization and/or second-order correction.
 Total Energy, E(TD-HF/TD-DFT) =  -2858.64055102
 Copying the excited state density for this state as the 1-particle RhoCI density.

 Excited State   2:      Singlet-A      1.9356 eV  640.55 nm  f=0.0000  <S**2>=0.000
      254 -> 256         0.13090
      254 -> 257         0.58905
      255 -> 257        -0.34558

 Excited State   3:      Singlet-A      2.8312 eV  437.92 nm  f=0.1826  <S**2>=0.000
      252 -> 256        -0.48026
      253 -> 257         0.48033

 Excited State   4:      Singlet-A      2.8421 eV  436.25 nm  f=0.0000  <S**2>=0.000
      252 -> 256         0.48038
      252 -> 257        -0.10266
      253 -> 256         0.10264
      253 -> 257         0.48031

 Excited State   5:      Singlet-A      2.9773 eV  416.43 nm  f=0.0000  <S**2>=0.000
      254 -> 256         0.47257
      254 -> 257         0.15886
      255 -> 256        -0.15688
      255 -> 257         0.47479

 Excited State   6:      Singlet-A      2.9774 eV  416.42 nm  f=0.0000  <S**2>=0.000
      254 -> 256         0.37393
      254 -> 257        -0.33195
      255 -> 256        -0.33289
      255 -> 257        -0.37111

 Excited State   7:      Singlet-A      3.2550 eV  380.90 nm  f=0.1130  <S**2>=0.000
      247 -> 257        -0.16905
      248 -> 257        -0.28245
      249 -> 257         0.30745
      250 -> 257         0.34691
      251 -> 257         0.27979
      253 -> 258         0.17398
      253 -> 259        -0.17448

 Excited State   8:      Singlet-A      3.2550 eV  380.90 nm  f=0.1130  <S**2>=0.000
      247 -> 256         0.16905
      248 -> 256        -0.28245
      249 -> 256        -0.30746
      250 -> 256         0.34691
      251 -> 256        -0.27979
      252 -> 258        -0.17457
      252 -> 259        -0.17390

 Excited State   9:      Singlet-A      3.4641 eV  357.92 nm  f=0.0006  <S**2>=0.000
      254 -> 258         0.24740
      254 -> 259         0.24912
      255 -> 258         0.42999
      255 -> 259         0.42694

 Excited State  10:      Singlet-A      3.4641 eV  357.92 nm  f=0.0006  <S**2>=0.000
      254 -> 258         0.42856
      254 -> 259        -0.42838
      255 -> 258        -0.24657
      255 -> 259         0.24995
```

3^{2+}
HOMO: 301, LUMO: 302
Excitation energies and oscillator strengths:

Excited State 1: Singlet-A 1.7740 eV 698.91 nm f=0.0000 <S**2>=0.000
299 -> 302 0.27269
299 -> 303 0.40962
300 -> 302 0.40698
300 -> 303 0.28044
This state for optimization and/or second-order correction.
Total Energy, E(TD-HF/TD-DFT) = -3396.67334725
Copying the excited state density for this state as the 1-particle RhoCI density.

Excited State 2: Singlet-A 1.7740 eV 698.91 nm f=0.0000 <S**2>=0.000
299 -> 302 0.40984
299 -> 303 -0.27290
300 -> 302 -0.28023
300 -> 303 0.40678

Excited State 3: Singlet-A 2.5340 eV 489.28 nm f=0.0000 <S**2>=0.000
301 -> 302 0.70621

Excited State 4: Singlet-A 2.5342 eV 489.24 nm f=0.0000 <S**2>=0.000
301 -> 303 0.70621

Excited State 5: Singlet-A 3.1886 eV 388.83 nm f=0.0000 <S**2>=0.000
290 -> 303 -0.11509
291 -> 302 0.15289
292 -> 303 0.19101
293 -> 302 -0.24936
296 -> 303 0.41903
297 -> 302 0.37069

Excited State 6: Singlet-A 3.1895 eV 388.73 nm f=0.1179 <S**2>=0.000
290 -> 302 -0.11343
291 -> 303 0.15130
292 -> 302 0.18959
293 -> 303 -0.24904
296 -> 302 0.41999
297 -> 303 0.37180

Excited State 7: Singlet-A 3.2013 eV 387.29 nm f=0.0000 <S**2>=0.000
299 -> 302 0.36640
299 -> 303 -0.34113
300 -> 302 0.34586
300 -> 303 -0.35989

Excited State 8: Singlet-A 3.2013 eV 387.29 nm f=0.0000 <S**2>=0.000
299 -> 302 0.34131
299 -> 303 0.36665
300 -> 302 -0.35965
300 -> 303 -0.34568

Excited State 9: Singlet-A 3.2984 eV 375.89 nm f=0.0000 <S**2>=0.000
299 -> 305 -0.49327
300 -> 304 0.49652

Excited State 10: Singlet-A 3.2986 eV 375.86 nm f=0.0017 <S**2>=0.000
299 -> 304 -0.49324
300 -> 305 0.49647

4^{2+}
HOMO: 347, LUMO: 348
Excitation energies and oscillator strengths:

```
 Excited State   1:      Singlet-A      1.6890 eV  734.09 nm  f=0.0000  <S**2>=0.000
      345 -> 348         0.69727
 This state for optimization and/or second-order correction.
 Total Energy, E(TD-HF/TD-DFT) =  -3934.69978141
 Copying the excited state density for this state as the 1-particle RhoCI density.

 Excited State   2:      Singlet-A      1.6890 eV  734.09 nm  f=0.0000  <S**2>=0.000
      344 -> 349         0.69727

 Excited State   3:      Singlet-A      2.2797 eV  543.87 nm  f=0.0000  <S**2>=0.000
      346 -> 348         0.40193
      346 -> 349         0.15237
      347 -> 348         0.54831
      347 -> 349        -0.11542

 Excited State   4:      Singlet-A      2.2797 eV  543.87 nm  f=0.0000  <S**2>=0.000
      346 -> 348        -0.11015
      346 -> 349         0.54939
      347 -> 348        -0.15622
      347 -> 349        -0.40045

 Excited State   5:      Singlet-A      2.6611 eV  465.91 nm  f=0.0000  <S**2>=0.000
      346 -> 349         0.41603
      347 -> 349         0.56953

 Excited State   6:      Singlet-A      2.6611 eV  465.91 nm  f=0.0000  <S**2>=0.000
      346 -> 348         0.56927
      347 -> 348        -0.41639

 Excited State   7:      Singlet-A      2.8552 eV  434.24 nm  f=0.2169  <S**2>=0.000
      337 -> 349        -0.48920
      338 -> 348         0.48971

 Excited State   8:      Singlet-A      2.8594 eV  433.60 nm  f=0.0000  <S**2>=0.000
      337 -> 349         0.49083
      338 -> 348         0.49030

 Excited State   9:      Singlet-A      3.1346 eV  395.53 nm  f=0.0378  <S**2>=0.000
      334 -> 348         0.11682
      335 -> 348        -0.13296
      336 -> 348         0.17313
      338 -> 351        -0.11383
      339 -> 348        -0.37766
      340 -> 348         0.42624
      341 -> 348        -0.26253

 Excited State  10:      Singlet-A      3.1346 eV  395.53 nm  f=0.0378  <S**2>=0.000
      334 -> 349         0.11682
      335 -> 349         0.13296
      336 -> 349         0.17313
      337 -> 350         0.11384
      339 -> 349         0.37767
      340 -> 349         0.42624
      341 -> 349         0.26252
```

5^{2+}

HOMO: 393, LUMO: 394

Excitation energies and oscillator strengths:

```
 Excited State   1:      Singlet-A      1.6378 eV  757.03 nm  f=0.0000  <S**2>=0.000
     389 -> 394        -0.45580
     389 -> 395        -0.16879
     390 -> 394         0.47006
     390 -> 395         0.17052
 This state for optimization and/or second-order correction.
 Total Energy, E(TD-HF/TD-DFT) =  -4472.72264070
 Copying the excited state density for this state as the 1-particle RhoCI density.

 Excited State   2:      Singlet-A      1.6378 eV  757.03 nm  f=0.0000  <S**2>=0.000
     389 -> 394        -0.17355
     389 -> 395         0.46894
     390 -> 394        -0.16567
     390 -> 395         0.45696

 Excited State   3:      Singlet-A      2.1383 eV  579.81 nm  f=0.0000  <S**2>=0.000
     391 -> 394         0.46963
     391 -> 395         0.16205
     392 -> 394         0.46793
     392 -> 395         0.18159

 Excited State   4:      Singlet-A      2.1383 eV  579.81 nm  f=0.0000  <S**2>=0.000
     391 -> 394        -0.16212
     391 -> 395         0.46981
     392 -> 394         0.18153
     392 -> 395        -0.46775

 Excited State   5:      Singlet-A      2.3468 eV  528.30 nm  f=0.0000  <S**2>=0.000
     393 -> 394         0.70384

 Excited State   6:      Singlet-A      2.3468 eV  528.30 nm  f=0.0000  <S**2>=0.000
     393 -> 395         0.70384

 Excited State   7:      Singlet-A      2.7064 eV  458.12 nm  f=0.0000  <S**2>=0.000
     391 -> 394        -0.17502
     391 -> 395         0.47084
     392 -> 394        -0.16903
     392 -> 395         0.46801

 Excited State   8:      Singlet-A      2.7064 eV  458.12 nm  f=0.0000  <S**2>=0.000
     391 -> 394         0.47102
     391 -> 395         0.17508
     392 -> 394        -0.46783
     392 -> 395        -0.16896

 Excited State   9:      Singlet-A      3.1587 eV  392.51 nm  f=0.0000  <S**2>=0.000
     389 -> 396         0.36985
     389 -> 397         0.33160
     390 -> 396         0.36040
     390 -> 397        -0.34017

 Excited State  10:      Singlet-A      3.1587 eV  392.51 nm  f=0.0016  <S**2>=0.000
     389 -> 396         0.34110
     389 -> 397        -0.35949
     390 -> 396         0.33063
     390 -> 397         0.37073
```

6^{2+}

HOMO: 439, LUMO: 440

Excitation energies and oscillator strengths:

```
 Excited State   1:      Singlet-B      1.5702 eV  789.61 nm  f=0.0000  <S**2>=0.000
      434 -> 441         0.49311
      435 -> 440         0.49311
 This state for optimization and/or second-order correction.
 Total Energy, E(TD-HF/TD-DFT) =  -5010.73853593
 Copying the excited state density for this state as the 1-particle RhoCI density.

 Excited State   2:      Singlet-A      1.5702 eV  789.61 nm  f=0.0000  <S**2>=0.000
      434 -> 440         0.49311
      435 -> 441         0.49311

 Excited State   3:      Singlet-B      2.0148 eV  615.36 nm  f=0.0000  <S**2>=0.000
      436 -> 441        -0.49930
      437 -> 440         0.49930

 Excited State   4:      Singlet-A      2.0148 eV  615.36 nm  f=0.0000  <S**2>=0.000
      436 -> 440        -0.49930
      437 -> 441         0.49930

 Excited State   5:      Singlet-A      2.1305 eV  581.95 nm  f=0.0000  <S**2>=0.000
      438 -> 440         0.49984
      439 -> 441         0.49989

 Excited State   6:      Singlet-B      2.1305 eV  581.95 nm  f=0.0000  <S**2>=0.000
      438 -> 441         0.49984
      439 -> 440         0.49989

 Excited State   7:      Singlet-A      2.3158 eV  535.40 nm  f=0.0000  <S**2>=0.000
      438 -> 440         0.49991
      439 -> 441        -0.49986

 Excited State   8:      Singlet-B      2.3158 eV  535.40 nm  f=0.0000  <S**2>=0.000
      438 -> 441         0.49991
      439 -> 440        -0.49986

 Excited State   9:      Singlet-A      2.6908 eV  460.78 nm  f=0.0000  <S**2>=0.000
      436 -> 440         0.49998
      437 -> 441         0.49998

 Excited State  10:      Singlet-B      2.6908 eV  460.78 nm  f=0.0000  <S**2>=0.000
      436 -> 441         0.49998
      437 -> 440         0.49998
```

DFT calculations of neutral species at the (U)B3LYP/6-31G(d) level

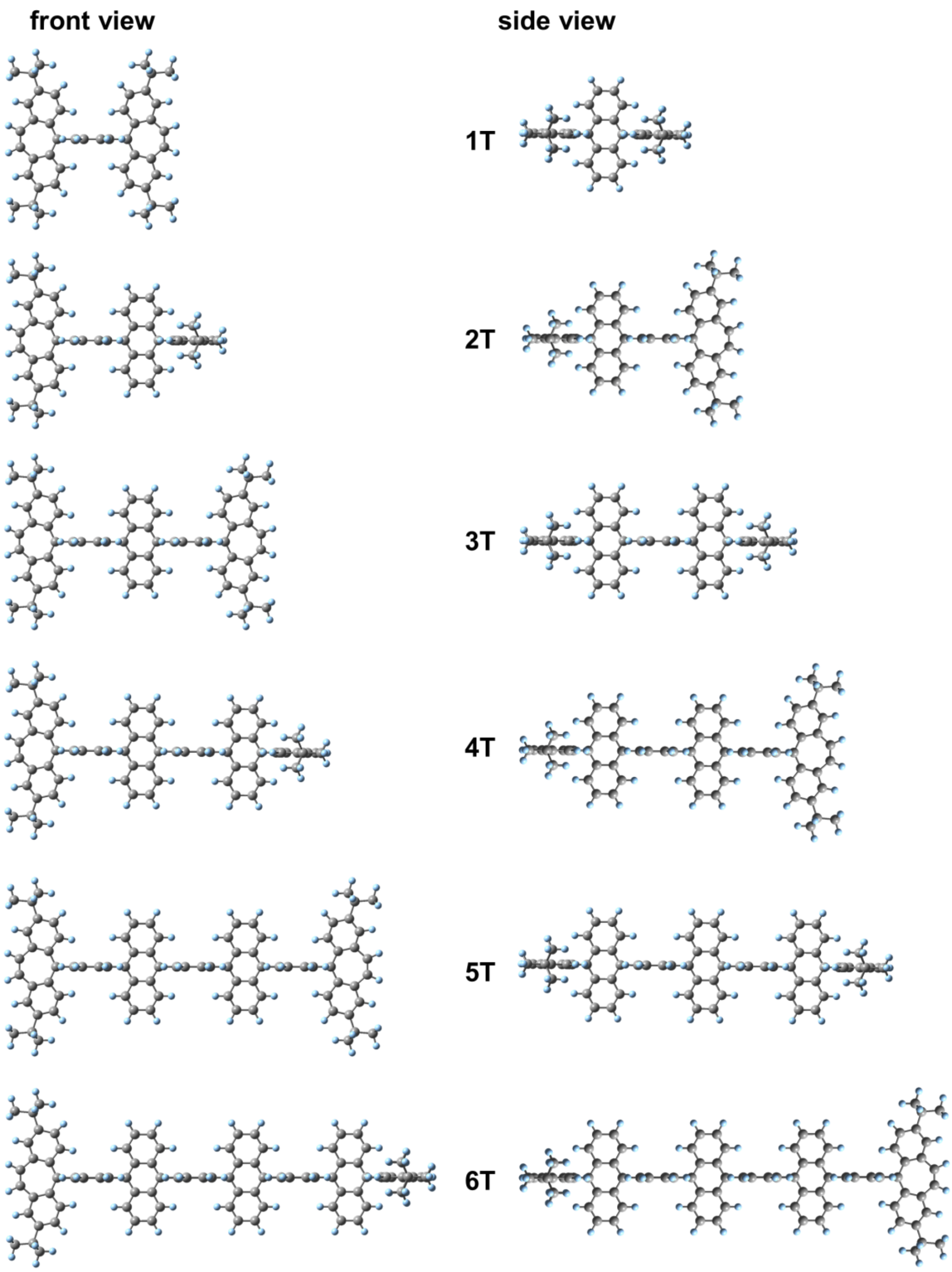

Figure S29. Optimized structures of biradicals **1T**-**6T** (triplet) based on DFT calculations at the UB3LYP/6-31G(d) level.

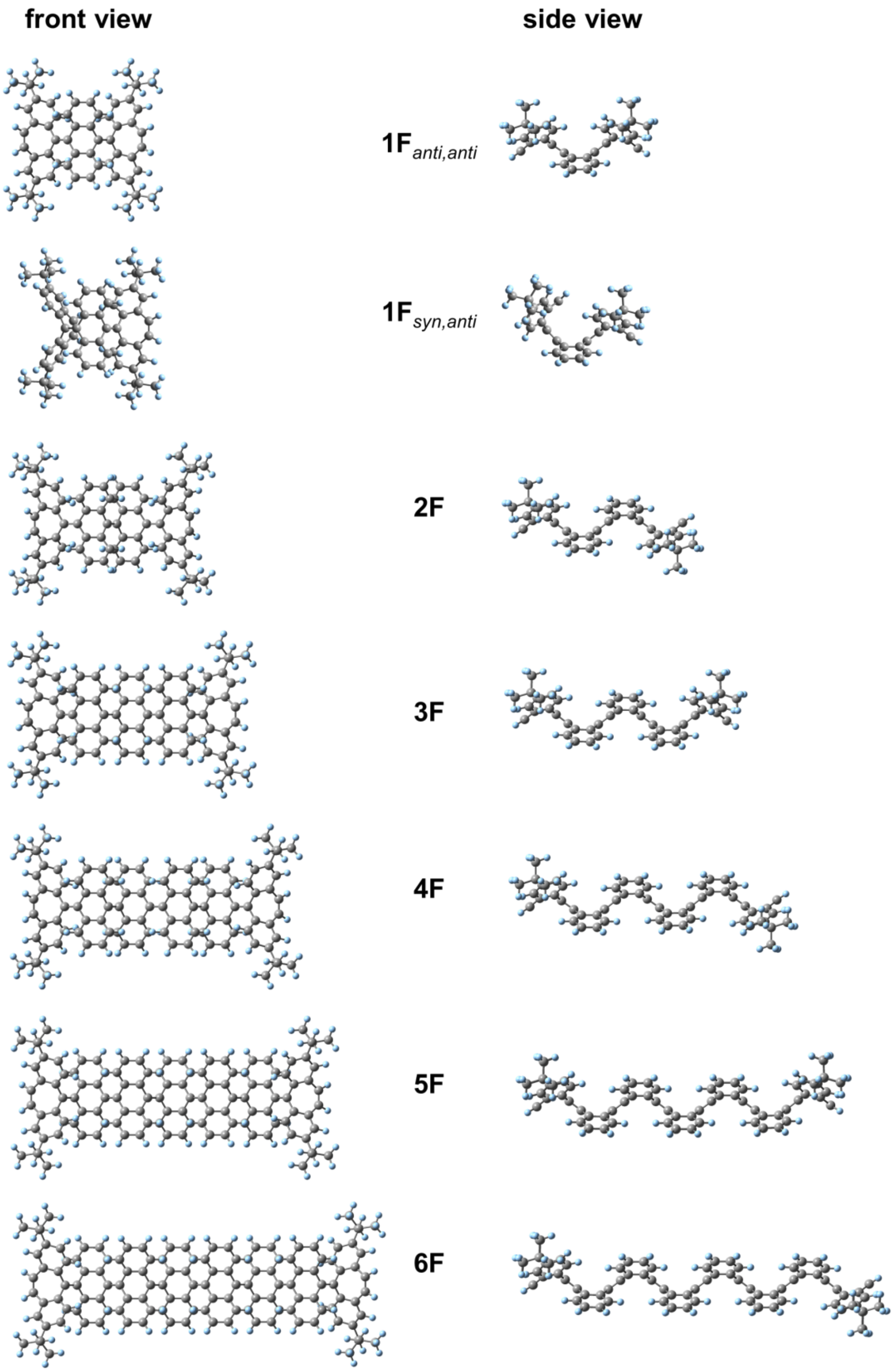

Figure S30. Optimized structures of closed-shell folded forms **1F**-**6F** based on DFT calculations at the B3LYP/6-31G(d) level.

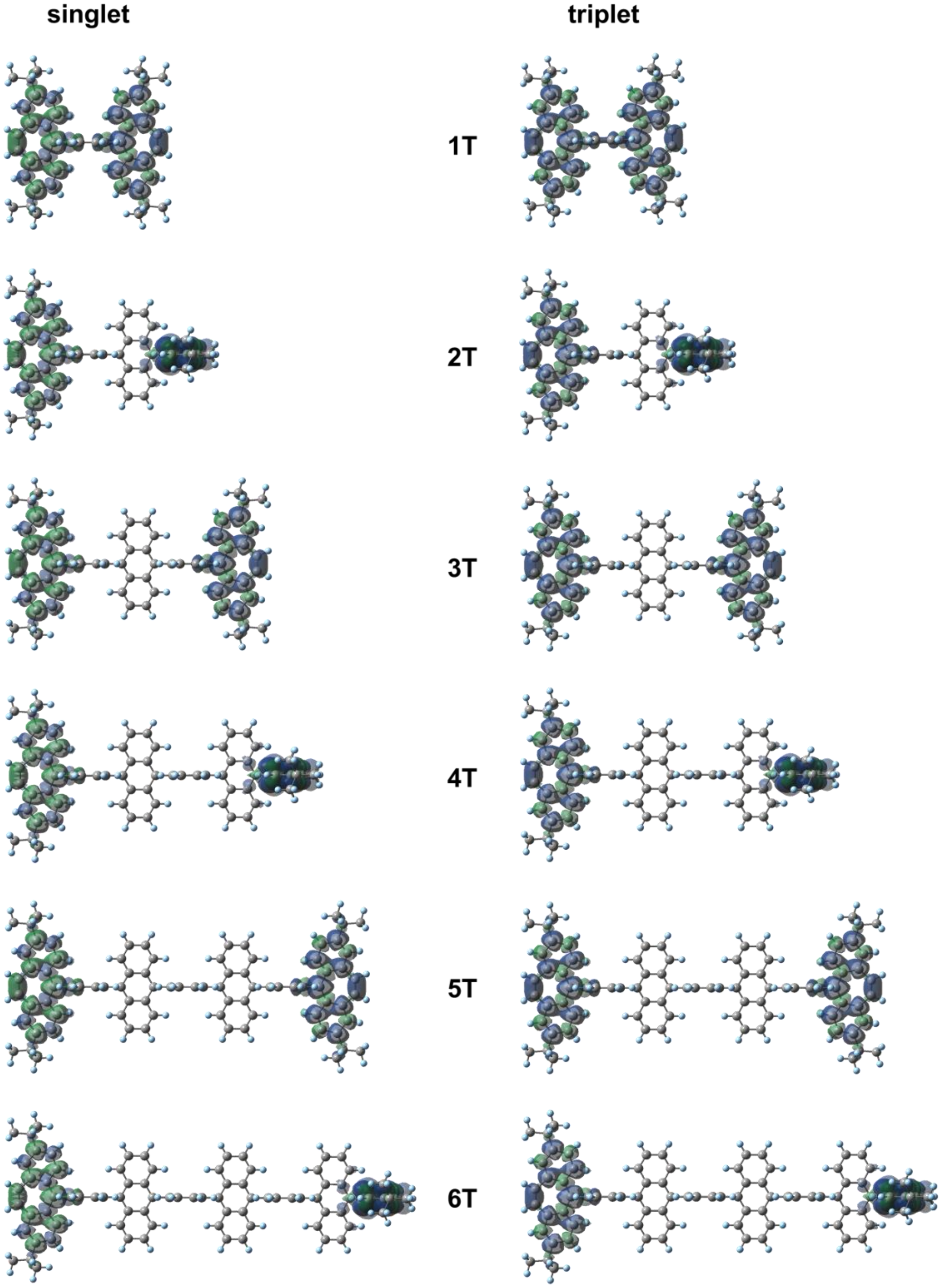

Figure S31. Spin density map of biradicals **1T-6T** based on DFT calculations at the UB3LYP/6-31G(d) level (left: singlet, right: triplet).

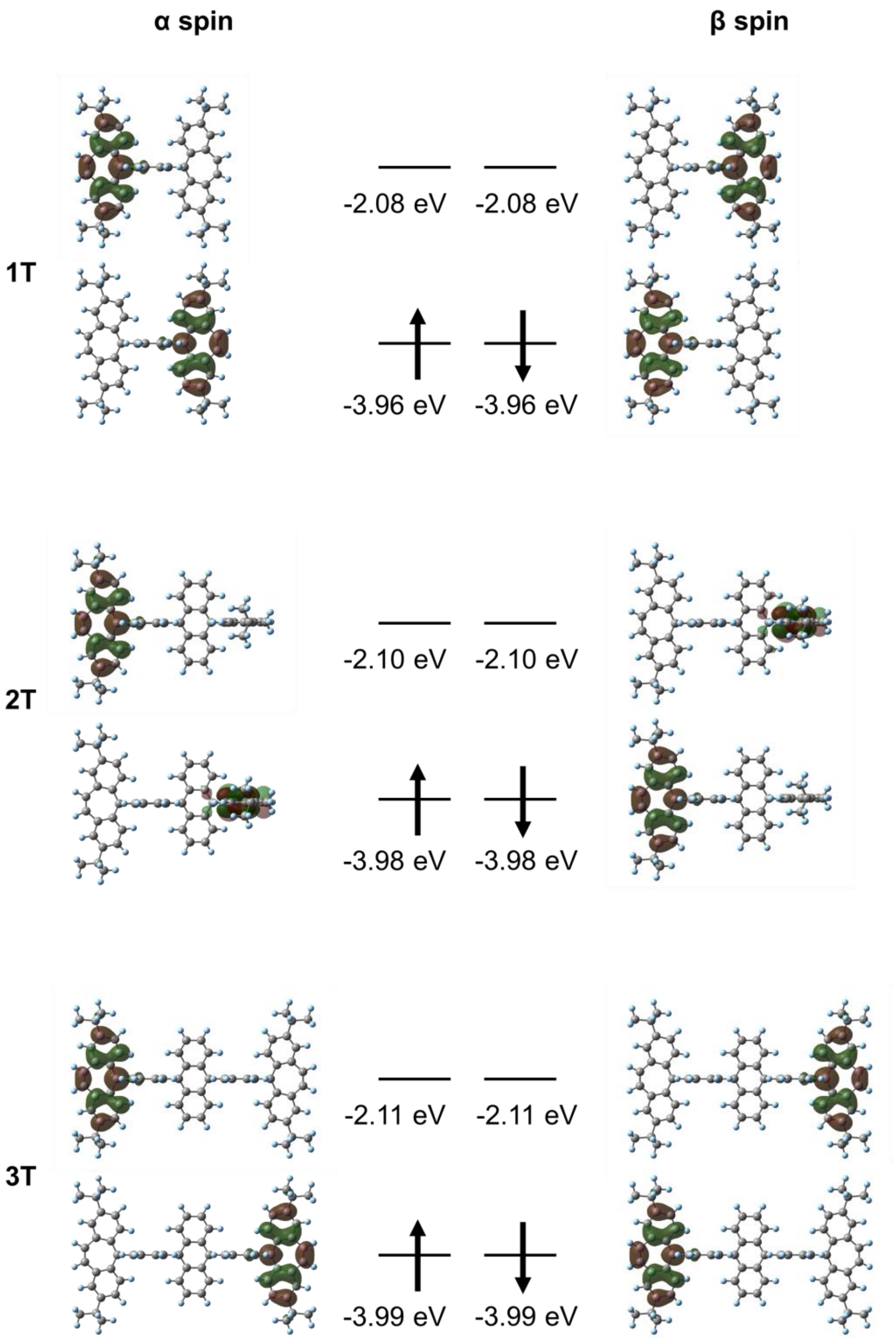

Figure S32. Molecular orbitals of singlet biradicals **1T**, **2T** and **3T** based on DFT calculations at the UB3LYP/6-31G(d) level.

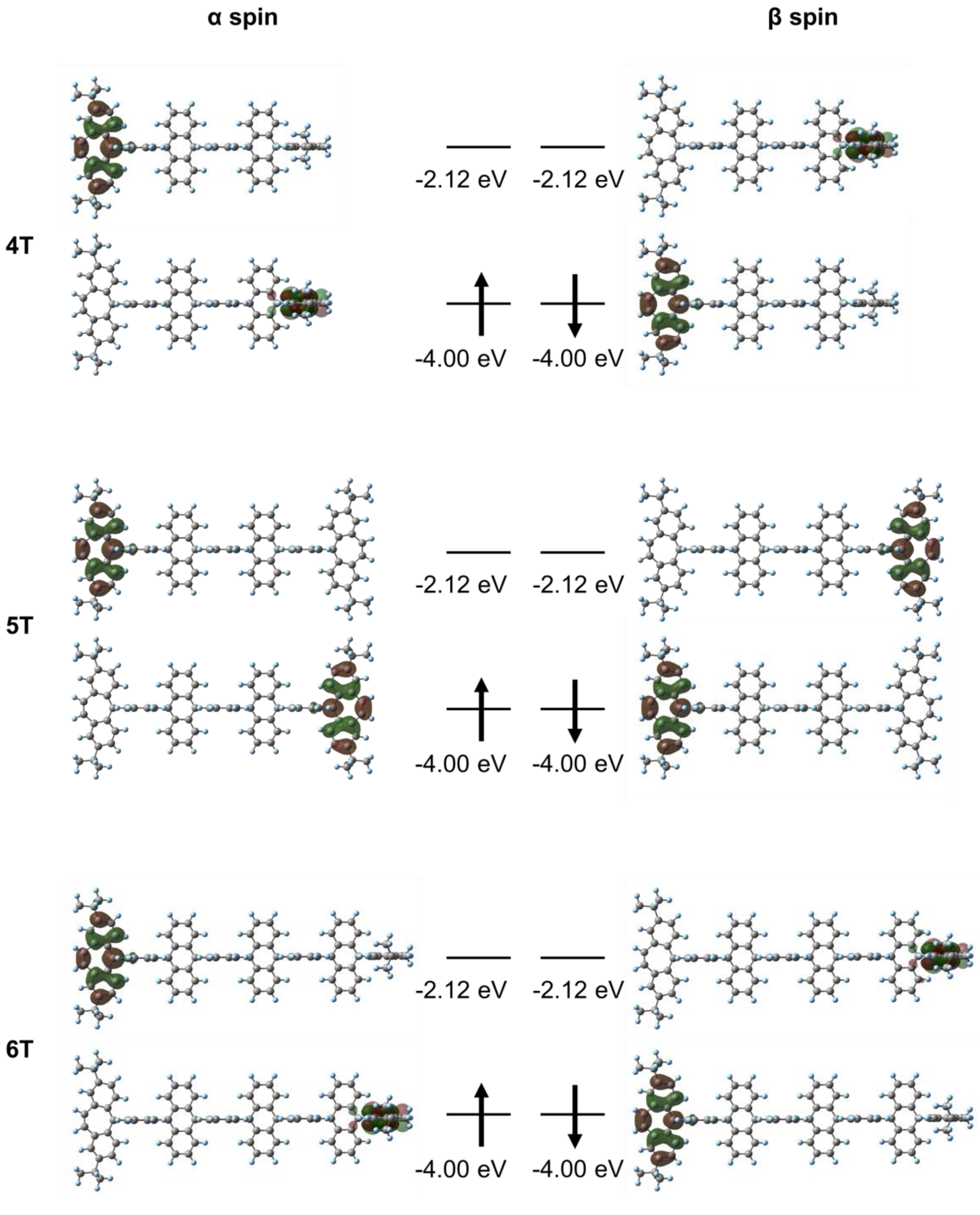

Figure S33. Molecular orbitals of singlet biradicals **4T**, **5T** and **6T** based on DFT calculations at the UB3LYP/6-31G(d) level.

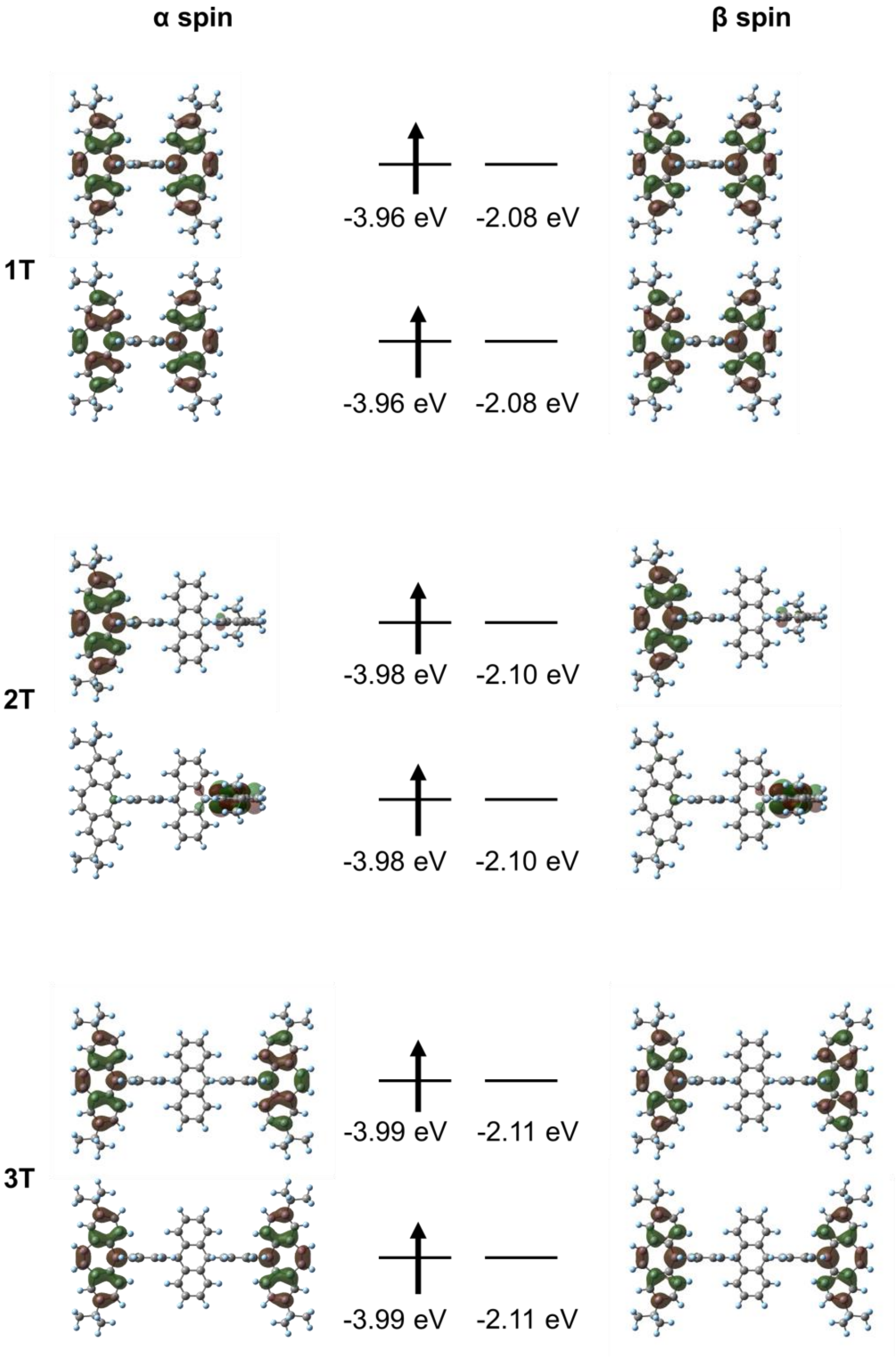

Figure S34. Molecular orbitals of triplet biradicals **1T**, **2T** and **3T** based on DFT calculations at the UB3LYP/6-31G(d) level.

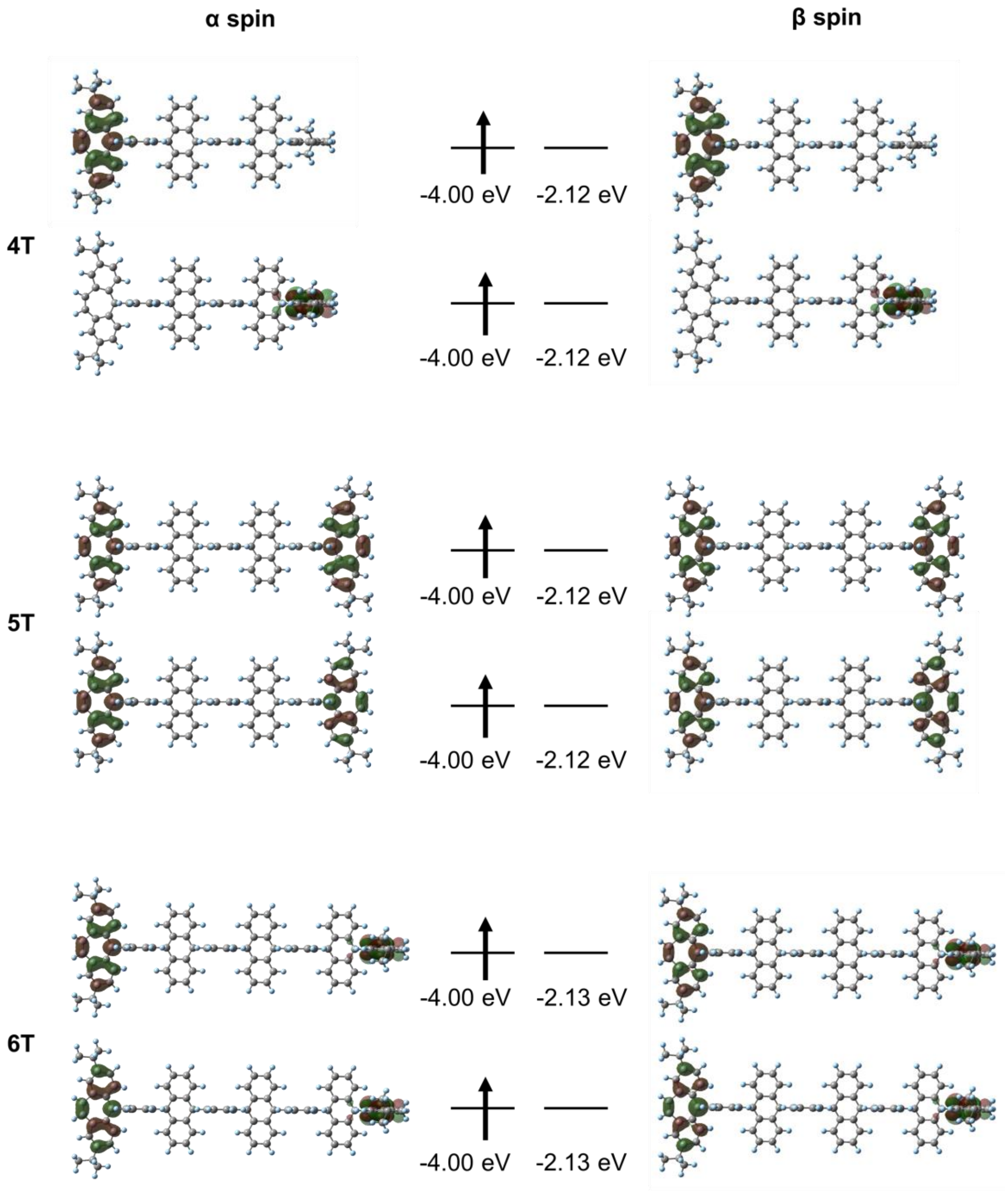

Figure S35. Molecular orbitals of triplet biradicals **4T**, **5T** and **6T** based on DFT calculations at the UB3LYP/6-31G(d) level.

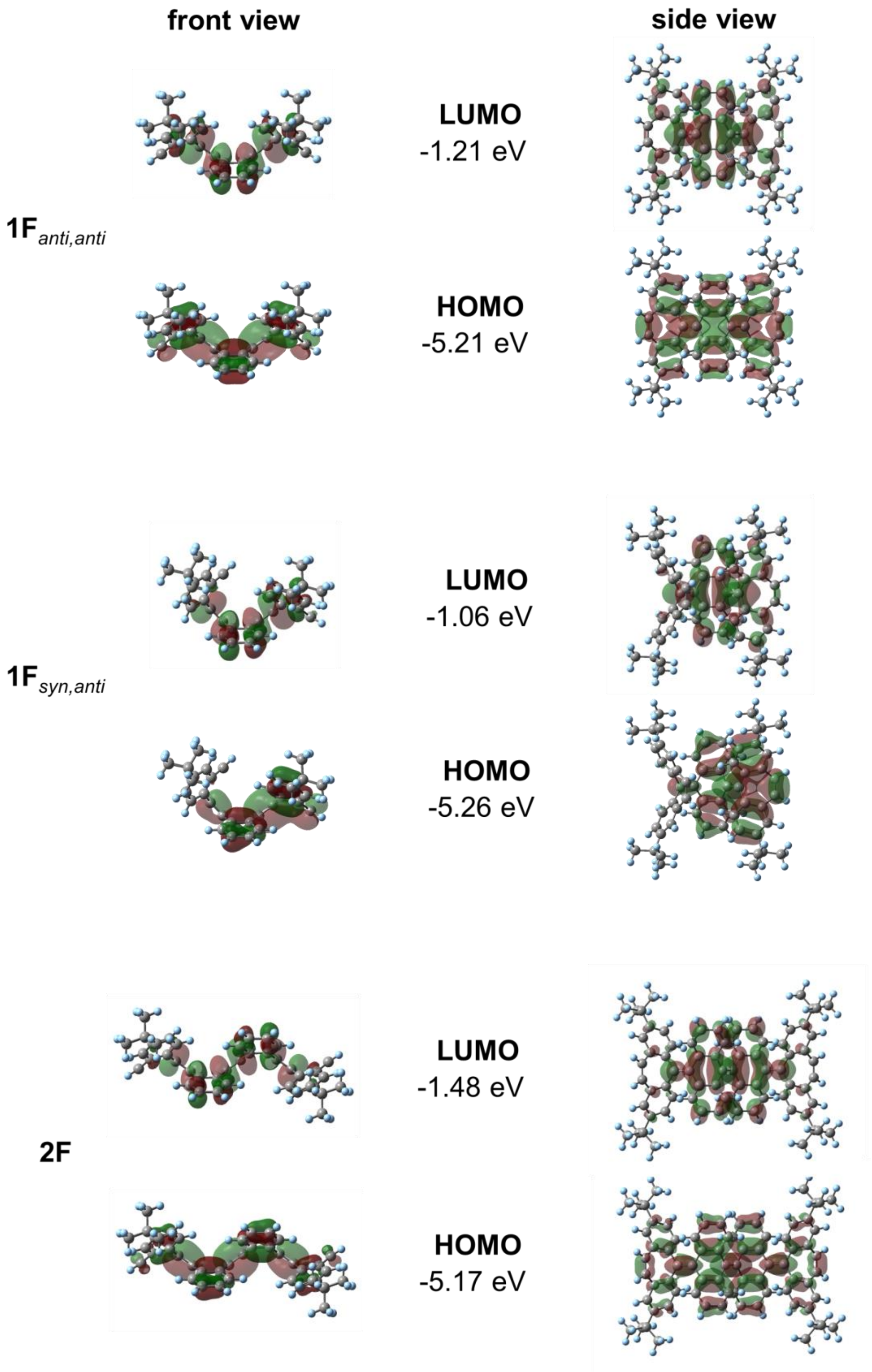

Figure S36. Molecular orbitals of closed-shell folded forms **1F**$_{anti,anti}$, **1F**$_{syn,anti}$ and **2F** based on DFT calculations at the B3LYP/6-31G(d) level.

Table S1. The relative energies of possible isomers for neutral species based on DFT calculations at the (U)B3LYP/6-31G(d) level (0 kcal/mol for open-shell triplet **T**-form). For **2F**-**6F**, the most favorable all-*anti*-type folded forms among the configurational isomers were chosen to be calculated.

	**1T(triplet)**	**1T(singlet)**	**1F*anti,anti***	**1F*syn,anti***
**Δ*E* (kcal/mol)**	0.00	0.00	-21.22	-16.34

	**2T(triplet)**	**2T(singlet)**	**2F**
**Δ*E* (kcal/mol)**	0.00	0.00	-8.72

	**3T(triplet)**	**3T(singlet)**	**3F**
**Δ*E* (kcal/mol)**	0.00	0.00	3.93

	**4T(triplet)**	**4T(singlet)**	**4F**
**Δ*E* (kcal/mol)**	0.00	0.00	16.63

	**5T(triplet)**	**5T(singlet)**	**5F**
**Δ*E* (kcal/mol)**	0.00	0.00	29.29

	**6T(triplet)**	**6T(singlet)**	**6F**
**Δ*E* (kcal/mol)**	0.00	0.00	42.02

Single-Crystal X-ray Structure Analyses (Tables S2 and S3, Figures S37 and S38)

Table S2. The dihedral angles between adjacent 14π-electron units determined by X-ray analyses based on the mean planes defined by the 14 (anthrylene) or 15 (dibenzotropylium) carbon atoms that compose each aromatic unit.

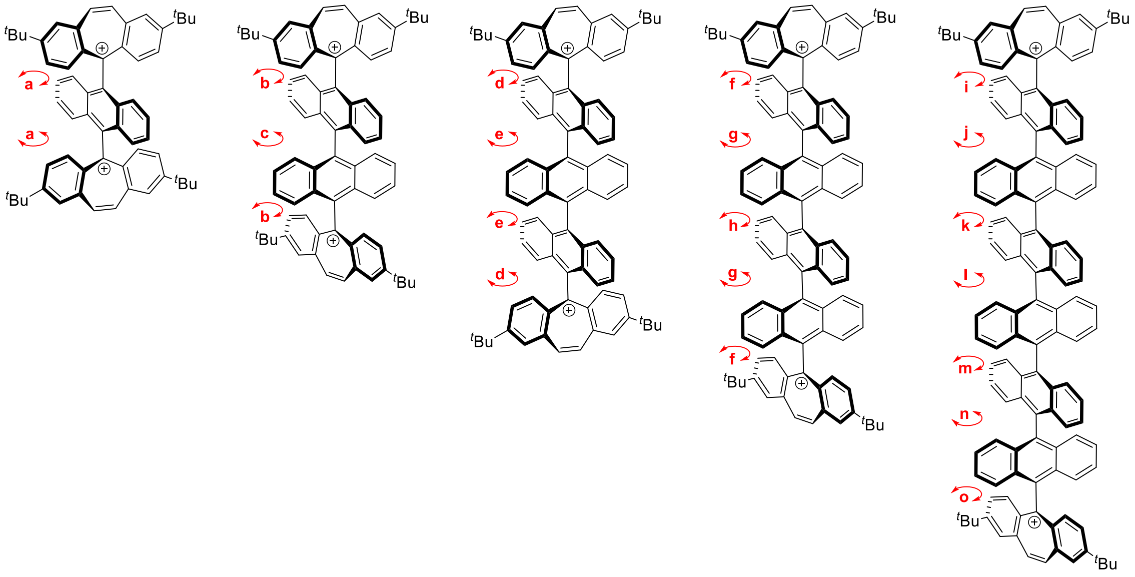

$\mathbf{1}^{2+}(BF_4^-)_2$	$\mathbf{2}^{2+}(PF_6^-)_2$		$\mathbf{3}^{2+}(BF_4^-)_2$		$\mathbf{4}^{2+}(BF_4^-)_2$		
a	b	c	d	e	f	g	h
83.89(3)°	81.04(2)°	78.23(3)°	88.52(5)°	88.63(5)°	89.20(5)°	88.51(5)°	90°

$\mathbf{6}^{2+}(NTf_2^-)_2$						
i	j	k	l	m	n	o
89.91(4)°	89.95(4)°	84.59(4)°	82.79(4)°	83.75(5)°	82.85(5)°	89.45(5)°

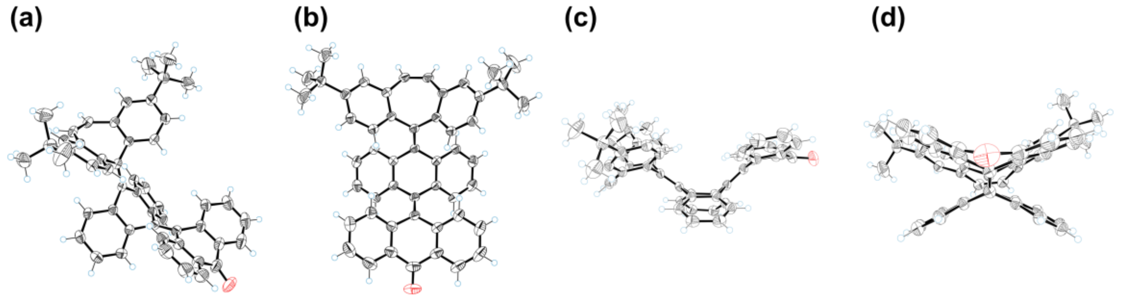

Figure S37. ORTEP drawings of **S7**: (a) best view, (b) front view, (c) side view and (d) top view. Solvent molecule is omitted for clarity. Thermal ellipsoids are shown at the 50% probability level.

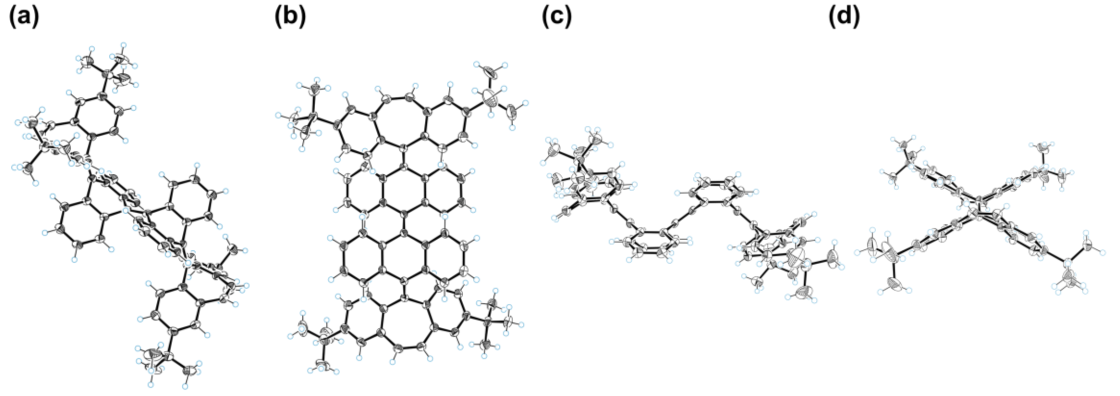

Figure S38. ORTEP drawings of **2F**: (a) best view, (b) front view, (c) side view and (d) top view. Solvent molecule is omitted for clarity. Thermal ellipsoids are shown at the 50% probability level.

Crystal data

Table S3. Crystal data of **S7**, $\mathbf{1}^{2+}(BF_4^-)_2$, $\mathbf{2}^{2+}(PF_6^-)_2$, $\mathbf{3}^{2+}(BF_4^-)_2$, $\mathbf{4}^{2+}(BF_4^-)_2$, $\mathbf{6}^{2+}(NTf_2^-)_2$ and **2F**.

	**S7**	$\mathbf{1}^{2+}(BF_4^-)_2$	$\mathbf{2}^{2+}(PF_6^-)_2$	$\mathbf{3}^{2+}(BF_4^-)_2$	$\mathbf{4}^{2+}(BF_4^-)_2$	$\mathbf{6}^{2+}(NTf_2^-)_2$	**2F**
**Recrystallization solvent**	$CHCl_3$/hexane	CH_3NO_2/ether	CH_3NO_2/ether	CH_3CN/ether	CH_3CN/ether	CH_3CN/ether	CH_2Cl_2/hexane
**Color and shape**	Yellow plate	Red plate	Red block	Red block	Red block	Red needle	Colourless needle
**Empirical formula**	$C_{54}H_{49}O$	$C_{62}H_{66}N_2O_4B_2F_8$	$C_{82}H_{88}O_2F_{12}P_2$	$C_{88}H_{76}B_2F_8$	$C_{102}H_{84}B_2F_8$	$C_{134}H_{100}F_{12}N_2O_8S_4$	$C_{78}H_{77}Cl_2$
**Formula weight**	713.93	1076.78	1395.46	1307.1	1483.31	2222.39	1085.29
**Temperature [K]**	150	150	150	150	150	150	150
**Crystal system**	triclinic	monoclinic	monoclinic	triclinic	tetragonal	triclinic	triclinic
**Space group**	P-1	$P2_1/n$	P2/n	P-1	$I4_1/a$	P-1	P-1
**a [Å]**	11.08121(12)	16.6120(3)	18.4135(3)	8.8004(3)	15.5948(6)	15.3955(5)	12.92157(16)
**b [Å]**	13.4917(2)	9.5042(2)	9.93257(11)	15.9682(6)	15.5948(6)	21.2106(7)	15.04323(19)
**c [Å]**	16.1866(2)	17.6414(3)	20.6392(3)	16.1181(5)	37.677(3)	21.4503(6)	16.4212(2)
**α [°]**	95.6869(12)	90	90	84.118(3)	90	77.909(3)	96.0445(11)
**β [°]**	106.8765(11)	94.0685(18)	104.9946(14)	82.203(3)	90	85.462(2)	101.7699(11)
**γ [°]**	113.9838(13)	90	90	81.164(3)	90	70.337(3)	96.5757(10)
**Volume [Å^3]**	2049.54(5)	2778.25(10)	3646.24(8)	2209.73(14)	9162.9(10)	6449.5(4)	3077.03(7)
**Z**	2	2	2	1	4	2	2
**ρ_{calc}g [cm^3]**	1.157	1.287	1.271	0.982	1.075	1.144	1.171
**μ [mm^{-1}]**	0.507	0.81	1.194	0.544	0.58	1.264	1.27
**Crystal size [mm^3]**	0.3 × 0.2 × 0.05	0.4 × 0.3 × 0.03	0.2 × 0.15 × 0.1	0.3 × 0.2 × 0.1	0.2 × 0.1 × 0.05	0.3 × 0.03 × 0.03	0.8 × 0.06 × 0.03
**Reflections collected**	24787	16305	22464	23240	12762	78230	22481
**Independent reflections**	8254	5581	7366	7835	4488	25718	22481
**R_{int}**	0.0198	0.0583	0.0305	0.0839	0.0378	0.1195	0.0409
**Data/restraints/parameters**	8254/0/503	5581/0/322	7366/0/461	7835/0/448	4488/0/290	25718/0/1453	22481/0/792
**GOF**	1.075	1.073	1.081	1.726	1.357	1.227	1.06
**R_1 [I>=2σ (I)]**	0.0495	0.0863	0.0794	0.1623	0.1225	0.127	0.0538
**wR_2 [I>=2σ (I)]**	0.1486	0.2558	0.2228	0.4269	0.3585	0.3345	0.1516
**R_1 [all data]**	0.0533	0.0937	0.0847	0.1839	0.1624	0.1608	0.0611
**wR_2 [all data]**	0.1533	0.2655	0.2272	0.4467	0.4046	0.3682	0.1575
**CCDC**	2218480	2218481	2218482	2218483	2218484	2218485	2218486

#Solvent mask procedure was used for the analyses of $\mathbf{1}^{2+}(BF_4^-)_2$, $\mathbf{3}^{2+}(BF_4^-)_2$, $\mathbf{4}^{2+}(BF_4^-)_2$, and $\mathbf{6}^{2+}(NTf_2^-)_2$.

Voltammetric Analyses (Figures S39-S43)

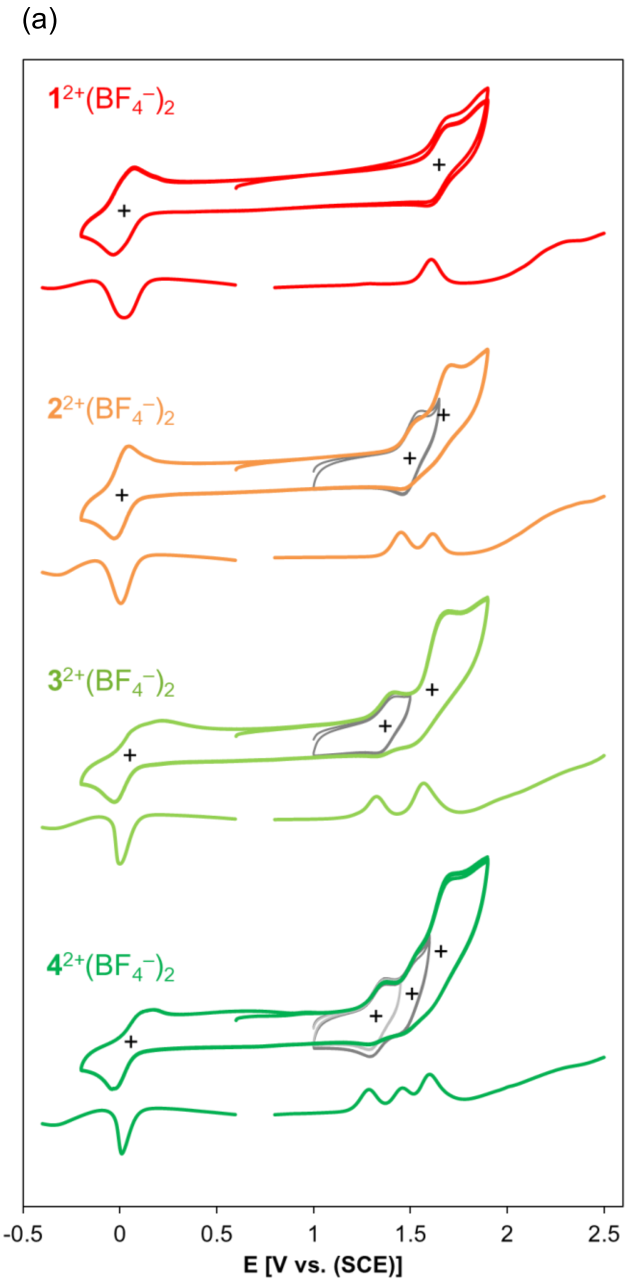

(b)

	E^{red}	$E(\gamma)$	$E(\beta)$	$E(\alpha)$
$1^{2+}(BF_4^-)_2$	+0.02			+1.61
$2^{2+}(BF_4^-)_2$	+0.00		+1.45	+1.62
$3^{2+}(BF_4^-)_2$	+0.00	+1.32		+1.57
$4^{2+}(BF_4^-)_2$	+0.01	+1.29	+1.46	+1.60
Anthracene		+1.17		

[V vs. (SCE)]

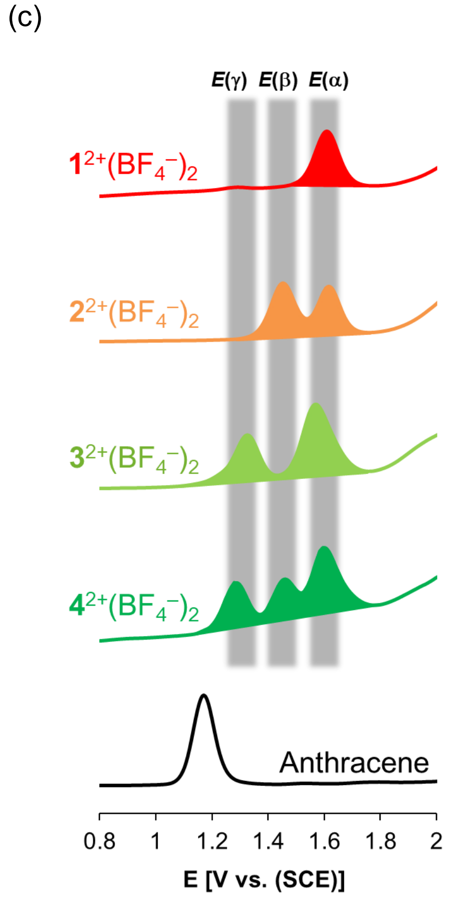

Figure S39. (a) Cyclic voltammograms (scan rate 100 mVs^{-1}) and differential pulse voltammograms of dications $\mathbf{1}^{2+}(BF_4^-)_2$-$\mathbf{4}^{2+}(BF_4^-)_2$ (1.0 mM) in CH_3CN containing 0.1 M Et_4NClO_4 as a supporting electrolyte (Pt electrode) and (b) their redox potentials. (c) The oxidation potentials are classified into three types by considering the charge state of the adjacent tricyclic units.

(a)

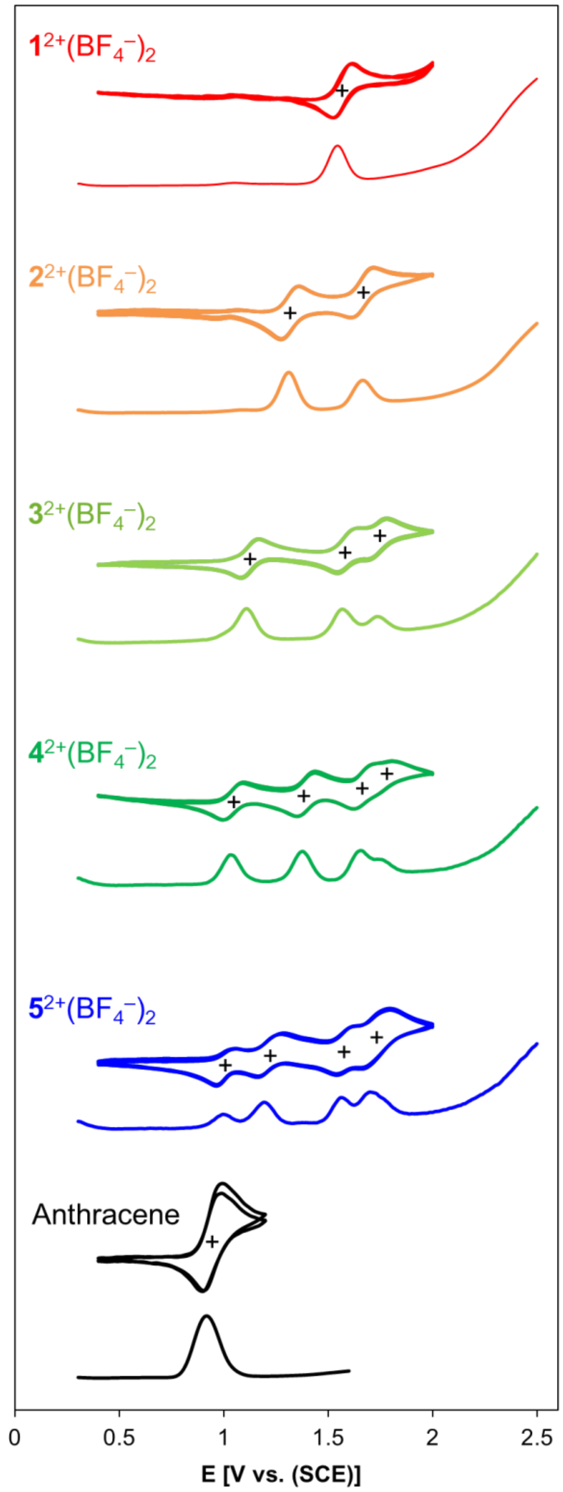

(b)

	E(γ)		*E*(β)	*E*(α)	
$1^{2+}(BF_4^-)_2$				+1.54	
$2^{2+}(BF_4^-)_2$			+1.31	+1.67	
$3^{2+}(BF_4^-)_2$	+1.11			+1.57	+1.74
$4^{2+}(BF_4^-)_2$	+1.04		+1.38	+1.66	+1.74
$5^{2+}(BF_4^-)_2$	+1.00	+1.19		+1.56	+1.70
Anthracene	+0.92				

[V vs. (SCE)]

Figure S40. (a) Cyclic voltammograms (scan rate 100 mVs^{-1}) and differential pulse voltammograms of dications $\mathbf{1}^{2+}(BF_4^-)_2$-$\mathbf{5}^{2+}(BF_4^-)_2$ (1.0 mM) and anthracene (1.0 mM) in HFIP containing 0.1 M Bu_4NBF_4 as a supporting electrolyte (Pt electrode) and (b) their redox potentials. Backgrounds were subtracted for all cyclic voltammograms.

(a)

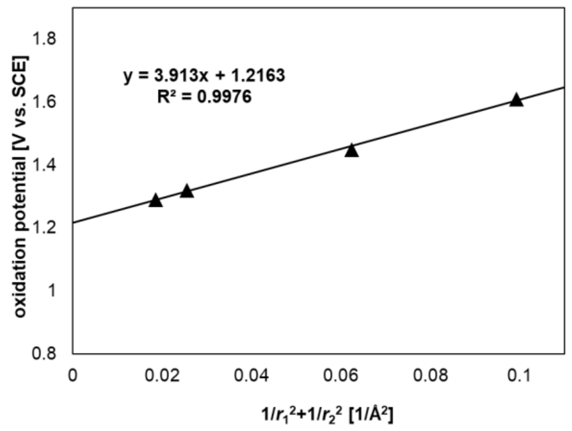

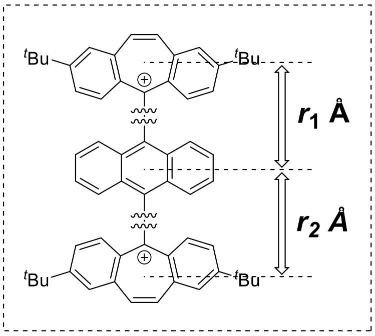

(b)

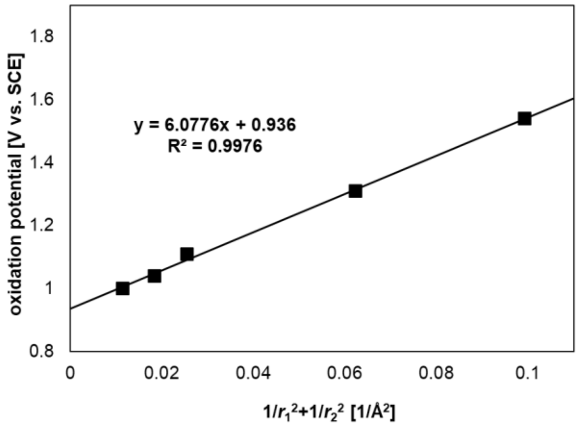

Figure S41. The plot of the oxidation potential for dications (a) $\mathbf{1}^{2+}(BF_4^-)_2$-$\mathbf{4}^{2+}(BF_4^-)_2$ in CH_3CN and (b) $\mathbf{1}^{2+}(BF_4^-)_2$-$\mathbf{5}^{2+}(BF_4^-)_2$ in HFIP against the sum of $1/r^2$ where r is the distance between the center of gravity of the dibenzotropylium unit and the central anthrylene unit(s) in the optimized structures. A linear correlation was observed and the differences between the value of the intercept (1.22 and 0.94) and the oxidation potential of the parent anthracene (+1.17 V and +0.92 V) are only 0.05 V and 0.02 V, respectively, in CH_3CN and HFIP.

(a)

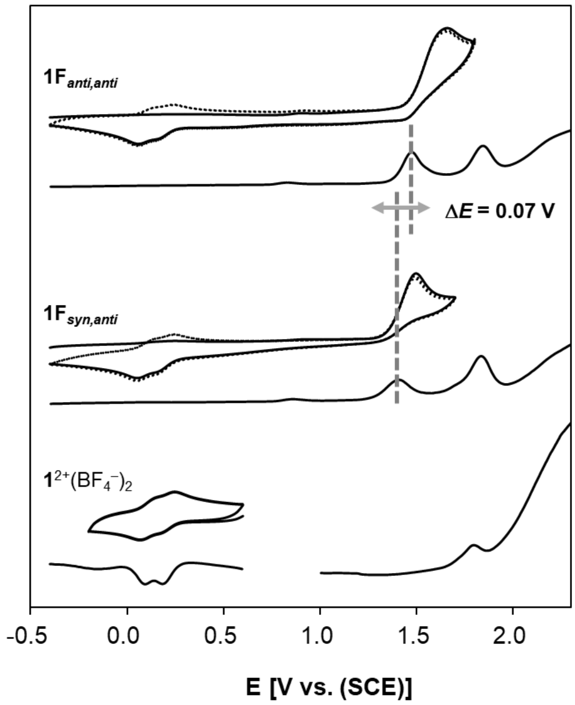

(b)

	E^{red}	E^{ox}	$E(\alpha)$
**1F**$_{anti,anti}$	-	+1.48	+1.84
as-prepared $\mathbf{1}^{2+}/\mathbf{1}^{+\bullet}$	+0.20	= E^{red}	-
as-prepared $\mathbf{1}^{+\bullet}$/**1T**	+0.10	= E^{red}	-
**1F**$_{syn,anti}$	-	+1.41	+1.84
as-prepared $\mathbf{1}^{2+}/\mathbf{1}^{+\bullet}$	+0.20	= E^{red}	-
as-prepared $\mathbf{1}^{+\bullet}$/**1T**	+0.09	= E^{red}	-
$\mathbf{1}^{2+}(BF_4^-)_2/\mathbf{1}^{+\bullet}$	+0.18	= E^{red}	+1.80
as-prepared $\mathbf{1}^{+\bullet}$/**1T**	+0.09	= E^{red}	

[V vs. (SCE)]

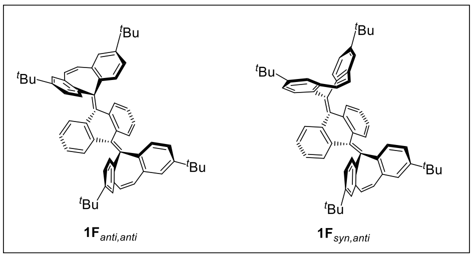

Figure S42. (a) Cyclic voltammograms (scan rate 500 mVs^{-1}) and differential pulse voltammograms of 1.0 mM solution of **1F**$_{anti,anti}$, **1F**$_{syn,anti}$ and $\mathbf{1}^{2+}(BF_4^-)_2$ in CH_2Cl_2 containing 0.1 M Bu_4NBF_4 as a supporting electrolyte (Pt electrode) and (b) their redox potentials. Backgrounds were subtracted for all cyclic voltammograms. The second and third cycles are shown by dotted lines in the cyclic voltammograms of **1F**$_{anti,anti}$ and **1F**$_{syn,anti}$.

(a)

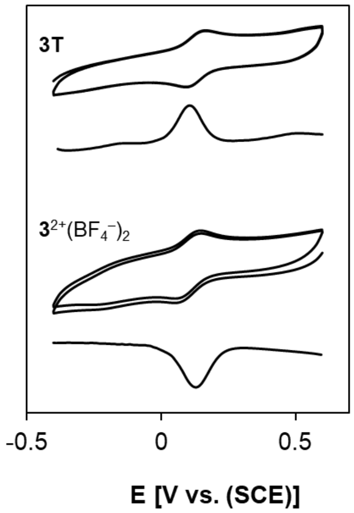

(b)

	E^{red}	E^{ox}
**3T**	+0.11	-
$\mathbf{3}^{2+}(BF_4^-)_2$	-	+0.13

[V vs. (SCE)]

Figure S43. (a) Cyclic voltammograms (scan rate 100 mVs^{-1}) and differential pulse voltammograms of 0.2 mM solution of **3T** and $\mathbf{3}^{2+}(BF_4^-)_2$ in CH_2Cl_2 containing 0.1 M Bu_4NBF_4 as a supporting electrolyte (Pt electrode) and (b) their redox potentials.

Spectroscopic Analyses (Table S4, Figures S44-S52)

UV-Vis-NIR spectra of dications

Table S4. Maximum absorption wavelengths λ_{max} and molar absorption coefficients ε of dications $\mathbf{1}^{2+}(BF_4^-)_2$-$\mathbf{6}^{2+}(BF_4^-)_2$ in CH_3CN.

dications	λ_{max} [nm] (ε [M^{-1} cm^{-1}])								
$\mathbf{1}^{2+}(BF_4^-)_2$	646 (833)	546 (7460)	519 (6840)	433 (40500)	400 (20800)	378 (18800)	318 (201000)	258 (83900)	228 (38000)
$\mathbf{2}^{2+}(BF_4^-)_2$	670 (840)	544 (8080)	515 (7550)	434 (37200)	403 (41800)	381 (31900)	321 (202000)	257 (158000)	228 (63200)
$\mathbf{3}^{2+}(BF_4^-)_2$	680 (797)	543 (7880)	514 (7390)	434 (35000)	404 (58000)	382 (39900)	320 (192000)	254 (210000)	228 (91400)
$\mathbf{4}^{2+}(BF_4^-)_2$	682 (808)	543 (7960)	514 (7500)	434 (35000)	405 (83400)	382 (48500)	321 (193000)	252 (259000)	229 (118000)
$\mathbf{5}^{2+}(BF_4^-)_2$	682 (805)	543 (7590)	514 (7510)	433 (35000)	407 (110000)	383 (58000)	321 (192000)	251 (322000)	229 (154000)
$\mathbf{6}^{2+}(BF_4^-)_2$	679 (854)	543 (7940)	514 (7510)	433 (35600)	407 (132000)	383 (65200)	321 (191000)	250 (369000)	229 (187000)

ESR spectra of biradicals

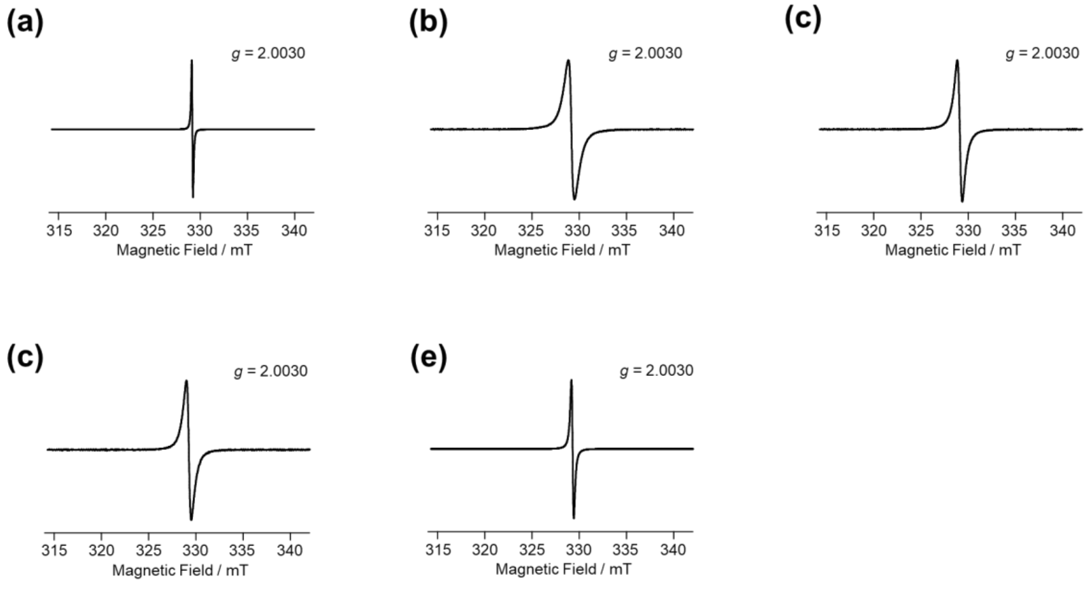

Figure S44. ESR spectra of biradicals: (a) **2T**, (b) **3T**, (c) **4T**, (d) **5T** and (e) **6T** in a solid state.

Electrochemical reduction and oxidation

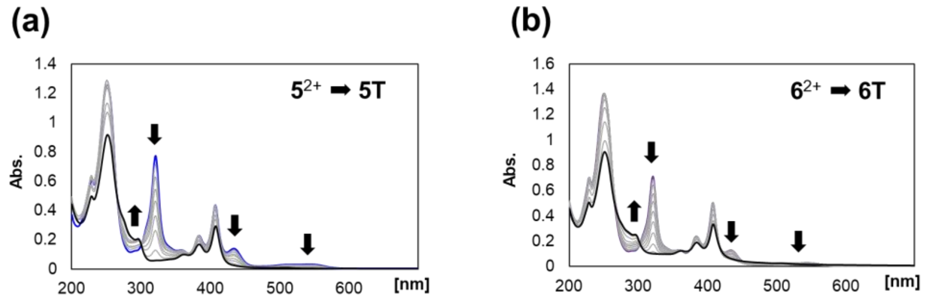

Figure S45. Changes in UV-Vis spectra upon electrochemical reduction (20 μA) of (a) **5**$^{2+}$(BF_4^-)$_2$ (4.02 μM) and (b) **6**$^{2+}$(BF_4^-)$_2$ (3.73 μM) in CH_3CN containing 0.05 M Et_4NClO_4 as a supporting electrolyte (every 30 seconds).

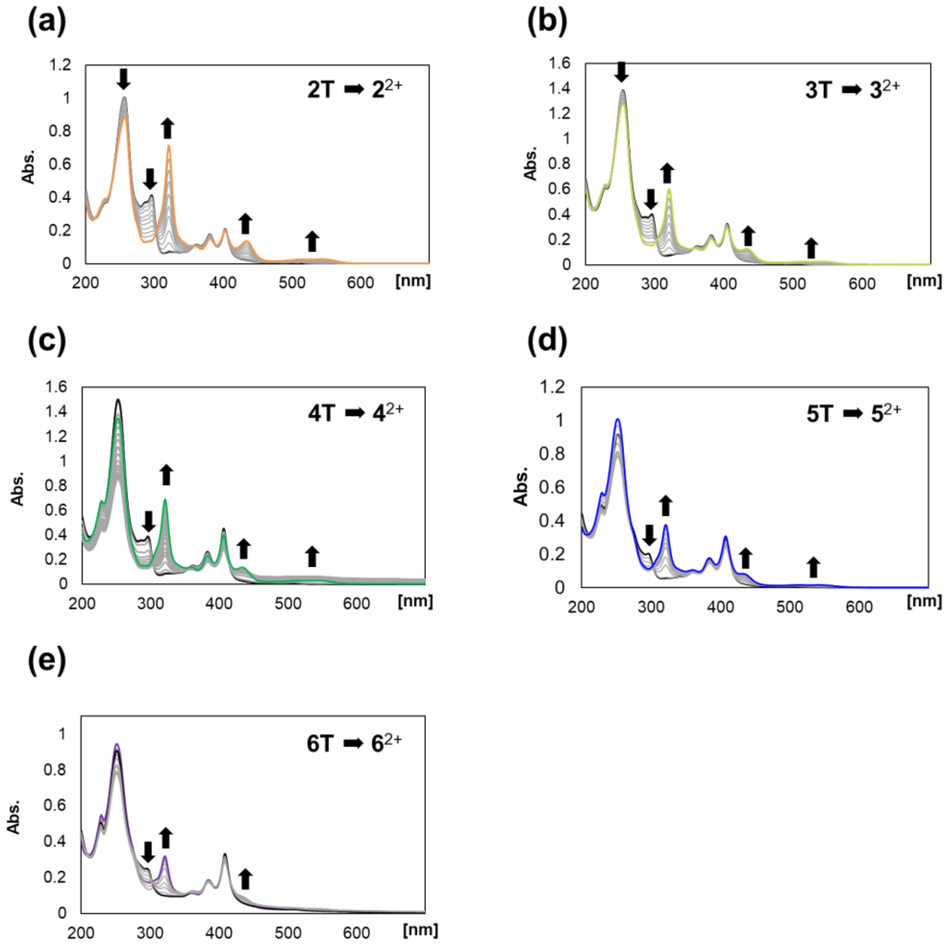

Figure S46. Changes in UV-Vis spectra upon electrochemical oxidation (20 μA) of as-prepared (a) **2T**, (b) **3T**, (c) **4T**, (d) **5T** and (e) **6T** in CH_3CN containing 0.05 M Et_4NClO_4 as a supporting electrolyte (every 30 seconds).

UV-Vis spectra of isolated neutral species

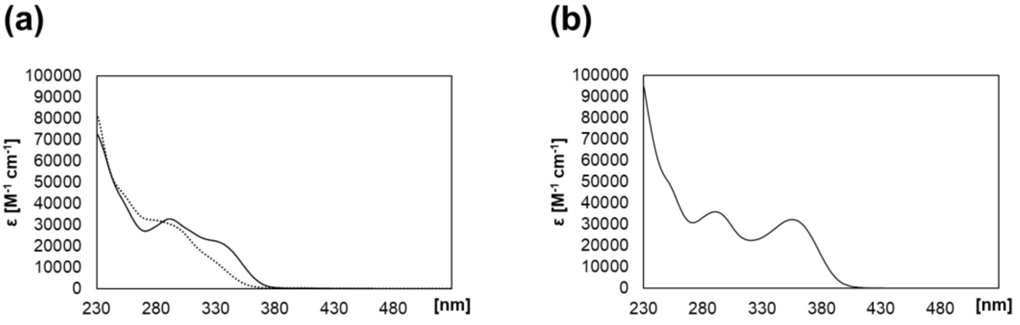

folded forms	λ_{max} [nm] (ε [M^{-1} cm^{-1}])	
**1F**$_{anti,anti}$	331 (22300)	291 (32800)
**1F**$_{syn,anti}$		295 (29600)
**2F**	355 (32300)	290 (36000)

Figure S47. UV-Vis spectra of closed-shell **F**-forms: (a) **1F**$_{anti,anti}$ (solid line) and **1F**$_{syn,anti}$ (dotted line) and (b) **2F** in CH_2Cl_2.

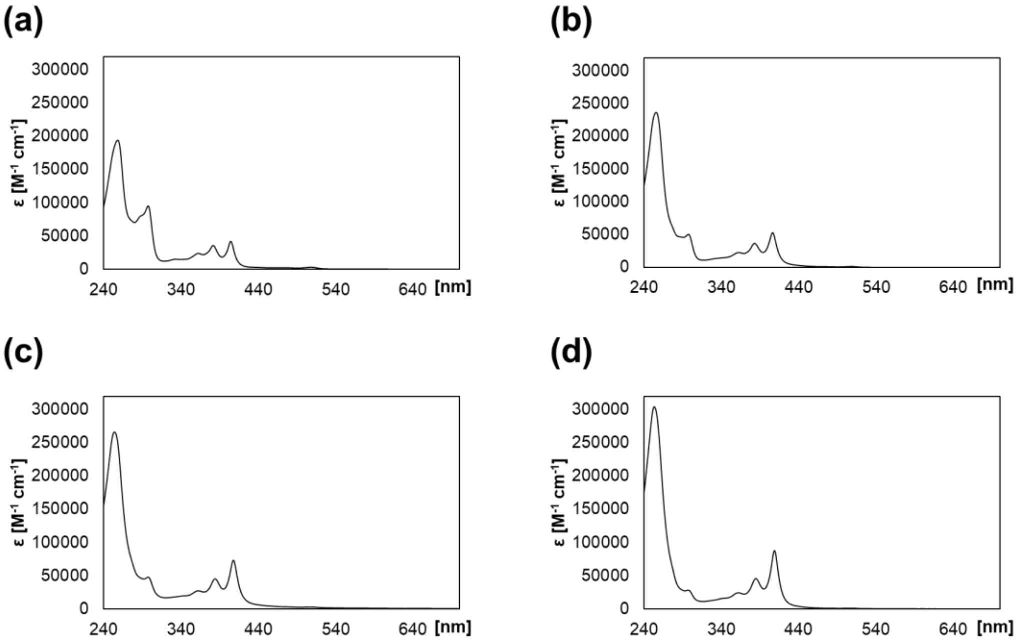

biradicals	λ_{max} [nm] (ε [M^{-1} cm^{-1}])					
**2T**	508 (2940)	404 (41900)	382 (35500)	362 (23800)	297 (95000)	258 (194000)
**3T**	508 (1440)	406 (53200)	383 (36800)	363 (22700)	297 (50600)	255 (237000)
**4T**	507 (2850)	408 (73100)	384 (45400)	362 (27200)	297 (47900)	254 (266000)
**5T**	507 (892)	409 (87400)	385 (45500)	362 (24200)	297 (28100)	253 (304000)

Figure S48. UV-Vis spectra of open-shell **T**-forms: (a) **2T**, (b) **3T**, (c) **4T** and (d) **5T** in THF.

Comparison of UV-Vis spectra of neutral species

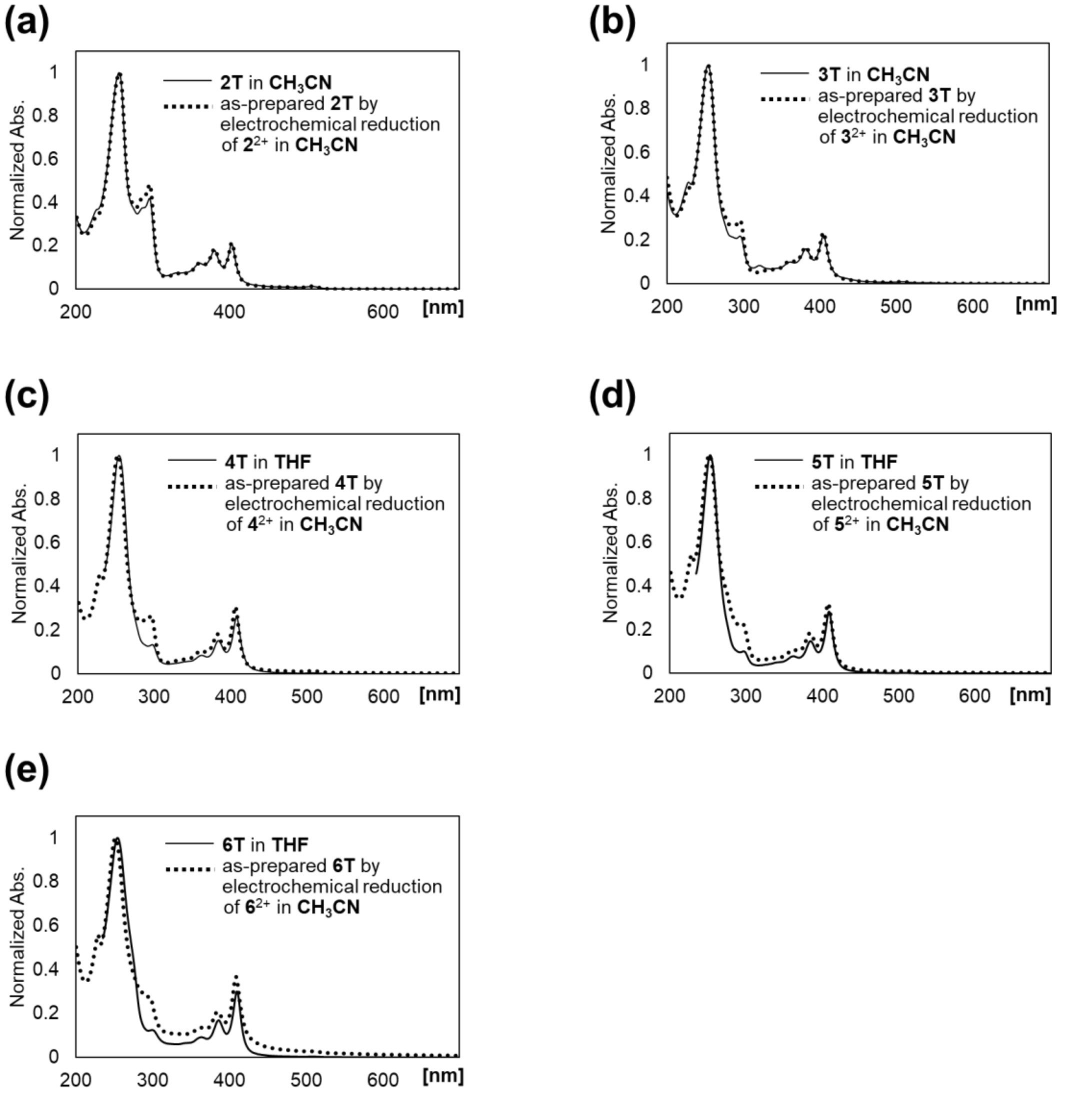

Figure S49. UV-Vis spectra of (a) isolated **2T** and as-prepared **2T** by electrochemical reduction of $\mathbf{2}^{2+}(BF_4^-)_2$ in CH_3CN, (b) isolated **3T** and as-prepared **3T** by electrochemical reduction of $\mathbf{3}^{2+}(BF_4^-)_2$ in CH_3CN, (c) isolated **4T** in THF and as-prepared **4T** by electrochemical reduction of $\mathbf{4}^{2+}(BF_4^-)_2$ in CH_3CN, (d) isolated **5T** in THF and as-prepared **5T** by electrochemical reduction of $\mathbf{5}^{2+}(BF_4^-)_2$ in CH_3CN and (e) isolated **6T** in THF and as-prepared **6T** by electrochemical reduction of $\mathbf{6}^{2+}(BF_4^-)_2$ in CH_3CN.

IR spectra of dications and corresponding neutral species

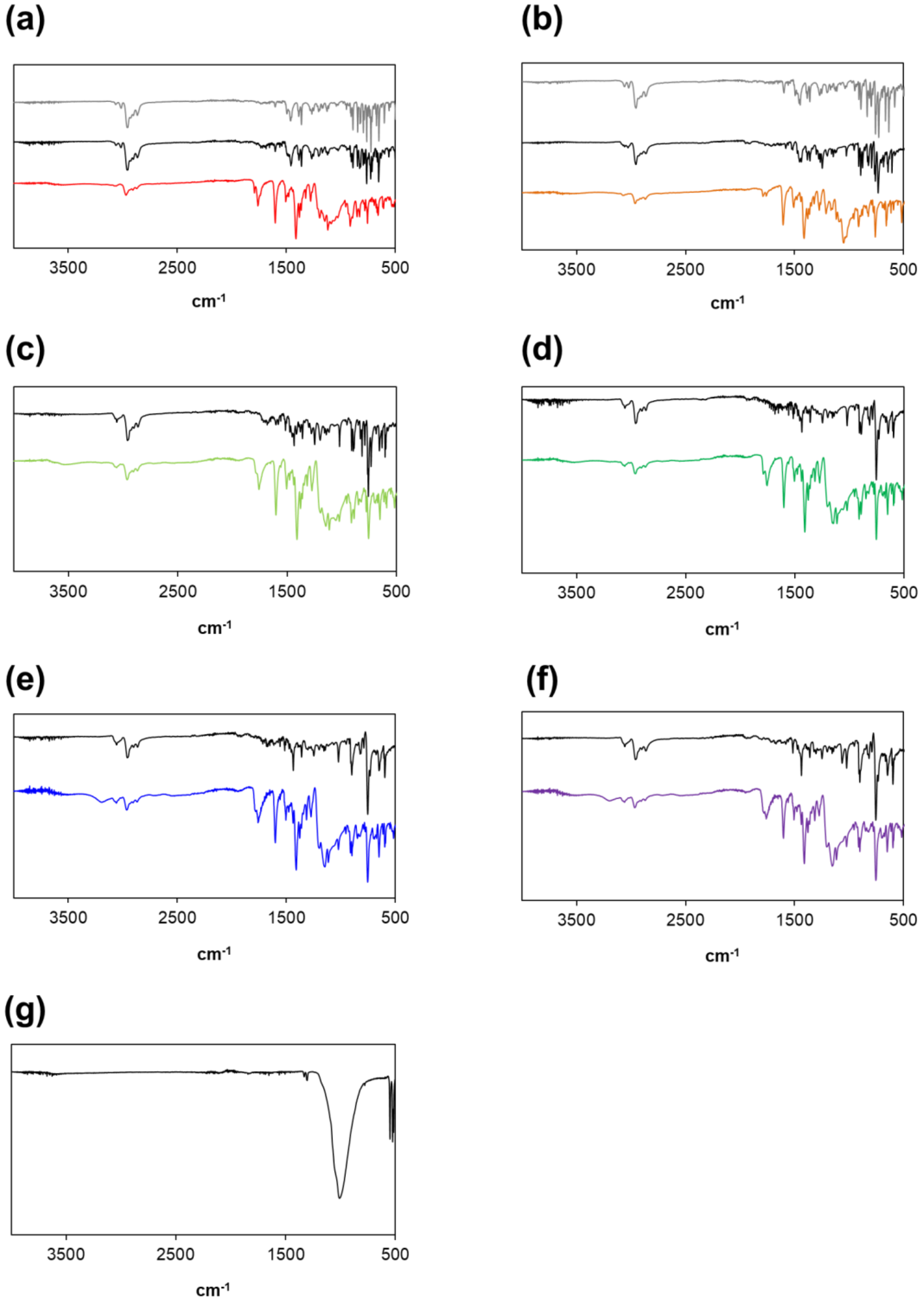

Figure S50. IR spectra of (a) **1**$^{2+}(BF_4^-)_2$ (red), **1F**$_{\mathit{syn,anti}}$ (black), and **1F**$_{\mathit{anti,anti}}$ (gray), (b) **2**$^{2+}(BF_4^-)_2$ (orange), **2T** (black), and **2F** (gray), (c) **3**$^{2+}(BF_4^-)_2$ (olive) and **3T** (black), (d) **4**$^{2+}(BF_4^-)_2$ (green) and **4T** (black), (e) **5**$^{2+}(BF_4^-)_2$ (blue) and **5T** (black), (f) **6**$^{2+}(BF_4^-)_2$ (purple) and **6T** (black), and (g) $NaBF_4$.

Thermal isomerization from 2T to 2F

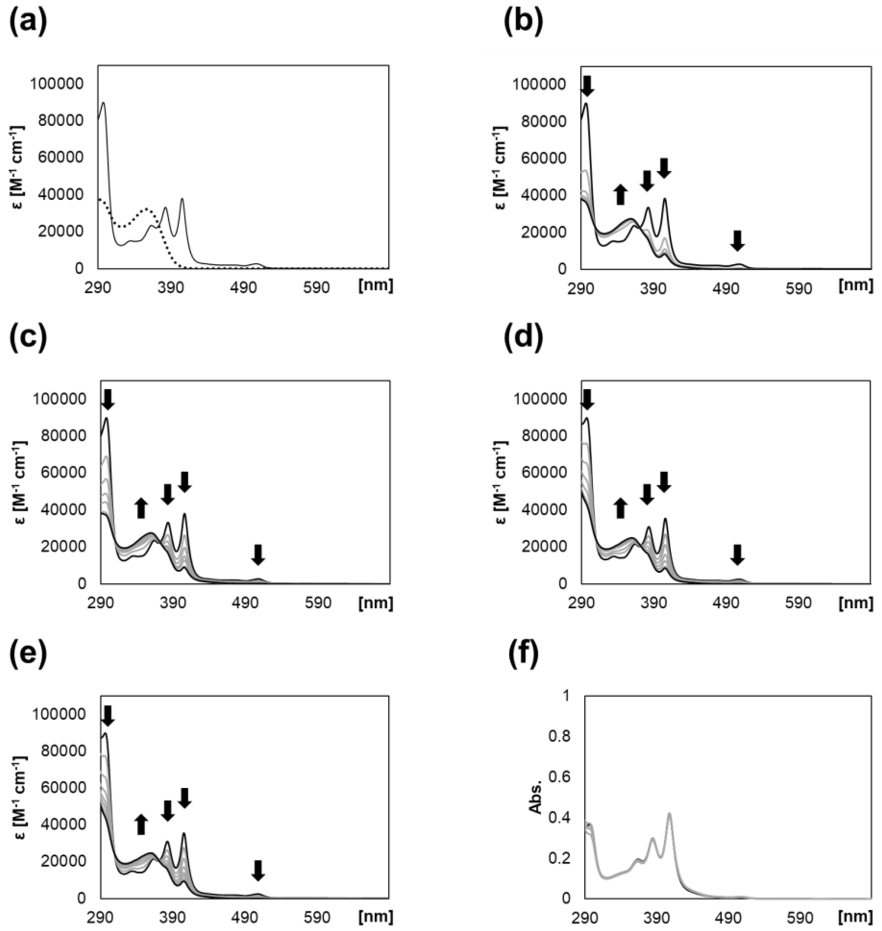

Figure S51. (a) UV-Vis spectra of **2T** (solid line) and **2F** (dotted line), and changes in UV-Vis spectra of (b) **2T** upon heating at 100 °C (0, 2, 4, 6, 8, 12, 16 min), (c) **2T** upon heating at 90 °C (0, 2, 4, 6, 8, 12, 16 min), (d) **2T** upon heating at 80 °C (0, 4, 8, 12, 16, 24, 32 min), (e) **2T** upon heating at 70 °C (0, 8, 16, 24, 32, 40, 48, 64 min), and (f) **3T** upon heating at 100 °C (0, 2, 4, 8, 16 min) in toluene.

from **2T** to **2F** in toluene	
T (°C)	*k* (s^{-1})
100	8.67 x 10^{-3}
90	4.02 x 10^{-3}
80	1.34 x 10^{-3}
70	6.08 x 10^{-4}

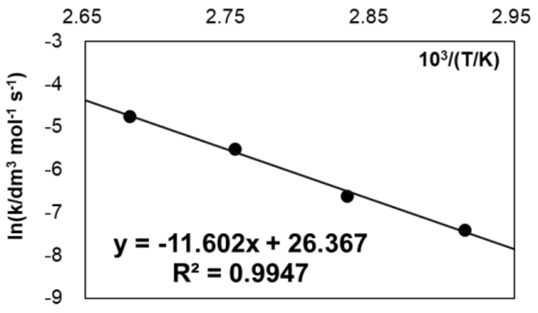

Figure S52. The rate constants *k* of thermal isomerization from **2T** to **2F** and an Arrhenius plot.

Optimized Coordinates

1^{2+}: CAM-B3LYP/6-31G(d)

SCF Done: E(RCAM-B3LYP) = -2320.67756199 A.U. after 6 cycles

Tag	Symbol	X	Y	Z	Tag	Symbol	X	Y	Z
1	C	1.4110497	0.0000179	0.0000027	38	C	-4.472538	4.0305133	-0.000126
2	C	0.7184365	0.0000312	1.222036	39	C	-5.3413649	2.9619625	-0.0000456
3	C	1.398049	0.0000494	2.4816955	40	C	-4.9300994	1.6098113	0.0000031
4	C	0.7107558	0.0000632	3.6557085	41	C	-6.0037405	0.6779694	0.0000835
5	C	-0.7107406	0.0000583	3.6557114	42	C	-6.0037215	-0.6780871	0.0001202
6	C	-1.3980389	0.0000384	2.4817014	43	H	-6.988251	1.1358501	0.0001125
7	C	-0.7184322	0.0000233	1.2220388	44	H	-6.988219	-1.1359957	0.0001725
8	C	-1.4110497	-0.0000004	0.000008	45	C	6.0037279	0.6780699	-0.0000134
9	C	-0.7184364	-0.0000183	-1.2220253	46	C	6.0037348	-0.6779867	-0.0000455
10	C	-1.3980491	-0.0000464	-2.4816844	47	H	6.9882296	1.1359695	-0.0000107
11	C	-0.710756	-0.0000594	-3.6556977	48	H	6.9882411	-1.1358763	-0.0000701
12	C	0.7107403	-0.0000442	-3.6557008	49	H	2.4820368	0.000052	2.4961335
13	C	1.3980389	-0.000018	-2.4816907	50	H	1.2456046	0.0000774	4.5993824
14	C	0.7184319	-0.000006	-1.2220283	51	H	-1.2455854	0.0000693	4.5993876
15	C	2.9245637	0.000026	-0.0000014	52	H	-2.4820265	0.0000333	2.4961447
16	C	3.5244718	-1.2952172	-0.0000036	53	H	-2.4820368	-0.0000568	-2.496122
17	C	4.9300851	-1.6098187	-0.0000404	54	H	-1.245605	-0.0000804	-4.5993715
18	C	5.3413367	-2.9619742	-0.000067	55	H	1.2455851	-0.0000534	-4.599377
19	C	4.4724988	-4.0305161	-0.0000497	56	H	2.4820266	-0.0000059	-2.4961338
20	C	3.090753	-3.7164758	0.0000043	57	H	6.4077472	-3.1467488	-0.000101
21	C	2.6471899	-2.4275825	0.0000254	58	H	2.360552	-4.5175409	0.0000301
22	C	3.5244586	1.2952754	-0.0000045	59	H	1.5817333	-2.2582577	0.0000672
23	C	4.9300688	1.6098911	0.0000076	60	H	6.4077155	3.1468359	0.000045
24	C	5.3413068	2.9620506	0.0000316	61	H	2.3605066	4.5175874	-0.0000011
25	C	4.4724582	4.0305838	0.000035	62	H	1.5817106	2.2582965	-0.0000352
26	C	3.0907156	3.7165296	0.0000057	63	H	-6.4076852	-3.1468579	0.0001567
27	C	2.6471654	2.4276319	-0.0000135	64	H	-2.3604623	-4.5175688	0.0000883
28	C	-2.9245635	-0.0000152	0.0000156	65	H	-1.5816891	-2.2582694	0.0000357
29	C	-3.524447	-1.295269	0.0000585	66	H	-1.5817548	2.2582853	-0.0001248
30	C	-4.9300538	-1.6098982	0.0000974	67	H	-2.3605964	4.5175595	-0.000214
31	C	-5.3412784	-2.9620619	0.0001278	68	H	-6.4077773	3.1467262	-0.0000196
32	C	-4.4724189	-4.0305864	0.0001232	69	C	4.9275025	5.4848269	0.0000635
33	C	-3.0906794	-3.7165183	0.0000902	70	C	4.3713645	6.1778119	1.2597139
34	C	-2.6471423	-2.4276162	0.0000611	71	H	4.6836883	7.2258821	1.2676833
35	C	-3.5244829	1.2952233	-0.0000255	72	H	3.2781872	6.1606054	1.2951376
36	C	-2.6472129	2.4275982	-0.0001013	73	H	4.749526	5.707113	2.1722145
37	C	-3.0907892	3.7164869	-0.0001523	74	C	4.3714195	6.1778456	-1.2595927

Tag	Symbol	X	Y	Z
75	H	4.6837476	7.2259149	-1.2675225
76	H	4.7496175	5.7071684	-2.1720894
77	H	3.2782437	6.1606443	-1.2950626
78	C	6.4540391	5.6188891	0.0000985
79	H	6.9061519	5.1689175	0.8896418
80	H	6.9061912	5.168934	-0.8894332
81	H	6.7244017	6.6777684	0.0001144
82	C	4.9275576	-5.4847546	-0.0000782
83	C	4.3714889	-6.1777746	1.2595836
84	H	3.2783132	-6.1605791	1.2950621
85	H	4.6838229	-7.2258421	1.2675125
86	H	4.7496914	-5.7070939	2.1720766
87	C	4.371419	-6.1777492	-1.2597229
88	H	4.7495668	-5.7070466	-2.1722274
89	H	4.6837574	-7.2258151	-1.2676931
90	H	3.2782413	-6.1605585	-1.2951382
91	C	6.4540955	-5.6188016	-0.0001223
92	H	6.9061994	-5.1688122	-0.8896612
93	H	6.9062476	-5.1688553	0.8894138
94	H	6.7244687	-6.6776782	-0.0001546
95	C	-4.9274486	-5.4848341	0.0001529
96	C	-4.3712952	-6.1778163	1.2597979
97	H	-4.6836085	-7.2258896	1.267767
98	H	-3.2781178	-6.1605992	1.2952143
99	H	-4.7494551	-5.7071232	2.1723022
100	C	-4.371367	-6.1778443	-1.2595087
101	H	-4.6836844	-7.2259167	-1.2674388
102	H	-4.7495759	-5.7071689	-2.1720018
103	H	-3.2781916	-6.1606317	-1.2949859
104	C	-6.4539838	-5.6189117	0.0001977
105	H	-6.9060951	-5.1689504	0.889747
106	H	-6.9061466	-5.1689552	-0.889328
107	H	-6.7243358	-6.6777936	0.0002087
108	C	-4.9276118	5.4847472	-0.0001839
109	C	-4.3714695	6.1778249	1.2594136
110	H	-3.2782913	6.1606429	1.2948225
111	H	-4.6838144	7.2258893	1.2673196
112	H	-4.7496082	5.7071774	2.1719502
113	C	-4.371561	6.1776957	-1.259893
114	H	-4.7497629	5.706952	-2.1723537
115	H	-4.68391	7.2257582	-1.267886
116	H	-3.2783853	6.1605137	-1.2953778
117	C	-6.454151	5.6187786	-0.0001352
118	H	-6.906307	5.1687563	-0.8896309
119	H	-6.9062417	5.1688559	0.8894441
120	H	-6.7245351	6.6776524	-0.0001844

2^{2+}: CAM-B3LYP/6-31G(d)

SCF Done: E(RCAM-B3LYP) = -2858.71168304 A.U. after 7 cycles

Tag	Symbol	X	Y	Z		Tag	Symbol	X	Y	Z
1	C	-3.5803496	0.0000011	0.0000029		38	H	-3.4151444	-3.2514527	-3.251705
2	C	-2.8883729	0.8637091	0.8637726		39	H	-4.6524328	-1.7656372	-1.7657751
3	C	-3.5681833	1.7543822	1.7545144		40	H	-8.572977	2.2264456	-2.2257947
4	C	-2.8800351	2.5841264	2.5843224		41	H	-4.5229085	3.1927545	-3.1921811
5	C	-1.4586172	2.5833729	2.583569		42	H	-3.7476439	1.5935486	-1.5933774
6	C	-0.7734919	1.7526193	1.752753		43	H	-8.5730033	-2.2264168	2.2257801
7	C	-1.4520638	0.8616245	0.8616905		44	H	-4.5229473	-3.1927537	3.1921894
8	C	-0.7500135	0.0000016	0.0000044		45	H	-3.7476625	-1.5935551	1.5933894
9	C	-1.4520626	-0.8616208	-0.8616832		46	C	3.5803493	0.0000017	0.0000046
10	C	-0.7734893	-1.7526145	-1.7527457		47	C	2.8883718	0.8637725	-0.8637018
11	C	-1.4586134	-2.5833672	-2.5835636		48	C	3.5681812	1.754518	-1.7543722
12	C	-2.8800313	-2.5841207	-2.584319		49	C	2.8800321	2.5843273	-2.5841144
13	C	-3.5681808	-1.7543774	-1.7545111		50	C	1.4586142	2.5835724	-2.5833611
14	C	-2.8883717	-0.8637054	-0.8637673		51	C	0.7734898	1.7527538	-1.7526093
15	C	-5.0909895	0.0000023	0.0000009		52	C	1.4520627	0.8616898	-0.8616168
16	C	-5.6922754	0.9161166	-0.9159427		53	C	0.750013	0.0000019	0.0000048
17	C	-7.0967495	1.1386692	-1.1383749		54	C	1.4520627	-0.8616872	0.8616251
18	C	-7.5066943	2.0950182	-2.0944638		55	C	0.7734899	-1.7527524	1.7526166
19	C	-6.635836	2.8497521	-2.8490807		56	C	1.4586145	-2.5835729	2.5833664
20	C	-5.2549381	2.6274384	-2.6269287		57	C	2.8800324	-2.5843289	2.5841184
21	C	-4.8127069	1.7153157	-1.7150359		58	C	3.5681813	-1.7545186	1.754377
22	C	-5.6922852	-0.916109	0.9159421		59	C	2.8883718	-0.863771	0.863709
23	C	-7.0967622	-1.1386515	1.1383677		60	C	5.0909892	-0.0000005	0.0000019
24	C	-7.5067189	-2.0949967	2.0944553		61	C	5.6922879	0.9159515	0.9161009
25	C	-6.6358703	-2.8497378	2.8490759		62	C	7.0967658	1.1383771	1.1386384
26	C	-5.2549697	-2.6274339	2.6269314		63	C	7.5067263	2.0944747	2.0949719
27	C	-4.812727	-1.7153143	1.7150411		64	C	6.6358808	2.8491074	2.8497044
28	C	-8.1710195	-0.4792846	0.4791488		65	C	5.2549794	2.6269649	2.6274039
29	C	-8.1710143	0.4793082	-0.4791623		66	C	4.8127331	1.7150646	1.715296
30	H	-9.1553848	-0.8034656	0.8032251		67	C	5.6922725	-0.9159573	-0.9161008
31	H	-9.1553759	0.8034949	-0.8032439		68	C	7.0967458	-1.1383942	-1.1386534
32	H	-4.6524354	1.7656419	1.7657769		69	C	7.5066873	-2.0944986	-2.0949884
33	H	-3.4151492	3.2514591	3.2517069		70	C	6.6358264	-2.8491259	-2.8497088
34	H	-0.9239214	3.2507863	3.2510333		71	C	5.2549293	-2.6269715	-2.6273928
35	H	0.3098424	1.754127	1.7542622		72	C	4.8127013	-1.7150648	-1.7152825
36	H	0.309845	-1.754122	-1.7542535		73	C	8.1710128	-0.4791719	-0.4793058
37	H	-0.9239166	-3.2507798	-3.2510279		74	C	8.171021	0.4791512	0.479275

Tag	Symbol	X	Y	Z
75	H	9.1553733	-0.8032571	-0.8034921
76	H	9.1553873	0.8032323	0.8034481
77	H	4.6524332	1.7657825	-1.7656312
78	H	3.4151455	3.2517145	-3.2514449
79	H	0.9239178	3.2510378	-3.2507728
80	H	-0.3098445	1.7542622	-1.7541166
81	H	-0.3098444	-1.7542602	1.7541246
82	H	0.9239181	-3.2510391	3.2507773
83	H	3.4151459	-3.2517177	3.2514472
84	H	4.6524333	-1.7657839	1.7656351
85	H	8.5730112	2.2257993	2.226388
86	H	4.5229592	3.1922319	3.1927175
87	H	3.7476681	1.5934164	1.5935384
88	H	8.5729695	-2.2258315	-2.2264175
89	H	4.5228977	-3.1922354	-3.1926948
90	H	3.7476386	-1.5934065	-1.5935114
91	C	7.0896018	3.8778538	3.8787108
92	C	6.5331163	3.4772176	5.2591069
93	H	6.8441599	4.2128551	6.0063839
94	H	5.4401065	3.4385686	5.2706459
95	H	6.9116982	2.4991654	5.5712334
96	C	6.5332994	5.2583924	3.478312
97	H	6.8443562	6.0054827	4.2141335
98	H	6.9120036	5.5706828	2.5003594
99	H	5.4402946	5.270058	3.4395665
100	C	8.6159443	3.9739637	3.9749975
101	H	9.0683359	3.0268144	4.2855663
102	H	9.0684665	4.2846623	3.0279529
103	H	8.8852051	4.7228941	4.7240958
104	C	7.0895268	-3.8778829	-3.8787138
105	C	6.5331836	-5.258406	-3.4783183
106	H	5.4401783	-5.2700381	-3.4395721
107	H	6.8442175	-6.0055037	-4.2141421
108	H	6.911879	-5.5707102	-2.5003667
109	C	6.5330623	-3.4772321	-5.2591142
110	H	6.8440991	-4.2128736	-6.0063901
111	H	5.4400536	-3.4385619	-5.2706648
112	H	6.9116667	-2.4991865	-5.5712344
113	C	8.6158673	-3.9740339	-3.9749898
114	H	9.0682884	-3.0268887	-4.2855287
115	H	9.0683728	-4.2847714	-3.0279501
116	H	8.8851131	-4.7229519	-4.7241059
117	C	-7.0895872	-3.8787539	3.8778143
118	C	-6.5332469	-3.4783861	5.2583466
119	H	-6.8442927	-4.214218	6.0054312
120	H	-5.4402413	-3.439652	5.2699849
121	H	-6.9119331	-2.5004352	5.5706641
122	C	-6.5331357	-5.2591542	3.4771449
123	H	-6.9117384	-5.571255	2.4990925
124	H	-5.4401269	-5.2707161	3.4384802
125	H	-6.8441845	-6.0064381	4.2127731
126	C	-8.6159293	-3.975015	3.9739559
127	H	-9.0684262	-3.0279753	4.284706
128	H	-9.0683488	-4.2855341	3.0268035
129	H	-8.8851871	-4.72414	4.7228607
130	C	-7.08954	3.8787762	-3.8778168
131	C	-6.5330375	5.2591602	-3.4771618
132	H	-5.4400278	5.2706832	-3.4385063
133	H	-6.8440665	6.0064498	-4.2127926
134	H	-6.9116208	5.5712808	-2.4991082
135	C	-6.5332381	3.478387	-5.2583584
136	H	-6.844285	4.2142203	-6.0054411
137	H	-5.4402337	3.4396299	-5.2700218
138	H	-6.9119522	2.5004421	-5.5706608
139	C	-8.6158808	3.975084	-3.9739308
140	H	-9.0684148	3.0280467	-4.2846343
141	H	-9.0682712	4.2856555	-3.0267817
142	H	-8.8851292	4.724189	-4.722859

3^{2+}: CAM-B3LYP/6-31G(d)

SCF Done: E(RCAM-B3LYP) = -3396.73853940 A.U. after 6 cycles										
Tag	Symbol	X	Y	Z		Tag	Symbol	X	Y	Z
1	C	-5.7492496	-0.0000012	-0.0001236		38	H	-5.5804843	0.0000904	-4.5990999
2	C	-5.0578321	-0.0000206	1.2217619		39	H	-6.8208042	0.0000525	-2.4999764
3	C	-5.7364743	-0.0000465	2.481984		40	H	-10.74046	3.1487565	0.0001978
4	C	-5.0462235	-0.0000673	3.6545058		41	H	-6.6892794	4.5135271	0.0000836
5	C	-3.6248383	-0.0000633	3.6526443		42	H	-5.915744	2.25075	-0.0001163
6	C	-2.9410532	-0.0000411	2.4768832		43	H	-10.740483	-3.1487304	-0.0005704
7	C	-3.6218421	-0.0000212	1.2179514		44	H	-6.6893136	-4.5135329	-0.0004496
8	C	-2.9187158	-0.000001	-0.0000757		45	H	-5.9157603	-2.2507628	-0.0001937
9	C	-3.6218008	0.0000201	-1.2181267		46	C	1.4196479	0.0000002	-0.0000043
10	C	-2.9409698	0.0000409	-2.4770357		47	C	0.7180821	1.2177905	0.0000062
11	C	-3.6247155	0.0000645	-3.6528197		48	C	1.3965208	2.4782584	0.0000388
12	C	-5.0461006	0.0000691	-3.654729		49	C	0.7107768	3.6526538	0.0000504
13	C	-5.7363907	0.0000474	-2.4822302		50	C	-0.7107795	3.6526533	0.0000299
14	C	-5.0577905	0.00002	-1.2219856		51	C	-1.3965227	2.4782574	-0.0000026
15	C	-7.2585021	0.0000004	-0.0001467		52	C	-0.7180831	1.21779	-0.0000169
16	C	-7.8609505	1.2954994	-0.0000763		53	C	-1.4196481	-0.0000007	-0.0000505
17	C	-9.2648466	1.6101303	-0.0000029		54	C	-0.7180823	-1.217791	-0.0000609
18	C	-9.6742401	2.9625011	0.000134		55	C	-1.396521	-2.4782589	-0.0000967
19	C	-8.8024946	4.0291647	0.0001772		56	C	-0.710777	-3.6526542	-0.0001064
20	C	-7.4220859	3.7148064	0.0000711		57	C	0.7107793	-3.6526537	-0.0000807
21	C	-6.9805118	2.4246335	-0.0000435		58	C	1.3965225	-2.4782579	-0.0000473
22	C	-7.860959	-1.2954953	-0.0002401		59	C	0.7180828	-1.2177905	-0.0000373
23	C	-9.2648577	-1.6101159	-0.0003299		60	H	2.4801347	2.4822915	0.0000548
24	C	-9.6742617	-2.9624834	-0.0004961		61	H	1.2457929	4.5967295	0.0000758
25	C	-8.8025248	-4.029154	-0.0005566		62	H	-1.2457963	4.5967286	0.0000405
26	C	-7.4221138	-3.7148064	-0.0004289		63	H	-2.4801366	2.4822897	-0.0000179
27	C	-6.9805294	-2.424637	-0.0002819		64	H	-2.4801349	-2.482292	-0.0001163
28	C	-10.339466	-0.6775454	-0.0002394		65	H	-1.2457931	-4.5967299	-0.0001337
29	C	-10.3394615	0.6775674	-0.0000729		66	H	1.2457961	-4.596729	-0.0000881
30	H	-11.323748	-1.1362005	-0.0002977		67	H	2.4801364	-2.4822902	-0.0000278
31	H	-11.3237403	1.1362292	-0.0000042		68	C	5.7492493	0.0000008	0.0000636
32	H	-6.8208884	-0.0000512	2.4996944		69	C	5.0578301	0.000021	-1.2218209
33	H	-5.5806389	-0.0000875	4.5988588		70	C	5.7364713	0.0000376	-2.4820435
34	H	-3.0887683	-0.0000787	4.5957463		71	C	5.0462195	0.0000569	-3.6545647
35	H	-1.8578007	-0.0000389	2.4756444		72	C	3.6248343	0.0000605	-3.6527018
36	H	-1.8577173	0.0000384	-2.4757606		73	C	2.9410503	0.0000427	-2.47694
37	H	-3.0886138	0.0000806	-4.5959037		74	C	3.6218403	0.0000209	-1.2180089

Tag	Symbol	X	Y	Z
75	C	2.9187156	0.0000006	0.0000193
76	C	3.6218022	-0.0000204	1.2180693
77	C	2.9409724	-0.0000431	2.476979
78	C	3.6247193	-0.000062	3.6527623
79	C	5.0461044	-0.0000589	3.65467
80	C	5.7363933	-0.0000388	2.4821707
81	C	5.057792	-0.0000209	1.2219265
82	C	7.2585019	-0.0000006	0.0000895
83	C	7.8609581	1.2954954	0.0001096
84	C	9.2648565	1.6101167	0.0001143
85	C	9.6742599	2.9624845	0.0000899
86	C	8.8025222	4.0291545	0.0000749
87	C	7.4221114	3.7148062	0.0000952
88	C	6.9805278	2.4246365	0.0001113
89	C	7.8609508	-1.2954992	0.0000858
90	C	9.2648471	-1.6101295	0.000154
91	C	9.6742413	-2.9625001	0.0001767
92	C	8.8024963	-4.0291642	0.0001211
93	C	7.4220875	-3.7148065	0.0000344
94	C	6.9805127	-2.4246338	0.0000205
95	C	10.3394616	-0.677566	0.0001941
96	C	10.3394654	0.6775468	0.0001611
97	H	11.3237406	-1.1362274	0.0002451
98	H	11.3237472	1.1362024	0.0001821
99	H	6.8208855	0.000034	-2.4997544
100	H	5.5806339	0.0000695	-4.5989182
101	H	3.0887633	0.0000768	-4.5958032
102	H	1.8577977	0.0000443	-2.4757001
103	H	1.8577199	-0.0000446	2.4757049
104	H	3.0886186	-0.000079	4.5958468
105	H	5.5804891	-0.0000724	4.5990404
106	H	6.8208068	-0.0000354	2.4999162
107	H	10.7404811	3.1487321	0.0000877
108	H	6.6893108	4.5135323	0.0000976
109	H	5.9157588	2.2507616	0.0001249
110	H	10.7404612	-3.1487549	0.0002361
111	H	6.6892814	-4.5135276	-0.0000212
112	H	5.9157449	-2.250751	-0.0000457

Tag	Symbol	X	Y	Z
113	C	9.2555775	5.4846411	0.0000421
114	C	8.6990793	6.1774459	1.2592797
115	H	9.0093204	7.2263369	1.2675238
116	H	7.6061612	6.1570343	1.2950266
117	H	9.0780122	5.7065804	2.171458
118	C	8.6990594	6.1773903	-1.2592177
119	H	9.0093	7.226281	-1.2675128
120	H	9.0779782	5.7064846	-2.171381
121	H	7.6061407	6.1569762	-1.2949462
122	C	10.7818517	5.6216974	0.000027
123	H	11.2344819	5.171637	0.8892916
124	H	11.2344684	5.171591	-0.8892213
125	H	11.0505394	6.6811843	-0.0000022
126	C	9.2555417	-5.4846538	0.0001472
127	C	8.6989381	-6.1774312	1.2593534
128	H	7.6060174	-6.1570077	1.295014
129	H	9.0091682	-7.2263251	1.2676401
130	H	9.0778041	-5.7065535	2.1715531
131	C	8.6991197	-6.1774228	-1.259144
132	H	9.0093573	-7.2263148	-1.2673966
133	H	7.606204	-6.1570061	-1.2949588
134	H	9.0781117	-5.7065344	-2.1712858
135	C	10.781815	-5.6217203	0.0002569
136	H	11.2345062	-5.171631	-0.8889622
137	H	11.2343769	-5.171649	0.8895507
138	H	11.0504955	-6.6812091	0.0002658
139	C	-9.2555808	-5.4846402	-0.0007465
140	C	-8.6991631	-6.1775579	1.2584647
141	H	-9.0094073	-7.2264489	1.2665964
142	H	-7.6062472	-6.1571523	1.294282
143	H	-9.0781521	-5.7067723	2.1706609
144	C	-8.6989831	-6.1772772	-1.2600327
145	H	-9.0778454	-5.7062912	-2.172178
146	H	-7.6060622	-6.1568583	-1.2956909
147	H	-9.0092218	-7.2261676	-1.2684402
148	C	-10.7818552	-5.6216956	-0.0008708
149	H	-11.2345419	-5.1717119	0.8884039
150	H	-11.2344148	-5.171512	-0.890109

Tag	Symbol	X	Y	Z
151	H	-11.0505434	-6.6811823	-0.0010087
152	C	-9.2555392	5.4846546	0.0003286
153	C	-8.6989347	6.1773219	1.259595
154	H	-7.6060139	6.156893	1.295254
155	H	-9.0091627	7.2262157	1.2679724
156	H	-9.0778018	5.7063659	2.1717538
157	C	-8.6991174	6.1775332	-1.2589024
158	H	-7.6062017	6.1571207	-1.2947188
159	H	-9.0781088	5.7067234	-2.1710851
160	H	-9.0093561	7.2264256	-1.2670643
161	C	-10.7818125	5.6217219	0.0004508
162	H	-11.2345044	5.1717079	-0.888806
163	H	-11.234374	5.1715757	0.889707
164	H	-11.0504924	6.6812108	0.0005493

4^{2+}: CAM-B3LYP/6-31G(d)

SCF Done: E(RCAM-B3LYP) = -3934.76184940 A.U. after 6 cycles									
**Tag**	**Symbol**	**X**	**Y**	**Z**	**Tag**	**Symbol**	**X**	**Y**	**Z**
1	C	7.9182273	-0.0000288	-0.0000067	38	H	7.7481826	-3.2521946	-3.2520381
2	C	7.2270758	0.8640991	0.8640793	39	H	8.9897699	-1.7685569	-1.7684708
3	C	7.9052469	1.7553845	1.7553119	40	H	12.9085703	-2.2269356	2.2262762
4	C	7.2140214	2.584196	2.5840811	41	H	8.8567256	-3.1915084	3.1906974
5	C	5.7926973	2.5826036	2.5824937	42	H	8.084108	-1.5908554	1.5904701
6	C	5.1095701	1.7508908	1.7508283	43	H	12.9084154	2.2270261	-2.2264199
7	C	5.7912934	0.8610652	0.8610499	44	H	8.8564975	3.191484	-3.1906555
8	C	5.0874968	-0.0000416	-0.000012	45	H	8.0839988	1.5907819	-1.5904122
9	C	5.7913044	-0.8611383	-0.8610749	46	C	0.7492705	-0.0000498	-0.0000221
10	C	5.1095922	-1.7509664	-1.7508594	47	C	1.4520663	-0.8606753	0.8606565
11	C	5.7927298	-2.5826671	-2.5825284	48	C	0.7715061	-1.7511884	1.7512227
12	C	7.214054	-2.5842435	-2.5841138	49	C	1.4558472	-2.582172	2.5822573
13	C	7.9052692	-1.7554287	-1.7553393	50	C	2.8774809	-2.5827552	2.5828419
14	C	7.227087	-0.864156	-0.8641023	51	C	3.5652399	-1.753061	1.7530997
15	C	9.4266266	-0.0000154	-0.0000127	52	C	2.8880521	-0.861288	0.8612735
16	C	10.0296177	-0.9162209	0.9159759	53	C	3.5890788	-0.0000461	-0.0000162
17	C	11.4332104	-1.1386973	1.1383642	54	C	2.8880534	0.8611935	-0.8613089
18	C	11.8423559	-2.0950993	2.0945027	55	C	3.565243	1.7529686	-1.7531315
19	C	10.9701644	-2.8492583	2.8484861	56	C	2.8774856	2.5826617	-2.5828762
20	C	9.5899988	-2.62696	2.6262792	57	C	1.4558519	2.5820755	-2.5822973
21	C	9.1487526	-1.7143778	1.7139413	58	C	0.7715091	1.7510898	-1.751266
22	C	10.0295603	0.9162108	-0.9160122	59	C	1.4520677	0.8605773	-0.860698
23	C	11.4331358	1.1387296	-1.1384562	60	H	-0.3119839	-1.7517897	1.751823
24	C	11.8422109	2.0951556	-2.0946011	61	H	0.9194729	-3.249158	3.2492829
25	C	10.9699625	2.8492991	-2.8485352	62	H	3.4118298	-3.2507162	3.2508427
26	C	9.5898128	2.6269534	-2.6262741	63	H	4.6490282	-1.7574495	1.7574894
27	C	9.1486346	1.7143445	-1.7139301	64	H	4.6490314	1.7573595	-1.7575166
28	C	12.5079547	0.4791282	-0.4790441	65	H	3.4118358	3.2506242	-3.2508743
29	C	12.5079855	-0.4790794	0.4788964	66	H	0.9194788	3.249061	-3.2493243
30	H	13.4921774	0.8036227	-0.8034577	67	H	-0.3119809	1.751689	-1.7518704
31	H	13.4922294	-0.8035561	0.8032634	68	C	-0.7492705	-0.0000507	-0.0000243
32	H	8.9897476	1.7685243	1.7684449	69	C	-1.4520687	0.8606234	0.8606037
33	H	7.7481416	3.2521567	3.2520025	70	C	-0.7715113	1.7511871	1.7511215
34	H	5.2560038	3.2492369	3.2490908	71	C	-1.4558552	2.5822172	2.5821074
35	H	4.0263646	1.7487922	1.7487311	72	C	-2.877489	2.5827995	2.5826883
36	H	4.0263867	-1.7488794	-1.7487643	73	C	-3.5652452	1.7530588	1.7529904
37	H	5.2560448	-3.2493026	-3.24913	74	C	-2.8880543	0.8612366	0.8612159

Tag	Symbol	X	Y	Z
75	C	-3.5890789	-0.0000512	-0.000029
76	C	-2.8880513	-0.8613393	-0.861272
77	C	-3.5652377	-1.7531619	-1.7530497
78	C	-2.8774776	-2.5829018	-2.5827453
79	C	-1.455844	-2.5823184	-2.5821591
80	C	-0.7715041	-1.7512879	-1.7511703
81	C	-1.4520654	-0.8607252	-0.8606547
82	H	-0.9194831	3.2492411	3.2490968
83	H	-3.4118401	3.2507973	3.2506504
84	H	-4.6490337	1.7574468	1.7573767
85	H	-4.649026	-1.7575509	-1.7574406
86	H	-3.4118255	-3.2508998	-3.2507099
87	H	-0.9194687	-3.2493415	-3.2491469
88	H	0.311986	-1.751889	-1.7517694
89	C	-7.9182273	-0.0000362	-0.0000252
90	C	-7.2270837	-0.8641286	0.8641026
91	C	-7.9052629	-1.7553601	1.755383
92	C	-7.2140448	-2.5841399	2.5841901
93	C	-5.7927207	-2.5825654	2.5825977
94	C	-5.109586	-1.750901	1.7508899
95	C	-5.7913013	-0.8611112	0.8610696
96	C	-5.0874969	-0.0000488	-0.0000304
97	C	-5.7912968	0.8610313	-0.8611162
98	C	-5.1095766	1.7508258	-1.7509281
99	C	-5.7927067	2.5825141	-2.5826156
100	C	-7.2140309	2.5841109	-2.5841934
101	C	-7.9052535	1.7553284	-1.7553929
102	C	-7.2270793	0.8640709	-0.8641346
103	C	-9.4266266	-0.0000066	-0.000001
104	C	-10.029506	0.9160787	0.916169
105	C	-11.4330651	1.1386081	1.1386957
106	C	-11.842073	2.0948652	2.0950387
107	C	-10.9697703	2.8488252	2.8490944
108	C	-9.5896356	2.6264671	2.6267488
109	C	-9.1485224	1.7140175	1.7142142
110	C	-10.0296719	-0.9160443	-0.9161279
111	C	-11.4332811	-1.1384177	-1.1385258
112	C	-11.8424937	-2.0946301	-2.0948249
113	C	-10.9703564	-2.8486964	-2.8489627
114	C	-9.5901758	-2.6265001	-2.6267507
115	C	-9.1488647	-1.7140982	-1.7142637
116	C	-12.5080146	-0.4788625	-0.4789269
117	C	-12.5079256	0.4791732	0.4791855
118	H	-13.4922787	-0.8032345	-0.8033376
119	H	-13.4921281	0.8036515	0.8036762
120	H	-8.9897636	-1.7684841	1.768522
121	H	-7.7481711	-3.2520606	3.2521467
122	H	-5.2560332	-3.2491714	3.2492268
123	H	-4.0263804	-1.7488135	1.7487916
124	H	-4.0263711	1.748723	-1.748839
125	H	-5.2560157	3.2491232	-3.2492388
126	H	-7.7481535	3.2520505	-3.252134
127	H	-8.9897541	1.7684685	-1.7685219
128	H	-12.908268	2.2267518	2.226917
129	H	-8.8562802	3.1908568	3.1912189
130	H	-8.083895	1.5904271	1.5906408
131	H	-12.9087174	-2.2263899	-2.2265993
132	H	-8.8569426	-3.1909811	-3.1912884
133	H	-8.0842117	-1.5906453	-1.5908061
134	H	0.3119787	1.7517892	1.7517245
135	C	11.4229419	-3.8787136	3.8776531
136	C	10.8664083	-5.2589296	3.4770407
137	H	11.1763757	-6.0066315	4.2128687
138	H	9.7735455	-5.2694942	3.4370144
139	H	11.2453529	-5.5708125	2.4990176
140	C	10.8664789	-3.478443	5.2579968
141	H	11.2454717	-2.5005156	5.5701209
142	H	9.773618	-3.4383801	5.2686168
143	H	11.17645	-4.2144783	6.0054932
144	C	12.9491649	-3.9759506	3.974804
145	H	13.4017717	-4.2862766	3.027661
146	H	13.4018221	-3.0289063	4.2853576
147	H	13.2176365	-4.7252836	4.7239318
148	C	11.4226636	3.8787803	-3.87771
149	C	10.8661203	3.4785274	-5.2580264
150	H	9.7732594	3.4384418	-5.2685761
151	H	11.1760279	4.214585	-6.0055272
152	H	11.2451131	2.5006144	-5.5701956

Tag	Symbol	X	Y	Z
153	C	10.8661377	5.2589823	-3.4770386
154	H	9.773277	5.2695364	-3.4369541
155	H	11.2451317	5.5708483	-2.4990292
156	H	11.1760588	6.0067029	-4.2128672
157	C	12.9488794	3.9760384	-3.9749526
158	H	13.4015422	4.2863243	-3.0278232
159	H	13.4015277	3.029014	-4.2855797
160	H	13.2172956	4.7254093	-4.7240623
161	C	-11.4232067	-3.8779452	-3.8783043
162	C	-11.4223986	3.8781211	3.8784865
163	C	-10.8658429	3.4775661	5.2587102
164	H	-9.7729836	3.4374328	5.2692296
165	H	-11.1757041	4.2134856	6.0063661
166	H	-11.2448703	2.499606	5.5706899
167	C	-10.8658159	5.2583766	3.4780779
168	H	-9.7729558	5.2688839	3.4379638
169	H	-11.2448224	5.5704589	2.5001424
170	H	-11.1756783	6.0059639	4.2140665
171	C	-12.9486075	3.9754319	3.9757839
172	H	-13.4012762	4.2859517	3.028734
173	H	-13.4012947	3.0283599	4.286209
174	H	-13.2169709	4.7246488	4.7250666
175	C	-10.866718	-5.2582569	-3.4779593
176	H	-11.1767338	-6.0058114	-4.2139167
177	H	-9.7738546	-5.268872	-3.4379626
178	H	-11.2456503	-5.5703043	-2.4999839
179	C	-10.8667677	-3.4774465	-5.2585916
180	H	-11.2457259	-2.4994446	-5.5705244
181	H	-9.7739054	-3.4374312	-5.2692381
182	H	-11.1767954	-4.2133305	-6.0062134
183	C	-12.9494365	-3.9751015	-3.9754285
184	H	-13.4020277	-4.2855976	-3.0283339
185	H	-13.4020646	-3.027977	-4.28578
186	H	-13.2179609	-4.7242748	-4.7246971

5^{2+}: CAM-B3LYP/6-31G(d)

SCF Done: E(RCAM-B3LYP) = -4472.78282776 A.U. after 6 cycles									
**Tag**	**Symbol**	**X**	**Y**	**Z**	**Tag**	**Symbol**	**X**	**Y**	**Z**
1	C	10.0876438	0.0000011	-0.0001653	38	H	9.9166787	-0.0011174	-4.5995169
2	C	9.3967552	0.0002998	1.2220257	39	H	11.1591249	-0.000611	-2.5019594
3	C	10.0746518	0.0006065	2.4825787	40	H	15.0772017	-3.1493634	0.0002125
4	C	9.3828768	0.0008894	3.6544339	41	H	11.0248505	-4.5123813	0.0005878
5	C	7.9615902	0.0008852	3.6520136	42	H	10.25301	-2.2484823	0.0002568
6	C	7.2788586	0.0005989	2.4755675	43	H	15.0772061	3.1493594	-0.0006867
7	C	7.9610884	0.0002967	1.2175113	44	H	11.0248568	4.512383	-0.0009745
8	C	7.2569567	0.0000021	-0.0001185	45	H	10.253013	2.2484853	-0.0006117
9	C	7.9610481	-0.000293	-1.2177718	46	C	2.9186654	0.0000034	-0.0000474
10	C	7.2787769	-0.0005948	-2.4758054	47	C	3.6222804	-1.2167724	0.000208
11	C	7.9614698	-0.0008817	-3.652274	48	C	2.9409121	-2.4757238	0.0004938
12	C	9.3827562	-0.0008869	-3.6547413	49	C	3.6245284	-3.6513675	0.0007426
13	C	10.0745698	-0.0006045	-2.4829088	50	C	5.0461621	-3.6525691	0.0007231
14	C	9.3967147	-0.0002972	-1.2223336	51	C	5.7348058	-2.4796047	0.0004545
15	C	11.5955538	0.0000004	-0.0001894	52	C	5.058149	-1.2180977	0.0001863
16	C	12.1988203	-1.2956109	0.000027	53	C	5.7588753	0.0000026	-0.0000939
17	C	13.6021966	-1.6102527	0.0000312	54	C	5.0581497	1.2181032	-0.000351
18	C	14.0110295	-2.962677	0.0002219	55	C	5.7348072	2.4796098	-0.0006412
19	C	13.1384189	-4.0288173	0.000423	56	C	5.0461642	3.6525746	-0.0008872
20	C	11.7584428	-3.7143781	0.0004316	57	C	3.6245305	3.6513739	-0.0008603
21	C	11.3175457	-2.4237928	0.0002427	58	C	2.9409135	2.4757305	-0.0005893
22	C	12.198822	1.2956109	-0.0004254	59	C	3.6222811	1.2167787	-0.0003258
23	C	13.6021988	1.6102508	-0.0004634	60	H	1.8574986	-2.4747388	0.000508
24	C	14.0110335	2.9626746	-0.0006707	61	H	3.0875328	-4.5942945	0.0009571
25	C	13.1384245	4.0288161	-0.0008588	62	H	5.5802624	-4.5974925	0.0009238
26	C	11.758448	3.7143788	-0.0008321	63	H	6.8186949	-2.4867286	0.0004398
27	C	11.317549	2.4237942	-0.0006247	64	H	6.8186963	2.4867331	-0.0006617
28	C	14.6770718	0.6774086	-0.0003201	65	H	5.580265	4.5974977	-0.0011052
29	C	14.6770709	-0.6774119	-0.0001286	66	H	3.0875354	4.5943011	-0.0010572
30	H	15.6612859	1.1363129	-0.0003892	67	H	1.8575	2.4747461	-0.0005681
31	H	15.6612844	-1.1363176	-0.0000768	68	C	1.4203583	0.0000038	-0.0000229
32	H	11.1592074	0.0006122	2.5015937	69	C	0.7179687	0.0002597	1.2172334
33	H	9.9168304	0.0011194	4.5991919	70	C	1.397209	0.0005251	2.4774588
34	H	7.4245237	0.0011116	4.5945256	71	C	0.7109447	0.0007714	3.6516826
35	H	6.1956843	0.0005942	2.4715944	72	C	-0.7108253	0.0007697	3.6517058
36	H	6.1956027	-0.0005893	-2.4717967	73	C	-1.397128	0.0005225	2.4775045
37	H	7.4243721	-0.0011077	-4.5947684	74	C	-0.7179289	0.0002593	1.2172568

Tag	Symbol	X	Y	Z
75	C	-1.4203582	0.0000043	0.0000235
76	C	-0.7179687	-0.0002509	-1.2172328
77	C	-1.3972089	-0.0005138	-2.4774583
78	C	-0.7109446	-0.0007612	-3.6516821
79	C	0.7108254	-0.0007635	-3.6517053
80	C	1.3971281	-0.0005174	-2.4775039
81	C	0.717929	-0.0002518	-1.2172563
82	H	1.2466758	0.0009703	4.5955517
83	H	-1.2465256	0.0009669	4.5955924
84	H	-2.4807679	0.0005211	2.4804331
85	H	-2.4808489	-0.000512	-2.4803515
86	H	-1.2466757	-0.0009582	-4.5955512
87	H	1.2465257	-0.0009626	-4.5955919
88	H	2.480768	-0.0005188	-2.4804326
89	C	-10.0876438	0.0000037	0.000166
90	C	-9.3967551	-0.0002535	-1.2220252
91	C	-10.0746517	-0.0005016	-2.4825782
92	C	-9.3828767	-0.0007407	-3.6544334
93	C	-7.9615901	-0.0007455	-3.6520131
94	C	-7.2788585	-0.0005065	-2.475567
95	C	-7.9610884	-0.0002491	-1.2175108
96	C	-7.2569567	0.0000047	0.000119
97	C	-7.961048	0.0002567	1.2177723
98	C	-7.2787766	0.0005136	2.475806
99	C	-7.9614695	0.00075	3.6522746
100	C	-9.382756	0.0007431	3.6547419
101	C	-10.0745696	0.0005045	2.4829094
102	C	-9.3967146	0.000259	1.2223342
103	C	-11.5955537	0.0000005	0.0001898
104	C	-12.1988141	-1.2956131	0.0003374
105	C	-13.6021886	-1.6102625	0.0002526
106	C	-14.0110137	-2.9626892	0.0002651
107	C	-13.138397	-4.0288245	0.0004138
108	C	-11.7584226	-3.7143774	0.0005748
109	C	-11.3175329	-2.4237896	0.000533
110	C	-12.1988282	1.2956087	0.0000621
111	C	-13.6022069	1.6102411	0.0001783
112	C	-14.0110494	2.9626624	0.0001814
113	C	-13.1384467	4.028809	0.0000218
114	C	-11.7584685	3.7143797	-0.0001715
115	C	-11.317562	2.4237976	-0.0001479
116	C	-14.6770752	0.6773932	0.0002392
117	C	-14.6770676	-0.6774272	0.0002071
118	H	-15.6612916	1.1362926	0.0002729
119	H	-15.6612788	-1.1363378	0.0001895
120	H	-11.1592073	-0.0004945	-2.5015933
121	H	-9.9168303	-0.0009268	-4.5991914
122	H	-7.4245235	-0.0009375	-4.5945251
123	H	-6.1956842	-0.0005047	-2.4715939
124	H	-6.1956025	0.0005132	2.4717971
125	H	-7.4243718	0.0009416	4.5947689
126	H	-9.9166784	0.0009273	4.5995175
127	H	-11.1591247	0.000496	2.5019601
128	H	-15.0771849	-3.1493817	0.0001732
129	H	-11.0248258	-4.5123764	0.0007288
130	H	-10.2529982	-2.2484724	0.0006568
131	H	-15.077223	3.149341	0.0002968
132	H	-11.024882	4.5123882	-0.0003375
133	H	-10.2530251	2.2484956	-0.0002967
134	C	-2.9186654	0.0000045	0.0000479
135	C	-3.6222808	-1.216771	0.000311
136	C	-2.9409129	-2.4757227	0.0005608
137	C	-3.6245298	-3.6513662	0.0008156
138	C	-5.0461635	-3.6525671	0.0008381
139	C	-5.7348067	-2.4796024	0.0006058
140	C	-5.0581494	-1.2180958	0.0003346
141	C	-5.7588752	0.0000048	0.0000943
142	C	-5.0581492	1.2181052	-0.000169
143	C	-5.7348061	2.4796121	-0.0004183
144	C	-5.0461627	3.6525766	-0.0006732
145	C	-3.624529	3.6513753	-0.0006969
146	C	-2.9409124	2.4757317	-0.0004644
147	C	-3.6222805	1.2167801	-0.0001923
148	H	-1.8574995	-2.4747381	0.0005428
149	H	-3.0875346	-4.5942933	0.0010025
150	H	-5.5802642	-4.5974903	0.0010423
151	H	-6.8186959	-2.4867259	0.0006234
152	H	-6.8186953	2.4867358	-0.0004008

Tag	Symbol	X	Y	Z
153	H	-5.5802631	4.5975	-0.0008602
154	H	-3.0875335	4.5943023	-0.0009014
155	H	-1.8574989	2.4747468	-0.0004816
156	H	2.480849	0.000526	2.4803521
157	C	13.5907339	-5.4846999	0.0006216
158	C	13.0341538	-6.177162	1.259917
159	H	11.9413351	-6.1557319	1.2958567
160	H	13.3436352	-7.2263436	1.2684004
161	H	13.4133672	-5.7061037	2.1719057
162	C	13.0339409	-6.177562	-1.2583596
163	H	11.9411168	-6.1561131	-1.2941343
164	H	13.4130234	-5.7068126	-2.1705622
165	H	13.3433938	-7.2267544	-1.2665466
166	C	15.116919	-5.6227343	0.0005158
167	H	15.5696184	-5.1727988	-0.8887964
168	H	15.5697721	-5.1724464	0.8895716
169	H	15.3850004	-6.6824382	0.0007014
170	C	13.5907416	5.484698	-0.0010755
171	C	13.0341458	6.1771514	-1.2603688
172	H	11.9413265	6.1557259	-1.2962924
173	H	13.3436316	7.2263316	-1.2688659
174	H	13.4133438	5.7060835	-2.1723589
175	C	13.0339663	6.1775704	1.2579079
176	H	11.9411427	6.15612	1.2936987
177	H	13.4130629	5.7068295	2.170109
178	H	13.3434178	7.2267633	1.266081
179	C	15.1169269	5.6227303	-0.0009914
180	H	15.5696369	5.1728107	0.8883235
181	H	15.569768	5.1724251	-0.8900446
182	H	15.3850097	6.6824338	-0.0011996
183	C	-13.5907722	5.4846884	0.0000397
184	C	-15.1169583	5.6227118	0.0003453
185	H	-15.5699158	5.1725194	-0.8887057
186	H	-15.5695468	5.1726744	0.8896624
187	H	-15.3850473	6.6824138	0.0003095
188	C	-13.0343489	6.1772982	-1.2592437
189	H	-11.9415343	6.1558849	-1.2953154
190	H	-13.4136651	5.7063383	-2.1712404
191	H	-13.3438432	7.2264774	-1.2675724

Tag	Symbol	X	Y	Z
192	C	-13.0338326	6.1774108	1.2590329
193	H	-13.4128058	5.7065579	2.1712275
194	H	-11.9410042	6.1559606	1.2946757
195	H	-13.3432875	7.2266014	1.2673743
196	C	-13.5907037	-5.4847097	0.0004113
197	C	-13.0342656	-6.1773027	1.2596975
198	H	-13.3437517	-7.2264842	1.2680387
199	H	-11.941451	-6.1558809	1.2957616
200	H	-13.4135791	-5.7063369	2.1716923
201	C	-15.1168879	-5.6227528	0.0001144
202	H	-15.569848	-5.1725422	0.8891549
203	H	-15.5694854	-5.1727452	-0.8892132
204	H	-15.3849632	-6.6824582	0.000179
205	C	-13.0337607	-6.1774345	-1.2585792
206	H	-13.4127485	-5.706597	-2.1707756
207	H	-11.9409328	-6.1559649	-1.2942294
208	H	-13.343197	-7.2266307	-1.2669082

6^{2+}: CAM-B3LYP/6-31G(d)

SCF Done: E(RCAM-B3LYP) = -5010.80250694 A.U. after 6 cycles

Tag	Symbol	X	Y	Z	Tag	Symbol	X	Y	Z
1	C	-0.0000007	-0.7490417	-0.0000205	38	H	3.2511787	-7.7490533	3.2509332
2	C	-0.8604292	-1.4522847	0.8604343	39	H	3.2484955	-5.2561458	3.2482445
3	C	-1.751224	-0.7722362	1.7512515	40	H	1.7490637	-4.0269271	1.7489201
4	C	-2.5817847	-1.457797	2.5818421	41	H	-1.7490616	-4.02695	-1.7489452
5	C	-2.5820492	-2.879595	2.5821161	42	H	-3.248495	-5.2561883	-3.2482514
6	C	-1.7520936	-3.5667808	1.7521386	43	H	-3.2511713	-7.7490958	-3.2509144
7	C	-0.8607491	-2.8881095	0.8607613	44	H	-1.7591053	-8.9883154	-1.7589612
8	C	0.0000012	-3.5902595	-0.000014	45	C	-0.0000055	-12.2569132	0.0000496
9	C	0.8607515	-2.8881125	-0.8607928	46	C	0.8642182	-11.5661542	-0.8642768
10	C	1.752099	-3.566786	-1.7521648	47	C	1.7555523	-12.2438956	-1.7557199
11	C	2.5820553	-2.8796027	-2.5821439	48	C	2.5840195	-11.5517593	-2.5842894
12	C	2.5817891	-1.4578049	-2.5818758	49	C	2.5822232	-10.1304977	-2.5824957
13	C	1.7512258	-0.7722418	-1.7512895	50	C	1.7502889	-9.4479887	-1.7504647
14	C	0.8604298	-1.4522876	-0.860472	51	C	0.8609138	-10.1305711	-0.8609817
15	H	-1.7521094	0.3113298	1.7521298	52	C	0.000003	-9.4262791	0.000021
16	H	-3.2489506	-0.921473	3.2490251	53	C	-0.8609128	-10.1305485	0.8610374
17	H	-3.2496511	-3.4150394	3.2497422	54	C	-1.7502845	-9.4479456	1.750508
18	H	-1.7549164	-4.6505094	1.7549675	55	C	-2.5822225	-10.130436	2.5825512
19	H	1.7549235	-4.6505146	-1.7549897	56	C	-2.5840268	-11.5516975	2.5843711
20	H	3.249659	-3.4150492	-3.2497664	57	C	-1.7555626	-12.2438529	1.7558142
21	H	3.2489558	-0.9214828	-3.2490595	58	C	-0.8642254	-11.5661303	0.8643598
22	H	1.75211	0.3113242	-1.7521725	59	C	-0.0000007	-13.764477	0.0000547
23	C	0.0000063	-7.9285029	0.000008	60	C	0.9162574	-14.3679095	0.9161184
24	C	0.8614416	-7.2280092	0.8613728	61	C	1.1388537	-15.7711677	1.1384634
25	C	1.7536152	-7.9043481	1.7534818	62	C	2.0953348	-16.1799136	2.0945939
26	C	2.5828709	-7.2151401	2.5826738	63	C	2.8491879	-15.3071399	2.8483731
27	C	2.5818777	-5.7935128	2.5816775	64	C	2.6266772	-13.9272476	2.6262381
28	C	1.7503605	-5.110311	1.7502202	65	C	1.713875	-13.4864787	1.713736
29	C	0.86033	-5.7922174	0.8602565	66	C	-0.9162516	-14.3679155	-0.9160119
30	C	0.0000041	-5.0882671	-0.0000082	67	C	-1.1387343	-15.7711746	-1.1384627
31	C	-0.8603212	-5.7922284	-0.8602648	68	C	-2.0951605	-16.1799251	-2.0946461
32	C	-1.7503561	-5.1103339	-1.7502329	69	C	-2.8490699	-15.3071547	-2.8483736
33	C	-2.5818741	-5.7935466	-2.5816806	70	C	-2.6267367	-13.9272617	-2.6260671
34	C	-2.5828636	-7.2151738	-2.582662	71	C	-1.7139715	-13.4864883	-1.7135297
35	C	-1.7536042	-7.9043709	-1.7534645	72	C	-0.4789675	-16.8461165	-0.4789205
36	C	-0.8614301	-7.2280203	-0.8613649	73	C	0.4791456	-16.8461135	0.4788688
37	H	1.7591206	-8.9882925	1.7589884	74	H	-0.803543	-17.8303134	-0.8034292

Tag	Symbol	X	Y	Z
75	H	0.8037775	-17.8303085	0.8033271
76	H	1.769236	-13.3284848	-1.7694072
77	H	3.2520949	-12.085592	-3.2524468
78	H	3.248543	-9.593206	-3.2488978
79	H	1.7471309	-8.3648229	-1.7473121
80	H	-1.747121	-8.3647798	1.7473358
81	H	-3.2485396	-9.5931287	3.2489435
82	H	-3.2521054	-12.0855142	3.2525381
83	H	-1.769249	-13.3284421	1.7695185
84	H	2.2274505	-17.2460954	2.22651
85	H	3.1908059	-13.1934626	3.1904152
86	H	1.5895941	-12.4219823	1.589719
87	H	-2.2271911	-17.2461074	-2.2266423
88	H	-3.1909722	-13.1934788	-3.1901403
89	H	-1.5898164	-12.4219913	-1.5893913
90	C	3.8789025	-15.7593666	3.8776582
91	C	3.4786484	-15.2028408	5.2579256
92	H	4.2148448	-15.5122916	6.00554
93	H	3.4380226	-14.1100464	5.2681797
94	H	2.5007822	-15.5820275	5.5700507
95	C	5.2589355	-15.2025758	3.476947
96	H	6.006884	-15.5120189	4.212805
97	H	5.5707395	-15.5815708	2.4989024
98	H	5.2689989	-14.1097742	3.4364731
99	C	3.9767139	-17.2855218	3.9752188
100	H	3.0297316	-17.7383612	4.2857328
101	H	4.2870357	-17.7381834	3.0280872
102	H	4.7261769	-17.5535441	4.7244347
103	C	-3.8786797	-15.7593859	-3.8777618
104	C	-5.2587808	-15.2026768	-3.4771882
105	H	-5.2689106	-14.1098772	-3.4366734
106	H	-6.0066284	-15.5121278	-4.2131476
107	H	-5.570677	-15.5817294	-2.4991969
108	C	-3.4782909	-15.2027813	-5.2579633
109	H	-4.2143859	-15.5122281	-6.0056771
110	H	-3.4377084	-14.1099849	-5.2681536
111	H	-2.5003706	-15.5819089	-5.5699963
112	C	-3.9764234	-17.2855415	-3.975386
113	H	-3.029427	-17.7383322	-4.2859323

Tag	Symbol	X	Y	Z
114	H	-4.2867118	-17.7382503	-3.0282673
115	H	-4.725881	-17.553566	-4.7246064
116	C	0.0000007	0.7490417	-0.0000205
117	C	0.8604292	1.4522847	0.8604343
118	C	-0.8604298	1.4522876	-0.860472
119	C	1.751224	0.7722362	1.7512515
120	C	0.8607491	2.8881095	0.8607613
121	C	-0.8607515	2.8881125	-0.8607928
122	C	-1.7512258	0.7722418	-1.7512895
123	C	2.5817847	1.457797	2.5818421
124	H	1.7521094	-0.3113298	1.7521298
125	C	1.7520936	3.5667808	1.7521386
126	C	-0.0000012	3.5902595	-0.000014
127	C	-1.752099	3.566786	-1.7521648
128	C	-2.5817891	1.4578049	-2.5818758
129	H	-1.75211	-0.3113242	-1.7521725
130	C	2.5820492	2.879595	2.5821161
131	H	3.2489506	0.921473	3.2490251
132	H	1.7549164	4.6505094	1.7549675
133	C	-0.0000041	5.0882671	-0.0000082
134	C	-2.5820553	2.8796027	-2.5821439
135	H	-1.7549235	4.6505146	-1.7549897
136	H	-3.2489558	0.9214828	-3.2490595
137	H	3.2496511	3.4150394	3.2497422
138	C	-0.86033	5.7922174	0.8602565
139	C	0.8603212	5.7922284	-0.8602648
140	H	-3.249659	3.4150492	-3.2497664
141	C	-0.8614416	7.2280092	0.8613728
142	C	-1.7503605	5.110311	1.7502202
143	C	1.7503561	5.1103339	-1.7502329
144	C	0.8614301	7.2280203	-0.8613649
145	C	-0.0000063	7.9285029	0.000008
146	C	-1.7536152	7.9043481	1.7534818
147	C	-2.5818777	5.7935128	2.5816775
148	H	-1.7490637	4.0269271	1.7489201
149	C	2.5818741	5.7935466	-2.5816806
150	H	1.7490616	4.02695	-1.7489452
151	C	1.7536042	7.9043709	-1.7534645
152	C	-0.000003	9.4262791	0.000021

Tag	Symbol	X	Y	Z
153	C	-2.5828709	7.2151401	2.5826738
154	H	-1.7591206	8.9882925	1.7589884
155	H	-3.2484955	5.2561458	3.2482445
156	C	2.5828636	7.2151738	-2.582662
157	H	3.248495	5.2561883	-3.2482514
158	H	1.7591053	8.9883154	-1.7589612
159	C	-0.8609138	10.1305711	-0.8609817
160	C	0.8609128	10.1305485	0.8610374
161	H	-3.2511787	7.7490533	3.2509332
162	H	3.2511713	7.7490958	-3.2509144
163	C	-0.8642182	11.5661542	-0.8642768
164	C	-1.7502889	9.4479887	-1.7504647
165	C	1.7502845	9.4479456	1.750508
166	C	0.8642254	11.5661303	0.8643598
167	C	0.0000055	12.2569132	0.0000496
168	C	-1.7555523	12.2438956	-1.7557199
169	C	-2.5822232	10.1304977	-2.5824957
170	H	-1.7471309	8.3648229	-1.7473121
171	C	2.5822225	10.130436	2.5825512
172	H	1.747121	8.3647798	1.7473358
173	C	1.7555626	12.2438529	1.7558142
174	C	0.0000007	13.764477	0.0000547
175	C	-2.5840195	11.5517593	-2.5842894
176	H	-1.769236	13.3284848	-1.7694072
177	H	-3.248543	9.593206	-3.2488978
178	C	2.5840268	11.5516975	2.5843711
179	H	3.2485396	9.5931287	3.2489435
180	H	1.769249	13.3284421	1.7695185
181	C	-0.9162574	14.3679095	0.9161184
182	C	0.9162516	14.3679155	-0.9160119
183	H	-3.2520949	12.085592	-3.2524468
184	H	3.2521054	12.0855142	3.2525381
185	C	-1.1388537	15.7711677	1.1384634
186	C	-1.713875	13.4864787	1.713736
187	C	1.1387343	15.7711746	-1.1384627
188	C	1.7139715	13.4864883	-1.7135297
189	C	-2.0953348	16.1799136	2.0945939
190	C	-0.4791456	16.8461135	0.4788688
191	C	-2.6266772	13.9272476	2.6262381

Tag	Symbol	X	Y	Z
192	H	-1.5895941	12.4219823	1.589719
193	C	2.0951605	16.1799251	-2.0946461
194	C	0.4789675	16.8461165	-0.4789205
195	C	2.6267367	13.9272617	-2.6260671
196	H	1.5898164	12.4219913	-1.5893913
197	C	-2.8491879	15.3071399	2.8483731
198	H	-2.2274505	17.2460954	2.22651
199	H	-0.8037775	17.8303085	0.8033271
200	H	-3.1908059	13.1934626	3.1904152
201	C	2.8490699	15.3071547	-2.8483736
202	H	2.2271911	17.2461074	-2.2266423
203	H	0.803543	17.8303134	-0.8034292
204	H	3.1909722	13.1934788	-3.1901403
205	C	-3.8789025	15.7593666	3.8776582
206	C	3.8786797	15.7593859	-3.8777618
207	C	-3.4786484	15.2028408	5.2579256
208	C	-5.2589355	15.2025758	3.476947
209	C	-3.9767139	17.2855218	3.9752188
210	C	5.2587808	15.2026768	-3.4771882
211	C	3.4782909	15.2027813	-5.2579633
212	C	3.9764234	17.2855415	-3.975386
213	H	-4.2148448	15.5122916	6.00554
214	H	-3.4380226	14.1100464	5.2681797
215	H	-2.5007822	15.5820275	5.5700507
216	H	-6.006884	15.5120189	4.212805
217	H	-5.5707395	15.5815708	2.4989024
218	H	-5.2689989	14.1097742	3.4364731
219	H	-3.0297316	17.7383612	4.2857328
220	H	-4.2870357	17.7381834	3.0280872
221	H	-4.7261769	17.5535441	4.7244347
222	H	5.2689106	14.1098772	-3.4366734
223	H	6.0066284	15.5121278	-4.2131476
224	H	5.570677	15.5817294	-2.4991969
225	H	4.2143859	15.5122281	-6.0056771
226	H	3.4377084	14.1099849	-5.2681536
227	H	2.5003706	15.5819089	-5.5699963
228	H	3.029427	17.7383322	-4.2859323
229	H	4.2867118	17.7382503	-3.0282673
230	H	4.725881	17.553566	-4.7246064

1T (triplet): UB3LYP/6-31G(d)

SCF Done: E(UB3LYP) = -2322.51738053 A.U. after 6 cycles									
Tag	Symbol	X	Y	Z	Tag	Symbol	X	Y	Z
1	C	1.4356531	-0.0000397	0.0000432	38	C	-4.5119495	4.074658	-0.0001768
2	C	0.7234199	-0.0000138	1.2209486	39	C	-5.3778184	2.9833314	0.0000454
3	C	1.4018482	0.0000037	2.4828605	40	C	-4.9619346	1.6356646	0.0001443
4	C	0.7113902	0.0000288	3.6652771	41	C	-6.0477302	0.6744797	0.0004218
5	C	-0.7113718	0.0000387	3.6652809	42	C	-6.0477643	-0.6743171	0.0004576
6	C	-1.4018366	0.0000234	2.4828682	43	H	-7.0340343	1.1353723	0.0006182
7	C	-0.7234157	-0.0000034	1.2209521	44	H	-7.034092	-1.1351592	0.0006786
8	C	-1.4356535	-0.0000206	0.0000492	45	C	6.0477429	0.6743745	0.0001051
9	C	-0.7234198	-0.000034	-1.2208574	46	C	6.0477501	-0.6744222	0.0000547
10	C	-1.4018482	-0.0000362	-2.4827688	47	H	7.0340563	1.1352473	0.0001606
11	C	-0.7113901	-0.0000496	-3.6651859	48	H	7.0340686	-1.1352841	0.0000743
12	C	0.7113716	-0.0000606	-3.6651895	49	H	2.4864857	-0.0000037	2.4847037
13	C	1.4018362	-0.0000576	-2.4827761	50	H	1.2499304	0.0000411	4.6092401
14	C	0.7234151	-0.0000444	-1.2208608	51	H	-1.2499069	0.0000585	4.6092467
15	C	2.9473891	-0.0000411	0.0000398	52	H	-2.4864741	0.0000308	2.4847186
16	C	3.5602599	-1.3190079	0.0000022	53	H	-2.4864858	-0.0000275	-2.4846117
17	C	4.961985	-1.6356419	-0.0000139	54	H	-1.2499305	-0.0000518	-4.6091487
18	C	5.3779157	-2.9832938	-0.0000812	55	H	1.249907	-0.0000708	-4.609155
19	C	4.5120855	-4.0746507	-0.0001234	56	H	2.4864736	-0.0000651	-2.4846262
20	C	3.1415155	-3.7608687	-0.0000918	57	H	6.4487793	-3.1564542	-0.0000978
21	C	2.6948431	-2.4525319	-0.0000316	58	H	2.3992439	-4.5546029	-0.0001159
22	C	3.5602465	1.3189295	0.0000635	59	H	1.6260887	-2.289854	-0.0000107
23	C	4.9619668	1.6355814	0.0000871	60	H	6.4487398	3.1564144	0.000121
24	C	5.3778787	2.9832394	0.0001038	61	H	2.399184	4.5545065	0.0000912
25	C	4.5120327	4.074584	0.0001019	62	H	1.6260606	2.2897441	0.0000636
26	C	3.1414669	3.7607829	0.0000885	63	H	-6.4488446	-3.156339	0.0003319
27	C	2.694813	2.4524399	0.0000715	64	H	-2.399339	-4.5545736	-0.0001433
28	C	-2.9473895	0.0000019	0.0000645	65	H	-1.6261362	-2.2898415	-0.0001166
29	C	-3.5602867	-1.3189533	0.0000923	66	H	-1.6260146	2.2897566	-0.0002853
30	C	-4.9620185	-1.6355586	0.0002371	67	H	-2.3990906	4.5545361	-0.0004453
31	C	-5.3779774	-2.983202	0.0002159	68	H	-6.4486758	3.1565285	0.0001591
32	C	-4.5121704	-4.0745773	0.0000689	69	C	4.9831268	5.5363147	0.0001163
33	C	-3.141594	-3.7608237	-0.0000415	70	C	4.4410725	6.2497072	1.2620781
34	C	-2.694894	-2.4524964	-0.0000259	71	H	4.7570107	7.3002115	1.2719969
35	C	-3.5602211	1.3189841	0.0000103	72	H	3.3469279	6.2304723	1.3025623
36	C	-2.6947637	2.4524758	-0.0001911	73	H	4.8173975	5.7723369	2.1739789
37	C	-3.1413902	3.7608281	-0.0002845	74	C	4.441073	6.2497277	-1.261835

Tag	Symbol	X	Y	Z
75	H	4.7570163	7.3002309	-1.2717398
76	H	4.8173908	5.7723676	-2.1737432
77	H	3.3469275	6.2305019	-1.3023136
78	C	6.5184884	5.6590343	0.0001162
79	H	6.9645581	5.196614	0.8880324
80	H	6.9645568	5.1966362	-0.8878123
81	H	6.8043606	6.7169756	0.0001297
82	C	4.983201	-5.5363745	-0.0001924
83	C	4.4412519	-6.2498101	1.2617929
84	H	3.3471098	-6.2306	1.3023523
85	H	4.7572151	-7.3003073	1.2716646
86	H	4.8176308	-5.7724511	2.1736769
87	C	4.4410643	-6.2497606	-1.2621207
88	H	4.8173127	-5.7723763	-2.1740454
89	H	4.7570162	-7.3002608	-1.2720738
90	H	3.3469156	-6.2305448	-1.3025236
91	C	6.5185644	-5.6590697	-0.0003127
92	H	6.9645564	-5.1966414	-0.8882639
93	H	6.9646958	-5.1966651	0.8875807
94	H	6.8044534	-6.7170065	-0.000348
95	C	-4.9833149	-5.5362922	0.0000307
96	C	-4.4411951	-6.2497824	1.2619078
97	H	-4.757168	-7.3002767	1.2717825
98	H	-3.3470471	-6.2305876	1.3023169
99	H	-4.8174392	-5.7724578	2.1738662
100	C	-4.441374	-6.2496443	-1.2620044
101	H	-4.75735	-7.3001373	-1.271951
102	H	-4.8177428	-5.7722161	-2.1738558
103	H	-3.3472307	-6.2304482	-1.3025594
104	C	-6.5186807	-5.6589613	0.0001324
105	H	-6.9646727	-5.1965667	0.888101
106	H	-6.9647966	-5.196509	-0.8877438
107	H	-6.8045877	-6.7168933	0.000118
108	C	-4.9830124	5.5363989	-0.0002976
109	C	-4.4408139	6.249942	1.2615158
110	H	-3.3466658	6.2306867	1.3018935
111	H	-4.7567259	7.3004552	1.27133
112	H	-4.8170583	5.7726999	2.1735172
113	C	-4.4410714	6.2496381	-1.2623966
114	H	-4.8174912	5.7721676	-2.1742047
115	H	-4.7569939	7.3001465	-1.272405
116	H	-3.3469303	6.2303836	-1.3029823
117	C	-6.5183713	5.6591529	-0.0001566
118	H	-6.9645411	5.1966532	-0.8879811
119	H	-6.9643605	5.1968548	0.8878636
120	H	-6.8042198	6.7171007	-0.000247

1F$_{anti,anti}$: B3LYP/6-31G(d)

SCF Done: E(RB3LYP) = -2322.55119663 A.U. after 15 cycles									
**Tag**	**Symbol**	**X**	**Y**	**Z**	**Tag**	**Symbol**	**X**	**Y**	**Z**
1	C	-1.3932326	-2.325944	2.4196614	38	C	2.4185356	-2.1399958	-0.7139266
2	H	-2.4772434	-2.3156731	2.4442281	39	C	4.2840245	-3.7059016	-0.81075
3	C	-0.7075142	-1.2288844	1.8782367	40	C	2.9720313	-3.3329279	-1.1588156
4	C	-0.6984209	-3.4115142	2.9459355	41	H	6.0297186	-3.0373145	0.2553598
5	C	0.6984152	-3.4115152	2.9459358	42	H	2.3642837	-3.9912386	-1.7733046
6	C	0.7075122	-1.2288855	1.8782368	43	H	1.3936373	-1.8924188	-0.9745031
7	C	1.3932287	-2.3259461	2.419662	44	H	6.1873362	-1.1681037	1.6736784
8	H	-1.2466865	-4.2477392	3.3717255	45	H	6.1873243	1.1680665	1.6736922
9	H	1.2466794	-4.247741	3.3717261	46	C	4.8589778	5.048084	-1.2973753
10	H	2.4772395	-2.3156768	2.4442292	47	C	4.8597338	5.076049	-2.844441
11	C	1.3974223	-0.0000101	1.3892193	48	C	3.9812353	6.2060864	-0.7654714
12	C	-1.3974224	-0.000008	1.3892192	49	C	6.3026193	5.2798298	-0.8129772
13	C	0.7075134	1.2289011	1.8781313	50	H	4.3716443	7.171043	-1.1116892
14	C	-0.7075116	1.2289022	1.8781313	51	H	2.9447428	6.1234761	-1.1087138
15	C	1.3932336	2.3260307	2.4194098	52	H	3.9695838	6.2172163	0.3303817
16	C	-1.3932301	2.3260328	2.41941	53	H	6.3680767	5.2966761	0.2809377
17	C	0.6984213	3.4116625	2.9455561	54	H	6.9869198	4.5090284	-1.1853169
18	C	-0.6984161	3.4116635	2.9455563	55	H	6.6640742	6.2470793	-1.180076
19	H	-1.2466801	4.2479466	3.3712342	56	H	5.2592047	6.0305457	-3.2088238
20	H	1.2466866	4.2479448	3.3712339	57	H	5.4811937	4.2697936	-3.250447
21	H	2.4772459	2.3157703	2.443955	58	H	3.8512873	4.9588216	-3.2548327
22	H	-2.4772424	2.3157739	2.4439554	59	C	4.8589785	-5.0481088	-1.297426
23	C	2.5087471	-0.0000245	0.6040042	60	C	6.3026301	-5.2798757	-0.8130317
24	C	3.1440682	-1.2484385	0.0902326	61	C	4.8597217	-5.076072	-2.8444837
25	C	3.144068	1.2483931	0.0902378	62	C	3.9812122	-6.2060608	-0.7654718
26	C	4.4835869	-1.5697472	0.4022929	63	H	6.664075	-6.2471281	-1.1801229
27	C	4.4835776	1.5696992	0.4023091	64	H	6.9869315	-4.5090755	-1.1853759
28	C	5.3699054	-0.6759956	1.1480241	65	H	6.3680733	-5.2967259	0.2808842
29	C	5.3699015	0.6759516	1.1480304	66	H	4.3715038	-7.1710339	-1.1117773
30	C	5.0078869	2.8070429	-0.0282386	67	H	3.9696913	-6.2172253	0.3303832
31	C	4.2839876	3.7058867	-0.8106941	68	H	2.9446861	-6.1233611	-1.1085914
32	C	2.4185155	2.1399665	-0.7138845	69	H	5.2590249	-6.0306341	-3.208885
33	C	2.9719888	3.3329101	-1.1587513	70	H	3.8513025	-4.9586783	-3.254887
34	H	6.0296865	3.0372919	0.2554033	71	H	5.4813091	-4.2699127	-3.2504877
35	H	2.3642377	3.9912302	-1.7732247	72	C	-2.5087471	-0.000021	0.6040042
36	H	1.3936159	1.8923665	-0.9744413	73	C	-3.1440663	1.248397	0.0902367
37	C	5.0079082	-2.8070734	-0.0282702	74	C	-3.14407	-1.2484346	0.0902334

Tag	Symbol	X	Y	Z
75	C	-4.4835755	1.5697051	0.4023077
76	C	-4.4835891	-1.5697411	0.402294
77	C	-5.3699005	0.6759594	1.1480299
78	C	-5.3699063	-0.6759878	1.1480246
79	C	-5.0079122	-2.8070669	-0.0282682
80	C	-4.2840298	-3.7058967	-0.8107475
81	C	-2.4185383	-2.1399938	-0.7139244
82	C	-2.9720356	-3.3329255	-1.1588125
83	H	-6.0297228	-3.0373065	0.2553623
84	H	-2.3642887	-3.991238	-1.7733001
85	H	-1.3936395	-1.8924187	-0.9745005
86	C	-5.0078831	2.8070491	-0.0282411
87	C	-2.4185127	2.1399687	-0.7138865
88	C	-4.2839827	3.7058912	-0.8106976
89	C	-2.9719843	3.3329126	-1.1587543
90	H	-6.0296823	3.0373	0.2554009
91	H	-2.3642324	3.9912314	-1.7732283
92	H	-1.3936134	1.8923671	-0.9744431
93	H	-6.1873225	1.1680759	1.6736915
94	H	-6.1873377	-1.1680944	1.6736796
95	C	-4.8589852	-5.0481039	-1.2974216
96	C	-3.9812196	-6.2060558	-0.7654663
97	C	-6.3026369	-5.279869	-0.8130263
98	C	-4.8597291	-5.0760692	-2.8444793
99	H	-6.6640815	-6.247123	-1.1801133
100	H	-6.3680801	-5.2967143	0.2808896
101	H	-6.9869384	-4.5090706	-1.1853741
102	H	-5.4813162	-4.26991	-3.2504841
103	H	-3.8513101	-4.9586767	-3.2548833
104	H	-5.2590332	-6.0306314	-3.2088791
105	H	-4.3715122	-7.1710292	-1.1117703
106	H	-2.9446936	-6.1233575	-1.1085866
107	H	-3.9696981	-6.2172189	0.3303887
108	C	-4.858971	5.0480889	-1.2973798
109	C	-6.3026131	5.2798359	-0.8129843
110	C	-4.8597243	5.0760539	-2.8444455
111	C	-3.9812285	6.2060908	-0.7654744
112	H	-6.6640656	6.2470872	-1.180081
113	H	-6.9869141	4.5090369	-1.1853276

Tag	Symbol	X	Y	Z
114	H	-6.3680728	5.2966791	0.2809306
115	H	-4.3716366	7.1710476	-1.1116923
116	H	-3.9695783	6.2172202	0.3303787
117	H	-2.9447356	6.1234801	-1.1087156
118	H	-5.2591932	6.0305511	-3.2088291
119	H	-3.8512772	4.958825	-3.2548355
120	H	-5.4811846	4.2697993	-3.2504526

1F$_{syn,anti}$: B3LYP/6-31G(d)

SCF Done: E(RB3LYP) = -2322.54341977 A.U. after 6 cycles

Tag	Symbol	X	Y	Z	Tag	Symbol	X	Y	Z
1	C	2.2271406	-1.5215512	3.0449371	38	C	2.2621303	1.8180042	-0.5299897
2	H	2.1493328	-2.5891953	3.2088335	39	C	3.9423072	3.5444307	-0.9069229
3	C	1.1766991	-0.8413071	2.4181228	40	C	3.4859984	2.2200424	-1.0476868
4	C	3.3578715	-0.8367622	3.4884972	41	H	3.3894552	5.4745551	-0.131837
5	C	3.4451512	0.5455057	3.3208078	42	H	4.1032462	1.4830552	-1.5539565
6	C	1.2644008	0.5622376	2.2516635	43	H	1.9489279	0.7826654	-0.6265513
7	C	2.4014709	1.238853	2.7111492	44	H	1.5396375	5.9708887	1.2292367
8	H	4.159486	-1.381524	3.9802664	45	H	-0.790986	6.1153473	1.2257721
9	H	4.3159403	1.0876578	3.6798746	46	C	-4.7802292	4.5757001	-1.4844092
10	H	2.4585922	2.3165052	2.6029032	47	C	-4.8289392	4.3355463	-3.0121759
11	C	0.0775366	1.2585397	1.6759686	48	C	-5.9810421	3.864228	-0.8164195
12	C	-0.0932561	-1.4858861	1.9683863	49	C	-4.9196031	6.0889035	-1.2326391
13	C	-1.1873425	0.7143832	2.2495399	50	H	-6.9258628	4.2526497	-1.2163813
14	C	-1.2743818	-0.6891821	2.4165962	51	H	-5.9639224	2.7835292	-0.9916185
15	C	-2.2327218	1.5267469	2.7068315	52	H	-5.9782839	4.0260974	0.2675417
16	C	-2.4021371	-1.2337905	3.041549	53	H	-4.918308	6.3261832	-0.1626256
17	C	-3.355258	0.9682854	3.3148766	54	H	-4.1142812	6.6581535	-1.7106233
18	C	-3.4401022	-0.4140601	3.4830269	55	H	-5.8683422	6.4463461	-1.6487852
19	H	-4.3038771	-0.8551907	3.9734198	56	H	-5.7630176	4.7297144	-3.4312842
20	H	-4.1528214	1.6142786	3.6722533	57	H	-3.9928816	4.8358745	-3.5140081
21	H	-2.1558572	2.6031182	2.5981583	58	H	-4.7767248	3.2699824	-3.2590947
22	H	-2.4576259	-2.3028279	3.2053109	59	C	5.3170679	3.9498041	-1.4679499
23	C	0.1397897	2.246209	0.7436488	60	C	5.6413831	5.434271	-1.2154459
24	C	1.422132	2.7168474	0.1447354	61	C	5.3415045	3.7047879	-2.9955107
25	C	-1.0730831	2.8714171	0.1414955	62	C	6.4182853	3.095793	-0.7954122
26	C	1.8275983	4.065797	0.2462025	63	H	6.6287152	5.6715755	-1.6276627
27	C	-1.3090587	4.2601642	0.2416583	64	H	4.9145115	6.098536	-1.6965586
28	C	0.9945481	5.1121498	0.8390671	65	H	5.6650862	5.6701676	-0.1453931
29	C	-0.3549368	5.19579	0.8370903	66	H	7.4053397	3.3643726	-1.1917969
30	C	-2.5182171	4.7852648	-0.2621947	67	H	6.4314697	3.2573501	0.2885169
31	C	-3.4682122	4.0036349	-0.9184148	68	H	6.2684981	2.0253968	-0.9705915
32	C	-2.0156488	2.0829307	-0.5357139	69	H	6.3186821	3.980347	-3.4110946
33	C	-3.1787548	2.632902	-1.0574706	70	H	5.1589702	2.6537084	-3.2425568
34	H	-2.6836779	5.850943	-0.1415083	71	H	4.5755413	4.304333	-3.5004813
35	H	-3.8806721	1.9776898	-1.5658671	72	C	-0.1576426	-2.5317171	1.1047235
36	H	-1.8323178	1.0168033	-0.6313008	73	C	1.0406419	-3.1629312	0.4746628
37	C	3.0939953	4.4374905	-0.2535652	74	C	-1.425024	-3.0132735	0.4778125

Tag	Symbol	X	Y	Z
75	C	1.3931722	-2.7790461	-0.8389205
76	C	-1.732611	-2.5903354	-0.8350175
77	C	0.5550625	-1.9032861	-1.6604677
78	C	-0.7973923	-1.8219488	-1.6591089
79	C	-2.9704908	-2.9605638	-1.399922
80	C	-3.8762317	-3.7987227	-0.7495806
81	C	-2.310449	-3.889219	1.1183443
82	C	-3.5048874	-4.2754579	0.5212239
83	H	-3.193421	-2.5902198	-2.3953273
84	H	-4.1646586	-4.9465703	1.0640899
85	H	-2.0579566	-4.2674044	2.1053168
86	C	2.575531	-3.2956613	-1.407779
87	C	1.8153487	-4.1404041	1.1117161
88	C	3.3749658	-4.2382426	-0.7609931
89	C	2.9524494	-4.6678384	0.5106011
90	H	2.8385875	-2.9540747	-2.4036621
91	H	3.5274828	-5.414738	1.0508525
92	H	1.5216669	-4.4867035	2.098969
93	H	1.079643	-1.353183	-2.4408847
94	H	-1.2538881	-1.2129652	-2.4386068
95	C	-5.2231199	-4.218356	-1.3657285
96	C	-6.3750873	-3.7626151	-0.4385795
97	C	-5.452977	-3.5957541	-2.7557596
98	C	-5.2646846	-5.7577962	-1.5149167
99	H	-6.4247928	-3.9168239	-3.147466
100	H	-5.4584487	-2.5004988	-2.7159496
101	H	-4.6878263	-3.9087309	-3.4752461
102	H	-4.4630717	-6.1091497	-2.1745349
103	H	-5.1495063	-6.2644353	-0.5509485
104	H	-6.2230109	-6.0739953	-1.9450867
105	H	-7.3438269	-4.0594844	-0.8591193
106	H	-6.2953728	-4.2065115	0.5592712
107	H	-6.3745706	-2.6731404	-0.3198475
108	C	4.6585713	-4.8178951	-1.3825147
109	C	4.9585789	-4.2244401	-2.7718818
110	C	4.5106092	-6.3504393	-1.5352669
111	C	5.8606213	-4.5093963	-0.4582739
112	H	5.8828774	-4.6605295	-3.16743
113	H	4.1589508	-4.4400354	-3.4896391

Tag	Symbol	X	Y	Z
114	H	5.0975489	-3.1381105	-2.7297428
115	H	6.784283	-4.9217566	-0.8828169
116	H	5.9942424	-3.428426	-0.3370941
117	H	5.7301931	-4.9428708	0.538818
118	H	5.4213457	-6.7806082	-1.969924
119	H	4.3377909	-6.8416352	-0.5719058
120	H	3.6695221	-6.5991688	-2.192412

2T (triplet): UB3LYP/6-31G(d)

SCF Done: E(UB3LYP) = -2860.84583276 A.U. after 6 cycles									
Tag	Symbol	X	Y	Z	Tag	Symbol	X	Y	Z
1	C	3.6188384	-0.0000065	0.0000052	38	H	3.437862	3.2510848	-3.2667124
2	C	2.9075062	-0.8614203	0.8655646	39	H	4.6719694	1.7511805	-1.7595827
3	C	3.5873895	-1.7509473	1.7593687	40	H	8.6305183	-2.2375966	-2.2266822
4	C	2.8985172	-2.5855731	2.5980292	41	H	4.5805441	-3.2281331	-3.2126478
5	C	1.4758395	-2.5862795	2.5987631	42	H	3.8075241	-1.623296	-1.6155779
6	C	0.7838224	-1.752932	1.7614058	43	H	8.6304751	2.237665	2.2266747
7	C	1.4607351	-0.8620995	0.866265	44	H	4.5804788	3.2281029	3.2126519
8	C	0.7511192	0.0000069	0.0000075	45	H	3.8074933	1.6232328	1.6155943
9	C	1.4607419	0.8621098	-0.8662478	46	C	-3.6188382	0.0000153	0.0000074
10	C	0.783835	1.7529513	-1.761384	47	C	-2.9075107	-0.8655459	-0.8614092
11	C	1.4758577	2.5862969	-2.5987389	48	C	-3.5873988	-1.7593517	-1.7509306
12	C	2.8985352	2.5855793	-2.5980063	49	C	-2.898531	-2.5980139	-2.5855588
13	C	3.5874021	1.750945	-1.7593496	50	C	-1.4758535	-2.5987476	-2.5862732
14	C	2.9075133	0.8614193	-0.8655484	51	C	-0.7838318	-1.7613891	-1.7529304
15	C	5.1303962	-0.0000092	0.0000067	52	C	-1.4607396	-0.866247	-0.8620956
16	C	5.7423037	-0.9350437	-0.9305313	53	C	-0.751119	0.000011	0.0000066
17	C	7.1439681	-1.1594843	-1.1538388	54	C	-1.4607369	0.8662711	0.8621083
18	C	7.5596754	-2.1147591	-2.1044774	55	C	-0.7838252	1.7614125	1.7529411
19	C	6.6935981	-2.8882076	-2.8742333	56	C	-1.4758433	2.5987733	2.5862842
20	C	5.3230013	-2.6656993	-2.6528763	57	C	-2.898521	2.5980423	2.5855728
21	C	4.8764105	-1.7382704	-1.7299375	58	C	-3.5873924	1.7593813	1.750947
22	C	5.7422887	0.9350293	0.9305477	59	C	-2.9075085	0.8655732	0.8614242
23	C	7.1439481	1.1595019	1.1538504	60	C	-5.130396	0.00002	0.0000173
24	C	7.5596348	2.1147958	2.104479	61	C	-5.742288	-0.9305423	0.9350354
25	C	6.6935403	2.8882284	2.8742318	62	C	-7.1439476	-1.1537891	1.1595632
26	C	5.3229482	2.6656838	2.6528818	63	C	-7.5596352	-2.1044633	2.1148113
27	C	4.8763774	1.7382371	1.7299513	64	C	-6.6935415	-2.8743118	2.8881494
28	C	8.2296167	0.4780814	0.4757275	65	C	-5.3229491	-2.6530095	2.6655584
29	C	8.2296247	-0.4780519	-0.475708	66	C	-4.8763777	-1.7300362	1.7381548
30	H	9.2159469	0.8047398	0.8007781	67	C	-5.7423039	0.9305654	-0.9350092
31	H	9.2159605	-0.8047006	-0.8007514	68	C	-7.1439682	1.1539311	-1.1593922
32	H	4.6719568	-1.7511909	1.7596	69	C	-7.5596747	2.1045505	-2.1146865
33	H	3.4378394	-3.2510804	3.2667372	70	C	-6.6935965	2.8742384	-2.8882017
34	H	0.9382035	-3.2525075	3.268216	71	C	-5.3229998	2.6528372	-2.6657364
35	H	-0.3006292	-1.7561685	1.7646807	72	C	-4.8764098	1.7299148	-1.7382908
36	H	-0.3006166	1.7561972	-1.7646563	73	C	-8.2296246	0.475902	-0.4778587
37	H	0.938226	3.252532	-3.268188	74	C	-8.2296164	-0.4755451	0.4782631

Tag	Symbol	X	Y	Z
75	H	-9.2159605	0.8010424	-0.8044105
76	H	-9.2159464	-0.8005067	0.8050103
77	H	-4.6719662	-1.7595832	-1.7511679
78	H	-3.437857	-3.2667232	-3.2510617
79	H	-0.9382211	-3.2682011	-3.2525033
80	H	0.3006197	-1.7646634	-1.7561733
81	H	0.3006264	1.7646848	1.7561811
82	H	-0.9382081	3.2682267	3.2525122
83	H	-3.437844	3.2667535	3.2510762
84	H	-4.6719596	1.7596161	1.7511869
85	H	-8.6304755	-2.226616	2.2377227
86	H	-4.5804802	-3.2128525	3.2279055
87	H	-3.8074935	-1.6157216	1.6231102
88	H	-8.6305175	2.2267975	-2.237483
89	H	-4.5805423	3.2125604	-3.2282179
90	H	-3.8075235	1.6155183	-1.6233511
91	C	-7.1645289	-3.9054422	3.9242568
92	C	-6.6222647	-3.5142949	5.3200714
93	H	-6.9381735	-4.2484131	6.0715464
94	H	-5.5281299	-3.4721646	5.3349475
95	H	-6.9985569	-2.5312552	5.6252138
96	C	-6.6225117	-5.3031805	3.539686
97	H	-6.9384492	-6.0511074	4.2774062
98	H	-6.9989581	-5.6129355	2.5581507
99	H	-5.5283832	-5.3184225	3.4975131
100	C	-8.6998541	-3.9919067	4.0113371
101	H	-9.1458219	-3.0363424	4.3100227
102	H	-9.1459901	-4.2950063	3.057242
103	H	-8.985731	-4.7381922	4.76117
104	C	-7.1646046	3.9053085	-3.9243596
105	C	-6.6224589	5.3030424	-3.5399641
106	H	-5.5283272	5.3182086	-3.4978666
107	H	-6.9383967	6.0509234	-4.2777306
108	H	-6.9988128	5.61292	-2.5584304
109	C	-6.6224902	3.5139962	-5.3201889
110	H	-6.9384298	4.2480604	-6.071704
111	H	-5.5283581	3.471813	-5.3351635
112	H	-6.9988609	2.5309472	-5.6251995
113	C	-8.6999316	3.9918629	-4.0113191

Tag	Symbol	X	Y	Z
114	H	-9.1459873	3.0362867	-4.3098358
115	H	-9.1459662	4.2951221	-3.0572273
116	H	-8.9858238	4.7380679	-4.7612263
117	C	7.1645259	3.9243854	3.9053132
118	C	6.6224475	3.5399229	5.3030546
119	H	6.9383668	4.2776884	6.0509443
120	H	5.5283187	3.4977618	5.3182543
121	H	6.9988678	2.5584029	5.6128951
122	C	6.62232	5.3201948	3.5140568
123	H	6.9986413	5.625254	2.531004
124	H	5.5281857	5.3351095	3.4719097
125	H	6.9382414	6.0717073	4.2481316
126	C	8.6998509	4.0114298	3.9918191
127	H	9.1459499	3.0573567	4.2950423
128	H	9.1458581	4.3099935	3.036235
129	H	8.9857253	4.7613363	4.7380318
130	C	7.1646072	-3.9243458	-3.9053228
131	C	6.622435	-5.3201685	-3.5140776
132	H	5.5283025	-5.3351021	-3.4719062
133	H	6.9383584	-6.0716674	-4.2481652
134	H	6.9987801	-5.6252349	-2.5310344
135	C	6.6225198	-3.5398842	-5.303064
136	H	6.938475	-4.2776277	-6.0509607
137	H	5.5283884	-3.497776	-5.318272
138	H	6.9988985	-2.5583443	-5.6128866
139	C	8.6999343	-4.0113439	-3.991837
140	H	9.1460057	-3.0572373	-4.2949954
141	H	9.1459526	-4.3099611	-3.0362749
142	H	8.9858272	-4.7611949	-4.7380983

2F: B3LYP/6-31G(d)

SCF Done: E(RB3LYP) = -2860.85972335 A.U. after 6 cycles

Tag	Symbol	X	Y	Z	Tag	Symbol	X	Y	Z
1	C	0.2203885	2.3050215	-1.5652243	38	C	4.6033813	2.1399116	0.738794
2	H	-0.8450988	2.2908151	-1.3618774	39	C	6.4439247	3.7102377	0.4413341
3	C	1.0125406	1.2235081	-1.153187	40	C	5.2346253	3.3349604	1.0565679
4	C	0.7835374	3.3837451	-2.2410038	41	H	7.9320665	3.0426237	-0.9625607
5	C	2.1519627	3.3916362	-2.5236173	42	H	4.7673037	3.9925378	1.7841909
6	C	2.396378	1.2269264	-1.4482635	43	H	3.6581918	1.8880761	1.2109859
7	C	2.9488381	2.3185664	-2.1340526	44	H	7.7998605	1.1676128	-2.3748542
8	H	0.1530954	4.2090169	-2.5612288	45	H	7.7998801	-1.1675747	-2.3748987
9	H	2.594651	4.2236553	-3.0647398	46	C	7.103445	-5.0554757	0.7942001
10	H	4.0050042	2.3126281	-2.3798711	47	C	7.4275681	-5.0877573	2.3068395
11	C	3.1737963	-0.0000371	-1.1074973	48	C	6.1296837	-6.2092396	0.4550072
12	C	0.4517119	-0.0000277	-0.5105426	49	C	8.4130088	-5.2902687	0.0179973
13	C	2.3963399	-1.2269357	-1.448424	50	H	6.5805366	-7.1763351	0.7095974
14	C	1.0125005	-1.2235069	-1.153344	51	H	5.1882832	-6.1247537	1.0077715
15	C	2.9487404	-2.31847	-2.1344352	52	H	5.8890317	-6.2170631	-0.6142133
16	C	0.2202928	-2.3048896	-1.5656261	53	H	8.2482505	-5.3038955	-1.0654883
17	C	2.1518156	-3.3914275	-2.5242016	54	H	9.1630351	-4.5229114	0.2408835
18	C	0.7833884	-3.3835162	-2.2416008	55	H	8.83952	-6.2597912	0.2991369
19	H	0.1529018	-4.2086837	-2.5620068	56	H	7.8905941	-6.0446724	2.5775731
20	H	2.5944606	-4.2233585	-3.0654949	57	H	8.1234539	-4.2848626	2.5755133
21	H	4.0048982	-2.3125258	-2.3802831	58	H	6.527787	-4.9682426	2.9193647
22	H	-0.8452013	-2.2906499	-1.3623242	59	C	7.1032382	5.055447	0.7943188
23	C	4.4221745	-0.0000572	-0.5680264	60	C	8.4128564	5.2902938	0.0182042
24	C	5.1482014	1.2490156	-0.1978513	61	C	7.4272532	5.0878224	2.3069657
25	C	5.148241	-1.2491244	-0.197891	62	C	6.1294411	6.2091234	0.4549784
26	C	6.3931209	1.5711453	-0.781948	63	H	8.839294	6.259856	0.2993094
27	C	6.3931593	-1.5711856	-0.7820089	64	H	9.1629001	4.5229815	0.2411951
28	C	7.1073255	0.6760897	-1.6925991	65	H	8.2481559	5.3038509	-1.06529
29	C	7.1073386	-0.6760832	-1.6926256	66	H	6.5801873	7.176263	0.7096
30	C	6.9921006	-2.8109003	-0.4726293	67	H	5.8889337	6.2168811	-0.614275
31	C	6.4440269	-3.7103195	0.4412216	68	H	5.1879794	6.1245817	1.0076267
32	C	4.6034741	-2.1400451	0.7387632	69	H	7.8901299	6.0448086	2.5777188
33	C	5.2347354	-3.3350854	1.0564988	70	H	6.5274662	4.9682036	2.9194499
34	H	7.932135	-3.0426244	-0.9626703	71	H	8.123225	4.2850229	2.5757002
35	H	4.767464	-3.9926844	1.7841341	72	C	-0.2203804	2.3050096	1.5652388
36	H	3.6582934	-1.888218	1.2109794	73	H	0.8451068	2.2908017	1.3618912
37	C	6.9920236	2.810864	-0.472536	74	C	-1.012536	1.223502	1.1531928

Tag	Symbol	X	Y	Z
75	C	-0.7835253	3.3837287	2.2410287
76	C	-2.1519501	3.3916206	2.5236447
77	C	-2.3963734	1.226922	1.4482698
78	C	-2.948829	2.3185568	2.1340711
79	H	-0.1530805	4.2089957	2.5612606
80	H	-2.5946351	4.2236357	3.0647761
81	H	-4.0049944	2.3126188	2.3798924
82	C	-3.1737951	-0.000037	1.1074942
83	C	-0.4517107	-0.0000304	0.5105391
84	C	-2.3963421	-1.2269402	1.4484109
85	C	-1.0125028	-1.223513	1.1533311
86	C	-2.9487473	-2.31848	2.1344092
87	C	-0.2202987	-2.3049018	1.5656041
88	C	-2.1518263	-3.3914436	2.5241663
89	C	-0.7833985	-3.3835331	2.2415682
90	H	-0.1529148	-4.2087057	2.5619667
91	H	-2.5944746	-4.223379	3.0654502
92	H	-4.0049059	-2.3125354	2.3802542
93	H	0.8451955	-2.2906638	1.362303
94	C	-4.4221739	-0.0000538	0.5680246
95	C	-5.1482014	1.249019	0.1978503
96	C	-5.1482409	-1.2491211	0.1978911
97	C	-6.3931199	1.5711488	0.781949
98	C	-6.3931583	-1.5711822	0.7820112
99	C	-7.1073229	0.6760935	1.6926016
100	C	-7.1073361	-0.6760793	1.6926286
101	C	-6.9920994	-2.8108975	0.472634
102	C	-6.444026	-3.710318	-0.4412159
103	C	-4.6034732	-2.1400442	-0.7387603
104	C	-5.2347343	-3.3350852	-1.0564935
105	H	-7.932133	-3.0426214	0.9626765
106	H	-4.7674626	-3.9926858	-1.7841271
107	H	-3.6582923	-1.8882181	-1.2109766
108	C	-6.9920238	2.8108669	0.4725369
109	C	-4.603385	2.139913	-0.7387991
110	C	-6.4439276	3.7102394	-0.441336
111	C	-5.2346303	3.3349611	-1.0565732
112	H	-7.9320657	3.0426268	0.9625633
113	H	-4.7673111	3.9925371	-1.7841991

Tag	Symbol	X	Y	Z
114	H	-3.6581972	1.8880767	-1.210994
115	H	-7.7998569	1.167617	2.3748576
116	H	-7.7998764	-1.1675705	2.3749032
117	C	-7.1034435	-5.0554755	-0.794191
118	C	-7.4275661	-5.0877613	-2.3068304
119	C	-6.1296815	-6.2092379	-0.4549947
120	C	-8.4130073	-5.2902671	-0.017988
121	H	-6.5805342	-7.1763344	-0.7095818
122	H	-5.1882813	-6.1247535	-1.0077595
123	H	-5.8890293	-6.2170581	0.6142258
124	H	-8.2482496	-5.3038883	1.0654978
125	H	-9.1630349	-4.5229122	-0.2408783
126	H	-8.8395166	-6.2597917	-0.2991231
127	H	-7.8905911	-6.0446776	-2.5775616
128	H	-8.1234526	-4.284868	-2.5755065
129	H	-6.527785	-4.9682473	-2.9193557
130	C	-7.1032426	5.0554479	-0.7943212
131	C	-8.4128575	5.2902966	-0.0182017
132	C	-7.4272641	5.0878195	-2.3069669
133	C	-6.129444	6.2091251	-0.4549879
134	H	-8.8392973	6.2598573	-0.2993088
135	H	-9.1629016	4.5229827	-0.241186
136	H	-8.2481521	5.3038583	1.0652918
137	H	-6.5801912	7.176264	-0.7096105
138	H	-5.8889325	6.2168858	0.6142645
139	H	-5.1879845	6.1245817	-1.0076397
140	H	-7.8901423	6.0448048	-2.5777203
141	H	-6.5274797	4.9681994	-2.9194546
142	H	-8.1232368	4.2850191	-2.5756964

3T (triplet): UB3LYP/6-31G(d)

SCF Done: E(UB3LYP) = -3399.17401350 A.U. after 6 cycles

Tag	Symbol	X	Y	Z	Tag	Symbol	X	Y	Z
1	C	-5.801354	-0.0000002	-0.0000086	38	H	-5.6199325	0.0006581	-4.6089345
2	C	-5.0901451	-0.0001695	1.2212775	39	H	-6.8543569	0.0003624	-2.4827287
3	C	-5.7699249	-0.0003543	2.4823196	40	H	-10.8127749	3.1570736	0.0002108
4	C	-5.0809297	-0.0005193	3.6654614	41	H	-6.7627846	4.5542561	-0.0000352
5	C	-3.6582359	-0.0005118	3.6664925	42	H	-5.9898735	2.2899406	-0.0001022
6	C	-2.9661655	-0.0003431	2.4851374	43	H	-10.8127804	-3.1570674	-0.0004788
7	C	-3.6433929	-0.0001683	1.2223835	44	H	-6.7627926	-4.554257	-0.0005319
8	C	-2.9343832	-0.0000003	0.0000659	45	H	-5.9898774	-2.2899431	-0.0001783
9	C	-3.6433296	0.0001679	-1.2222894	46	C	1.4321559	-0.0000006	0.0001492
10	C	-2.966037	0.0003427	-2.4850081	47	C	0.7234085	1.2223289	0.000296
11	C	-3.6580465	0.0005118	-3.6663992	48	C	1.4017064	2.484358	0.0004665
12	C	-5.08074	0.0005196	-3.6654414	49	C	0.7113022	3.6665165	0.0006092
13	C	-5.7697961	0.0003546	-2.4823348	50	C	-0.7113013	3.6665166	0.0005894
14	C	-5.0900813	0.0001694	-1.2212585	51	C	-1.4017057	2.4843582	0.0004261
15	C	-7.3129455	0.0000003	-0.0000442	52	C	-0.723408	1.222329	0.0002736
16	C	-7.9247715	1.3191737	0.0000102	53	C	-1.4321556	-0.0000004	0.0001027
17	C	-9.3264102	1.6358334	0.000094	54	C	-0.7234082	-1.2223299	-0.0000399
18	C	-9.7419749	2.9835808	0.000137	55	C	-1.401706	-2.4843591	-0.0002123
19	C	-8.8757815	4.0746799	0.0000861	56	C	-0.7113018	-3.6665175	-0.0003476
20	C	-7.5052065	3.7607052	0.0000017	57	C	0.7113018	-3.6665176	-0.0003214
21	C	-7.0587411	2.4522881	-0.0000329	58	C	1.4017061	-2.4843592	-0.0001617
22	C	-7.9247736	-1.3191724	-0.0001717	59	C	0.7234084	-1.22233	-0.0000152
23	C	-9.3264129	-1.6358295	-0.0002228	60	H	2.4860997	2.4873269	0.0004822
24	C	-9.74198	-2.9835763	-0.0004427	61	H	1.2497361	4.6104504	0.0007384
25	C	-8.8757887	-4.0746768	-0.0005976	62	H	-1.2497352	4.6104506	0.0007041
26	C	-7.505213	-3.7607047	-0.0004716	63	H	-2.486099	2.4873273	0.0004112
27	C	-7.0587452	-2.4522886	-0.0002662	64	H	-2.4860993	-2.4873281	-0.0002321
28	C	-10.4120437	-0.6744386	-0.0000025	65	H	-1.2497357	-4.6104515	-0.0004753
29	C	-10.4120427	0.6744442	0.0001485	66	H	1.2497356	-4.6104516	-0.0004287
30	H	-11.3983798	-1.1352335	0.0000572	67	H	2.4860994	-2.4873282	-0.0001421
31	H	-11.3983779	1.1352407	0.0003118	68	C	5.8013544	-0.0000005	0.0001803
32	H	-6.8544857	-0.000362	2.4826577	69	C	5.0901215	0.0001447	-1.2210928
33	H	-5.6201703	-0.0006575	4.608927	70	C	5.7698771	0.0002984	-2.4821471
34	H	-3.1205342	-0.0006429	4.6108977	71	C	5.0808595	0.0004425	-3.6652761
35	H	-1.8816863	-0.0003409	2.49005	72	C	3.658166	0.0004445	-3.6662801
36	H	-1.8815576	0.0003403	-2.4898652	73	C	2.9661182	0.0003019	-2.4849115
37	H	-3.1202958	0.0006429	-4.6107765	74	C	3.6433697	0.0001462	-1.2221709

Tag	Symbol	X	Y	Z
75	C	2.9343835	-0.0000007	0.0001613
76	C	3.6433534	-0.0001475	1.2225021
77	C	2.9660848	-0.0003032	2.4852339
78	C	3.6581168	-0.0004458	3.6666116
79	C	5.0808106	-0.0004437	3.6656267
80	C	5.7698443	-0.0002996	2.4825073
81	C	5.0901056	-0.0001459	1.2214432
82	C	7.3129458	-0.0000005	0.0001927
83	C	7.9247733	1.3191725	0.0001336
84	C	9.3264124	1.6358304	0.0001417
85	C	9.7419788	2.9835774	-0.0000095
86	C	8.8757869	4.0746775	-0.0001635
87	C	7.5052114	3.7607046	-0.0001256
88	C	7.0587443	2.4522882	0.0000161
89	C	7.9247725	-1.3191736	0.0002217
90	C	9.3264115	-1.6358325	0.0003108
91	C	9.7419769	-2.9835797	0.0003226
92	C	8.8757841	-4.0746794	0.0002506
93	C	7.5052089	-3.7607054	0.000188
94	C	7.0587427	-2.4522886	0.0001775
95	C	10.4120433	-0.6744428	0.0004179
96	C	10.4120437	0.67444	0.0003359
97	H	11.3983789	-1.1352388	0.0005732
98	H	11.3983795	1.1352354	0.0004311
99	H	6.8544379	0.0002989	-2.482506
100	H	5.6200826	0.0005568	-4.6087516
101	H	3.1204459	0.0005613	-4.6106749
102	H	1.8816389	0.0003057	-2.4898038
103	H	1.8816055	-0.000307	2.4901112
104	H	3.1203843	-0.0005627	4.6109993
105	H	5.6200205	-0.0005581	4.6091099
106	H	6.8544051	-0.0003	2.4828806
107	H	10.8127792	3.1570689	0.0000036
108	H	6.7627906	4.5542565	-0.0002078
109	H	5.9898765	2.2899422	0.0000308
110	H	10.812777	-3.1570717	0.0003983
111	H	6.7627876	-4.5542569	0.0001493
112	H	5.9898751	-2.2899418	0.0001313
113	C	9.3465056	5.5365234	-0.0003711

Tag	Symbol	X	Y	Z
114	C	8.8040664	6.249962	1.2614046
115	H	9.119742	7.3005247	1.2710629
116	H	7.7099324	6.2305355	1.3017642
117	H	9.1804778	5.7729458	2.1734617
118	C	8.8043344	6.2494862	-1.2625372
119	H	9.1200535	7.3000325	-1.2725577
120	H	9.180892	5.7720905	-2.1743338
121	H	7.7102065	6.2300896	-1.3030849
122	C	10.8818375	5.659573	-0.0002259
123	H	11.3279529	5.1975258	0.8878666
124	H	11.3281584	5.1971404	-0.888016
125	H	11.167407	6.7175784	-0.0004219
126	C	9.3464999	-5.5365263	0.0002407
127	C	8.8040107	-6.2498036	1.2620865
128	H	7.7098752	-6.2303705	1.3024009
129	H	9.1196848	-7.3003654	1.2718911
130	H	9.1803873	-5.7726715	2.1740971
131	C	8.8043719	-6.2496461	-1.261855
132	H	9.1200857	-7.3001954	-1.2717307
133	H	7.7102454	-6.2302491	-1.3024461
134	H	9.1809661	-5.7723682	-2.173698
135	C	10.8818316	-5.6595821	0.0004585
136	H	11.3281868	-5.197279	-0.8873816
137	H	11.3279167	-5.1974094	0.8885009
138	H	11.1673967	-6.7175887	0.000424
139	C	-9.346509	-5.5365223	-0.0008775
140	C	-8.8041195	-6.2500074	1.2608934
141	H	-9.1197949	-7.3005707	1.2705001
142	H	-7.7099871	-6.2305815	1.3012966
143	H	-9.1805672	-5.7730253	2.1729532
144	C	-8.8042913	-6.2494404	-1.2630486
145	H	-9.1808116	-5.77201	-2.1748424
146	H	-7.7101618	-6.2300471	-1.3035534
147	H	-9.1200142	-7.2999851	-1.27312
148	C	-10.881841	-5.6595687	-0.000796
149	H	-11.3279894	-5.197563	0.8873014
150	H	-11.328127	-5.1970927	-0.8885811
151	H	-11.1674126	-6.7175736	-0.0010528
152	C	-9.3464973	5.5365269	0.000121

Tag	Symbol	X	Y	Z
153	C	-8.8039878	6.2497643	1.2619819
154	H	-7.7098517	6.2303251	1.302277
155	H	-9.1196589	7.3003267	1.2718244
156	H	-9.1803516	5.7726036	2.1739827
157	C	-8.8043914	6.2496878	-1.2619597
158	H	-7.7102656	6.2302985	-1.3025704
159	H	-9.180998	5.772438	-2.1738126
160	H	-9.1201097	7.300236	-1.2717969
161	C	-10.881829	5.6595813	0.0003649
162	H	-11.328197	5.197282	-0.8874709
163	H	-11.3279003	5.1974038	0.8884117
164	H	-11.167395	6.7175876	0.0003417

3F: B3LYP/6-31G(d)

SCF Done: E(RB3LYP) =   -3399.16774272   A.U. after   6 cycles

Tag	Symbol	X	Y	Z	Tag	Symbol	X	Y	Z
1	C	-2.514565	2.3028593	2.2240759	38	C	-6.2613939	2.1395421	-1.0204249
2	H	-1.4310173	2.2841284	2.27372	39	C	-8.1222762	3.7088041	-1.1490937
3	C	-3.19305	1.2231066	1.640975	40	C	-6.8048377	3.3339451	-1.4739903
4	C	-3.215954	3.382159	2.7539169	41	H	-9.8891983	3.0416001	-0.1175996
5	C	-4.6126826	3.3916436	2.7153224	42	H	-6.185343	3.9913851	-2.0775146
6	C	-4.607734	1.2271263	1.6120945	43	H	-5.2338209	1.8877253	-1.2663994
7	C	-5.300881	2.3195539	2.1535106	44	H	-10.0788385	1.1677351	1.2905379
8	H	-2.6746626	4.205875	3.2115995	45	H	-10.0788629	-1.1676042	1.2905904
9	H	-5.166438	4.2239317	3.1415369	46	C	-8.6859227	-5.0527759	-1.6441491
10	H	-6.3852564	2.3149286	2.151802	47	C	-8.6586763	-5.082612	-3.1909516
11	C	-5.2873417	-0.0000072	1.1034201	48	C	-7.8155304	-6.2082524	-1.0949088
12	C	-2.5017194	0.0000015	1.1417685	49	C	-10.1375743	-5.2867462	-1.1854133
13	C	-4.6077333	-1.2271131	1.6121596	50	H	-8.1977387	-7.1743798	-1.4468518
14	C	-3.1930478	-1.2230841	1.6410338	51	H	-6.773215	-6.1241364	-1.4196413
15	C	-5.300871	-2.3195031	2.1536675	52	H	-7.8236896	-6.2180562	0.0010077
16	C	-2.5145581	-2.3027832	2.224232	53	H	-10.2227314	-5.3020716	-0.092821
17	C	-4.6126662	-3.3915458	2.7155584	54	H	-10.8167385	-4.5180857	-1.5713377
18	C	-3.2159378	-3.3820478	2.7541539	55	H	-10.4902741	-6.2553168	-1.5574312
19	H	-2.6746413	-4.2057208	3.2119075	56	H	-9.0496654	-6.0383576	-3.5611293
20	H	-5.166416	-4.2238008	3.1418448	57	H	-9.2743159	-4.2781397	-3.6091559
21	H	-6.385246	-2.3148798	2.1519749	58	H	-7.6432659	-4.9639631	-3.5833301
22	H	-1.4310121	-2.2840349	2.2738902	59	C	-8.6857144	5.0527878	-1.6443117
23	C	-6.3805325	-0.0000112	0.2944569	60	C	-10.1373927	5.2868127	-1.1856565
24	C	-7.0038768	1.2489717	-0.2308208	61	C	-8.6583754	5.0826447	-3.1911022
25	C	-7.0039211	-1.2489903	-0.2307887	62	C	-7.8153115	6.2082052	-1.0949934
26	C	-8.3487408	1.5709197	0.0561931	63	H	-10.4900353	6.255417	-1.5576337
27	C	-8.3487871	-1.5708761	0.0562582	64	H	-10.8165528	4.51819	-1.5716659
28	C	-9.2502007	0.6761203	0.782292	65	H	-10.2225928	5.3020775	-0.0930675
29	C	-9.2502169	-0.6760244	0.7823217	66	H	-8.1974154	7.1743576	-1.4469864
30	C	-8.8626195	-2.8099739	-0.381759	67	H	-7.8235876	6.2180117	0.0009225
31	C	-8.1223935	-3.7088188	-1.148957	68	H	-6.7729689	6.1240148	-1.4196183
32	C	-6.261483	-2.1395979	-1.0203919	69	H	-9.049225	6.038443	-3.5612989
33	C	-6.8049542	-3.3340007	-1.473907	70	H	-7.6429634	4.9638827	-3.5834291
34	H	-9.8892834	-3.0415262	-0.1174686	71	H	-9.2740817	4.2782476	-3.6093525
35	H	-6.1854997	-3.9914659	-2.0774424	72	C	-1.3932815	2.2989863	-0.7451616
36	H	-5.2339129	-1.8877951	-1.2663974	73	H	-2.4777628	2.2886982	-0.7661429
37	C	-8.8625327	2.8100123	-0.3818704	74	C	-0.7075931	1.221931	-0.1637179

Tag	Symbol	X	Y	Z
75	C	-0.6988497	3.365721	-1.307907
76	C	0.69887	3.3657133	-1.3079148
77	C	0.7076041	1.2219269	-0.1637176
78	C	1.3932968	2.2989734	-0.7451729
79	H	-1.2465362	4.1865198	-1.7632028
80	H	1.2465601	4.1865062	-1.7632167
81	H	2.4777775	2.2886748	-0.7661643
82	C	1.3937499	-0.0000051	0.3477326
83	C	-1.3937466	0.000004	0.3477305
84	C	0.7075972	-1.2219006	-0.1637958
85	C	-0.7076006	-1.221896	-0.1637987
86	C	1.3932855	-2.2989034	-0.745339
87	C	-1.393291	-2.2988881	-0.7453588
88	C	0.6988553	-3.36559	-1.3081765
89	C	-0.6988643	-3.365581	-1.3081887
90	H	-1.2465536	-4.1863309	-1.763569
91	H	1.2465423	-4.1863465	-1.7635478
92	H	2.4777661	-2.2886075	-0.7663298
93	H	-2.4777709	-2.2885806	-0.7663662
94	C	6.3805255	-0.0000264	0.2944582
95	C	7.0038987	1.2489611	-0.2307825
96	C	7.0038939	-1.2490017	-0.2308175
97	C	8.3487644	1.5708693	0.0562531
98	C	8.3487576	-1.5709231	0.0562127
99	C	9.2502053	0.6760339	0.7823249
100	C	9.2502018	-0.6761102	0.7823083
101	C	8.862563	-2.8100154	-0.381843
102	C	8.1223133	-3.7088259	-1.1490619
103	C	6.2614322	-2.1395753	-1.020434
104	C	6.8048787	-3.3339772	-1.4739866
105	H	9.8892239	-3.0415965	-0.1175665
106	H	6.1854061	-3.9914167	-2.0775314
107	H	5.2338637	-1.8877498	-1.2664233
108	C	8.8625744	2.8099705	-0.3817723
109	C	6.2614394	2.1395584	-1.020375
110	C	8.1223273	3.7088037	-1.1489667
111	C	6.8048903	3.3339693	-1.4738982
112	H	9.8892371	3.0415399	-0.1174921
113	H	6.1854196	3.9914269	-2.0774252
114	H	5.2338696	1.8877431	-1.2663691
115	H	10.0788454	1.1676231	1.2905934
116	H	10.0788392	-1.1677161	1.290565
117	C	8.685815	-5.0527793	-1.6442976
118	C	8.6585592	-5.0825682	-3.1911009
119	C	10.1374651	-5.2867902	-1.1855772
120	C	7.8154046	-6.2082562	-1.0950873
121	H	10.4901442	-6.2553569	-1.5576249
122	H	10.8166414	-4.5181317	-1.5714843
123	H	10.2226289	-5.3021483	-0.092986
124	H	7.8235693	-6.2180933	0.0008288
125	H	6.7730889	-6.1241114	-1.4198117
126	H	8.1975931	-7.1743799	-1.4470619
127	H	9.0495277	-6.0383102	-3.5613095
128	H	7.6431488	-4.9638878	-3.5834697
129	H	9.2742119	-4.2780952	-3.6092846
130	C	8.685835	5.0527656	-1.6441721
131	C	10.137487	5.2867584	-1.1854484
132	C	7.815432	6.208235	-1.0949348
133	C	8.6585773	5.082589	-3.1909748
134	H	10.4901707	6.2553317	-1.5574746
135	H	10.2226521	5.3020912	-0.0928569
136	H	10.8166589	4.5181053	-1.5713738
137	H	9.0495513	6.0383368	-3.5611629
138	H	9.2742244	4.2781214	-3.609177
139	H	7.6431657	4.9639236	-3.5833448
140	H	8.1976252	7.1743644	-1.4468886
141	H	6.7731152	6.1241034	-1.4196591
142	H	7.8235988	6.2180479	0.0009816
143	C	5.3008797	2.3194703	2.1536489
144	H	6.3852548	2.3148419	2.1519552
145	C	4.6077362	1.2270754	1.612161
146	C	4.6126791	3.3915294	2.7155159
147	C	3.2159505	3.3820444	2.7541012
148	C	3.1930511	1.2230551	1.6410347
149	C	2.5145644	2.3027741	2.2241972
150	H	5.1664329	4.223791	3.1417843
151	H	2.6746569	4.2057333	3.21183
152	H	1.4310171	2.2840382	2.2738415

Tag	Symbol	X	Y	Z
153	C	2.5017203	-0.0000286	1.1417738
154	C	5.2873395	-0.0000371	1.1034288
155	C	3.1930436	-1.2231354	1.6409903
156	C	4.6077289	-1.2271636	1.612116
157	C	2.5145516	-2.3028669	2.2241238
158	C	5.3008659	-2.3195828	2.1535632
159	C	3.2159313	-3.3821602	2.7539892
160	C	4.6126596	-3.3916558	2.7153959
161	H	5.1664088	-4.2239361	3.1416338
162	H	2.6746334	-4.2058591	3.2116948
163	H	1.431005	-2.2841231	2.2737764
164	H	6.3852411	-2.3149622	2.1518669

4T (triplet): UB3LYP/6-31G(d)

SCF Done: E(UB3LYP) = -3937.50229271 A.U. after 5 cycles									
**Tag**	**Symbol**	**X**	**Y**	**Z**	**Tag**	**Symbol**	**X**	**Y**	**Z**
1	C	-7.9836517	0.0000366	0.0000223	38	H	-7.8024524	3.259228	3.2587986
2	C	-7.2723928	-0.8635868	-0.8634857	39	H	-9.0367704	1.7556553	1.7554167
3	C	-7.952203	-1.7553218	-1.7551034	40	H	-12.9952034	-2.231872	2.2324535
4	C	-7.263211	-2.5919862	-2.5916541	41	H	-8.9452895	-3.2205437	3.2203335
5	C	-5.8405245	-2.5927127	-2.5923747	42	H	-8.1722285	-1.6196218	1.6193853
6	C	-5.1484118	-1.757328	-1.7570989	43	H	-12.9952327	2.232166	-2.232142
7	C	-5.8256541	-0.8643678	-0.8642621	44	H	-8.9453336	3.220083	-3.2208349
8	C	-5.116738	0.0000361	0.0000316	45	H	-8.1722494	1.6193187	-1.6197426
9	C	-5.8256597	0.8644378	0.8643215	46	C	-0.751029	0.0000217	0.0000326
10	C	-5.1484216	1.7573959	1.7571642	47	C	-1.4591282	-0.8643895	0.8645556
11	C	-5.8405381	2.5927785	2.5924381	48	C	-0.780448	-1.7566115	1.7568819
12	C	-7.263225	2.5920521	2.5917101	49	C	-1.4709246	-2.5924581	2.5928337
13	C	-7.9522132	1.7553902	1.7551547	50	C	-2.8935367	-2.5924644	2.5928474
14	C	-7.2723993	0.8636566	0.8635372	51	C	-3.584116	-1.7566576	1.7569427
15	C	-9.4952177	0.000043	0.0000293	52	C	-2.9059149	-0.8643098	0.864484
16	C	-10.1070127	-0.9327386	0.9328718	53	C	-3.6145854	0.0000346	0.0000352
17	C	-11.5086746	-1.156486	1.1568593	54	C	-2.9059088	0.8643744	-0.8644132
18	C	-11.9243686	-2.1094429	2.1098307	55	C	-3.5841032	1.7567297	-1.7568693
19	C	-11.058311	-2.8811584	2.8813341	56	C	-2.8935177	2.5925308	-2.5927747
20	C	-9.6877102	-2.6593352	2.6592902	57	C	-1.4709058	2.5925102	-2.592765
21	C	-9.2411306	-1.7341571	1.7340986	58	C	-0.7804353	1.7566561	-1.7568155
22	C	-10.1070231	0.9327936	-0.932839	59	C	-1.4591221	0.8644406	-0.8644878
23	C	-11.5086885	1.156727	-1.1566222	60	H	0.3039726	-1.7590562	1.7593208
24	C	-11.9243961	2.1095854	-2.1096859	61	H	-0.9324279	-3.2598371	3.2602905
25	C	-11.05835	2.881036	-2.8814668	62	H	-3.4318518	-3.2599222	3.2603889
26	C	-9.6877461	2.6590655	-2.65959	63	H	-4.6684921	-1.7588595	1.7591508
27	C	-9.241153	1.7339787	-1.7343134	64	H	-4.6684792	1.7589438	-1.7590733
28	C	-12.5943335	0.4771604	-0.4766256	65	H	-3.431828	3.2599956	-3.2603132
29	C	-12.594328	-0.4765686	0.477227	66	H	-0.932404	3.2598844	-3.2602225
30	H	-13.5806631	0.8031293	-0.8023396	67	H	0.3039854	1.7590902	-1.759257
31	H	-13.5806536	-0.8022459	0.8032444	68	C	0.751029	0.0000124	0.0000283
32	H	-9.0367602	-1.7555855	-1.7553699	69	C	1.4591168	-0.8645113	-0.8643916
33	H	-7.8024353	-3.2591633	-3.2587437	70	C	0.7804244	-1.7568317	-1.7566102
34	H	-5.3028518	-3.2605667	-3.260136	71	C	1.4708896	-2.5927838	-2.592466
35	H	-4.0639203	-1.7609373	-1.7607011	72	C	2.8935015	-2.5928037	-2.5924854
36	H	-4.0639301	1.7610043	1.7607719	73	C	3.5840922	-1.7569057	-1.7566813
37	H	-5.302869	3.2606308	3.2602038	74	C	2.9059034	-0.8644475	-0.8643238

Tag	Symbol	X	Y	Z	Tag	Symbol	X	Y	Z
75	C	3.6145854	-0.0000065	0.0000192	114	C	9.6876832	2.6595371	2.6590675
76	C	2.9059203	0.8644443	0.8643658	115	C	9.241114	1.7342503	1.7339797
77	C	3.584127	1.7568947	1.7567177	116	C	12.5943241	0.4769158	0.4768886
78	C	2.8935529	2.5928029	2.5925252	117	C	12.5943376	-0.4769628	-0.4768143
79	C	1.4709408	2.5928017	2.5925154	118	H	13.5806469	0.8027835	0.8027241
80	C	0.7804589	1.7568578	1.7566652	119	H	13.58067	-0.802845	-0.8026066
81	C	1.4591336	0.8645273	0.8644432	120	H	9.0367591	-1.755457	1.7556014
82	H	0.9323836	-3.2602358	-3.2598424	121	H	7.8024324	-3.2588425	3.2591655
83	H	3.4318076	-3.2603447	-3.2599511	122	H	5.302849	-3.2602209	3.2605801
84	H	4.6684682	-1.7591183	-1.7588937	123	H	4.0639188	-1.7607625	1.7609731
85	H	4.6685031	1.7590931	1.7589223	124	H	4.0639316	1.7607515	-1.7609274
86	H	3.4318722	3.2603378	3.2599863	125	H	5.3028718	3.2602053	-3.2605311
87	H	0.9324483	3.2602617	3.2598946	126	H	7.8024553	3.2587974	-3.2591283
88	H	-0.3039616	1.7593061	1.7591073	127	H	9.0367715	1.7553883	-1.7555808
89	C	7.9836517	-0.0000366	0.0000056	128	H	12.9952545	-2.2323398	-2.2319527
90	C	7.2723927	-0.8635506	0.8636217	129	H	8.9453666	-3.2207513	-3.2201956
91	C	7.9522019	-1.7551839	1.7553427	130	H	8.1722649	-1.619713	-1.6193878
92	C	7.2632089	-2.5917412	2.5919992	131	H	12.9951817	2.2323856	2.2319554
93	C	5.8405222	-2.5924542	2.5927319	132	H	8.9452565	3.22071	3.2201381
94	C	5.1484103	-1.7571655	1.7573599	133	H	8.172213	1.6195983	1.6193692
95	C	5.8256535	-0.8643217	0.864407	134	H	-0.3039963	-1.7592656	-1.7590452
96	C	5.116738	-0.0000164	0.0000148	135	C	-11.5293308	-3.9147648	3.9149513
97	C	5.8256604	0.8642835	-0.8643772	136	C	-10.9870497	-3.5269796	5.3116994
98	C	5.1484231	1.7571413	-1.7573203	137	H	-11.3030287	-4.2628782	6.0613888
99	C	5.8405405	2.5924277	-2.5926902	138	H	-9.8929072	-3.4849863	5.3267231
100	C	7.263227	2.5916981	-2.5919639	139	H	-11.363281	-2.5446579	5.6192092
101	C	7.9522144	1.7551274	-1.755316	140	C	-10.9873461	-5.3115781	3.5269861
102	C	7.2723994	0.8634953	-0.8635988	141	H	-11.3637765	-5.6189554	2.5447
103	C	9.4952177	-0.0000392	0.0000073	142	H	-9.893212	-5.3267868	3.4848312
104	C	10.1070307	-0.932924	-0.9327235	143	H	-11.3033416	-6.0612643	4.262881
105	C	11.5086986	-1.156792	-1.1565582	144	C	-13.0646582	-4.0013754	4.001808
106	C	11.9244165	-2.1098193	-2.1094485	145	H	-13.5106011	-3.046525	4.3028034
107	C	11.0583791	-2.881497	-2.881012	146	H	-13.5108102	-4.3021886	3.0469976
108	C	9.6877729	-2.6595705	-2.6591058	147	H	-13.3505541	-4.7494448	4.7498429
109	C	9.2411697	-1.7343243	-1.7339935	148	C	-11.5293849	3.9145275	-3.9151919
110	C	10.1070052	0.9328588	0.9327367	149	C	-10.987186	5.3113416	-3.527529
111	C	11.5086645	1.1567625	1.1565817	150	H	-9.8930457	5.3264257	-3.4855039
112	C	11.9243482	2.1098338	2.1094433	151	H	-11.3031804	6.0609414	-4.2635122
113	C	11.0582818	2.8815148	2.8809716	152	H	-11.3634635	5.6189302	-2.5452497

Tag	Symbol	X	Y	Z
153	C	-10.9873331	3.5264477	-5.3119473
154	H	-9.8931965	3.4843411	-5.3271121
155	H	-11.3637072	2.5441134	-5.6192395
156	H	-11.3033358	4.2622528	-6.0617186
157	C	-13.0647136	4.0013003	-4.0018631
158	H	-13.5108079	3.0464416	-4.3026083
159	H	-13.5107044	4.3023568	-3.0470542
160	H	-13.3506209	4.7492555	-4.7500078
161	C	11.5292905	3.915249	3.9144659
162	C	11.5294252	-3.9151735	-3.9145471
163	C	10.9873101	-5.3119358	-3.5265822
164	H	9.8931722	-5.3270644	-3.4845093
165	H	11.3033108	-6.0616679	-4.2624279
166	H	11.3636424	-5.6193094	-2.5442565
167	C	10.9873002	-3.5274018	-5.3113601
168	H	9.8931609	-3.4853548	-5.3264919
169	H	11.3636121	-2.5451099	-5.6188636
170	H	11.3033123	-4.2633413	-6.0609955
171	C	13.0647548	-4.0018883	-4.0012607
172	H	13.5107909	-3.0470672	-4.3022117
173	H	13.5107971	-4.3027345	-3.0464096
174	H	13.3506698	-4.749977	-4.7492689
175	C	10.9871701	3.5275254	5.3112932
176	H	11.3031466	4.2635149	6.0608943
177	H	9.8930331	3.4854307	5.3264191
178	H	11.363522	2.5452644	5.6188491
179	C	10.9871343	5.3119774	3.5264329
180	H	11.3634516	5.6193121	2.5440901
181	H	9.8929951	5.3270724	3.4843676
182	H	11.3031164	6.0617554	4.2622401
183	C	13.064617	4.0020165	4.0011815
184	H	13.5106801	3.0472287	4.3021977
185	H	13.5106554	4.3028134	3.046313
186	H	13.3505051	4.7501617	4.7491434

4F: B3LYP/6-31G(d)

SCF Done: E(RB3LYP) = -3937.47578545 A.U. after 15 cycles									
Tag	Symbol	X	Y	Z	Tag	Symbol	X	Y	Z
1	C	3.4956285	2.3010492	1.0671162	38	C	4.2521009	-2.302588	-2.0135666
2	H	4.574329	2.2923418	0.9536616	39	C	6.27753	-3.3917679	-2.7484962
3	C	2.7448373	1.2222765	0.5764142	40	C	4.8859716	-3.3820806	-2.622234
4	C	2.8747767	3.3676379	1.7105571	41	H	4.2944543	-4.2058455	-3.0127329
5	C	1.4880362	3.3657996	1.8851525	42	H	6.7770738	-4.2242788	-3.2367333
6	C	1.3404969	1.2215688	0.7518731	43	H	8.1043845	-2.3152277	-2.3979577
7	C	0.7307895	2.2975039	1.4138633	44	H	3.170272	-2.2837096	-1.9348495
8	H	3.4737187	4.1895151	2.0935886	45	C	8.3184158	-0.0000032	-0.5526514
9	H	1.0002538	4.1855966	2.4056027	46	C	8.9985838	1.2489233	-0.1032804
10	H	-0.3420333	2.2825715	1.573354	47	C	8.9985864	-1.2489227	-0.1032656
11	C	0.5972456	0.0000059	0.3282237	48	C	10.301261	1.5708826	-0.5437979
12	C	3.3635878	0.0000033	-0.0144885	49	C	10.3012647	-1.5708838	-0.5437791
13	C	1.340501	-1.2215381	0.7519237	50	C	11.1127565	0.6760775	-1.3692441
14	C	2.7448417	-1.2222484	0.5764642	51	C	11.1127581	-0.6760868	-1.3692357
15	C	0.7308006	-2.2974409	1.4139735	52	C	10.8623515	-2.8098791	-0.1676745
16	C	3.4956381	-2.3009938	1.0672193	53	C	10.2157809	-3.7084487	0.6802425
17	C	1.4880521	-3.3657107	1.8853133	54	C	8.3525053	-2.1391992	0.7672776
18	C	2.8747919	-3.3675534	1.7107134	55	C	8.944744	-3.3334557	1.1552106
19	H	3.4737382	-4.1894083	2.0937863	56	H	11.85165	-3.0414957	-0.5486924
20	H	1.0002743	-4.1854822	2.405808	57	H	8.3992851	-3.9906264	1.8266385
21	H	-0.3420208	-2.2825027	1.5734709	58	H	7.3604992	-1.887118	1.1308278
22	H	4.5743387	-2.292286	0.9537664	59	C	10.8623449	2.8098836	-0.1677077
23	C	4.2520942	2.3025399	-2.0136217	60	C	8.3525031	2.1392061	0.7672568
24	H	3.1702657	2.2836627	-1.9348982	61	C	10.2157732	3.7084606	0.6802008
25	C	4.9947897	1.2229583	-1.5146239	62	C	8.9447391	3.3334683	1.1551764
26	C	4.8859608	3.3820179	-2.6223193	63	H	11.8516423	3.0414986	-0.5487295
27	C	6.2775185	3.3917025	-2.7485902	64	H	8.3992798	3.990644	1.8265992
28	C	6.4029941	1.2270496	-1.6528591	65	H	7.3604989	1.8871256	1.1308128
29	C	7.0273347	2.3196523	-2.2720217	66	H	11.8771808	1.1676958	-1.9697505
30	H	4.2944407	4.2057723	-3.0128361	67	H	11.8771833	-1.1677107	-1.9697363
31	H	6.7770587	4.2242007	-3.2368526	68	C	10.8322729	-5.0524063	1.1075146
32	H	8.1043758	2.315174	-2.3980318	69	C	9.9042761	-6.2076734	0.6619157
33	C	7.1378155	-0.0000109	-1.227697	70	C	12.2215376	-5.2867551	0.4849353
34	C	4.3677975	-0.0000106	-0.9363363	71	C	10.9831462	-5.0822268	2.6471709
35	C	6.4029973	-1.2270847	-1.6528253	72	H	12.6144892	-6.2552754	0.8142642
36	C	4.994793	-1.2229937	-1.5145911	73	H	12.1805058	-5.3024508	-0.6102048
37	C	7.0273427	-2.319704	-2.2719532	74	H	12.9406731	-4.5180957	0.7899836

Tag	Symbol	X	Y	Z	Tag	Symbol	X	Y	Z
75	H	11.6432212	-4.278049	2.9916609	114	H	-1.0002628	-4.1855326	-2.4057248
76	H	10.0197689	-4.9631319	3.153925	115	H	-3.4737354	4.1894546	-2.0936865
77	H	11.4137198	-6.0381655	2.9698803	116	C	-4.9947938	1.2229663	1.5146112
78	H	10.3240634	-7.1738693	0.9678345	117	C	-4.9947909	-1.2229846	1.514613
79	H	8.9061028	-6.1232514	1.1040335	118	H	-3.4737268	-4.1894604	-2.0937037
80	H	9.786663	-6.2175733	-0.4277092	119	C	-4.2521011	2.3025537	2.0136004
81	C	10.8322624	5.0524245	1.1074571	120	C	-6.4029981	1.2270561	1.6528453
82	C	12.2215203	5.2867755	0.4848638	121	C	-6.4029954	-1.2270775	1.6528471
83	C	10.9831493	5.082258	2.6471118	122	C	-4.2520958	-2.302569	2.0136052
84	C	9.9042555	6.2076833	0.6618574	123	H	-3.1702724	2.2836763	1.934882
85	H	12.6144722	6.2552979	0.8141861	124	C	-4.8859712	3.3820385	2.6222823
86	H	12.9406609	4.518119	0.7899073	125	C	-7.0273429	2.3196675	2.2719877
87	H	12.1804779	5.3024681	-0.6102759	126	C	-7.1378161	-0.0000116	1.2276995
88	H	10.3240401	7.1738838	0.9677654	127	C	-7.0273366	-2.3196859	2.2719989
89	H	9.7866334	6.2175744	-0.4277667	128	C	-4.8859629	-3.3820529	2.6222918
90	H	8.9060865	6.123259	1.1039841	129	H	-3.1702671	-2.2836899	1.9348858
91	H	11.4137224	6.0382007	2.9698098	130	C	-6.2775296	3.3917247	2.748545
92	H	10.0197769	4.9631638	3.1538755	131	H	-4.2944536	4.2057982	3.0127918
93	H	11.6432301	4.2780852	2.9916025	132	H	-8.1043849	2.3151902	2.3979925
94	C	-0.5972484	-0.0000026	-0.3282225	133	C	-8.3184154	-0.0000096	0.552652
95	C	-1.3405033	1.2215491	-0.7518974	134	C	-6.2775207	-3.39174	2.7485589
96	C	-1.3404995	-1.2215573	-0.7518987	135	H	-8.1043777	-2.3152087	2.3980088
97	C	-2.7448439	1.2222556	-0.5764392	136	H	-4.294443	-4.2058099	3.0128034
98	C	-0.7307993	2.2974717	-1.4139109	137	H	-6.7770731	4.2242291	3.2367935
99	C	-2.7448401	-1.2222683	-0.5764399	138	C	-8.9985853	1.2489166	0.1032842
100	C	-0.7307954	-2.297471	-1.4139273	139	C	-8.9985827	-1.2489291	0.1032604
101	C	-3.4956389	2.3010131	-1.0671692	140	H	-6.7770614	-4.2242425	3.2368137
102	C	-3.3635898	-0.0000075	0.0144899	141	C	-10.3012638	1.5708713	0.5438017
103	C	-1.4880495	3.3657536	-1.8852258	142	C	-8.3525032	2.1392067	-0.7672443
104	H	0.3420236	2.2825396	-1.5734006	143	C	-10.3012608	-1.5708952	0.5437705
105	C	-3.495633	-2.3010254	-1.0671743	144	C	-8.352501	-2.1391976	-0.7672908
106	C	-1.4880434	-3.3657525	-1.8852464	145	C	-11.1127585	0.6760611	1.3692431
107	H	0.342026	-2.2825327	-1.5734249	146	C	-10.8623499	2.8098728	0.1677163
108	H	-4.5743395	2.2923035	-0.953718	147	C	-8.9447411	3.3334696	-1.1551589
109	C	-2.8747904	3.3675896	-1.7106337	148	H	-7.3604968	1.887131	-1.1307974
110	C	-4.3677992	-0.0000088	0.9363389	149	C	-11.1127574	-0.6761032	1.3692297
111	H	-1.0002696	4.1855401	-2.4056948	150	C	-10.8623443	-2.8098899	0.1676594
112	C	-2.8747832	-3.367596	-1.7106471	151	C	-8.9447368	-3.3334535	-1.1552307
113	H	-4.5743335	-2.292319	-0.9537218	152	H	-7.3604966	-1.8871112	-1.1308416

Tag	Symbol	X	Y	Z
153	H	-11.8771845	1.1676757	1.9697505
154	C	-10.2157782	3.7084555	-0.680186
155	H	-11.8516487	3.0414837	0.5487371
156	H	-8.3992813	3.9906507	-1.8265757
157	H	-11.8771828	-1.1677308	1.9697269
158	C	-10.2157716	-3.7084533	-0.6802627
159	H	-11.8516424	-3.0415109	0.5486758
160	H	-8.3992769	-3.9906181	-1.8266637
161	C	-10.8322691	5.0524204	-1.1074365
162	C	-10.8322606	-5.0524099	-1.1075422
163	C	-12.2215328	5.2867611	-0.4848523
164	C	-10.9831439	5.0822653	-2.6470922
165	C	-9.9042703	6.2076794	-0.6618205
166	C	-9.9042561	-6.2076767	-0.6619584
167	C	-12.2215205	-5.2867698	-0.4849568
168	C	-10.983143	-5.0822185	-2.6471979
169	H	-12.6144841	6.2552865	-0.8141666
170	H	-12.9406692	4.5181066	-0.7899109
171	H	-12.1804997	5.3024409	0.610288
172	H	-11.4137171	6.0382093	-2.9697862
173	H	-10.0197672	4.9631776	-3.1538492
174	H	-11.6432198	4.2780933	-2.9915943
175	H	-10.3240566	7.1738806	-0.967724
176	H	-9.7866565	6.2175624	0.4278045
177	H	-8.9060976	6.1232629	-1.1039403
178	H	-10.3240408	-7.173872	-0.967883
179	H	-8.9060858	-6.1232463	-1.1040811
180	H	-9.786637	-6.2175852	0.4276658
181	H	-12.6144718	-6.255287	-0.814295
182	H	-12.1804815	-5.302479	0.6101828
183	H	-12.9406599	-4.5181084	-0.7899909
184	H	-11.6432224	-4.2780399	-2.9916775
185	H	-10.019769	-4.9631165	-3.1539568
186	H	-11.4137154	-6.0381558	-2.9699126

5T (triplet): UB3LYP/6-31G(d)

SCF Done: E(UB3LYP) = -4475.83044064 A.U. after 5 cycles									
Tag	Symbol	X	Y	Z	Tag	Symbol	X	Y	Z
1	C	10.1663196	0.0000001	-0.0001654	38	H	9.9850591	-0.0008044	-4.6090769
2	C	9.455098	0.0002165	1.2211189	39	H	11.2193747	-0.0004399	-2.482775
3	C	10.134915	0.0004373	2.4821346	40	H	15.1777115	-3.1570548	0.0003095
4	C	9.4459791	0.0006391	3.6653065	41	H	11.1277646	-4.5543115	0.0005536
5	C	8.0232912	0.0006316	3.6663604	42	H	10.354799	-2.29004	0.0002098
6	C	7.3311773	0.0004243	2.48503	43	H	15.1777129	3.1570522	-0.0007194
7	C	8.0083491	0.0002117	1.2222499	44	H	11.1277667	4.554311	-0.0011095
8	C	7.2994319	0.0000003	-0.0001105	45	H	10.3548002	2.2900401	-0.0006926
9	C	8.0083022	-0.0002111	-1.2224983	46	C	2.9336415	0.0000012	-0.0000327
10	C	7.3310826	-0.0004237	-2.4852526	47	C	3.6417277	-1.2225421	0.0001117
11	C	8.0231518	-0.000631	-3.6666092	48	C	2.9631374	-2.4844481	0.0002819
12	C	9.4458396	-0.0006391	-3.6656094	49	C	3.653623	-3.6665766	0.0004256
13	C	10.1348201	-0.0004372	-2.4824635	50	C	5.0762357	-3.6665713	0.0004078
14	C	9.4550508	-0.0002161	-1.2214224	51	C	5.7667475	-2.4844617	0.0002447
15	C	11.6779148	-0.0000003	-0.0001907	52	C	5.0885173	-1.2224202	0.0000896
16	C	12.2896902	-1.3191948	0.0000313	53	C	5.7972016	0.0000006	-0.0000823
17	C	13.6913326	-1.6358357	0.0000791	54	C	5.0885178	1.2224219	-0.0002286
18	C	14.1069108	-2.9835752	0.0002803	55	C	5.7667483	2.4844631	-0.0004066
19	C	13.2407425	-4.0746913	0.0004523	56	C	5.0762368	3.666573	-0.0005453
20	C	11.8701594	-3.7607374	0.0004204	57	C	3.6536242	3.6665787	-0.0005145
21	C	11.4236785	-2.4523267	0.0002212	58	C	2.9631383	2.4844505	-0.0003478
22	C	12.2896908	1.3191937	-0.0004442	59	C	3.6417282	1.2225443	-0.0002007
23	C	13.6913336	1.6358339	-0.000468	60	H	1.8787235	-2.4878615	0.0002947
24	C	14.1069124	2.9835732	-0.0007128	61	H	3.1151462	-4.6104584	0.0005535
25	C	13.2407447	4.0746898	-0.0009494	62	H	5.6145753	-4.6105367	0.0005233
26	C	11.8701613	3.7607366	-0.0009308	63	H	6.8511297	-2.4874494	0.0002289
27	C	11.4236797	2.4523261	-0.0006895	64	H	6.8511306	2.4874506	-0.0004276
28	C	14.7769538	0.6744413	-0.00026	65	H	5.6145767	4.6105382	-0.0006785
29	C	14.7769534	-0.6744438	-0.000042	66	H	3.1151476	4.6104606	-0.0006236
30	H	15.7632878	1.1352323	-0.0002998	67	H	1.8787243	2.4878644	-0.0003236
31	H	15.7632871	-1.1352355	0.0000654	68	C	1.4314547	0.0000014	-0.0000079
32	H	11.2194695	0.0004396	2.4824058	69	C	0.7234032	0.0001384	1.2226183
33	H	9.9852343	0.0008042	4.6087534	70	C	1.4019602	0.00028	2.484525
34	H	7.4856294	0.0007901	4.6107871	71	C	0.7113744	0.000412	3.6666141
35	H	6.2466931	0.0004167	2.4900652	72	C	-0.7112549	0.0004107	3.6666373
36	H	6.2465982	-0.0004159	-2.490247	73	C	-1.4018795	0.0002775	2.4845706
37	H	7.4854541	-0.0007895	-4.6110155	74	C	-0.7233638	0.0001375	1.2226419

Tag	Symbol	X	Y	Z
75	C	-1.4314552	0.0000011	0.000039
76	C	-0.7234039	-0.0001356	-1.2225873
77	C	-1.4019607	-0.0002762	-2.4844938
78	C	-0.7113748	-0.0004096	-3.6665831
79	C	0.7112544	-0.0004105	-3.6666062
80	C	1.4018788	-0.0002776	-2.4845397
81	C	0.7233632	-0.0001362	-1.2226109
82	H	1.2497902	0.0005179	4.6105153
83	H	-1.2496398	0.0005153	4.6105562
84	H	-2.4862839	0.0002753	2.4879928
85	H	-2.4863652	-0.0002741	-2.4878809
86	H	-1.2497906	-0.0005147	-4.6104843
87	H	1.2496395	-0.0005168	-4.6105251
88	H	2.4862835	-0.0002771	-2.487962
89	C	-10.1663199	0.0000005	0.0001763
90	C	-9.4550948	-0.0001833	-1.2211057
91	C	-10.1349069	-0.0003643	-2.4821241
92	C	-9.4459668	-0.0005356	-3.6652935
93	C	-8.023279	-0.0005345	-3.6663424
94	C	-7.3311694	-0.0003617	-2.4850095
95	C	-8.0083459	-0.0001805	-1.2222319
96	C	-7.2994322	0.0000009	0.0001306
97	C	-8.0083064	0.0001821	1.2225163
98	C	-7.3310915	0.0003629	2.4852732
99	C	-8.0231651	0.0005351	3.6666274
100	C	-9.4458529	0.0005359	3.6656225
101	C	-10.1348291	0.0003649	2.482474
102	C	-9.4550549	0.0001843	1.2214356
103	C	-11.6779152	-0.0000001	0.0001903
104	C	-12.28969	-1.3191948	0.0003753
105	C	-13.6913326	-1.6358362	0.0002625
106	C	-14.1069101	-2.9835756	0.0003813
107	C	-13.2407412	-4.0746914	0.0006413
108	C	-11.8701585	-3.7607371	0.0007985
109	C	-11.4236781	-2.4523262	0.0006687
110	C	-12.2896913	1.3191938	0.0000325
111	C	-13.6913348	1.6358339	-0.0000162
112	C	-14.1069137	2.9835723	-0.0001066
113	C	-13.2407457	4.0746895	-0.000179

Tag	Symbol	X	Y	Z
114	C	-11.8701631	3.7607369	-0.0001786
115	C	-11.4236811	2.4523262	-0.0000809
116	C	-14.7769544	0.6744405	-0.0000324
117	C	-14.7769535	-0.6744445	0.0000517
118	H	-15.7632886	1.1352311	-0.000152
119	H	-15.7632872	-1.1352365	-0.0000165
120	H	-11.2194613	-0.0003612	-2.4824
121	H	-9.9852186	-0.0006709	-4.6087424
122	H	-7.4856141	-0.00067	-4.6107674
123	H	-6.2466851	-0.0003578	-2.4900406
124	H	-6.2466071	0.0003591	2.4902715
125	H	-7.4854709	0.0006702	4.6110357
126	H	-9.9850758	0.0006705	4.6090879
127	H	-11.2193837	0.0003612	2.4827809
128	H	-15.1777108	-3.1570559	0.0002758
129	H	-11.1277632	-4.5543107	0.0010258
130	H	-10.3547985	-2.2900396	0.0008036
131	H	-15.1777142	3.1570518	-0.0001303
132	H	-11.1277682	4.5543112	-0.0002599
133	H	-10.3548013	2.2900415	-0.0000886
134	C	-2.9336419	0.000001	0.0000631
135	C	-3.6417282	-1.2225422	0.0002172
136	C	-2.963138	-2.4844484	0.0003531
137	C	-3.6536237	-3.6665766	0.0005039
138	C	-5.0762365	-3.6665712	0.0005285
139	C	-5.7667483	-2.4844614	0.0004007
140	C	-5.0885178	-1.22242	0.0002406
141	C	-5.797202	0.0000009	0.0001069
142	C	-5.0885179	1.2224221	-0.0000491
143	C	-5.7667483	2.4844633	-0.0001904
144	C	-5.0762368	3.6665732	-0.0003402
145	C	-3.6536243	3.6665787	-0.0003576
146	C	-2.9631385	2.4844504	-0.0002262
147	C	-3.6417283	1.2225443	-0.0000696
148	H	-1.8787241	-2.487862	0.0003332
149	H	-3.115147	-4.6104584	0.0006051
150	H	-5.614576	-4.6105365	0.0006481
151	H	-6.8511306	-2.4874488	0.0004164
152	H	-6.8511306	2.4874511	-0.0001759

Tag	Symbol	X	Y	Z
153	H	-5.6145766	4.6105385	-0.0004457
154	H	-3.1151475	4.6104605	-0.0004755
155	H	-1.8787246	2.4878644	-0.0002388
156	H	2.4863647	0.0002795	2.4879119
157	C	13.7114861	-5.5365267	0.0006555
158	C	13.1691968	-6.2495553	1.2627333
159	H	12.075062	-6.2302093	1.3031811
160	H	13.4849362	-7.3000906	1.2727254
161	H	13.5456631	-5.7722129	2.1745939
162	C	13.1691706	-6.249893	-1.2612204
163	H	12.0750348	-6.2305362	-1.3016509
164	H	13.5456272	-5.7728016	-2.1732158
165	H	13.4848944	-7.3004364	-1.2709353
166	C	15.2468192	-5.6595439	0.0006601
167	H	15.6930265	-5.1974751	-0.887376
168	H	15.6930428	-5.1971336	0.8885109
169	H	15.5324025	-6.7175405	0.0008571
170	C	13.7114889	5.5365247	-0.0011999
171	C	13.1691634	6.2495266	-1.2632767
172	H	12.0750276	6.2301715	-1.3036987
173	H	13.4848937	7.3000643	-1.2732935
174	H	13.5456119	5.7721727	-2.1751385
175	C	13.1692096	6.2499179	1.2606771
176	H	12.0750747	6.2305743	1.3011314
177	H	13.5456806	5.7728357	2.1726712
178	H	13.4849464	7.3004576	1.2703688
179	C	15.2468221	5.6595416	-0.0012534
180	H	15.6930593	5.197455	0.8867585
181	H	15.6930157	5.197149	-0.8891282
182	H	15.5324057	6.7175381	-0.0014388
183	C	-13.7114896	5.5365237	-0.0002654
184	C	-15.246823	5.6595422	-0.0003429
185	H	-15.6929925	5.1973191	-0.8883179
186	H	-15.6930859	5.1972878	0.8875692
187	H	-15.5324048	6.7175394	-0.0003394
188	C	-13.169127	6.2496697	-1.2622455
189	H	-12.0749897	6.2303172	-1.3026349
190	H	-13.5455472	5.7724176	-2.1741721
191	H	-13.4848576	7.3002084	-1.2721535

Tag	Symbol	X	Y	Z
192	C	-13.1692427	6.2497698	1.2617078
193	H	-13.5457157	5.7725654	2.1736372
194	H	-12.0751079	6.2304488	1.3021774
195	H	-13.4850045	7.3003006	1.2715264
196	C	-13.7114838	-5.5365265	0.0007371
197	C	-13.1693417	-6.2496055	1.2628494
198	H	-13.4850865	-7.3001397	1.272763
199	H	-12.0752113	-6.2302669	1.3034252
200	H	-13.5459116	-5.7722981	2.1746855
201	C	-15.2468168	-5.6595457	0.0005575
202	H	-15.6931444	-5.19717	0.8883741
203	H	-15.6929218	-5.1974436	-0.8875127
204	H	-15.5323988	-6.7175427	0.0006797
205	C	-13.1690174	-6.2498402	-1.2611043
206	H	-13.5453572	-5.7727038	-2.1731242
207	H	-12.0748764	-6.2304876	-1.3013974
208	H	-13.4847475	-7.300381	-1.2709068

5F: B3LYP/6-31G(d)

SCF Done: E(RB3LYP) = -4475.78376262 A.U. after 5 cycles									
Tag	Symbol	X	Y	Z	Tag	Symbol	X	Y	Z
1	C	6.4049801	2.3022994	-2.0525478	38	C	10.1520775	2.139521	1.1934745
2	H	5.3214558	2.2831728	-2.1028734	39	C	12.0129358	3.7088695	1.321436
3	C	7.0834734	1.2229768	-1.4687372	40	C	10.6956498	3.3339284	1.6468762
4	C	7.1063246	3.3816215	-2.5823906	41	H	13.7794865	3.0417911	0.2892499
5	C	8.5030198	3.3914392	-2.5429636	42	H	10.0764209	3.9912723	2.2507678
6	C	8.4981452	1.2271236	-1.4392881	43	H	9.1246572	1.887578	1.4399854
7	C	9.1912295	2.3196558	-1.980558	44	H	13.9688305	1.1677978	-1.1187569
8	H	6.5651261	4.2050535	-3.0406767	45	H	13.9688901	-1.1675294	-1.1187692
9	H	9.0567932	4.2237433	-2.9690951	46	C	12.5767836	-5.0526691	1.8164582
10	H	10.2755927	2.315341	-1.978362	47	C	12.5502364	-5.0823281	3.3632706
11	C	9.1777353	0.0000198	-0.9304796	48	C	11.70605	-6.208112	1.2677184
12	C	6.3923191	-0.0000432	-0.969151	49	C	14.028214	-5.2867676	1.3570589
13	C	8.4982037	-1.2270967	-1.439333	50	H	12.088331	-7.1742179	1.6196156
14	C	7.0835308	-1.223014	-1.4687735	51	H	10.663886	-6.1239005	1.5929203
15	C	9.1913371	-2.3195723	-1.9806553	52	H	11.7137485	-6.2180652	0.1717966
16	C	6.405087	-2.3023429	-2.0526276	53	H	14.1128593	-5.3021574	0.2644266
17	C	8.5031736	-3.3913662	-2.5430989	54	H	14.7076073	-4.5181479	1.7426592
18	C	7.106478	-3.3816118	-2.5825161	55	H	14.3810115	-6.2553411	1.7289473
19	H	6.5653143	-4.2050514	-3.0408298	56	H	12.9412904	-6.0380779	3.7333496
20	H	9.0569821	-4.2236291	-2.9692651	57	H	13.1661576	-4.2778901	3.7811165
21	H	10.2757	-2.3152052	-1.9784683	58	H	11.5350319	-4.9635566	3.7561308
22	H	5.3215618	-2.2832624	-2.1029476	59	C	12.5765331	5.0528346	1.816514
23	C	10.2709376	0.000037	-0.1215417	60	C	14.0279825	5.2869685	1.3571935
24	C	10.8943365	1.249026	0.4035886	61	C	12.5498974	5.0825063	3.3633247
25	C	10.8943987	-1.2489289	0.4035718	62	C	11.7057997	6.2082496	1.2677151
26	C	12.2390874	1.5709844	0.1160567	63	H	14.3807175	6.2555752	1.7290549
27	C	12.2391661	-1.570817	0.1160382	64	H	14.7073837	4.5183969	1.7428754
28	C	13.1403277	0.6761872	-0.610301	65	H	14.1126947	5.3023054	0.2645654
29	C	13.1403619	-0.6759664	-0.6103086	66	H	12.088045	7.1743694	1.6196134
30	C	12.7530786	-2.8099438	0.5538625	67	H	11.7135483	6.2181846	0.1717934
31	C	12.0131211	-3.7087248	1.3213977	68	H	10.6636227	6.1240237	1.5928709
32	C	10.1521844	-2.1394687	1.19345	69	H	12.9408921	6.038275	3.7334178
33	C	10.6958161	-3.3338534	1.6468404	70	H	11.5346748	4.9636969	3.7561271
34	H	13.7796388	-3.0415476	0.2892189	71	H	13.1658268	4.2780965	3.7812127
35	H	10.0766188	-3.9912361	2.250722	72	C	5.2857191	2.3001998	0.9175173
36	H	9.124753	-1.8875772	1.4399668	73	H	6.3702374	2.2915446	0.9352915
37	C	12.7529385	2.8101325	0.5538932	74	C	4.5997633	1.222038	0.3385792

Tag	Symbol	X	Y	Z
75	C	4.5915506	3.3659561	1.4827484
76	C	3.1938821	3.3639942	1.4887472
77	C	3.1845267	1.2213032	0.3431964
78	C	2.4991707	2.2963472	0.9280496
79	H	5.1397821	4.1873021	1.9363456
80	H	2.6468315	4.1831589	1.9476254
81	H	1.4149029	2.2811196	0.9570158
82	C	2.4983069	-0.0000898	-0.1680685
83	C	5.2850982	-0.0000776	-0.1740072
84	C	3.1845313	-1.2215155	0.3431044
85	C	4.5997692	-1.2222388	0.3384829
86	C	2.4991863	-2.2966029	0.9278895
87	C	5.2857354	-2.3004298	0.9173524
88	C	3.1939092	-3.3642818	1.4885128
89	C	4.5915778	-3.3662313	1.4825118
90	H	5.139817	-4.1876044	1.9360507
91	H	2.6468665	-4.1834839	1.9473339
92	H	1.4149185	-2.2813846	0.9568666
93	H	6.3702539	-2.2917645	0.9351264
94	C	-1.3933082	2.298201	-2.0592762
95	H	-2.4776432	2.2846643	-2.0849934
96	C	-0.7076575	1.2217169	-1.4775093
97	C	-0.6988218	3.3651263	-2.6219353
98	C	0.698829	3.3651036	-2.6219619
99	C	0.7076347	1.2216992	-1.4775257
100	C	1.3933023	2.2981573	-2.0593229
101	H	-1.246462	4.185026	-3.0787594
102	H	1.2464762	4.1849893	-3.0788023
103	H	2.4776349	2.2845875	-2.0850734
104	C	1.3931943	-0.0000614	-0.9657638
105	C	-1.3932399	-0.0000302	-0.9657557
106	C	0.7076058	-1.2217854	-1.4775718
107	C	-0.7076856	-1.2217725	-1.4775635
108	C	1.3932472	-2.2982381	-2.0594074
109	C	-1.3933632	-2.2982237	-2.0593628
110	C	0.6987528	-3.3651487	-2.6220866
111	C	-0.6988978	-3.3651437	-2.6220599
112	H	-1.2465533	-4.1850177	-3.0789118
113	H	1.2463844	-4.1850269	-3.0789592

Tag	Symbol	X	Y	Z
114	H	2.4775802	-2.2846912	-2.0851539
115	H	-2.4776977	-2.2846673	-2.0850743
116	C	-6.4051358	-2.3023842	-2.0525412
117	H	-5.3216109	-2.2832912	-2.1028893
118	C	-7.08358	-1.223044	-1.4687152
119	C	-7.1065313	-3.3816898	-2.5823549
120	C	-8.5032242	-3.3914677	-2.5428808
121	C	-8.4982513	-1.2271489	-1.4392189
122	C	-9.1913882	-2.3196607	-1.9804582
123	H	-6.5653733	-4.2051423	-3.0406524
124	H	-9.0570324	-4.2237612	-2.9689878
125	H	-10.2757513	-2.315319	-1.9782164
126	C	-9.1777793	-0.0000184	-0.9303919
127	C	-6.3923732	-0.0000407	-0.9691533
128	C	-8.4982318	1.2270845	-1.4392588
129	C	-7.0835613	1.2229576	-1.4687553
130	C	-9.191352	2.319591	-1.9805298
131	C	-6.4051001	2.3022683	-2.0526167
132	C	-8.503172	3.3913692	-2.5429874
133	C	-7.1064795	3.3815677	-2.5824643
134	H	-6.5653102	4.204997	-3.0407899
135	H	-9.0569681	4.2236573	-2.9691207
136	H	-10.2757152	2.3152671	-1.9782845
137	H	-5.3215762	2.2831573	-2.1029642
138	C	-10.2709107	0.0000082	-0.1213726
139	C	-10.8943343	-1.2489547	0.4037861
140	C	-10.8943195	1.2490086	0.4037125
141	C	-12.2390811	-1.5708955	0.116206
142	C	-12.2390635	1.5709473	0.1161151
143	C	-13.1403041	-0.6760699	-0.6101169
144	C	-13.1402966	0.6760894	-0.6101553
145	C	-12.7529171	2.8101323	0.5538465
146	C	-12.0129306	3.7089133	1.3213531
147	C	-10.1520787	2.1395435	1.1935615
148	C	-10.69566	3.3339846	1.6468667
149	H	-13.7794603	3.0417754	0.2891707
150	H	-10.0764439	3.9913738	2.2507224
151	H	-9.1246653	1.8876111	1.4401159
152	C	-12.7529491	-2.8100485	0.5540113

Tag	Symbol	X	Y	Z
153	C	-10.1521056	-2.1394484	1.193693
154	C	-12.0129741	-3.7087913	1.3215741
155	C	-10.6957007	-3.3338564	1.6470694
156	H	-13.7794943	-3.0416965	0.2893474
157	H	-10.0764941	-3.9912147	2.2509684
158	H	-9.1246908	-1.8875111	1.4402364
159	H	-13.9688671	-1.1676466	-1.1185076
160	H	-13.9688539	1.1676462	-1.1185744
161	C	-12.5765031	5.052958	1.8162434
162	C	-12.5498568	5.082907	3.3630461
163	C	-11.7057396	6.2082511	1.2672293
164	C	-14.0279587	5.2870396	1.3569133
165	H	-12.0879077	7.1744475	1.6190027
166	H	-10.6635507	6.1239999	1.5923438
167	H	-11.7135433	6.2180229	0.1713067
168	H	-14.1126825	5.3023211	0.2642854
169	H	-14.7073372	4.5184697	1.7426396
170	H	-14.3807092	6.2556619	1.7287184
171	H	-12.9408424	6.038749	3.7329574
172	H	-13.1657986	4.2785834	3.7810824
173	H	-11.5346414	4.9641671	3.7558855
174	C	-12.5765605	-5.0528025	1.8165405
175	C	-14.0280287	-5.2868821	1.3572486
176	C	-12.5498878	-5.0826798	3.3633438
177	C	-11.7058248	-6.2081348	1.2675644
178	H	-14.3807859	-6.2554839	1.7291007
179	H	-14.7073877	-4.518286	1.7429572
180	H	-14.112775	-5.3022075	0.264623
181	H	-12.0880006	-7.1743094	1.6193894
182	H	-11.7136483	-6.217957	0.1716424
183	H	-10.663629	-6.123883	1.5926564
184	H	-12.94089	-6.0384955	3.7333057
185	H	-11.5346634	-4.9639454	3.7561613
186	H	-13.1658042	-4.2783226	3.7813533
187	C	-5.285768	-2.3003936	0.9173449
188	H	-6.3702869	-2.2917393	0.9351108
189	C	-4.5998129	-1.2221893	0.3384883
190	C	-4.5916012	-3.3661961	1.4824912
191	C	-3.1939323	-3.3642396	1.4884845

Tag	Symbol	X	Y	Z
192	C	-3.1845761	-1.2214568	0.3431078
193	C	-2.4992189	-2.2965525	0.9278666
194	H	-5.1398333	-4.1875799	1.9360191
195	H	-2.6468831	-4.1834443	1.9472932
196	H	-1.4149499	-2.2813329	0.9568247
197	C	-2.4983552	-0.0000361	-0.168079
198	C	-5.2851537	-0.0000413	-0.1740122
199	C	-3.1845805	1.2213753	0.3431214
200	C	-4.5998181	1.2221021	0.3385082
201	C	-2.4992314	2.2964583	0.9279086
202	C	-5.2857804	2.3002895	0.9173896
203	C	-3.193948	3.364131	1.4885497
204	C	-4.5916169	3.3660835	1.482557
205	H	-5.1398502	4.1874569	1.9361022
206	H	-2.6468998	4.1833257	1.9473775
207	H	-1.4149624	2.2812382	0.9568738
208	H	-6.3702991	2.2916297	0.935159

6T (triplet): UB3LYP/6-31G(d)

SCF Done: E(UB3LYP) = -5014.15873687 A.U. after 6 cycles									
**Tag**	**Symbol**	**X**	**Y**	**Z**	**Tag**	**Symbol**	**X**	**Y**	**Z**
1	C	0.7510346	0.0000015	-0.0001532	38	H	7.7966384	3.2601958	3.2600662
2	C	1.4590022	-0.8645493	0.8644097	39	H	5.2972443	3.2601486	3.2599788
3	C	0.7804726	-1.7568638	1.7567237	40	H	4.060779	1.7593586	1.7591782
4	C	1.4711506	-2.5926858	2.5925588	41	H	4.0608632	-1.7593122	-1.7594412
5	C	2.8937748	-2.5926628	2.5925521	42	H	5.2974007	-3.2601013	-3.2601826
6	C	3.5843699	-1.7568003	1.7566931	43	H	7.7967948	-3.2601244	-3.260174
7	C	2.9057602	-0.8645238	0.8644024	44	H	9.0333073	-1.7588901	-1.7589528
8	C	3.6137859	0.0000197	-0.0001347	45	C	12.3483542	0.0000265	0.0000656
9	C	2.9057608	0.8645561	-0.8646809	46	C	11.6371267	0.8635894	-0.8635331
10	C	3.5843699	1.7568475	-1.756957	47	C	12.3169901	1.7552391	-1.7551914
11	C	2.8937745	2.5927058	-2.5928203	48	C	11.6280585	2.5918528	-2.5918399
12	C	1.4711507	2.5927116	-2.5928441	49	C	10.205374	2.5925988	-2.5926158
13	C	0.7804729	1.7568767	-1.7570214	50	C	9.5132054	1.7572931	-1.7573057
14	C	1.4590023	0.8645636	-0.8647063	51	C	10.1903882	0.8643906	-0.8643675
15	H	-0.3039451	-1.7594293	1.759277	52	C	9.4814801	0.0000411	-0.000019
16	H	0.9328	-3.2601525	3.2600241	53	C	10.1903279	-0.8643173	0.8643683
17	H	3.4321743	-3.2600992	3.259999	54	C	9.5130852	-1.7572146	1.7572667
18	H	4.6687799	-1.7592562	1.759162	55	C	10.2051981	-2.59253	2.5926131
19	H	4.6687797	1.7593184	-1.7594126	56	C	11.6278832	-2.5918005	2.5919171
20	H	3.4321741	3.2601528	-3.2602564	57	C	12.3168704	-1.7551919	1.7553096
21	H	0.9327999	3.2601761	-3.2603115	58	C	11.6370653	-0.8635322	0.8636163
22	H	-0.3039447	1.7594302	-1.7595874	59	C	13.859919	0.0000227	0.0000996
23	C	7.9793283	0.0000466	-0.0000602	60	C	14.4716932	0.9328066	0.9329561
24	C	7.2706286	0.8644432	0.8643151	61	C	15.8733494	1.156721	1.1567666
25	C	7.9488537	1.7568289	1.7567098	62	C	16.2890452	2.1096884	2.1097453
26	C	7.25832	2.5927076	2.5925725	63	C	15.4230003	2.8812286	2.8814174
27	C	5.8357121	2.5927138	2.5925558	64	C	14.0523904	2.6592083	2.6595769
28	C	5.1452048	1.7568304	1.7566666	65	C	13.6058114	1.734041	1.7343876
29	C	5.8238435	0.8645355	0.8643858	66	C	14.4717128	-0.9327867	-0.9327149
30	C	5.1158499	0.0000342	-0.0001163	67	C	15.8733721	-1.1566077	-1.1565948
31	C	5.8238845	-0.8644622	-0.8645902	68	C	16.2890823	-2.1095695	-2.1095727
32	C	5.1452889	-1.7567726	-1.7568882	69	C	15.4230483	-2.8811989	-2.881169
33	C	5.8358365	-2.5926556	-2.5927447	70	C	14.052435	-2.6593234	-2.6592042
34	C	7.2584441	-2.592636	-2.5927064	71	C	13.6058424	-1.7341566	-1.7340209
35	C	7.9489377	-1.7567452	-1.7568224	72	C	16.9590172	-0.4768044	-0.4768351
36	C	7.2706698	-0.864357	-0.864463	73	C	16.9590075	0.4769889	0.4769561
37	H	9.0332232	1.7589841	1.7588818	74	H	17.9453455	-0.8026239	-0.8026916

Tag	Symbol	X	Y	Z
75	H	17.9453294	0.8028561	0.8027844
76	H	13.4015445	1.7554724	-1.7554034
77	H	12.1673168	3.2589659	-3.2589587
78	H	9.6677449	3.2604095	-3.2604541
79	H	8.4287111	1.7609537	-1.7609916
80	H	8.4285904	-1.7608629	1.7608911
81	H	9.6675241	-3.2603363	3.2604198
82	H	12.1670961	-3.2589216	3.2590646
83	H	13.4014252	-1.7554339	1.7555799
84	H	17.3598787	2.2322536	2.2322238
85	H	13.3099944	3.220268	3.2208009
86	H	12.5369071	1.6193509	1.6198461
87	H	17.3599175	-2.2320642	-2.2321055
88	H	13.310047	-3.2205016	-3.2203204
89	H	12.536936	-1.6195822	-1.6193756
90	C	15.8940161	3.9148523	3.9150179
91	C	15.3519112	3.5269752	5.3118092
92	H	15.6679357	4.2628704	6.0614807
93	H	14.2577719	3.4849272	5.3269647
94	H	15.72824	2.5446688	5.6192415
95	C	15.3518624	5.3116177	3.5271184
96	H	15.6678917	6.0613262	4.2629733
97	H	15.7281433	5.6190345	2.5447871
98	H	14.2577216	5.3267385	3.4851083
99	C	17.4293379	4.0015894	4.0017319
100	H	17.8753947	3.0467527	4.3026008
101	H	17.8753792	4.3025323	3.0469094
102	H	17.7152393	4.7496167	4.7498033
103	C	15.8940781	-3.9147874	-3.9147985
104	C	15.351884	-5.3115648	-3.5269953
105	H	14.2577416	-5.3266666	-3.4850065
106	H	15.6679113	-6.0612286	-4.2628971
107	H	15.7281444	-5.6190467	-2.5446781
108	C	15.3520242	-3.5268211	-5.311584
109	H	15.6680654	-4.262667	-6.0612962
110	H	14.2578862	-3.4847623	-5.3267693
111	H	15.7283668	-2.5444971	-5.6189485
112	C	17.4294011	-4.0015712	-4.0014495
113	H	17.8755058	-3.0467572	-4.3023253

Tag	Symbol	X	Y	Z
114	H	17.8753879	-4.3025027	-3.0465998
115	H	17.7153106	-4.7496243	-4.7494919
116	C	-0.7510346	-0.0000015	-0.0001532
117	C	-1.4590022	0.8645493	0.8644097
118	C	-1.4590023	-0.8645636	-0.8647063
119	C	-0.7804726	1.7568638	1.7567237
120	C	-2.9057602	0.8645238	0.8644024
121	C	-2.9057608	-0.8645561	-0.8646809
122	C	-0.7804729	-1.7568767	-1.7570214
123	C	-1.4711506	2.5926858	2.5925588
124	H	0.3039451	1.7594293	1.759277
125	C	-3.5843699	1.7568003	1.7566931
126	C	-3.6137859	-0.0000197	-0.0001347
127	C	-3.5843699	-1.7568475	-1.756957
128	C	-1.4711507	-2.5927116	-2.5928441
129	H	0.3039447	-1.7594302	-1.7595874
130	C	-2.8937748	2.5926628	2.5925521
131	H	-0.9328	3.2601525	3.2600241
132	H	-4.6687799	1.7592562	1.759162
133	C	-5.1158499	-0.0000342	-0.0001163
134	C	-2.8937745	-2.5927058	-2.5928203
135	H	-4.6687797	-1.7593184	-1.7594126
136	H	-0.9327999	-3.2601761	-3.2603115
137	H	-3.4321743	3.2600992	3.259999
138	C	-5.8238435	-0.8645355	0.8643858
139	C	-5.8238845	0.8644622	-0.8645902
140	H	-3.4321741	-3.2601528	-3.2602564
141	C	-7.2706286	-0.8644432	0.8643151
142	C	-5.1452048	-1.7568304	1.7566666
143	C	-5.1452889	1.7567726	-1.7568882
144	C	-7.2706698	0.864357	-0.864463
145	C	-7.9793283	-0.0000466	-0.0000602
146	C	-7.9488537	-1.7568289	1.7567098
147	C	-5.8357121	-2.5927138	2.5925558
148	H	-4.060779	-1.7593586	1.7591782
149	C	-5.8358365	2.5926556	-2.5927447
150	H	-4.0608632	1.7593122	-1.7594412
151	C	-7.9489377	1.7567452	-1.7568224
152	C	-9.4814801	-0.0000411	-0.000019

Tag	Symbol	X	Y	Z
153	C	-7.25832	-2.5927076	2.5925725
154	H	-9.0332232	-1.7589841	1.7588818
155	H	-5.2972443	-3.2601486	3.2599788
156	C	-7.2584441	2.592636	-2.5927064
157	H	-5.2974007	3.2601013	-3.2601826
158	H	-9.0333073	1.7588901	-1.7589528
159	C	-10.1903882	-0.8643906	-0.8643675
160	C	-10.1903279	0.8643173	0.8643683
161	H	-7.7966384	-3.2601958	3.2600662
162	H	-7.7967948	3.2601244	-3.260174
163	C	-11.6371267	-0.8635894	-0.8635331
164	C	-9.5132054	-1.7572931	-1.7573057
165	C	-9.5130852	1.7572146	1.7572667
166	C	-11.6370653	0.8635322	0.8636163
167	C	-12.3483542	-0.0000265	0.0000656
168	C	-12.3169901	-1.7552391	-1.7551914
169	C	-10.205374	-2.5925988	-2.5926158
170	H	-8.4287111	-1.7609537	-1.7609916
171	C	-10.2051981	2.59253	2.5926131
172	H	-8.4285904	1.7608629	1.7608911
173	C	-12.3168704	1.7551919	1.7553096
174	C	-13.859919	-0.0000227	0.0000996
175	C	-11.6280585	-2.5918528	-2.5918399
176	H	-13.4015445	-1.7554724	-1.7554034
177	H	-9.6677449	-3.2604095	-3.2604541
178	C	-11.6278832	2.5918005	2.5919171
179	H	-9.6675241	3.2603363	3.2604198
180	H	-13.4014252	1.7554339	1.7555799
181	C	-14.4716932	-0.9328066	0.9329561
182	C	-14.4717128	0.9327867	-0.9327149
183	H	-12.1673168	-3.2589659	-3.2589587
184	H	-12.1670961	3.2589216	3.2590646
185	C	-15.8733494	-1.156721	1.1567666
186	C	-13.6058114	-1.734041	1.7343876
187	C	-15.8733721	1.1566077	-1.1565948
188	C	-13.6058424	1.7341566	-1.7340209
189	C	-16.2890452	-2.1096884	2.1097453
190	C	-16.9590075	-0.4769889	0.4769561
191	C	-14.0523904	-2.6592083	2.6595769

Tag	Symbol	X	Y	Z
192	H	-12.5369071	-1.6193509	1.6198461
193	C	-16.2890823	2.1095695	-2.1095727
194	C	-16.9590172	0.4768044	-0.4768351
195	C	-14.052435	2.6593234	-2.6592042
196	H	-12.536936	1.6195822	-1.6193756
197	C	-15.4230003	-2.8812286	2.8814174
198	H	-17.3598787	-2.2322536	2.2322238
199	H	-17.9453294	-0.8028561	0.8027844
200	H	-13.3099944	-3.220268	3.2208009
201	C	-15.4230483	2.8811989	-2.881169
202	H	-17.3599175	2.2320642	-2.2321055
203	H	-17.9453455	0.8026239	-0.8026916
204	H	-13.310047	3.2205016	-3.2203204
205	C	-15.8940161	-3.9148523	3.9150179
206	C	-15.8940781	3.9147874	-3.9147985
207	C	-15.3519112	-3.5269752	5.3118092
208	C	-15.3518624	-5.3116177	3.5271184
209	C	-17.4293379	-4.0015894	4.0017319
210	C	-15.351884	5.3115648	-3.5269953
211	C	-15.3520242	3.5268211	-5.311584
212	C	-17.4294011	4.0015712	-4.0014495
213	H	-15.6679357	-4.2628704	6.0614807
214	H	-14.2577719	-3.4849272	5.3269647
215	H	-15.72824	-2.5446688	5.6192415
216	H	-15.6678917	-6.0613262	4.2629733
217	H	-15.7281433	-5.6190345	2.5447871
218	H	-14.2577216	-5.3267385	3.4851083
219	H	-17.8753947	-3.0467527	4.3026008
220	H	-17.8753792	-4.3025323	3.0469094
221	H	-17.7152393	-4.7496167	4.7498033
222	H	-14.2577416	5.3266666	-3.4850065
223	H	-15.6679113	6.0612286	-4.2628971
224	H	-15.7281444	5.6190467	-2.5446781
225	H	-15.6680654	4.262667	-6.0612962
226	H	-14.2578862	3.4847623	-5.3267693
227	H	-15.7283668	2.5444971	-5.6189485
228	H	-17.8755058	3.0467572	-4.3023253
229	H	-17.8753879	4.3025027	-3.0465998
230	H	-17.7153106	4.7496243	-4.7494919

6F: B3LYP/6-31G(d)

SCF Done: E(RB3LYP) = -5014.09176975 A.U. after 6 cycles									
Tag	Symbol	X	Y	Z	Tag	Symbol	X	Y	Z
1	C	8.2206061	-2.3022575	2.1097873	38	C	12.1907492	-2.138996	-0.8592836
2	H	7.1362711	-2.2830889	2.0821141	39	C	14.0562388	-3.7080691	-0.8550835
3	C	8.9393287	-1.222958	1.5763139	40	C	12.7654311	-3.3330865	-1.2733775
4	C	8.882067	-3.3816929	2.6884521	41	H	15.74467	-3.0412857	0.3007139
5	C	10.2780002	-3.3915795	2.7493611	42	H	12.1909178	-3.9901278	-1.9202625
6	C	10.3524613	-1.2271082	1.6485443	43	H	11.1836114	-1.8869425	-1.1786012
7	C	11.0048764	-2.3197689	2.2379515	44	H	15.8327512	-1.1676642	1.719678
8	H	8.3093642	-4.2051875	3.1065769	45	H	15.8327267	1.1677443	1.719706
9	H	10.7997118	-4.2240165	3.2139539	46	C	14.6536936	5.051777	-1.3095222
10	H	12.0866197	-2.315499	2.3134328	47	C	14.7385072	5.0799819	-2.8542539
11	C	11.0668114	0.0000002	1.1898265	48	C	13.7456331	6.2075494	-0.8258413
12	C	8.2861694	-0.0000238	1.0278171	49	C	16.068316	5.2866919	-0.7472118
13	C	10.3524408	1.2270864	1.6485626	50	H	14.152191	7.1733906	-1.1501679
14	C	8.9393086	1.2229162	1.5763247	51	H	12.7295624	6.1229837	-1.2250913
15	C	11.0048376	2.3197529	2.2379822	52	H	13.6744576	6.2184464	0.2677855
16	C	8.2205673	2.302199	2.1098037	53	H	16.0742807	5.3030629	0.3486733
17	C	10.2779426	3.3915467	2.7493981	54	H	16.7738726	4.5179718	-1.0823289
18	C	8.8820089	3.3816383	2.6884827	55	H	16.4466656	6.2550578	-1.0936539
19	H	8.3092913	4.2051199	3.1066126	56	H	15.154945	6.0355207	-3.1961188
20	H	10.7996388	4.2239874	3.2140016	57	H	15.3831474	4.275362	-3.2258617
21	H	12.086581	2.3154989	2.3134669	58	H	13.7542796	4.9605312	-3.3191234
22	H	7.1362327	2.2830117	2.0821277	59	C	14.6537904	-5.0516546	-1.3096351
23	C	12.2152984	0.0000183	0.4615142	60	C	16.068395	-5.286581	-0.7472839
24	C	12.8746691	-1.2488521	-0.0180178	61	C	14.7386583	-5.0798093	-2.8543648
25	C	12.8746446	1.2489125	-0.0179896	62	C	13.7457182	-6.2074466	-0.8260239
26	C	14.195566	-1.5707791	0.3644435	63	H	16.4467685	-6.2549258	-1.0937589
27	C	14.1955347	1.5708565	0.3644804	64	H	16.7739548	-4.517836	-1.0823374
28	C	15.0426318	-0.6760434	1.1534228	65	H	16.0743196	-5.3030059	0.3486005
29	C	15.0426181	0.6761196	1.1534388	66	H	14.1522871	-7.1732749	-1.150375
30	C	14.7394787	2.8097497	-0.0362117	67	H	13.6745106	-6.2183844	0.2676004
31	C	14.0561661	3.7081724	-0.8549964	68	H	12.7296589	-6.1228662	-1.2253007
32	C	12.1907103	2.139059	-0.8592399	69	H	15.1551219	-6.0353316	-3.1962445
33	C	12.7653695	3.3331695	-1.2733066	70	H	13.7544462	-4.9603585	-3.3192662
34	H	15.7446086	3.041398	0.3007897	71	H	15.3833007	-4.2751689	-3.2259244
35	H	12.1908485	3.990209	-1.9201865	72	C	7.3224872	-2.3009817	-0.9334997
36	H	11.1835809	1.8869883	-1.1785704	73	H	8.4052473	-2.2925049	-0.8696488
37	C	14.7395344	-2.8096508	-0.036279	74	C	6.5951712	-1.2222728	-0.4085179

Tag	Symbol	X	Y	Z
75	C	6.6726443	-3.3670832	-1.5485366
76	C	5.2793989	-3.3649233	-1.6597823
77	C	5.1842799	-1.2214757	-0.5195306
78	C	4.5447164	-2.2967956	-1.1536756
79	H	7.2533157	-4.1888054	-1.9590229
80	H	4.7682824	-4.184281	-2.1580452
81	H	3.4657367	-2.2813666	-1.2642326
82	C	4.4614654	-0.0000263	-0.0616317
83	C	7.2404523	-0.000019	0.153376
84	C	5.1842772	1.2214377	-0.5194941
85	C	6.5951681	1.2222466	-0.4084796
86	C	4.5447012	2.2967773	-1.1535983
87	C	7.322472	2.3009772	-0.9334238
88	C	5.2793726	3.3649323	-1.6596625
89	C	6.672618	3.3670956	-1.5484223
90	H	7.2532818	4.1888411	-1.9588725
91	H	4.7682474	4.1843086	-2.1578859
92	H	3.4657199	2.281347	-1.2641466
93	H	8.4052322	2.2925043	-0.8695751
94	C	0.4378824	-2.2981023	1.5309042
95	H	-0.6453144	-2.2843544	1.4751471
96	C	1.1654358	-1.2217096	1.0023306
97	C	1.0879423	-3.3652925	2.1438475
98	C	2.4816571	-3.3655034	2.2486784
99	C	2.5767318	-1.2217709	1.1087037
100	C	3.2165553	-2.2985431	1.7400386
101	H	0.5074703	-4.1852264	2.5580807
102	H	2.9933731	-4.1856376	2.7450119
103	H	4.2958881	-2.2852483	1.8469121
104	C	3.2990453	-0.0000317	0.650103
105	C	0.5205156	-0.000057	0.4399749
106	C	2.5767083	1.221703	1.1086739
107	C	1.1654125	1.2216237	1.0022914
108	C	3.2165192	2.2985075	1.7399725
109	C	0.4378463	2.2980228	1.5308269
110	C	2.4816082	3.3654753	2.2485731
111	C	1.0878926	3.3652385	2.1437424
112	H	0.5074105	4.185178	2.5579498
113	H	2.9933139	4.1856311	2.7448819
114	H	4.2958534	2.2852342	1.8468427
115	H	-0.6453499	2.2842558	1.4750721
116	C	-8.2205343	-2.3022258	-2.1098047
117	H	-7.1361997	-2.2830509	-2.0821018
118	C	-8.9392726	-1.222936	-1.5763323
119	C	-8.8819791	-3.3816463	-2.6885116
120	C	-10.2779119	-3.3915297	-2.7494612
121	C	-10.3524008	-1.2270814	-1.6485958
122	C	-11.0048009	-2.3197286	-2.2380522
123	H	-8.3092656	-4.2051298	-3.106644
124	H	-10.79961	-4.2239599	-3.2140814
125	H	-12.0865414	-2.3154545	-2.3135646
126	C	-11.066763	0.0000177	-1.1898748
127	C	-8.2861258	-0.0000125	-1.0277998
128	C	-10.3523793	1.2271107	-1.6485736
129	C	-8.939251	1.2229392	-1.5763028
130	C	-11.0047557	2.3197865	-2.2380019
131	C	-8.2204903	2.3022368	-2.1097364
132	C	-10.2778465	3.3915888	-2.7493772
133	C	-8.8819135	3.3816843	-2.6884153
134	H	-8.3091862	4.2051705	-3.106523
135	H	-10.7995283	4.2240378	-3.213982
136	H	-12.0864952	2.3155293	-2.3135243
137	H	-7.1361566	2.2830451	-2.0820279
138	C	-12.2152718	0.0000228	-0.4615793
139	C	-12.8746649	-1.2488455	0.0179296
140	C	-12.8746583	1.2489045	0.017908
141	C	-14.1955408	-1.5707789	-0.3645914
142	C	-14.1955277	1.5708357	-0.3646374
143	C	-15.0425489	-0.6760596	-1.1536582
144	C	-15.0425408	0.6760999	-1.1536778
145	C	-14.7395237	2.8097086	0.0360472
146	C	-14.0562805	3.7081238	0.8548953
147	C	-12.1907941	2.1390425	0.8592247
148	C	-12.7655026	3.3331313	1.2732806
149	H	-15.7446369	3.0413445	-0.3010137
150	H	-12.1910367	3.9901677	1.9202124
151	H	-11.1836803	1.8869809	1.1786119
152	C	-14.7395433	-2.8096307	0.0361415

Tag	Symbol	X	Y	Z
153	C	-12.1907974	-2.1389682	0.8592631
154	C	-14.0563021	-3.7080257	0.8550148
155	C	-12.7655115	-3.3330392	1.2733643
156	H	-15.7446642	-3.0412651	-0.3008959
157	H	-12.1910397	-3.9900651	1.9203017
158	H	-11.1836782	-1.8869077	1.1786333
159	H	-15.8326122	-1.1676924	-1.7199807
160	H	-15.8325958	1.1677269	-1.7200176
161	C	-14.6538624	5.0517017	1.3094246
162	C	-14.7387523	5.0798665	2.8541533
163	C	-13.7458084	6.2075098	0.8258171
164	C	-16.0684635	5.2865937	0.7470503
165	H	-14.1524101	7.173332	1.1501451
166	H	-12.7297558	6.1229637	1.2251176
167	H	-13.674577	6.2184344	-0.2678058
168	H	-16.0743761	5.3029807	-0.3488348
169	H	-16.7740185	4.5178527	1.0821227
170	H	-16.4468507	6.254946	1.0934888
171	H	-15.1552297	6.0353871	3.1960211
172	H	-15.3833913	4.2752223	3.2257102
173	H	-13.7545446	4.9604277	3.3190676
174	C	-14.6538867	-5.0515896	1.3095797
175	C	-16.0684623	-5.2865274	0.747158
176	C	-14.7388459	-5.0797012	2.8543058
177	C	-13.745788	-6.2073995	0.8260572
178	H	-16.4468663	-6.2548515	1.0936569
179	H	-16.7740296	-4.5177559	1.0821343
180	H	-16.0743261	-5.3030061	-0.3487261
181	H	-14.1523765	-7.1732172	1.1504146
182	H	-13.6745156	-6.2183708	-0.2675625
183	H	-12.7297525	-6.1228031	1.2253916
184	H	-15.1553343	-6.0352134	3.1961828
185	H	-13.7546627	-4.9602437	3.319266
186	H	-15.383508	-4.275049	3.2258056
187	C	-7.3225011	-2.3010279	0.9334387
188	H	-8.4052606	-2.2925396	0.8695618
189	C	-6.5951647	-1.2223013	0.4085169
190	C	-6.672682	-3.3671551	1.5484497
191	C	-5.2794376	-3.3650017	1.6597347

Tag	Symbol	X	Y	Z
192	C	-5.1842748	-1.2215146	0.5195617
193	C	-4.5447366	-2.2968603	1.1536873
194	H	-7.2533684	-4.1888927	1.958883
195	H	-4.7683353	-4.184382	2.1579749
196	H	-3.4657601	-2.2814378	1.2642792
197	C	-4.4614462	-0.0000597	0.0617116
198	C	-7.2404286	-0.0000287	-0.1533439
199	C	-5.1842654	1.2213939	0.5195998
200	C	-6.595153	1.2222159	0.4085592
201	C	-4.5447001	2.2967114	1.1537563
202	C	-7.3224626	2.3009374	0.9335184
203	C	-5.2793751	3.3648544	1.6598383
204	C	-6.6726186	3.3670318	1.5485664
205	H	-7.2532872	4.1887685	1.9590272
206	H	-4.7682547	4.1842119	2.1580977
207	H	-3.4657216	2.2812754	1.264324
208	H	-8.4052221	2.2924702	0.8696438
209	C	-0.43781	-2.2981314	-1.5307067
210	H	0.6453866	-2.2843784	-1.4749316
211	C	-1.1653786	-1.2217286	-1.0021667
212	C	-1.0878523	-3.3653388	-2.1436334
213	C	-2.4815668	-3.3655539	-2.2484909
214	C	-2.576674	-1.2217995	-1.1085628
215	C	-3.2164782	-2.2985869	-1.739887
216	H	-0.5073701	-4.1852818	-2.5578332
217	H	-2.9932722	-4.1856988	-2.7448177
218	H	-4.2958087	-2.2852937	-1.8467857
219	C	-3.2989967	-0.0000612	-0.6499885
220	C	-0.5204713	-0.0000648	-0.4398275
221	C	-2.5766574	1.2216678	-1.1085746
222	C	-1.1653637	1.2216017	-1.0021745
223	C	-3.2164642	2.2984571	-1.7399092
224	C	-0.4377966	2.2979919	-1.5307243
225	C	-2.481553	3.365419	-2.2485208
226	C	-1.0878385	3.3651917	-2.1436718
227	H	-0.5073537	4.1851268	-2.5578843
228	H	-2.9932563	4.1855613	-2.7448543
229	H	-4.2957968	2.2851797	-1.8467902
230	H	0.6453994	2.2842312	-1.4749515

References

(1) Suzuki, K.; Kobayashi, A.; Kaneko, S.; Takehira, K.; Yoshihara, T.; Ishida, H.; Shiina, Y.; Oishi, S.; Tobita, S. Reevaluation of Absolute Luminescence Quantum Yields of Standard Solutions Using a Spectrometer with an Integrating Sphere and a Back-Thinned CCD Detector. *Phys. Chem. Chem. Phys.* **2009**, *11* (42), 9850. DOI: 10.1039/b912178a.

(2) Frisch, M. J.; Trucks, G. W.; Schlegel, H. B.; Scuseria, G. E.; Robb, M. A.; Cheeseman, J. R.; Scalmani, G.; Barone, V.; Petersson, G. A.; Nakatsuji, H.; Li, X.; Caricato, M.; Marenich, A. V.; Bloino, J.; Janesko, B. G.; Gomperts, R.; Mennucci, B.; Hratchian, H. P.; Ortiz, J. V.; Izmaylov, A. F.; Sonnenberg, J. L.; Williams-Young, D.; Ding, F.; Lipparini, F.; Egidi, F.; Goings, J.; Peng, B.; Petrone, A.; Henderson, T.; Ranasinghe, D.; Zakrzewski, V. G.; Gao, J.; Rega, N.; Zheng, G.; Liang, W.; Hada, M.; Ehara, M.; Toyota, K.; Fukuda, R.; Hasegawa, J.; Ishida, M.; Nakajima, T.; Honda, Y.; Kitao, O.; Nakai, H.; Vreven, T.; Throssell, K.; Montgomery, J. A. J.; Peralta, J. E.; Ogliaro, F.; Bearpark, M. J.; Heyd, J. J.; Brothers, E. N.; Kudin, K. N.; Staroverov, V. N.; Keith, T. A.; Kobayashi, R.; Normand, J.; Raghavachari, K.; Rendell, A. P.; Burant, J. C.; Iyengar, S. S.; Tomasi, J.; Cossi, M.; Millam, J. M.; Klene, M.; Adamo, C.; Cammi, R.; Ochterski, J. W.; Martin, R. L.; Morokuma, K.; Farkas, O.; Foresman, J. B.; Fox, D. J. *Gaussian 16, Revision B.01*; Gaussian, Inc., Wallingford CT, **2016**.

(3) Dolomanov, O. V.; Bourhis, L. J.; Gildea, R. J.; Howard, J. A. K.; Puschmann, H. OLEX2 : A Complete Structure Solution, Refinement and Analysis Program. *J. Appl. Crystallogr.* **2009**, *42* (2), 339–341. DOI: 10.1107/S0021889808042726.

(4) Sheldrick, G. M. SHELXT – Integrated Space-Group and Crystal-Structure Determination. *Acta Crystallogr. Sect. A Found. Adv.* **2015**, *71* (1), 3–8. DOI: 10.1107/S2053273314026370.

(5) Sheldrick, G. M. Crystal Structure Refinement with SHELXL. *Acta Crystallogr. Sect. C Struct. Chem.* **2015**, *71* (1), 3–8. DOI: 10.1107/S2053229614024218.

(6) Shimajiri, T.; Kawakami, Y.; Kawaguchi, S.; Hayashi, Y.; Hada, K.; Suzuki, T.; Ishigaki, Y. Ultralong C(sp3)–C(sp3) Single Bonds Shortened and Stabilized by London Dispersion. *Synlett* **2022**, in press. DOI: 10.1055/a-1934-1346.

(7) Raasch, M. S. Monothioanthraquinones. *J. Org. Chem.* **1979**, *44* (4), 632–633. DOI: 10.1021/jo01318a034.
